# A Rapid Evolving microRNA Cluster Rewires Its Target Regulatory Networks in *Drosophila*

**DOI:** 10.3389/fgene.2021.760530

**Published:** 2021-10-28

**Authors:** Yang Lyu, Zhongqi Liufu, Juan Xiao, Tian Tang

**Affiliations:** State Key Laboratory of Biocontrol and Guangdong Key Laboratory of Plant Resources, School of Life Sciences, Sun Yat-sen University, Guangzhou, China

**Keywords:** new miRNA, miRNA cluster, miRNA evolution, regulatory network, testis

## Abstract

New miRNAs are evolutionarily important but their functional evolution remains unclear. Here we report that the evolution of a microRNA cluster, *mir-972C* rewires its downstream regulatory networks in *Drosophila*. Genomic analysis reveals that *mir-972C* originated in the common ancestor of *Drosophila* where it comprises six old miRNAs. It has subsequently recruited six new members in the *melanogaster* subgroup after evolving for at least 50 million years. Both the young and the old *mir-972C* members evolved rapidly in seed and non-seed regions. Combining target prediction and cell transfection experiments, we found that the seed and non-seed changes in individual *mir-972C* members cause extensive target divergence among *D. melanogaster*, *D. simulans*, and *D. virilis*, consistent with the functional evolution of *mir-972C* reported recently. Intriguingly, the target pool of the cluster as a whole remains relatively conserved. Our results suggest that clustering of young and old miRNAs broadens the target repertoires by acquiring new targets without losing many old ones. This may facilitate the establishment of new miRNAs in existing regulatory networks.

## Introduction

Newly evolved genes constitute at least 10–20% of the genome in every taxonomic group (Khalturin et al., 2009; Kondo et al., 2017), and they play a significant role in the innovations of biological traits (Kaessmann, 2010; Chen et al., 2013). Increasing evidence suggests that a large fraction of the new genes are functionally important (Kaessmann, 2010; McLysaght and Hurst, 2016; Kondo et al., 2017; Xia et al., 2021). For instance, they are primarily expressed in testes, and are often involved in reproductive functions including male fertility (Gubala et al., 2017; Kondo et al., 2017; Lange et al., 2021), sperm competition (Yeh et al., 2012), courtship (Dai et al., 2008), and pheromone metabolism (Zhang J. et al., 2004). The functional importance of these testes-biased genes is also supported by the prominent signatures of positive selection at these loci (Zhao et al., 2014). Despite the evolutionary significance of the new genes, we know few about the mechanisms through which these novel elements integrated into the regulatory networks. Transcriptomic and protein-protein interaction studies suggest that the targets of some new genes changed dramatically even among closely related species (Chen et al., 2012; Ross et al., 2013), yet the underlying mechanisms are unclear.

While the techniques involved in determining the targets of young protein-coding genes are challenging, it is feasible to predict the targets of newly evolved microRNAs (miRNAs). miRNAs are a class of ubiquitous post-transcriptional regulators that participate in diverse biological processes in eukaryotes (Bartel, 2004; DeVeale et al., 2021). In animals, mature miRNAs (about 22 nt long) prevent the protein accumulation of the targets by either repressing translation or inducing mRNA degradation, through binding to the 3′ untranslated region (3′ UTR) of the transcripts with their seed region (the 2nd–8th nucleotides of the mature sequence) (Bartel, 2009, 2018). Collectively, miRNAs have broad impacts on the transcriptome, as each of them potentially have hundreds of targets (Agarwal et al., 2015). Their effects on individual targets, however, are usually weak (Baek et al., 2008; Selbach et al., 2008). Even for the most highly-expressed miRNAs, the repression effects on individual targets are usually less than 50% (Guo et al., 2010; Stadler et al., 2012).

Using next-generation sequencing techniques, previous studies have identified a large cohort of new miRNAs across taxa (Berezikov, 2011). In *Drosophila*, we have reported that the birth and death of miRNAs is extremely rapid (Lyu et al., 2014). It has been shown that over 40% of the miRNAs are only observed in the specific lineages (Lyu et al., 2014). Among these evolutionarily young miRNAs, 95% of them likely arose from scratch, as their seeds and precursors are different from that of the existing miRNAs (Lyu et al., 2014). It appears that these newly-evolved miRNAs have introduced a wide array of novel miRNA-mRNA interactions. Similar to new protein-coding genes, young miRNAs are inclined to express in testes, and they exhibit strong signatures of positive selection (Lyu et al., 2014; Mohammed et al., 2014). Understanding the mechanisms through which new miRNAs and targets evolve will provide key insights into the evolutionary processes of new genes. For example, how novel components originated and integrated into biological networks.

Our previous study has identified a *Drosophila*-specific miRNA cluster that we refer as *mir-972C* (Lyu et al., 2014). In *Drosophila melanogaster*, it consists of at least 12 miRNA members including *mir-972,* making it the largest new miRNA cluster in this species by far (Marco et al., 2013; Lyu et al., 2014; Mohammed et al., 2018). We speculated that the *mir-972C* is evolutionarily important, as it is highly expressed in testes and its DNA sequences exhibit strong signal of positive selection (Lyu et al., 2014). To understand how newly-evolved miRNAs influence gene regulatory networks, we investigated the evolution of miR-972C sequences and the regulatory networks within *Drosophila*. We found that not only the members of *mir-972C* vary across species, their sequences also undergo rapid changes, which cause the evolution of the target repertoire. In the end, we discussed the selective forces that may drive the evolution of new miRNA clusters in the long-term.

## Materials and Methods

### Genomic Data

*mir-972C* sequences and coordinates were obtained from miRBase (mirbase.org, Release 22.1) (Kozomara et al., 2019). Genome sequences were retrieved from UCSC (genome.ucsc.edu). The genome versions used here are: *D. melanogaster*, dm6; *D. simulans*, droSim1; *D. sechellia*, droSec1; *D. yakuba*, droYak2; *D. erecta*, droEre2; *D. ananassae*, droAna3; *D. pseudoobscura*, dp4; *D. virilis*, droVir3; *Anopheles gambiae*, MOZ2; *Apis mellifera*, Amel_2.0. GTF annotation files, 3′UTR sequences and 3′UTR locations were downloaded from flyBase (flybase.org, file version: *D. melanogaster*, r6.17; *D. simulans*, r2.02; *D. virilis*, r.1.06). Small RNA and mRNA testes deep-sequence libraries from *D. melanogaster*, *D. simulans, D. pseudoobscura*, and *D. virilis* (Czech et al., 2008; Rozhkov et al., 2010; Brown et al., 2014; Lyu et al., 2014; Ahmed-Braimah et al., 2017; Zhao et al., 2018) were retrieved from the GEO database (GEO accession IDs are listed in Supplementary Table S1).

### miRNA Homolog Search, Reads Validation, and Phylogenetic Inference

We searched for *mir-972C* sequences in the *Drosophila* genomes using BLAT (Kent, 2002) with default parameters and an E-value threshold of 0.001. Homologs of the *mir-972C members* in each species were identified using BLAST (Altschul et al., 1990) with queries of the known precursor sequences (miRBase Release 22.1) and an E-value threshold of 0.001. miRNA homologs from different species were aligned using MUSCLE (Edgar, 2004) with default parameters. To validate these miRNAs in *D. melanogaster*, *D. simulans*, D*. pseudoobscura*, and *D. virilis*, we used miRDeep2 (Friedländer et al., 2012) to map the small RNA sequencing reads (see Supplementary Table S1 for the information of the libraries) back to the genomic sequences of the entire cluster with default parameters. We used five standards that derived from a publication (Fromm et al., 2015) to validate miRNAs: 1) at least one miR* read; 2) at least 20 reads mapping to miR and miR* in total; 3) a hairpin structure with at least 13 paired nucleotides in miR:miR* duplex; 4) The top 3 iso-miR reads account for 85% of the miR arm reads and 5) the miR:miR* duplex to background reads ratio is >1. A maximum parsimony analysis was used to infer the origination of the *mir-972C* members by assuming that a miRNA emerged in the most recent common ancestor of all species bearing verified homologs.

### Target Evolution and Functional Analyses

We predicted the target sites using TargetScan (Lewis et al., 2005). “8mer” and “7mer-8A” targets were used for the following analyses. To select testes-expressed genes, we used the published testes RNA-seq data (Supplementary Table S1) and mapped the reads to the genome using STAR (parameters: -runThreadN 4 -runMode genome Generate) (Dobin et al., 2013). Read counts at gene level were calculated by counting all the reads that overlapped any exon for each gene using featureCounts (Liao et al., 2014) followed by TPM (Transcripts Per Kilobase Million) normalization. Genes with an average TPM (across multiple replicates) <  1 were removed from the following analyses. Overlapping targets of the different *Drosophila* species were visualized using BioVenn (Hulsen et al., 2008). We predicted the biological functions of the targets with the DAVID Functional Annotation Clustering analysis (v6.7, david.ncifcrf.gov) (Huang et al., 2009). Categories showing a significant enrichment (adjusted *p*-value < 0.05) are listed in Supplementary Table S2.

### *In vitro* Validation of miR-975 Targets

To construct *pUAST-mir-975* plasmids from each species, we amplified *mir-975* genes from the genomic sequences of *D. melanogaster* (ISO-1), *D. simulan*s (simNC48S), and *D. virilis* (V46) and cloned the fragments into a *pUAST* vector (see Supplementary Table S3 for primers and restriction sites). PCR reactions were carried out using the EX-Taq DNA Polymerase (TAKARA). Cells were transfected in a 48-well plate with 100 ng of *ub-GAL4* and 200 ng of conspecific *pUAST-mir-975* or the control vector (*pUAST* only) using Lipofectamine 2000 (Thermo Fisher Scientific, catalog no.12566014). Samples were collected 48 h after transfection.

Total RNAs were extracted from the samples using TRIzol (Thermo Fisher Scientific, catalog no. 15596026) for qRT-PCR and RNA-seq analyses. To quantify miRNA expression, total RNAs were reverse-transcribed into cDNAs using stem-loop reverse transcription and analyzed using the TaqMan qRT-PCR method following the miRNA UPL (Roche Diagnostics) probe assay protocol (He et al., 2016). The 2S RNA was used as the endogenous control (see Supplementary Table S4 for the qRT-PCR primers). cDNA libraries for each RNA sample were sequenced using the Illumina HiSeq 2000 at the Beijing Genomics Institute (Shenzhen). Reads were mapped to the genomes using TopHat (v.1.3.1) with a parameter -r 20 (Trapnell et al., 2009). Gene expression was estimated by FPKM (Fragments Per Kilobase per Million) using Cufflinks (v.2.1.1) with default parameters (Trapnell et al., 2010). Differentially expressed genes were determined using Cuffdiff (v.2.1.1) with default parameters (Trapnell et al., 2010). Non-expressed genes (FPKM = 0) were removed from the further analyses.

To confirm the *trans* effects of miRNA evolution on target repression, we expressed *dme-mir-975* and *dsi-mir-975* in S2 cells, respectively, and measured the expression of nine predicted targets of *dme-mir-975*. gDNA was removed from total RNAs by using TURBO DNA-free kit (Thermo Fisher Scientific, catalog no. AM 1907). Total RNAs were transcribed into cDNAs with the PrimeScript first strand cDNA synthesis kit (TAKARA Bio, catalog no. 6110A) and followed by a qPCR analysis using the SYBR Premix Ex Taq II kit (TAKARA Bio, catalog no. RR82WR). *rp49* was used as an internal control. Primers used are listed in Supplementary Table S5.

## Results

### Gain and Loss of Members in a Fast-Evolving miRNA Cluster

**FIGURE 1:**
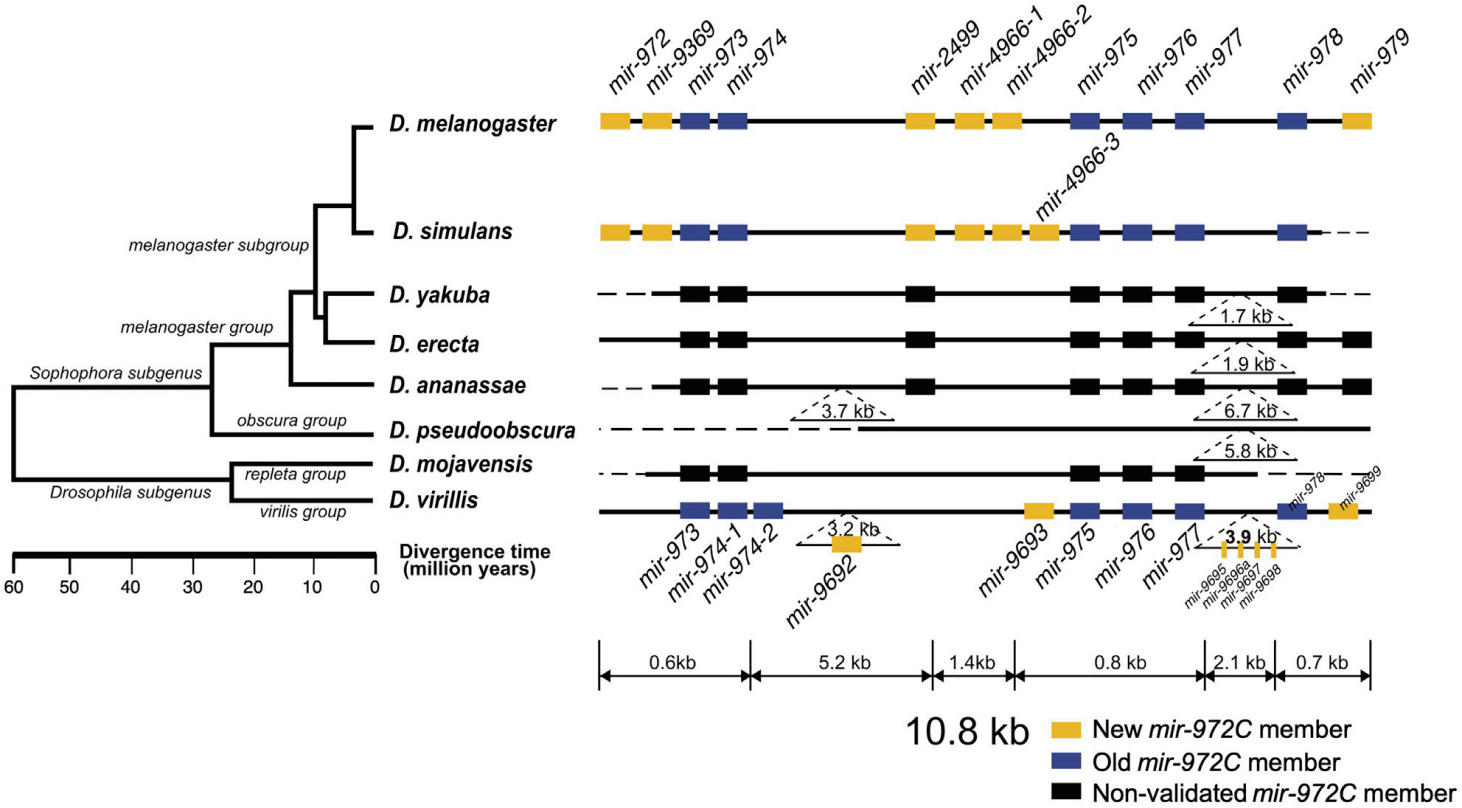
Evolutionary history of the *mir-972* cluster (*mir-972C*) in *Drosophila*. This cluster includes twelves miRNAs with distinct seeds. New (yellow boxes) and old (blue boxes) *mir-972C* members validated through deep sequencing (see Methods) are shown. miRNA homologs are colored in black. Deletions and insertions observed in sequence alignments are represented by dashed lines and inverted triangles. The genomic region is not drawn to scale. The phylogenetic tree was previously reconstructed based on whole-genome sequences (Clark et al., 2007).

The *mir-972C* cluster of *D. melanogaster* comprises 12 miRNAs spanning a 10.8-kb region located in the 18C-D band of the X chromosome. Based on the genomic proximity among members, the cluster was further divided into three sub-clusters (*mir-972/9369/973/974*, *mir-4966-1/4966-2/975/976/977*, *mir-978/979*), spanning less than 1 kb of each, along with an orphan miRNA *mir-2499* (Figure 1). The *mir-972C* members most likely originated *de novo*, as no sequence similarity was found either between the cluster members except for the *mir-4966* duplicates, or between them and other miRNAs that have been characterized in *D. melanogaster* (BLAST search, E < 0.001).

To investigate the origin and evolution of *mir-972C*, we searched for the orthologs of these miRNA genes in other *Drosophila* species, and in *Aedes* (mosquito) and *Apis* (honey bee) which have diverged from *Drosophila* 250 and 300 million years ago, respectively (Honeybee Genome Sequencing Consortium 2006; Yeates and Wiegmann, 1999). We found homologous sequences in all the seven *Drosophila* genomes surveyed (*D. simulans*, *D. yakuba*, *D. erecta*, *D. ananassae*, *D. pseudoobscura*, *D. mojavensis*, and *D. virilis*), but failed to detect any homologs in the mosquito or the honey bee genomes. This result indicates that *mir-972C* most likely emerged in the common ancestor of *Drosophila* between 60 and 250 million years ago. After origination, individual members of *mir-972C* have undergone rapid birth and death. In the *D. pseudoobscura* genome, the homologous sequences of *mir-2499* and *mir-979* were identified. Using the conspecific testes library, we were unable to detect the expression of these sequences, suggesting the loss of the entire cluster in this species (Figure 1). The distribution of individual miRNAs also varies across the remaining species. For example, *mir-973/974/975/976/977/978* sequences are represented in all the species except *D. pseudoobscura*, while other miRNAs have been lost in various lineages (Figure 1).

To date the origin time of each miRNA, we validated the expression of individual *mir-972C* members in the *Drosophila* species where small RNA sequencing data from testes were available, including: *D. melanogaster*, *D. simulans*, *D. pseudoobscura*, and *D. virilis* (see Supplementary Table S1 for data information). Using the recently proposed criteria for miRNA annotation (Fromm et al., 2015), we found that *mir-973/974/975/976/977/978* are expressed in *D. melanogaster*, *D. simulans*, and *D. virilis*; *mir-972/9369/2499/4966* are expressed in *D. melanogaster* and *D. simulans*, *mir-979* is only expressed in *D. melanogaster*, and *mir-9692/9693/9695/9696a/9697/9698/9699* only exist in *D. virilis* (see Supplementary Data for arm occupancy). miRNA expression in *D. pseudoobscura* was not detected.

Taken together, these results indicate that *mir-972C* initially originated in the common ancestor of *Drosophila* and subsequently diverged among different clades. Although the cluster originated more than 60 million years ago, the youngest member, *mir-979*, emerged in the recent 4 million years. Based on the phylogeny, we classified the miRNAs into the new members that originated after the *Sophophora*/*Drosophila* split (*mir-972/9369/2499/4966/979*) and the old members that arose before that event (*mir-973/974/975/976/977/978*) (Figure 1).

### Evolution of miR-972C Targets Mediated by Seed Innovation

**FIGURE 2:**
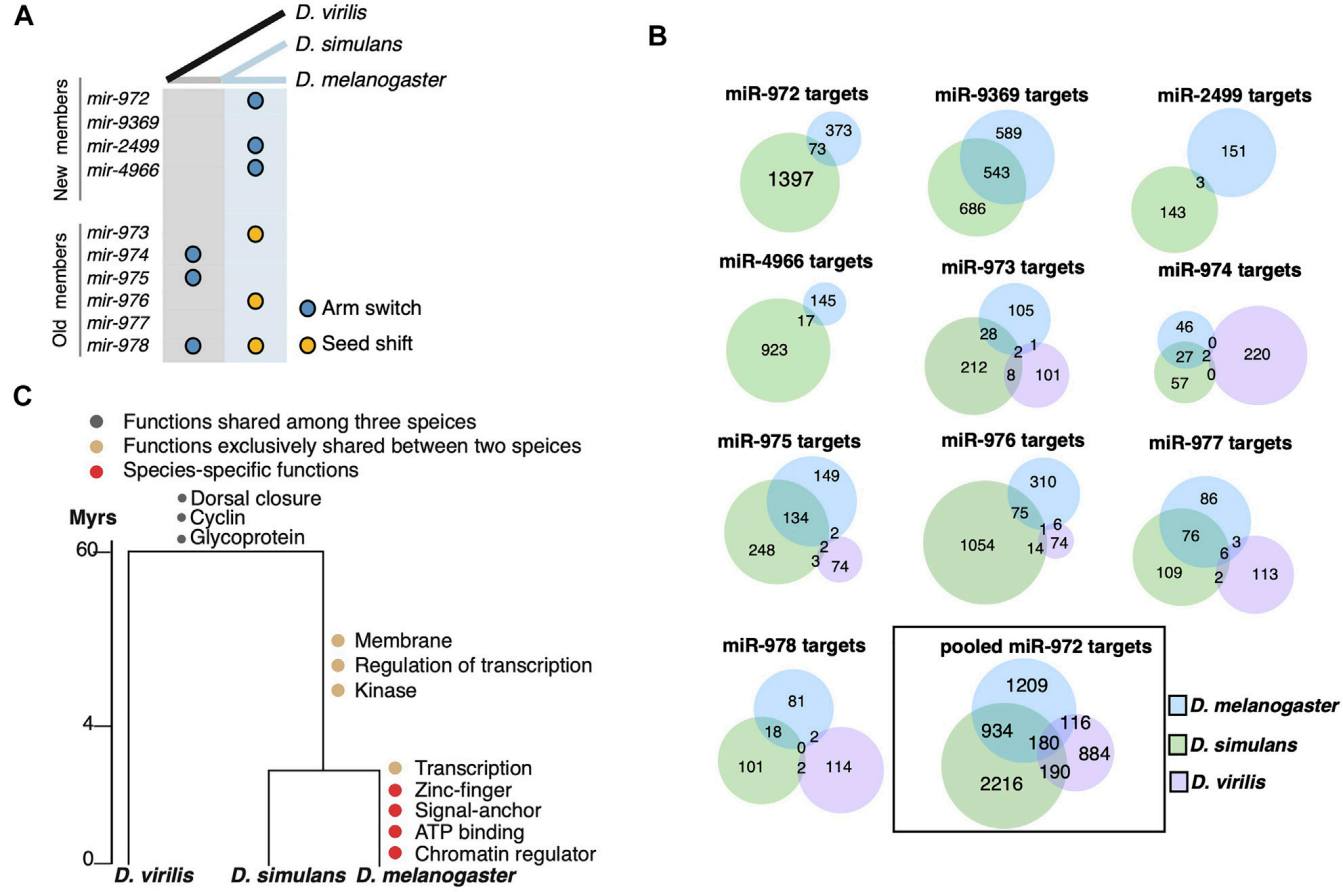
Evolution of miR-972C targets. **(A)** *miR-972C* seed innovations. Arm switching (blue circles) and seed shifts (yellow circles) were inferred and denoted along ancestral (grey) and recent (light blue) branches. **(B)** Venn diagrams depict the number of shared targets of individual miR-972C member targets or pooled cluster targets. **(C)** Functional evolution of targets. GO categories of shared and lineage-specific targets are indicated on the corresponding evolutionary branches.

After showing the evolution of the *mir-972C* members, we continued to investigate the sequence changes in individual miRNAs among *D. melanogaster*, *D. simulans*, and *D. virilis*. We are particularly interested in the alterations in the seed sequences as they are responsible for target recognition. The precursor alignments (Supplementary Figure S1) reveal two types of seed changes: 1) seed shifting, in which the dominant mature miRNA is shifted due to the changes in Drosha and Dicer processing (e.g., *mir-976*), and 2) arm switching, in which the mature miRNA switches to the other arms of the precursor (e.g., *mir-975*). We further inferred the time that the these two types of events occurred on the phylogenetic tree (Figure 2A). As shown in Figure 2A, six of the nine events occurred after the split of *D. melanogaster* and *D. simulans* in the recent 4 million years. Both the new and the old *mir-972C* members are involved in this seed innovation. The arm switching of *mir-975* occurred after the split of *D. virilis* and *D. melanogaster*/*D. simulans* but it is unclear on which branch (Figure 2A). *mir-978* is the only member that experienced both seed shifting and arm switching, and its seed is different among all three species (Supplementary Figure S1).

Observing the significant changes in the seed region, we studied the evolution of miR-972C targets. Since *mir-975C* appears exclusively expressed in testes, we first excluded genes that are not expressed in this tissue from the target analysis. To this end, RNA-seq reads from the testes of *D. melanogaster*, *D. simulans*, and *D. virilis* (Brown et al., 2014; Ahmed-Braimah et al., 2017) were mapped to the conspecific genome and the number of reads within each gene was normalized to TPM (Transcript Per Million). After removing the genes whose expression was not supported by enough reads (TPM <1), we retained 11,149 genes in *D. melanogaster*, 11,832 in *D. simulans*, and 10,453 in *D. virilis* for further analyses. Overall, 37.9% of the transcripts expressed in testes were overlapped among the three species (see Supplementary Figure S2A for the numbers of overlapping and species-specific transcripts. 37.9% is as the proportion of the overlap targets relative to the total targets in the species). It has been reported that testes-expressed genes exhibit lineage-specific bursts of rapid evolution and positive selection (Haerty et al., 2007; Parsch and Ellegren, 2013), which likely to contributes to the limited overlap among the *Drosophila* species. Next, we searched for the miR-972C target sites on the 3′-UTR of these testes-specific transcripts using TargetScan (Agarwal et al., 2018). As shown in Figure 2B, the number of the overlapping targets between the *D. melanogaster*/*D. simulans* branch and *D. virilis* is extremely small (<10 for each miRNA). Since TargetScan searches for miRNA target sites on the 3′UTR that pair with the seed sequences, target divergence among species is either due to the changes in the seed sequences themselves, or due to the alterations of miRNA binding sites on the 3′-UTRs. As for *mir-974* and *mir-977*, their seeds are fully conserved among the *Drosophila* species. Even so, their targets are not shared much among the species, suggesting significant changes in the miRNA binding sites on the 3′UTRs. Considering the sequences of testes-expressed genes have evolved much faster (Haerty et al., 2007; Parsch and Ellegren, 2013), the limited number of sharing targets is not completely surprising. In addition, 3′UTRs of testes-expressed genes are significantly shorter (Sanfilippo et al., 2017), which makes them less likely to be targeted by miRNAs. The 3′UTR shortening might contribute to the low number of sharing targets as well. These results suggest that 3′UTR divergence also plays an important role in the target evolution of miR-972C.

We took a closer look at the target divergence between *D. melanogaster* and *D. simulans*. While the *mir-9369/974/975/977* seeds are identical between these species (Supplementary Figure S1), the proportion of overlapping targets ranges from 22.0 to 29.9% (Figure 2B). Both seed shifting and arm switching significantly reduced the overlap: only 1.0–4.0% of targets are shared between the two species after arm switching (*mir-972/2499/4966,* Figure 2B), and 5.2–8.8% are shared after seed shifting (*mir-973/976/978*) (Figure 2B). Although the number of overlapping targets between *D. melanogaster* and *D. simulans* was small for each miRNA after seed changes, the overlap in targets for the entire cluster (22.8%) was largely comparable with that of the miRNAs with identical seeds (Figure 2B). This is likely because a 3′UTR targeted by a *mir-972C* member in one species can be targeted by a different member in another. These observations support the idea that although the targets of each miRNA evolve rapidly, the entire miRNA cluster keeps a relatively conserved target pool.

To understand the biological consequences of miR-972C target evolution*,* we examined the functional enrichment of the predicted targets on each evolutionary branch using DAVID (Huang et al., 2009). Mutual targets that are shared among the three species are enriched in “dorsal closure” (*p* = 1.0E-05), “Cyclin” (*p* = 0.046) and “Glycoprotein” (*p* = 0.039), indicating a possible role of this cluster in the common ancestor (Figure 2C, Supplementary Table S2). After the split of *D. virilis* and the *D. melanogaster/D. simulans* branch, we observed a burst of new GO categories on the *D. melanogaster/D. simulans* branch and also in *D. melanogaster* (i.e., regulation of transcription, *p* = 0.027), consistent with the increase in the target number of this lineage (Figure 2B). Interestingly, *D. melanogaster* continued to gain new targets that are involved in “transcription” (*p* = 1.1E-03), suggesting a reinforcement of the ancestral functions. These results indicate that the evolution of miR-972C targets may contribute to functional innovation between species.

### Both Seed and Non-seed Mutations Contributed to the Target Evolution of miR-975

Among the members of miR-972C, miR-975 is especially intriguing as it has distinct effects on male reproduction between *D. melanogaster* and *D. simulans* (Zhao et al., 2021), and thus offers an opportunity to study the functional evolution of miRNAs. *mir-975* has undergone substitutions in both seed and non-seed regions (Figure 2A, Supplementary Figure S1). The *mir-975* seed in *D. virilis* is completely different from those in *D. melanogaster* and *D. simulans* as a result of arm switching (Figure 2A, Supplementary Figure S1). Furthermore, there is a single nucleotide substitution right next the seed region in the mature miR-975 sequences between *D. melanogaster* and *D. simulans* (Supplementary Figure S1).

**FIGURE 3:**
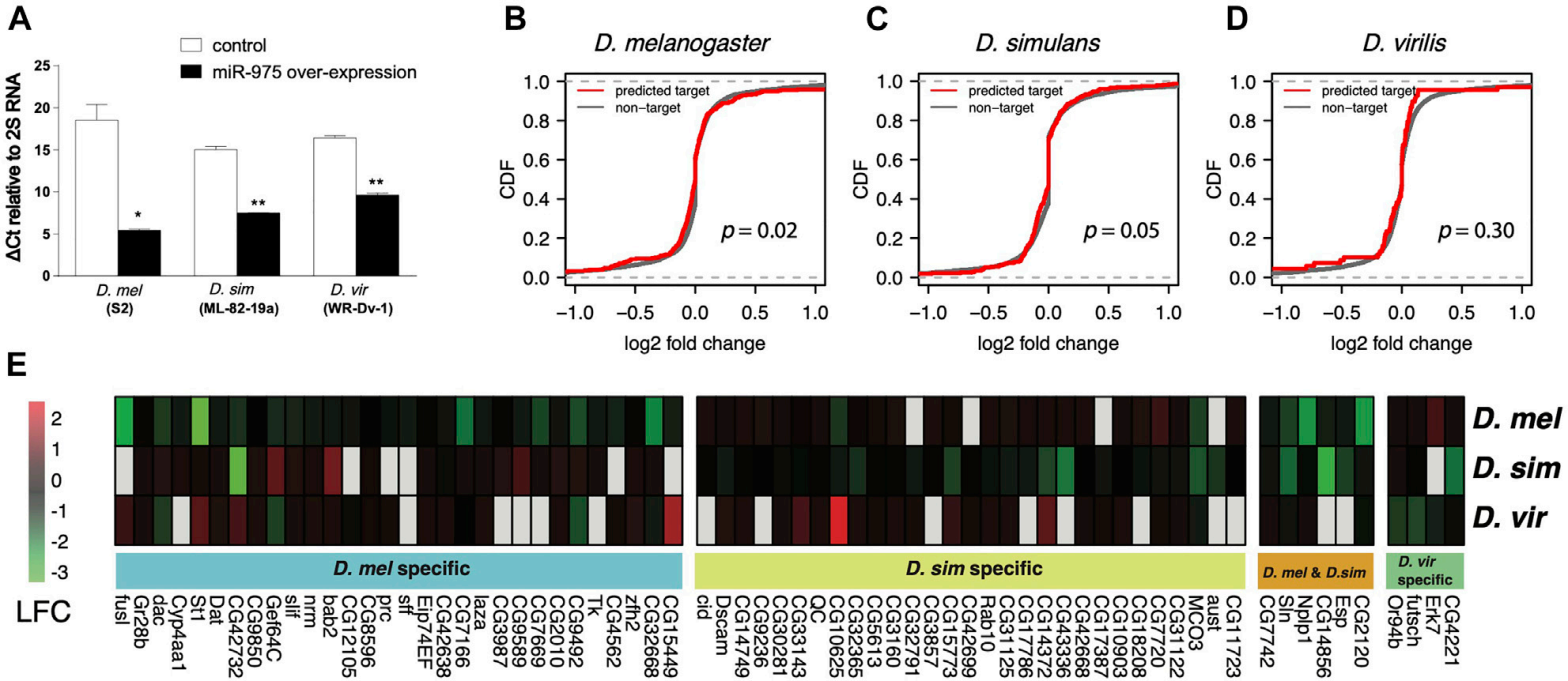
*In vitro* validation of *miR-975* target divergence. **(A)** Relative miR-975 expression levels in *D. melanogaster* (S2), *D. simulans* (ML-82-19a), and *D. virilis* (WR-Dv-1) cell lines after transfection with the pUAST-only vector (control) or conspecific pUAST-mir-975. The bar plot shows ΔCt values (Ct_miR-975_—Ct_2S RNA_) from the qRT-PCR assays. **(B–D)** Effect of *miR-975* on predicted targets and non-targets in cell lines derived from *D. melanogaster* **(B)** *, D. simulans* **(C)**, and *D. virilis* **(D)**. (CDF: cumulative distribution function. *p*-values from the Kolmogorov-Smirnov test are shown). **(E)** Heatmap shows the log2 fold change (LFC) of validated *miR-975* targets in the three species.

To understand the impact of *mir-975* sequence evolution on its target repertoire, we overexpressed the conspecific *mir-975* sequences in cells derived from *D. melanogaster* (S2), *D. simulans* (ML-82-19a), and *D. virilis* (WR-Dv-1) and monitored the expression changes of both miR-975 itself and the transcriptome as a whole. Quantitative PCR confirmed that miR-975 was only expressed in cells transfected with the *pUAST-mir-975* vector but not in cells transfected with the control *pUAST* vector (Figure 3A). Venn diagram shows that the vast majority of the transcripts expressed in the three cell lines were overlapped (Supplementary Figure S2B). When miR-975 was overexpressed, predicted targets were significantly down-regulated compared to the transcriptomes of the *D. melanogaster* and the *D. simulans* cells (Figures 3B,C, Kolmogorov-Smirnov test, both *p* < 0.05). The repression magnitude is small, consistent with the weak repression from miRNAs (Zhao et al., 2017; Chen et al., 2019). Target repression is not significant in *D. virilis* cells (Kolmogorov-Smirnov test, *p* = 0.41, Figure 3D), probably because there are only a few predicted targets in this species (*n* = 65).

Using 1.2-fold repression as a cut-off (Wen et al., 2011), we found 36 targets that were down-regulated in *D. melanogaster*, 35 in *D. simulans*, and four in *D. virilis* (Figure 3E). As expected, none of these targets were shared between *D. virilis* and the other two species. We also confirmed arm switching of dvi-miR-975 in WR-Dv-1 cells using qPCR (Supplementary Figure S3). These results indicated that the target pool was completely changed by arm switching. Between *D. melanogaster* and *D. simulans,* although the seed of miR-975 is identical, only six (9.2%) of the down-regulated targets are identical (Figure 3E). Taken together, our *in vitro* experiments demonstrate that both seed and non-seed changes in *mir-975* contribute to the evolution of target regulatory networks among the *Drosophila* species.

### *D. simulans* miR-975 Has a Weaker Effect on the *D. melanogaster* Transcriptome Than the Conspecific miR-975

**FIGURE 4:**
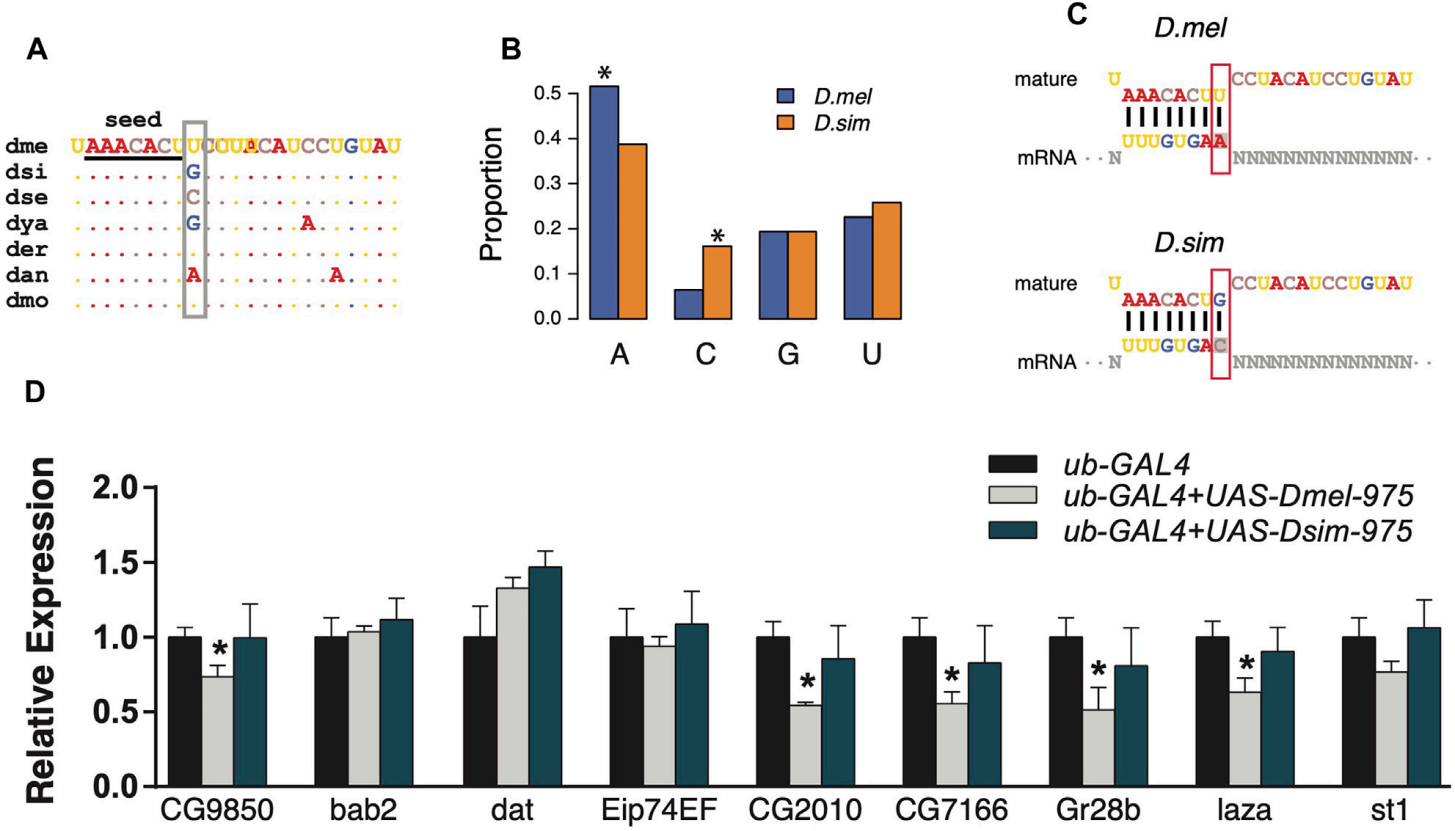
Divergent regulation effects of dme-miR-975 and dsi-miR-975 in S2 cells. **(A)** miR-975 mature sequences from seven *Drosophila* species. They share the same seed (underlined), but their 9th nucleotide (grey box) varies among species. Species abbreviations: dme, *D. melanogaster*; dsi, *D. simulans*; dse, *D. sechellia*; dya, *D. yakuba*; der, *D. erecta*; dan, *D. ananassae*; dmo, *D. mojavensis*. **(B, C)** Bar plot demonstrates that the 3′UTR sites bound to the 9^th^ base of mature sequences are enriched for A in *D. melanogaster* but are enriched for C in *D. simulans* (see **C** for the sequence match). **(D)** qPCR results validated that evolution of the 9^th^ nucleotide led to the differential regulation of dme-miR-975 and dsi-miR-975 in S2 cells.

Since the evolution of miRNA sequences and 3′UTRs could both contribute to the changes in miRNA targeting (Hausser and Zavolan, 2014), we next investigated the trans effects of miR-975 on the *D. melanogaster* transcriptome. As shown in Supplementary Figure S1, the ninth base of the *mir-975* mature sequences underwent a transversion (G ->U) after the split of *D. melanogaster* and *D. simulans* (Figure 4A). As a result, target sites complementary to the ninth base of the *mir-975* mature sequence were enriched for adenine (A) in *D. melanogaster* but enriched for cytosine (C) in *D. simulans* (*p* < 0.05, Fisher’s exact test, Figures 4B,C). To determine the effects of this mutation, we conducted reporter assays by transferring either the *dme-mir-975* or the *dsi-mir-975* fragment to the *D. melanogaster* S2 cells, along with a ub-Gal4 driver (see Materials and Methods). qPCR assay shows that both dme-miR-975 and dsi-miR-975 were highly expressed in the S2 cells after transfection (Supplementary Figure S4), and miR-975 was not detected in the S2 cells transfected with the ub-Gal4 control. We examined the expression of nine *D. melanogaster* specific targets that have an “A” site complementary to the ninth base of the mature dme-miR-975. Six out of the nine targets were significantly down-regulated by overexpressing dme-miR-975 (*p* < 0.05, student’s t-test), whereas none was repressed by overexpressing dsi-miR-975 (Figure 4D), despite the expression level of dsi-miR-975 was much higher than dme-miR-975 (Supplementary Figure S4). These results suggest that even the innovation of non-seed region mediates significant changes in target repertoire. Importantly, since 70.6–86.0% of the transcripts that expressed (TPM ≥ 1) in the S2 cells were also detected in the testes (Supplementary Figure S2C), our *in vitro* analysis has strong implications on the evolution of miRNA target repertoire *in vivo*.

## Discussion

New genes continuously contribute to genetic novelty and offer a unique opportunity to understand the phenotypic divergence between species and the evolution of genetic regulatory networks (Tang et al., 2010; Chen et al., 2012; Ross et al., 2013; Zhao et al., 2021). As key players in gene regulation, miRNAs repress their targets weakly but broadly in animals (Zhao et al., 2017; Chen et al., 2019). However, it remains unclear how their functions have evolved, which might ultimately determine their evolutionary fate (Wu et al., 2009; Lyu et al., 2014; Penso-Dolfin et al., 2018). Some debates even have been focused on whether new miRNAs have biological functions at all (Nozawa et al., 2010). Here we show that the adaptive evolution of the *mir-972C* is accompanied by dramatic evolution of the target repertoires between distantly and closely related *Drosophila* species. Importantly, we found that changes in both seed and non-seed regions contribute to the evolution of the target pool. While the sequence evolution of each member has recruited new targets that represent novel functions in specific lineages, the vast majority of old targets are conserved when we consider the cluster as a whole. These results shed light on the survival and evolution of new genes in general.

Our results suggest that in a clustering form, new miRNAs may have a higher chance to survive and integrate into the regulatory networks (Zhang et al., 2007; Wang et al., 2016). Individual miRNAs, especially the evolutionarily young ones, have little effects on their targets (Sood et al., 2006; Chen and Rajewsky, 2007; Chen et al., 2019). However in a cluster, new miRNAs are co-expressed and function as a unit (Kabekkodu et al., 2018). As a result, the target pool is predicted to expand greatly. Indeed, while only 0.7–10.1% of the testes-specific transcriptome is potentially targeted by individual *mir-972C* members in *D. melanogaster* (Figure 2B), 21.1% can be influenced by the whole cluster together. Recent studies have shown that large miRNA target pools are evolutionarily beneficial in maintaining stability of gene expression through broad and weak regulation (Zhao et al., 2017; Chen et al., 2019). Consistent with this notion, a significant proportion of the *miR-972C* target pool remains unchanged (e.g., 22.8% of the targets are conserved between *D. melanogaster* and *D. simulans*, Figure 2B), despite arm switching and seed shifting occurred frequently between species.

Functional analysis of the target repertoire shows a reinforcement of the ancestral functional categories of miR-972C targets (Figure 2C). It also suggests that this miRNA cluster continues to recruit additional targets either through the evolution of existing miRNAs or the birth of new hairpins. Such processes may also bring novel functions. It is thus not unexpected that members of *mir-972C* does not follow the reported pattern for insect miRNAs that conserved miRNAs tend to express at a higher level and possess more targets than lineage-specific miRNAs (Chen and Rajewsky, 2007; Ylla et al., 2016). We found no significant difference in expression levels between young and old members of *mir-972C* in any of the three species on survey (all *p* > 0.05, Mann-Whitney U test, Supplementary Figure S5A). Old members have more targets than the young ones in *D. simulans* (*p* = 0.044, Mann-Whitney U test, Supplementary Figure S5B) but not in *D. melanogaster or D. virilis* (*p* > 0.05, Mann-Whitney U test, Supplementary Figure S5B). It is likely that new miRNAs in this cluster have quickly increased their expression levels and recruited a large number of targets.

Fast-evolving targeting implies that these miRNAs have never been deeply integrated into the existing gene regulatory networks. The long-term survival of these novel miRNAs remains unclear. Previously we have shown that miR-975 exerts different influences on male fertility between *D. melanogaster* and *D. simulans*, and its loss-of-function might be adaptive in *some lineages* (Lu et al., 2018a, 2018b; Zhao et al., 2021). Another good example in *Drosophila* is the *mir-310/311/312/313* cluster (*mir-310C*), which is another adaptive miRNA cluster with a same age as *mir-972C* (Lu et al., 2008; Lyu et al., 2014). *mir-310C* is known to affect egg morphology, hatchability, and male fertility (Pancratov et al., 2013; Liufu et al., 2017). Redundant and incoherent regulation of multiple phenotypes by *mir-310C* suggests that these miRNAs play a role in stability control (Liufu et al., 2017). It is thus not surprising that the miRNA-target interactions could be readily changed. Unlike *mir-310C* that was duplicated from *mir-92a/b, mir-972C* seems to have evolved from non-functional sequences and transcribed specifically in testes (Marco, 2014; Mohammed et al., 2014). The cost of gene loss is more acceptable when the expression of the gene is restricted in fewer tissues (Fraïsse et al., 2019). For this reason, the loss of the entire *mir-972* cluster in the *D. pseudoobscura* lineage is not surprising (Figure 1).

It should be noted that as a testes-biased miRNA cluster, the fast evolution of *mir-972C* may be associated with the rapid turnover of cellular environments in this tissue. It is well established that testis is the most rapidly evolving tissue due to the selective forces associated with sperm competition, reproductive isolation, and sexual conflict (Kaessmann, 2010). Previous investigations in many taxa have demonstrated that male-biased genes evolve relatively quickly at both sequence and expression level (Meiklejohn et al., 2003; Zhang Z. et al., 2004; Ellegren and Parsch, 2007; Yang et al., 2016). Changes of chromatin states during spermatogenesis allow aberrant transcription which makes testis a hotspot for new gene origination (Kaessmann, 2010). This cellular environment may boost the evolutionary rate of genes with which it has co-evolved, including miRNAs (Tang et al., 2010; Wang et al., 2016). Interestingly, *mir-972C* targets do not show GO enrichment in male functions (Figure 2C), despite the testes-specific expression of this cluster (Mohammed et al., 2014; Lu et al., 2018b)*.* Why would *mir-972C* be beneficial to the male reproductive system? One plausible explanation is that the high complexity of the testes transcriptome (Vibranovski et al., 2009) that requires substantial regulations to stabilize the system (Wu et al., 2009). *mir-972C* would be an excellent candidate to do so as it is highly abundant and broadly tied to the testes transcriptome.

## Key Resources Table

**Table:** 

**Reagent or Resource**	**Source or Reference**	**Identifiers**
**Fly Strains**		
ISO-1 (*D. melanogaster*)	Flybase	FBsn0000272
simNC48S (*D. simulans*)	Flybase	FBst0201377
V46 (*D. virilis*)	Flybase	FBst0200545
**Cell lines**		
S2 (*D. melanogaster*)	DGRC	Stork #6
ML-82-19a (*D. simulans*)	DGRC	Stock #27
WR-Dv-1 (*D. virilis*)	DGRC	Stock #40
**Oligonucleotides**		
Primers for vector construction and qPCR	see Supplementary Table S3–S5	
**Plasmids**		
pUAST	DGRC: Vector	Barcode #1000
ub-Gal4	Liufu et al. (2017)	
**Commercial kits**		
EX-Taq DNA Polymerase	TAKARA	Catalog #RR001C
TRIzol	Thermo Fisher Scientific	Catalog #15596026
miRNA UPL probe	Roche Diagnostics	#21
TURBO DNA-free kit	Thermo Fisher Scientific	Catalog #AM1907
PrimeScript first strand cDNA synthesis kit	TAKARA	Catalog #6110A
Lipofectamine 2000	Thermo Fisher Scientific	Catalog #12566014
SYBR Premix Ex Taq II kit	TAKARA	Catalog #RR82WR
**Deposited data**		
RNA-seq data	see Supplementary Table S1	
**Software, Algorithms and Tools**		
BLAT	http://hgdownload.soe.ucsc.edu/admin/exe/	
BLAST	https://ftp.ncbi.nlm.nih.gov/blast/executables/blast+/LATEST/	
MUSCLE	https://www.drive5.com/muscle/downloads.htm	
TopHat	http://ccb.jhu.edu/software/tophat/downloads/	
miRDeep2	https://github.com/rajewsky-lab/mirdeep2/releases/tag/v0.1.3	
STAR	https://github.com/alexdobin/STAR	
DAVID	https://david.ncifcrf.gov/	
TargetScan	http://www.targetscan.org/fly_72/	

## Data Availability

The datasets presented in this study can be found in online repositories. The names of the repository/repositories and accession number(s) can be found below: https:https://www.ncbi.nlm.nih.gov/geo/query/acc.cgi?acc=GSE107390.
