# Supplementary material for: A Rapid Evolving microRNA Cluster Rewires Its Target Regulatory Networks in Drosophila
Source: Front Genet. 2021 Oct 28;12:760530. doi: 10.3389/fgene.2021.760530 (PMC8581666; doi:10.3389/fgene.2021.760530)
Supplement: Supplementary file 1 [file DataSheet1.ZIP › Supplementary Data.pdf]

miRBase precursor : dme-mir-972
Total read count : 90
dme-miR-972-5p read count : 1
dme-miR-972-3p read count : 89
remaining reads : 0

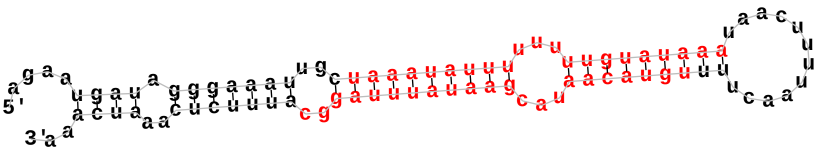

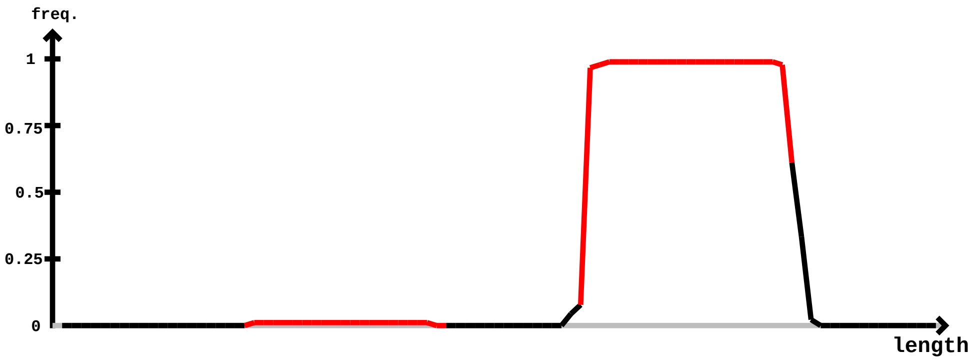

dme-miR-972-5p

dme-miR-972-3p

```
5'- agaaugauagggaaauugcuaaauauuuuuuuuguauaaauaacuuuuaacuuuuguacaauacgaauauuuaggcauuucucaaaucaaa   -3'    exp
    ....((((.(((((((..((((((((((...(((((((((...........)))))))))...))))))))))..))))))).))))..     reads    mm    sample
    ...................uaaauauuuuuuuuguaua.....................................................     1        0     seq
    ....................................................uuuguacaauacgaauauuuGgg................     1        1     seq
    ....................................................uuuguacaauacgaauauuuagg................     3        0     seq
    .....................................................uuguacaauacgaauauuuagg................     3        0     seq
    ......................................................uguacaauacgaauauuuag.................     1        0     seq
    ......................................................uguacaauacgaauauuuaUg................     1        1     seq
    ......................................................uguacaauacgaauauuuagg................     23       0     seq
    ......................................................uguGcaauacgaauauuuagg................     2        1     seq
    ......................................................uguacaauacgaauauuuaggc...............     25       0     seq
    ......................................................uAuacaauacgaauauuuaggca..............     1        1     seq
    ......................................................uguacaauacgaauauuuaggcU..............     2        1     seq
    ......................................................uguacaauacgaauauuuaggca..............     23       0     seq
    ......................................................uguacaauacgaauauuuaggcaA.............     1        1     seq
    ......................................................uguacaauacgaauauuuaggcau.............     1        0     seq
    .......................................................guacaauacgaauauuuaggca..............     1        0     seq
    ........................................................uacaauacgaauauuuaggca..............     1        0     seq
```

miRBase precursor : dme-mir-9369
Total read count : 667
dme-miR-9369-5p read count : 265
dme-miR-9369-3p read count : 401
remaining reads : 1

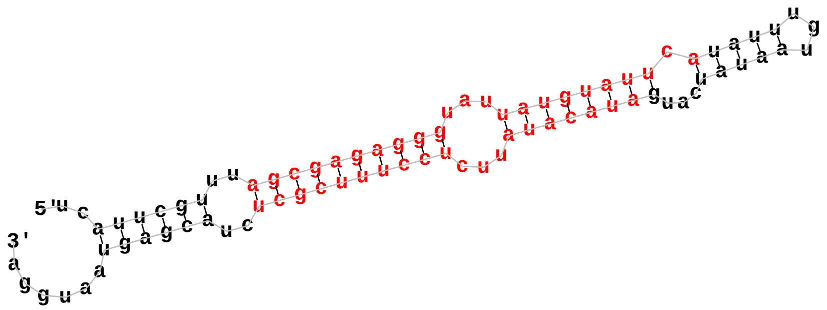

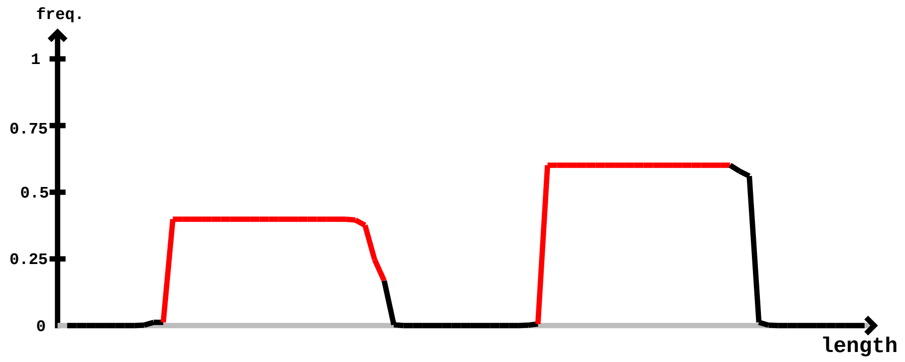


```
                                                  dme-miR-9369-3p
         dme-miR-9369-5p
5'- ucauucguuuagcgagaggguauuauguauucauauuuguaauaucaugauacauauucuccuuucgcucuacgaguaaugga   -3'   exp
    ..((((((..((((((((((...((((((((.(((((...)))))...))))))))...))))))))))..))))))......    reads    mm    sample
    .......uuuagcgagaggguauuaugua......................................................    1        0     seq
    ........uuagcgagagggGauuauguau.....................................................    1        1     seq
    ........uuagcgagaggguauuauguau.....................................................    6        0     seq
    .........agcgagaggguauuaugua.......................................................    1        0     seq
    .........agcgagaggguauuauguau......................................................    6        0     seq
    .........agcgagaggguauuauguauu.....................................................    77       0     seq
    .........agcgagagggGauuauguauu.....................................................    4        1     seq
    .........agcgagaggguauuauguauA.....................................................    1        1     seq
    .........agcgaCaggguauuauguauu.....................................................    1        1     seq
    .........agcgUgaggguauuauguauu.....................................................    1        1     seq
    .........agcgaAaggguauuauguauu.....................................................    1        1     seq
    .........agcgagagggAauuauguauu.....................................................    1        1     seq
    .........agcgGgaggguauuauguauuc....................................................    1        1     seq
    .........agcgagaggguauuauAuauuc....................................................    1        1     seq
    .........agcgagaggguUuuauguauuc....................................................    1        1     seq
    .........agcgagaggguauuauguauAc....................................................    1        1     seq
    .........agcgagaggguauuauguauuA....................................................    1        1     seq
    .........agcgagaggguauuauguauuU....................................................    1        1     seq
    .........agcgagaggguauuauguauuc....................................................    47       0     seq
    .........agcgagaggguauuauguauuGa...................................................    1        1     seq
    .........agUgagaggguauuauguauuca...................................................    1        1     seq
    .........agcgagaggguauuauguaAuca...................................................    1        1     seq
    .........agcgagagUguauuauguauuca...................................................    1        1     seq
    .........agcgagaggguauuauguauuca...................................................    100      0     seq
    .........agcgagagggGauuauguauuca...................................................    4        1     seq
    .........agcgagagggAauuauguauuca...................................................    1        1     seq
    .........agcgaAaggguauuauguauuca...................................................    1        1     seq
    .........agcgagaggguauuauguauucau..................................................    1        0     seq
    .........agcgagaggguauuauguauucaA..................................................    1        1     seq
    ...............................................ugauacauauucuccuuucgcu..............    1        0     seq
    ................................................gauacauauucuccuuucgcuc.............    1        0     seq
    ................................................gauacauauucuccuuucgcucu............    2        0     seq
    .................................................auaAauauucuccuuucgcu..............    1        1     seq
    .................................................auacauauucuccuuucgcu..............    13       0     seq
```

```
                                                 dme-miR-9369-3p
          dme-miR-9369-5p
ucauucguuuagcgagaggguauuauguauucauauuuguaauaucaugauacauauucuccuuucgcucuacgaguaaugga

.................................................auacauauucuccuuucgcuc.............    11    0    seq
.................................................auacauauucuccuuucgcuGu............    1     1    seq
.................................................auacauauucuccuuucgcucu............    347   0    seq
.................................................auacauauucuccuuucgGucu............    1     1    seq
.................................................auCcauauucuccuuucgcucu............    1     1    seq
.................................................auacauauucuccuuuGgcucu............    1     1    seq
.................................................auacauUuucuccuuucgcucu............    1     1    seq
.................................................auacauGuucuccuuucgcucu............    1     1    seq
.................................................auacauauucuccuuucgUucu............    1     1    seq
.................................................auacauauucuUcuuucgcucu............    1     1    seq
.................................................auacauauGcuccuuucgcucu............    1     1    seq
.................................................auacaGauucuccuuucgcucu............    1     1    seq
.................................................auacauauucuccuuucgcuUu............    1     1    seq
.................................................auacauauucuccuuuAgcucu............    1     1    seq
.................................................auacauauucucUuuucgcucu............    1     1    seq
.................................................Uuacauauucuccuuucgcucu............    1     1    seq
.................................................auacauauucuccuuucAcucu............    1     1    seq
.................................................aGacauauucuccuuucgcucu............    2     1    seq
.................................................auacauauucuccuuucgcucuU...........    5     1    seq
.................................................auacauauucuccuuucgcucua...........    2     0    seq
.................................................auacauauucuccuuucgcucuaA..........    1     1    seq
```

miRBase precursor : dme-mir-973
Total read count : 137
dme-miR-973-5p read count: 51
dme-miR-973-3p read count: 86
remaining reads : 0

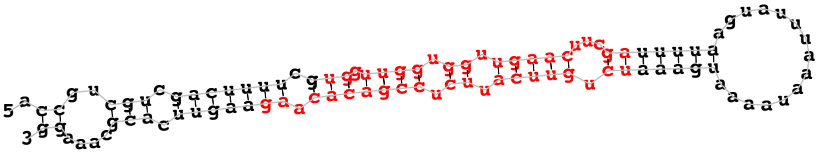

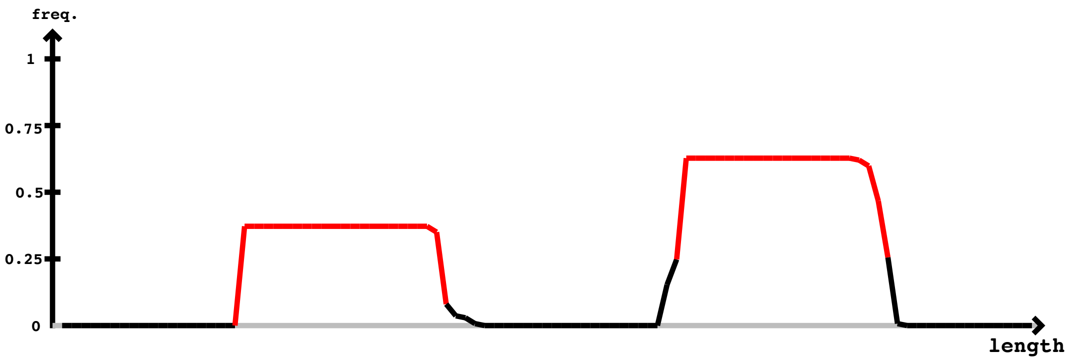

dme-miR-973-5p

dme-miR-973-3p

```
5'- accgucgucgacuuuucgugguuggugguugaacuucgauuuuaaguauuuaaauaaaaugaaaucuguucauucuccgacacaagaaguucacgcaaagg  -3'    exp
    .((..(((.(((((((.(((.((((.((.(((((...(((((((...............))))))).))))).)).))))))).))))))).)))....))  reads  mm  sample
    ..................ugguuggugguugaacuucg...............................................................  3      0   seq
    ..................ugguuggugguugaacuucga..............................................................  36     0   seq
    ..................ugguuggugguugaacuAcga..............................................................  1      1   seq
    ..................ugguuggugguugaacuucgaA.............................................................  2      1   seq
    ..................ugguuggugguugaacuucgau.............................................................  4      0   seq
    ..................ugguuggugguugaacuucgauu............................................................  1      0   seq
    ..................ugguuggugguugaacuucgauuu...........................................................  3      0   seq
    ..................ugguuggugguugaacuucgauuuu..........................................................  1      0   seq
    ..............................................................aaucuguucauucuccgaca...................  1      0   seq
    ..............................................................aaucuguucauucuccgacac..................  3      0   seq
    ..............................................................aaucuguucauucuccgacaca.................  15     0   seq
    ..............................................................aaucuguucauucuccgacacaa................  2      0   seq
    ...............................................................aucuguucauucuUcgacacaa................  1      1   seq
    ...............................................................aucuguucauucuccgacacGa................  1      1   seq
    ...............................................................aucuguucauucuccgacacaa................  9      0   seq
    ...............................................................aucuguucauucuccgacacaag...............  2      0   seq
    ................................................................ucuguucauucuccgacaca.................  3      0   seq
    ................................................................ucuguucauucuccgacacaa................  14     0   seq
    ................................................................ucuguucaAucuccgacacaa................  1      1   seq
    ................................................................ucAguucauucuccgacacaa................  1      1   seq
    ................................................................ucuguucauucuccgacacaag...............  27     0   seq
    ................................................................ucuguAcauucuccgacacaag...............  1      1   seq
    ................................................................ucuguucauucucGgacacaag...............  1      1   seq
    ................................................................ucuguucauucuccgacUcaag...............  1      1   seq
    ................................................................ucuguucauuAuccgacacaag...............  1      1   seq
    ................................................................ucuguucauucucUgacacaag...............  1      1   seq
    ................................................................ucuguucauucuccgacacaagU..............  1      1   seq
```

miRBase precursor : dme-mir-974
Total read count : 3796
dme-miR-974-5p read count : 3786
dme-miR-974-3p read count : 8
remaining reads : 2

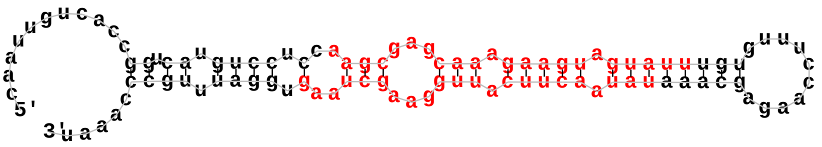

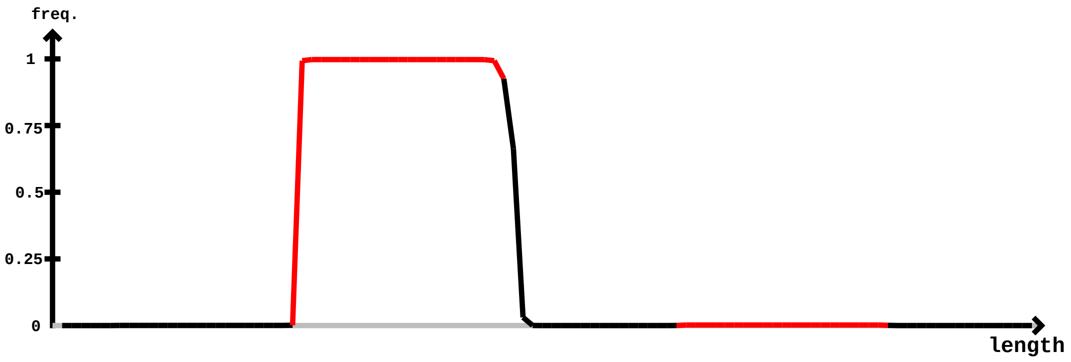

dme-miR-974-3p
dme-miR-974-5p

	reads	mm	sample
5'- caauugucaccggucauguccuccaagcgagcaaagaaguaguauuuguguuuccaagagcaaauauaacuucauuggaagcuaaguggauuugcccaaau -3'	exp		
...........((.((.((((.(..(((...(((.(((((.((((((((..........)))))))).))))).)))...)))..).)))).)).))....	reads	mm	sample
.....gucaccggucauguccucc...	2	0	seq
.......................caagcgagcaaagaaguaguau..	1	0	seq
.......................Uaagcgagcaaagaaguaguauu...	2	1	seq
........................aagcgagcaaagaaguagu..	1	0	seq
........................aagcgagcaaagaaguaguU...	2	1	seq
........................aagcgagcaaagaaguagua...	13	0	seq
........................aagcgagcaaagaagGaguau..	1	1	seq
........................aagcgagcUaagaaguaguau..	1	1	seq
........................aagcgagcaaagaaguaguau..	252	0	seq
........................aagUgagcaaagaaguaguau..	1	1	seq
........................aagcgagcaaagaaguaAuauu...	1	1	seq
........................aaUcgagcaaagaaguaguauu...	1	1	seq
........................aagcAagcaaagaaguaguauu...	1	1	seq
........................aagcgagcGaagaaguaguauu...	2	1	seq
........................aagcgagcaaagaaguaguauG...	2	1	seq
........................aaAcgagcaaagaaguaguauu...	3	1	seq
........................aagcgagcaaGgaaguaguauu...	1	1	seq
........................aagcgaAcaaagaaguaguauu...	1	1	seq
........................aagcgaUcaaagaaguaguauu...	1	1	seq
........................aagcgagcaaagaaUuaguauu...	1	1	seq
........................aagcgagcaaaAaaguaguauu...	1	1	seq
........................aagcgagcaaagaUguaguauu...	2	1	seq
........................aagcgagcaGagaaguaguauu...	2	1	seq
........................aagcgagcaaagaaguaguauu...	962	0	seq
........................aagcgGgcaaagaaguaguauu...	2	1	seq
........................aagAgagcaaagaaguaguauu...	2	1	seq
........................aGgcgagcaaagaaguaguauu...	2	1	seq
........................aagcgagcaaagaGguaguauu...	1	1	seq
........................aagcgagcaaagGaguaguauu...	1	1	seq
........................aagcgagcaaagaaguaguaGu...	1	1	seq
........................aagcgaCcaaagaaguaguauu...	1	1	seq
........................aagcgagcaaagaaguaguaAu...	2	1	seq
........................aagcgagcaaagaaguagCauu...	1	1	seq
........................aagcgagcaUagaaguaguauuu..	1	1	seq

```
                                                                 dme-miR-974-3p
                        dme-miR-974-5p
caauugucaccggucauguccuccaagcgagcaaagaaguaguauuuuguguuuccaagagcaaauauaacuucauuggaagcuaaguggauuugcccaaau

........................aagcgaUcaaagaaguaguauuu.......................................................  1     1  seq
........................aagUgagcaaagaaguaguauuu.......................................................  1     1  seq
........................aagcgGgcaaagaaguaguauuu.......................................................  3     1  seq
........................aagcgagcaaagaaguaguauuA.......................................................  3     1  seq
........................aagcgCgcaaagaaguaguauuu.......................................................  2     1  seq
........................aagcgagcaaagaaguagGauuu.......................................................  2     1  seq
........................aagcgagcaaagGaguaguauuu.......................................................  4     1  seq
........................aagcgagcaaagaaguaguGuuu.......................................................  4     1  seq
........................aagcgagcUaagaaguaguauuu.......................................................  2     1  seq
........................aagcgagcaaagaaguaAuauuu.......................................................  1     1  seq
........................aagcgagcaCagaaguaguauuu.......................................................  1     1  seq
........................aagcgagcaaagUaguaguauuu.......................................................  2     1  seq
........................aagcgagcaaagaaguaguCuuu.......................................................  1     1  seq
........................aaUcgagcaaagaaguaguauuu.......................................................  2     1  seq
........................aagcgagcaaagaaguagAauuu.......................................................  4     1  seq
........................aagcgagGaaagaaguaguauuu.......................................................  2     1  seq
........................aagcgagcaaagaaguaguaAuu.......................................................  2     1  seq
........................aagcgagcaaagaaUuaguauuu.......................................................  1     1  seq
........................aagcAagcaaagaaguaguauuu.......................................................  1     1  seq
........................aagcgagcaaagaagAaguauuu.......................................................  3     1  seq
........................aagGgagcaaagaaguaguauuu.......................................................  1     1  seq
........................aGgcgagcaaagaaguaguauuu.......................................................  4     1  seq
........................aaAcgagcaaagaaguaguauuu.......................................................  1     1  seq
........................aagcgagcaaCgaaguaguauuu.......................................................  1     1  seq
........................aagcgagcaaagaagGaguauuu.......................................................  1     1  seq
........................aagcgagcaGagaaguaguauuu.......................................................  2     1  seq
........................aagcgagcaaagaaguaUuauuu.......................................................  2     1  seq
........................aagcgagcaaagaaguaguauGu.......................................................  2     1  seq
........................aagcgagcaaagaaguaguauuG.......................................................  2     1  seq
........................Uagcgagcaaagaaguaguauuu.......................................................  1     1  seq
........................aagcgUgcaaagaaguaguauuu.......................................................  1     1  seq
........................aagcgaAcaaagaaguaguauuu.......................................................  4     1  seq
........................aagcgagcGaagaaguaguauuu.......................................................  2     1  seq
........................aagcgagcaaagaaguaguauuu.......................................................  2298  0  seq
........................aCgcgagcaaagaaguaguauuu.......................................................  2     1  seq
........................aagcgagcaaagaGguaguauuu.......................................................  1     1  seq
........................aagcgagcaaGgaaguaguauuu.......................................................  5     1  seq
........................aagcgagcaaaAaaguaguauuu.......................................................  3     1  seq
........................aagcgagAaaagaaguaguauuu.......................................................  1     1  seq
........................aagcgagcaaagaaguGguauuu.......................................................  2     1  seq
........................aagcgagcaaagaaguaguaGuu.......................................................  3     1  seq
........................aagcgagcaaaCaaguaguauuu.......................................................  1     1  seq
........................aagcCagcaaagaaguaguauuu.......................................................  2     1  seq
........................aagcgagcaaagaaguaguauAu.......................................................  2     1  seq
........................aagcgagcaaagaaguaguauuuC......................................................  1     1  seq
........................aagcgagcaaagaaguaguauuuA......................................................  1     1  seq
........................aagcgagcaaagaaguaguauuuU......................................................  107   1  seq
........................aagcgagcaaagaaguaguauuug......................................................  5     0  seq
........................aagcgagcaaagaaguaguauuuUu.....................................................  1     1  seq
.........................agcgagcaaagaaguaguauu........................................................  1     0  seq
.........................agcgagcaaagaaguaguauuu.......................................................  13    0  seq
.........................agcgagcaaagaaguaguauuuA......................................................  2     1  seq
................................................................auauaacuucauuggaagcuaa................  1     0  seq
.................................................................uauaacuucauuggaagcuaa................  4     0  seq
.................................................................uauaacuucauuggaagcuaag...............  3     0  seq
```

miRBase precursor : dme-mir-2499
Total read count : 91
dme-miR-2499-5p read count : 23
dme-miR-2499-3p read count : 41
remaining reads : 27

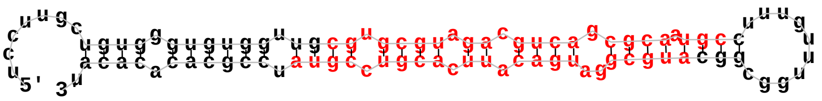

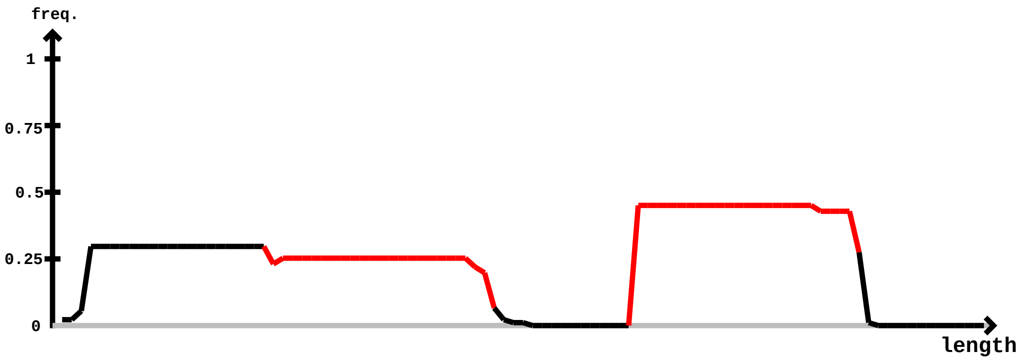

dme-miR-2499-3p
dme-miR-2499-5p

```
5'- uccuugcugugggugugguugcgugcguagacgucagcgcaaugccuuuguuuggcggcaugcggaugacauucacguccguauccgcacacacau   -3'   exp
    .......((((.((((((.((((.((((.((.((((.((((.((((..........))))))))..)))).)).)))).)))).)))))).)))).    reads   mm   sample
    uccuugcugugggugugguug...........................................................................    2       0    seq
    .ccuugcugugggugugguug...........................................................................    3       0    seq
    ..cuugcugugggugugguug...........................................................................    22      0    seq
    .....................cgugcguagacgucagcgcaa......................................................    3       0    seq
    .....................cgugcguagacgucagcgcaau.....................................................    2       0    seq
    .....................cgugcguagacgucagcgcaaug....................................................    12      0    seq
    .....................cgugcguagacgucagcgcaaugc...................................................    2       0    seq
    .....................cgugcguagacgucagcgcaaugcc..................................................    1       0    seq
    .....................cgugcguagacgucagcgcaaugccuu................................................    1       0    seq
    ......................gugcguGgacgucagcgcaaugc...................................................    1       1    seq
    ......................gugcguagacgucagcgcaaugc...................................................    1       0    seq
    ...........................................................augcggaugacauucacgu..................    2       0    seq
    ...........................................................augcggaugacauucacguccgu..............    14      0    seq
    ...........................................................augcggaugacauucacguccgua.............    22      0    seq
    ...........................................................augcggaugaAauucacguccgua.............    1       1    seq
    ...........................................................augcggaugacauucacguccgCa.............    1       1    seq
    ...........................................................augcggaugacauucacguccguaA............    1       1    seq
```

miRBase precursor : dme-mir-4966-1
Total read count : 562
dme-miR-4966-5p read count : 112
dme-miR-4966-3p read count : 450
remaining reads : 0

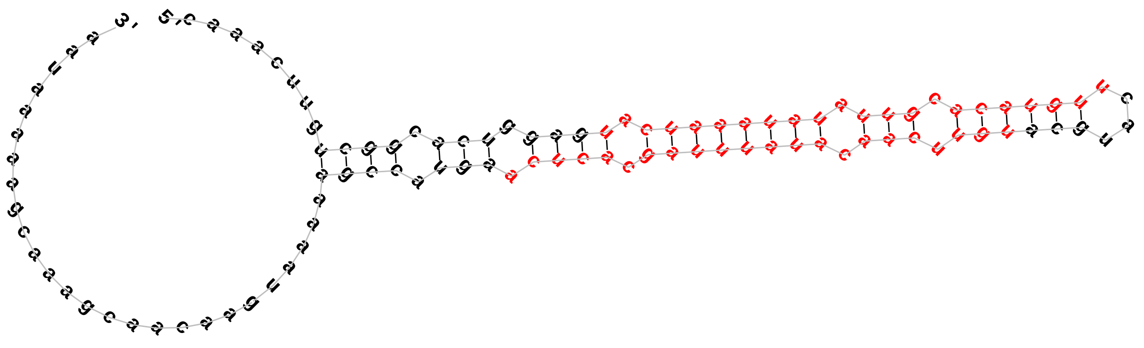

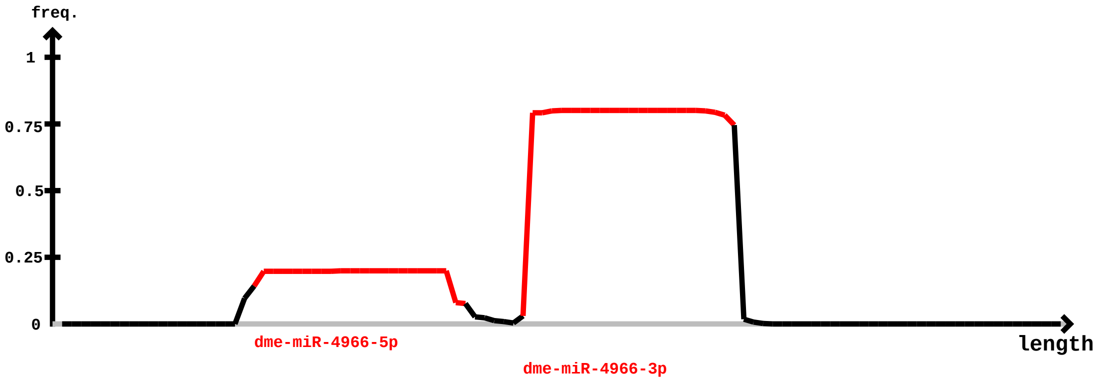


```
5'- caaacuugucggcacuggaguacuaaauauauugcacauguucaugcauguucaacauauuuagcacucaaguaccgaaaaaugaacaacgaaacgaaaaauaa  -3'   exp
    ........((((.(((.((((.((((((((.(((.((((((....)))))).))).)))))))).)))).))).))))..........................   reads  mm  sample
    ..................aguacuaGauauauugcacaug................................................................   1      1   seq
    ..................aguacuaaauauauugcacaug................................................................   47     0   seq
    ..................aguacuaaauauauugcacCug................................................................   1      1   seq
    ..................aguacuaaauauGuugcacaug................................................................   4      1   seq
    ..................aCuacuaaauauauugcacaug................................................................   1      1   seq
    ...................guacuaaauauauugcacaGg................................................................   1      1   seq
    ...................guacuaaauaGauugcacaug................................................................   1      1   seq
    ...................guacuaaauauauugcacaug................................................................   10     0   seq
    ...................guacuaaauauauugcacaugu...............................................................   1      0   seq
    ...................guacuaaauauauugcacauguuc.............................................................   1      0   seq
    ...................guUcuaaauauauugcacauguuca............................................................   1      1   seq
    ...................guacuaaauauauugCcauguuca............................................................   1      1   seq
    ...................guacuaaauauauugcacauguuca............................................................   4      0   seq
    ...................guacuaaauauauugcacauguucau...........................................................   2      0   seq
    ...................guacuaaauauauugcacauguucaug..........................................................   4      0   seq
    ....................uacuaaauauauugcacauU................................................................   1      1   seq
    ....................uacuaaauauauugcacaugu...............................................................   1      0   seq
    ....................uacuaaauauauugcacauguu..............................................................   27     0   seq
    ....................uacuaaauauaGugcacauguu..............................................................   1      1   seq
    ....................uacuaaauauauugcacauguuU.............................................................   1      1   seq
    ............................auauugcacauguucauU..........................................................   1      1   seq
    ..............................................cauguucaacauauuuagcacu....................................   2      0   seq
    ...............................................auguucaacauauuuagca......................................   1      0   seq
    ...............................................auguucaacauauuuagcacuc...................................   11     0   seq
    ...............................................auguucaacauauuuagcacuca..................................   1      0   seq
    ...............................................auguucaacauauuuagcacucU..................................   2      1   seq
    ................................................uguucaacauauuuagcac.....................................   3      0   seq
    ................................................uguucaacauauuuagcacu....................................   4      0   seq
    ................................................uguucaacauauuuagcacuc...................................   10     0   seq
    ................................................uguucaacauauuuaCcacuca..................................   1      1   seq
    ................................................uguucaacauauuAagcacuca..................................   1      1   seq
    ................................................uguucaacauauuuagcacuca..................................   374    0   seq
    ................................................uguucaacauauuuagcaUuca..................................   2      1   seq
    ................................................uAuucaacauauuuagcacuca..................................   2      1   seq
```

```
                    dme-miR-4966-5p
                                                dme-miR-4966-3p
caaacuugucggcacuggaguacuaaauauauugcacauguucaugcauguucaacauauuuagcacucaaguaccgaaaaaugaacaacgaaacgaaaaauaa

................................................uguucaacauauuuagGacuca..................................   1   1   seq
................................................uguucaacauCuuuagcacuca..................................   1   1   seq
................................................uguucaacauauAuagcacuca..................................   1   1   seq
................................................uguucaacauauuuagcacucG..................................   1   1   seq
................................................uguucaacauauuuagcGcuca..................................   1   1   seq
................................................uguucaacauauuuagcacucU..................................   7   1   seq
................................................uguucaacauauuuaUcacuca..................................   1   1   seq
................................................uguucaacauauuuGgcacuca..................................   2   1   seq
................................................uguucaacauauuuagcacucC..................................   3   1   seq
................................................uguucaacauauuuagcacAca..................................   2   1   seq
................................................uguucaacauauGuagcacuca..................................   1   1   seq
................................................uguucaacauauuuagcacCca..................................   2   1   seq
................................................ugGucaacauauuuagcacuca..................................   2   1   seq
................................................uguucaacauauuuagcacucaa.................................   3   0   seq
................................................uguucaacauauuuagcacucaU.................................   2   1   seq
................................................uguucaacauauuuagcacucaC.................................   1   1   seq
..................................................uucaacauauuuagcacuca..................................   1   0   seq
..................................................uucaacauauuuagcacucaag................................   3   0   seq
...................................................ucaacauauuuagcacucaagu...............................   1   0   seq
```

miRBase precursor : dme-mir-4966-2
Total read count : 562
dme-miR-4966-5p read count : 112
dme-miR-4966-3p read count : 450
remaining reads : 0

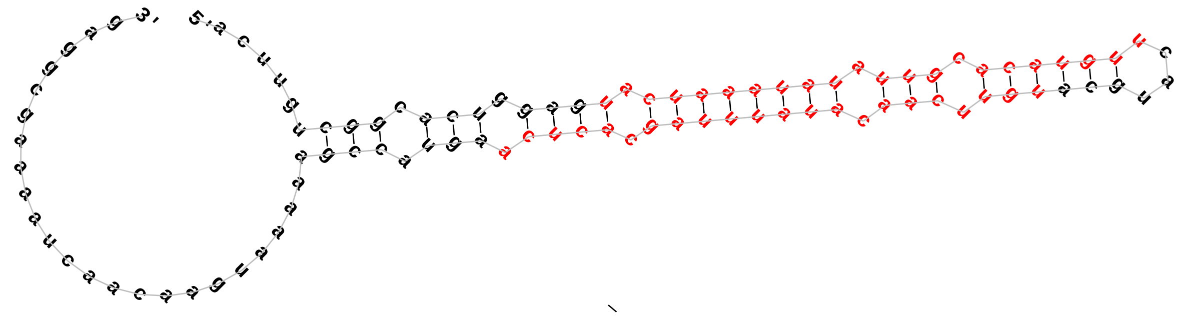

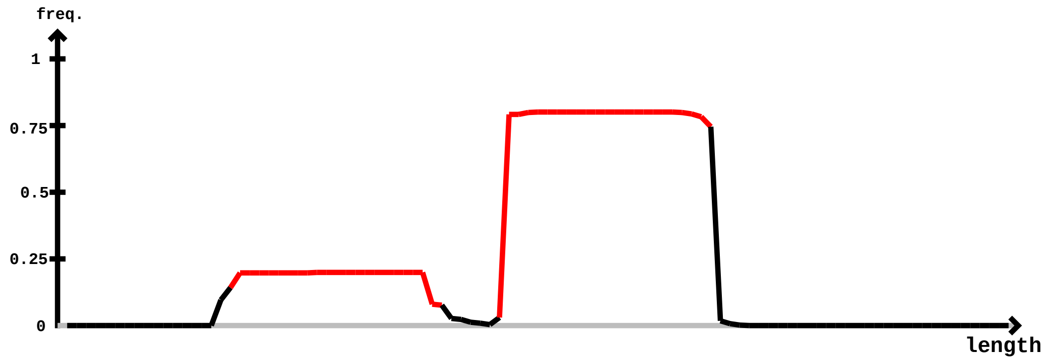


```
                                                           dme-miR-4966-3p
                          dme-miR-4966-5p
5'- acuugucggcacuggaguacuaaauauauugcacauguucaugcauguucaacauauuuagcacucaaguaccgaaaaaugaacaacuaaaagcggag  -3'   exp
    .....((((.(((.((((.((((((((.(((.((((((....)))))).))).)))))))).)))).))).)))).......................    reads     mm       sample
    ...............aguacuaaauauGuugcacaug.............................................................    4         1        seq
    ...............aguacuaGauauauugcacaug.............................................................    1         1        seq
    ...............aguacuaaauauauugcacaug.............................................................    47        0        seq
    ...............aguacuaaauauauugcacCug.............................................................    1         1        seq
    ...............aCuacuaaauauauugcacaug.............................................................    1         1        seq
    ................guacuaaauauauugcacaug.............................................................    10        0        seq
    ................guacuaaauaGauugcacaug.............................................................    1         1        seq
    ................guacuaaauauauugcacaGg.............................................................    1         1        seq
    ................guacuaaauauauugcacaugu............................................................    1         0        seq
    ................guacuaaauauauugcacauguuc..........................................................    1         0        seq
    ................guUcuaaauauauugcacauguuca.........................................................    1         1        seq
    ................guacuaaauauauugcacauguuca.........................................................    4         0        seq
    ................guacuaaauauauugcCcauguuca.........................................................    1         1        seq
    ................guacuaaauauauugcacauguucau........................................................    2         0        seq
    ................guacuaaauauauugcacauguucaug.......................................................    4         0        seq
    .................uacuaaauauauugcacauU.............................................................    1         1        seq
    .................uacuaaauauauugcacaugu............................................................    1         0        seq
    .................uacuaaauauauugcacauguu...........................................................    27        0        seq
    .................uacuaaauauaGugcacauguu...........................................................    1         1        seq
    .................uacuaaauauauugcacauguuU..........................................................    1         1        seq
    .........................auauugcacauguucauU.......................................................    1         1        seq
    ...........................................cauguucaacauauuuagcacu.................................    2         0        seq
    ............................................auguucaacauauuuagca...................................    1         0        seq
    ............................................auguucaacauauuuagcacuc................................    11        0        seq
    ............................................auguucaacauauuuagcacucU...............................    2         1        seq
    ............................................auguucaacauauuuagcacuca...............................    1         0        seq
    .............................................uguucaacauauuuagcac..................................    3         0        seq
    .............................................uguucaacauauuuagcacu.................................    4         0        seq
    .............................................uguucaacauauuuagcacuc................................    10        0        seq
    .............................................uguucaacauauAuagcacuca...............................    1         1        seq
    .............................................uAuucaacauauuuagcacuca...............................    2         1        seq
    .............................................uguucaacauauuuGgcacuca...............................    2         1        seq
    .............................................uguucaacauCuuuagcacuca...............................    1         1        seq
    .............................................uguucaacauauuuagcacucG...............................    1         1        seq
```

```
                                              dme-miR-4966-3p
                 dme-miR-4966-5p
acuugucggcacuggaguacuaaaauauauugcacauguucaugcauguucaacauauuuagcacucaaguaccgaaaaaugaacaacuaaaagcggag

..............................................uguucaacauauuuagGacuca...............................   1     1     seq
..............................................uguucaacauauuuagcaUuca...............................   2     1     seq
..............................................uguucaacauauuuagcacCca...............................   2     1     seq
..............................................uguucaacauauuuagcGcuca...............................   1     1     seq
..............................................uguucaacauauGuagcacuca...............................   1     1     seq
..............................................uguucaacauauuAagcacuca...............................   1     1     seq
..............................................uguucaacauauuuagcacuca...............................   374   0     seq
..............................................uguucaacauauuuaCcacuca...............................   1     1     seq
..............................................uguucaacauauuuagcacAca...............................   2     1     seq
..............................................uguucaacauauuuaUcacuca...............................   1     1     seq
..............................................uguucaacauauuuagcacucU...............................   7     1     seq
..............................................ugGucaacauauuuagcacuca...............................   2     1     seq
..............................................uguucaacauauuuagcacucC...............................   3     1     seq
..............................................uguucaacauauuuagcacucaU..............................   2     1     seq
..............................................uguucaacauauuuagcacucaC..............................   1     1     seq
..............................................uguucaacauauuuagcacucaa..............................   3     0     seq
................................................uucaacauauuuagcacuca...............................   1     0     seq
................................................uucaacauauuuagcacucaag.............................   3     0     seq
.................................................ucaacauauuuagcacucaagu............................   1     0     seq
```

miRBase precursor : dme-mir-975
Total read count : 1119
dme-miR-975-5p read count : 764
dme-miR-975-3p read count : 342
remaining reads : 13

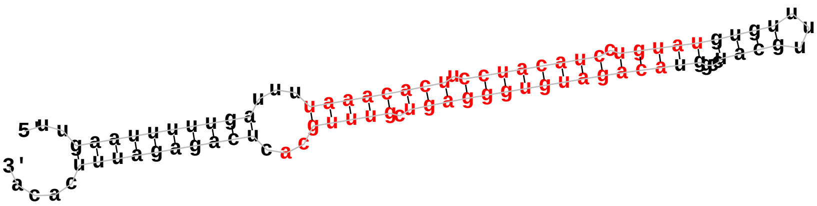

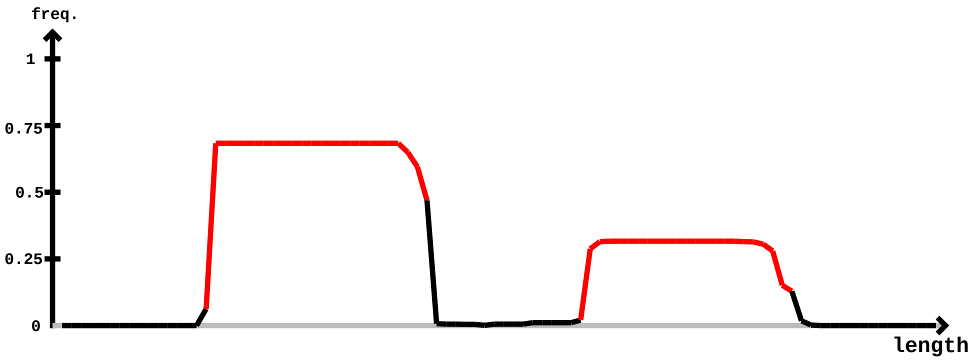

dme-miR-975-3p
dme-miR-975-5p

5'- uugaauuuuugauuuuaaacacuuccuacauccuguauguguuuugcauccgguacagaugugggagucguuugcacucagagauuucaca	-3'	exp		
..((((((((((...((((((((.((((((((.(((((((((...))))...)))))))))))))))))).)))))...))))))))))....		reads	mm	sample
..............uAaaacacuuccuacauccug..		1	1	seq
..............uuaaacacuuccuacauccug..		25	0	seq
..............uuaaacacuuccuacaucAugu..		1	1	seq
..............uuaaacacuuccuacauccugu...		18	0	seq
..............uuaaacacuuccuacauUcugua..		1	1	seq
..............uuaaacacuuccuacauccugua..		14	0	seq
..............uuaGacacuuccuacauccugua..		1	1	seq
..............uuaaacacuuccuacauccuguaA...		9	1	seq
...............uaaacacuuccuacauccug..		10	0	seq
...............uaaacacuuccAacauccug..		1	1	seq
...............uaaacacuGccuacauccug..		1	1	seq
...............uaaacacuuccuacauccugu...		42	0	seq
...............uaaacacuuccuacauccugGa..		1	1	seq
...............uaGacacuuccuacauccugua..		2	1	seq
...............uaaacaGuuccuacauccugua..		1	1	seq
...............uaaacacuuccuacaucGugua..		1	1	seq
...............uaaacacuuccuacaGccugua..		1	1	seq
...............uaaacacuuccuacauccuguU..		1	1	seq
...............uaaGcacuuccuacauccugua..		1	1	seq
...............uaaacacuuccuacauccugua..		117	0	seq
...............uaaacacuuccuacaucGuguau...		1	1	seq
...............uaaGcacuuccuacauccuguau...		1	1	seq
...............uaaacacuuGcuacauccuguau...		3	1	seq
...............uaaacacuuccuacauccuguaC...		2	1	seq
...............uaaacacuGccuacauccuguau...		1	1	seq
...............uaaacacuuccuacaucUuguau...		1	1	seq
...............uaaacacuuccuacauccuguau...		476	0	seq
...............uaaacaUuuccuacauccuguau...		2	1	seq
...............uaaacacuuccuacauAcuguau...		1	1	seq
...............uaaacacuuccuacauccuguGu...		1	1	seq
...............uaaacacuucGuacauccuguau...		2	1	seq
...............uaaacaGuuccuacauccuguau...		1	1	seq
...............uaaacacuuccAacauccuguau...		1	1	seq
...............uaaacacuuUcuacauccuguau...		2	1	seq

dme-miR-975-3p

dme-miR-975-5p

uugaauuuuugauuuuaaacacuuccuacauccuguauguguuuugcauccgguacagaugugggagucguuugcacucagagauuucaca

...............uaUacacuuccuacauccuguau...	1	1	seq
...............uaaacacuuccuacauccuguaA...	12	1	seq
...............uaaacacuuccuacauccuguauA..	1	1	seq
...............uaaacacuuccuacauccuguaug..	1	0	seq
...............uaaacacuuccuacauccuguaugug..	1	0	seq
...............uaaacacuuccuacauccuguauguguu..	3	0	seq
...............uaaacacuuUcuacauccuguauguguu..	1	1	seq
...............uaaacacuuccuacauccuguauguguuu...	1	0	seq
....ugcauccgguacagaugugggagucgu....................	1	0	seq
....ugcauccgguacagaugugggagucguu...................	4	0	seq
....ugcauccgguacagaugugggagucguuu..................	1	0	seq
....uccgguacagaugugggagucg.....................	2	0	seq
....uccgguacagaugugggagucguu...................	2	0	seq
....uccgguacagaugugggagucguuug.................	1	0	seq
....uccgguacagaugugggagucguuugca...............	1	0	seq
....uacagaugugggagucguu...................	1	0	seq
....uacagaugugggagucguuug.................	1	0	seq
....uacagaugugggagucguuugc................	3	0	seq
....uacagaugugggagucguuugca...............	6	0	seq
....acagaugugggagucguu...................	2	0	seq
....acagaugugggagucguuu..................	28	0	seq
....acagaugGgggagucguuug.................	1	1	seq
....acagaugugggagAcguuug.................	1	1	seq
....acagaugugggGgucguuug.................	2	1	seq
....acagaugugggaCucguuug.................	2	1	seq
....acagaugugggaAucguuug.................	2	1	seq
....aAagaugugggagucguuug.................	1	1	seq
....aGagaugugggagucguuug.................	1	1	seq
....acagaugugggagucguuuU.................	2	1	seq
....acagaugugggagucgAuug.................	1	1	seq
....acagaugugggagucguuug.................	122	0	seq
....acagaCgugggagucguuug.................	1	1	seq
....acaAaugugggagucguuug.................	1	1	seq
....acagaGgugggagucguuug.................	1	1	seq
....acagaugugggagucguuGgc................	1	1	seq
....acagaugugggagucguuugA................	1	1	seq
....acagaugugggagCcguuugc................	1	1	seq
....acagaugugggagucguuugc................	17	0	seq
....acagaugugggagucgGuugc................	1	1	seq
....acagaugugggGgucguuugca...............	1	1	seq
....acagaugugggagucguuugcG...............	1	1	seq
....acagaugugggagucguuugca...............	103	0	seq
....acagaugugggagucguuugGa...............	1	1	seq
....acagaugugggagucguuugcac..............	1	0	seq
....acagaugugggagucguuugcaA..............	2	1	seq
....acagaugugggagucguuugcaUu.............	1	1	seq
....cagaugugggagucguuug.................	3	0	seq
....cagaugugggaguGguuugca...............	1	1	seq
....cagaugugggagucguuugca...............	9	0	seq
....cagaugugggagucguuugcG...............	1	1	seq
....cagaugugggagucguuugcac..............	10	0	seq
....cagaugugggagucguuugcaA..............	1	1	seq
....cagaugGgggagucguuugcac..............	1	1	seq
....cagaugugggagucguuugcaU..............	3	1	seq
....cagaugugggagucguuugcacA.............	1	1	seq
....agaugugggagucguuug.................	1	0	seq
....aCaugugggagucguuugc................	1	1	seq

miRBase precursor : dme-mir-976
Total read count : 78
dme-miR-976-5p read count : 3
dme-miR-976-3p read count : 75
remaining reads : 0

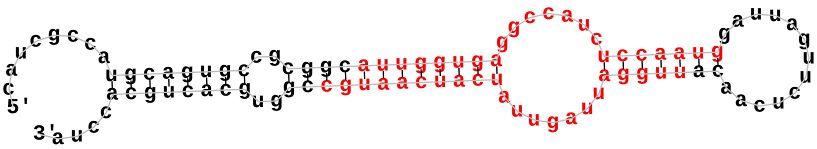

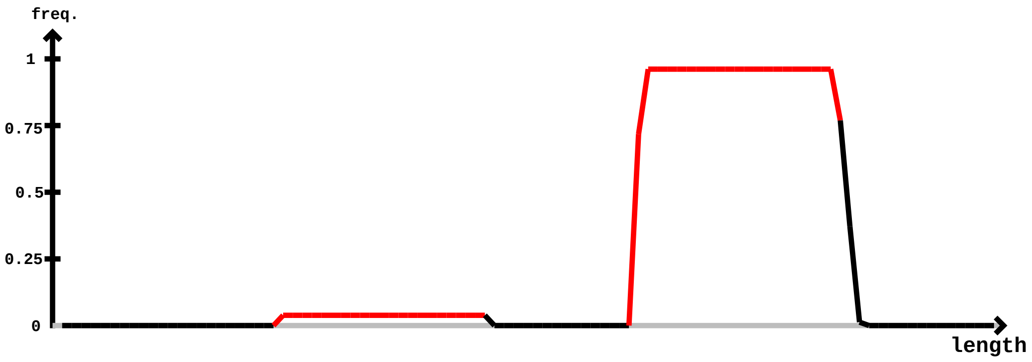


```
                                                                  dme-miR-976-3p
                        dme-miR-976-5p
5'- caucgccaugcagugccgcggcauuggugaggccaucuccaauggauuaguucucaacauuggauuaguuaucaucaaugccggugcacugcaccua   -3'    exp
    ........((((((((..((((((((((((.......(((((((.............))))))).......))))))))))))..))))))))....    reads     mm       sample
    ......................auuggugaggccaucuccaaug.....................................................    3         0        seq
    ...........................................................uuggauuaguuaucaucaaug.................    13        0        seq
    ...........................................................uuggauuaAuuaucaucaaug.................    1         1        seq
    ...........................................................uuggauuaguuCucaucaaug.................    1         1        seq
    ...........................................................uuggauuaguuaucaucaauCc................    1         1        seq
    ...........................................................uuggauuaguuaucaucaaugc................    26        0        seq
    ...........................................................uuggauuaUuuaucaucaaugc................    2         1        seq
    ...........................................................uuggauuaguuaucaucaaugU................    1         1        seq
    ...........................................................uuggauuaguuaucaucaaugcc...............    10        0        seq
    ...........................................................uuggauuaguuaucaucaaugccA..............    1         1        seq
    ............................................................uggauuaguuaucaucaaugc................    1         0        seq
    ............................................................uggauuaguuaucaucaaugUc...............    1         1        seq
    ............................................................uggauuaguuaucaucaaugcc...............    17        0        seq
```

miRBase precursor : dme-mir-977
Total read count : 792
dme-miR-977-5p read count : 96
dme-miR-977-3p read count : 672
remaining reads : 24

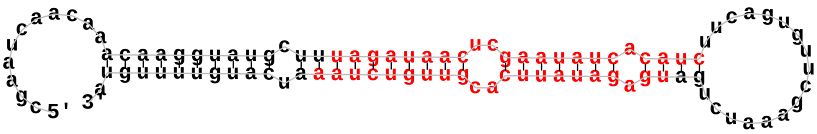

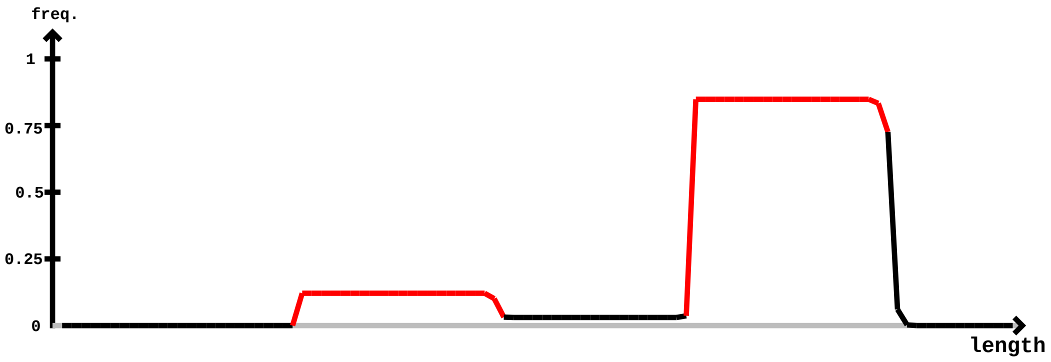


```
                                  dme-miR-977-5p
                                                                  dme-miR-977-3p
5'- cgaaucaacaaacaagguaugcuuuagauaacucgaauaucacaucuucaguguucgaaaucugaugagauauucacguugucuaaaucauguuuugua -3'   exp
    ..........((((((((((.((((((((((..(((((((.((((..................)))).)))))))..)))))))))).)))))))))).   reads   mm   sample
    ........................uagauaacucgaauaucaca.......................................................   16      0    seq
    ........................uagauaacucgaauaucacau......................................................   54      0    seq
    ........................uUgauaacucgaauaucacau......................................................   1       1    seq
    ........................uagauaacucgaauaucacauc.....................................................   24      0    seq
    ........................uagauaacucgaaAucacauc......................................................   1       1    seq
    ..............................................uucaguguucgaaaucugU..................................   1       1    seq
    ..............................................uucaguguucgaaaucuga..................................   22      0    seq
    ..............................................uucaguguucgaaaucuAa..................................   1       1    seq
    ................................................................augagauauucacguugucua..............   1       0    seq
    ................................................................augagauauucacguugucuaa.............   3       0    seq
    ................................................................augagauauucacguugucuaaa............   1       0    seq
    .................................................................ugagauauucacguugucu...............   12      0    seq
    .................................................................ugagauaAucacguugucua..............   1       1    seq
    .................................................................ugagauauucacguuguAua..............   1       1    seq
    .................................................................ugagauauucacAuugucua..............   1       1    seq
    .................................................................ugagauauucacguugucua..............   80      0    seq
    .................................................................ugagauauuUacguugucua..............   1       1    seq
    .................................................................ugagauauucacguugucuaa.............   510     0    seq
    .................................................................ugGgauauucacguugucuaa.............   4       1    seq
    .................................................................uAagauauucacguugucuaa.............   1       1    seq
    .................................................................ugagauauucacguugGcuaa.............   1       1    seq
    .................................................................ugagauGuucacguugucuaa.............   3       1    seq
    .................................................................ugagauauucacguugucuaU.............   4       1    seq
    .................................................................ugaUauauucacguugucuaa.............   1       1    seq
    .................................................................ugagauauucacguugucuaaa............   44      0    seq
    .................................................................ugagauauucacguugucuaaU............   1       1    seq
    .................................................................ugagauauucacguugucuaaaA...........   1       1    seq
    .................................................................ugagauauucacguugucuaaau...........   1       0    seq
```

miRBase precursor : dme-mir-978
Total read count : 194
dme-miR-978-5p read count : 7
dme-miR-978-3p read count : 187
remaining reads : 0

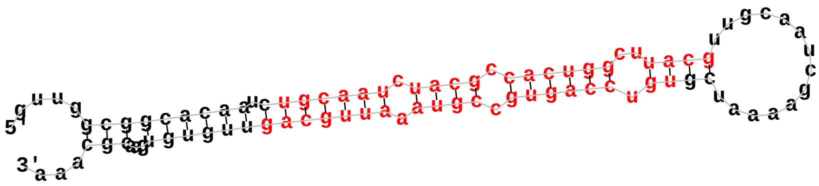

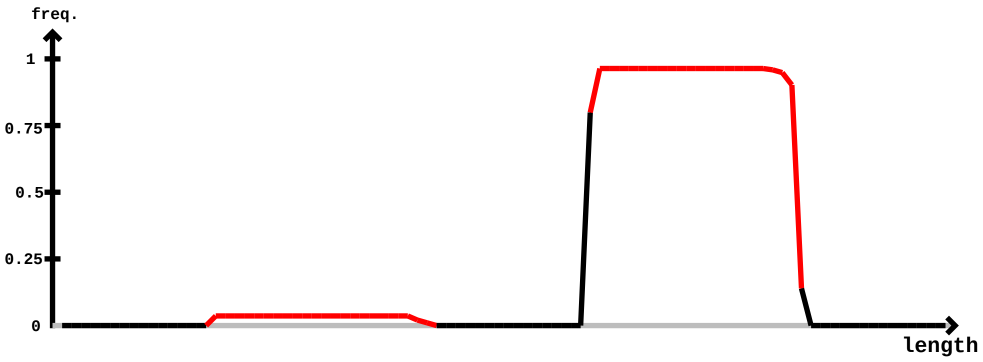

dme-miR-978-5p

dme-miR-978-3p

```
5'- guuggcggcacaaucugcaaucuacgccacuggcuuacguugcaaucgaaaaucguguccagugccguaaauugcaguugugugaacgcaaa   -3'    exp
    ....((((((((.(((((((.((((.((((((..((((................)))).)))))).)))).))))))))))))...)))...   reads       mm          sample
    ...............ugcaaucuacgccacuggcuu........................................................   3           0           seq
    ...............ugcaaucuacgccacuggcuua.......................................................   2           0           seq
    ...............ugcaaucuacgccacuggcuuac......................................................   1           0           seq
    ...............ugcaaucuacgccacuggcuuaA......................................................   1           1           seq
    ......................................................guguccagugccguaaaAu...................   1           1           seq
    ......................................................guguccagugccguaaauug..................   1           0           seq
    ......................................................guguccagugccguaaauugc.................   8           0           seq
    ......................................................guguccagugccguaaauugAa................   1           1           seq
    ......................................................guguccagugccgGaaauugca................   1           1           seq
    ......................................................guguccagugccguGaauugca................   1           1           seq
    ......................................................guguccagugccguaaauugca................   131         0           seq
    ......................................................guguccagugccCuaaauugca................   1           1           seq
    ......................................................guguccagugccguaaauugcag...............   8           0           seq
    ......................................................guguccagugccguaaauugcaA...............   2           1           seq
    .......................................................uguccagugccguaaauug..................   1           0           seq
    .......................................................uguccagugccguaaauugc.................   1           0           seq
    .......................................................uguccagugccguaaauugca................   13          0           seq
    .......................................................uguccagugccguaaauugcag...............   17          0           seq
```

miRBase precursor : dme-mir-979
Total read count : 417
dme-miR-979-5p read count: 108
dme-miR-979-3p read count: 303
remaining reads : 6

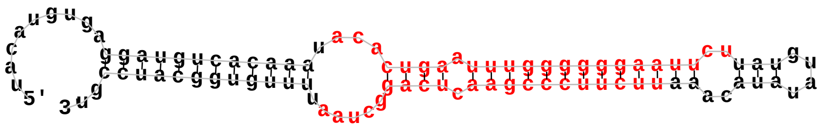

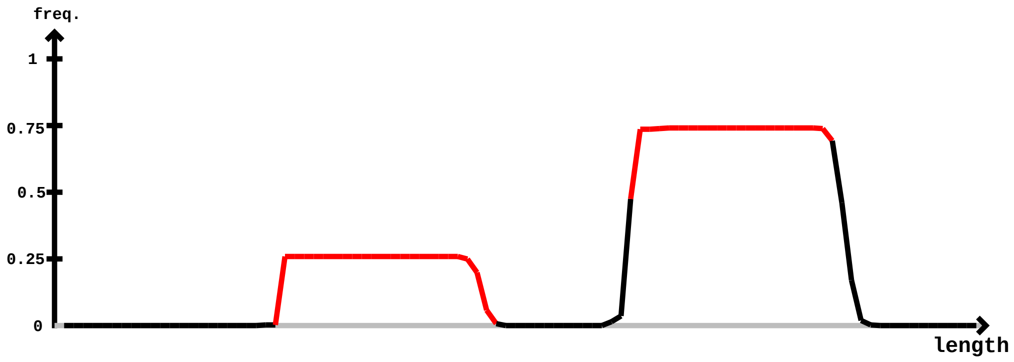

dme-miR-979-5p

dme-miR-979-3p

```
5'- uacaugugaggaugucacaaauacacugaauuuggggggaauucuuauguauauacaaauucuucccgaacucaggcuaauuuguggcauccgu  -3'    exp
    .........((((((((((((....((((.(((((((((((((..(((....)))..))))))))))))).)))).......))))))))))))..   reads    mm    sample
    ....................auacacugaauuuggggggaauu...................................................   1        0     seq
    ......................acacugaauuuggggggaa.....................................................   4        0     seq
    ......................acacugaauuuggggggaau....................................................   20       0     seq
    ......................acacugaauuuggggggaaA....................................................   1        1     seq
    ......................acacugaauuuggggggaauu...................................................   57       0     seq
    ......................acGcugaauuuggggggaauu...................................................   1        1     seq
    ......................acacugaauuuggggggaauuU..................................................   13       1     seq
    ......................acacugaauuugggUggaauuc..................................................   1        1     seq
    ......................acacugaauuuggggggaauuA..................................................   1        1     seq
    ......................acacugaauuuggggggaauuc..................................................   6        0     seq
    ......................acacugaauuuggggggaauucu.................................................   3        0     seq
    ........................................................aaauucuucccgaacucaggcua...............   3        0     seq
    ........................................................aaauucuucccgaacucaggcuaa..............   3        0     seq
    .........................................................aauucuucccgaacucaggcu................   1        0     seq
    .........................................................aauucuucccgaacucaggcua...............   2        0     seq
    .........................................................aauucuucccgaacucaggcuaa..............   1        0     seq
    .........................................................aauucuucccgaacucaggcuaau.............   3        0     seq
    .........................................................aauucuucccgaacucaggcuaauu............   2        0     seq
    ..........................................................auucuucccgaacucaggcua...............   11       0     seq
    ..........................................................auucuuUccgaacucaggcua...............   2        1     seq
    ..........................................................auucuucccgaGcucaggcuaa..............   1        1     seq
    ..........................................................auucuucccgaacucaggcuaG..............   1        1     seq
    ..........................................................auucuucccgaacucaggcuaa..............   64       0     seq
    ..........................................................auucuucccgaGcucaggcuaau.............   1        1     seq
    ..........................................................auucuucccgGacucaggcuaau.............   1        1     seq
    ..........................................................auucuucccgaacucaggcuaau.............   83       0     seq
    ..........................................................auucuucccgaacuGaggcuaau.............   1        1     seq
    ..........................................................auuUuucccgaacucaggcuaau.............   1        1     seq
    ..........................................................auucuucccgaacuGaggcuaauu............   1        1     seq
    ..........................................................auucuucccgaacucaggcuaauu............   16       0     seq
    ...........................................................uucuucccgaacucaggcua...............   1        0     seq
    ...........................................................uucuucccgaacucagCcuaa..............   1        1     seq
    ...........................................................uucuucccgaacucaggcuaa..............   26       0     seq
    ...........................................................uucuucccgaacucaggcuaau.............   29       0     seq
```

```
                      dme-miR-979-5p
                                                           dme-miR-979-3p
uacaugugaggaugucacaaauacacugaauuuggggggaauucuuauguauauacaaauucuucccgaacucaggcuaauuuuguggcauccgu

...........................................................uuUuucccgaacucaggcuaau..............    1    1    seq
...........................................................uucuucccgaacucaggcuaauu.............    42   0    seq
...........................................................uucuucAcgaacucaggcuaauu.............    1    1    seq
...........................................................uucuucccgaacucaggcuaGuu.............    1    1    seq
...........................................................uucuucccgaacucaggcuaauuu............    6    0    seq
...........................................................uucuucccgaacucaggcuaauuC............    1    1    seq
.............................................................Uuucccgaacucaggcuaau..............    1    1    seq
..............................................................uucccgaacucaggcuaauuuC...........    1    1    seq
```

miRBase precursor : dsi-mir-972
Total read count : 97
dsi-miR-972-5p read count : 74
dsi-miR-972-3p read count : 23
remaining reads : 0

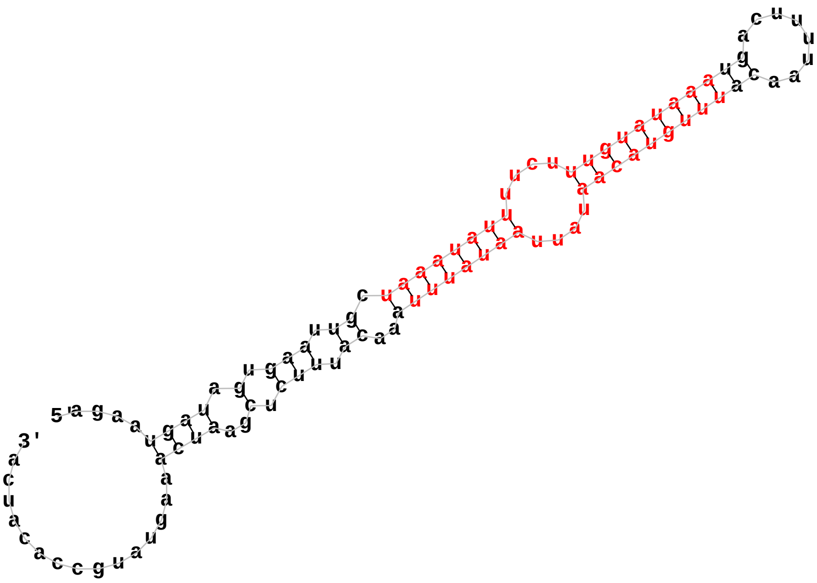

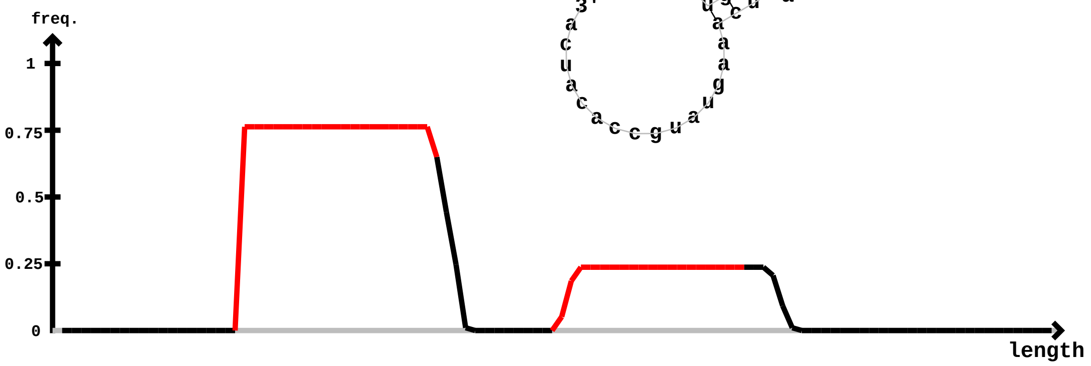

dsi-miR-972-3p
dsi-miR-972-5p

```
5'- agaaugauagugaauugcuaaauauuuucuuuguauaaaugacuuuuaacauuuguacaauauuaauauuuaaacauuucucgaaucaaaguaugccacauca   -3'   exp
    ....((((.(.(((.((.((((((((....((((((((((........))))))))))....))))))))..)).))).)..))))................   reads   mm   sample
    ..................uaaauauuuucuuuguauaa.................................................................   11      0    seq
    ..................uaaauauuuucuuuguauaaa................................................................   18      0    seq
    ..................uaaauauuuuUuuuguauaaa................................................................   2       1    seq
    ..................uaaauauuuucuuuguauaaau...............................................................   19      0    seq
    ..................uaaauauuuucuuuguauaaaug..............................................................   23      0    seq
    ..................uaaauauuuucuuuguauaaauga.............................................................   1       0    seq
    ...................................................uuuguacaauauuaauauuuaa..............................   1       0    seq
    ...................................................uuuguacaauauuaauauuuaaa.............................   2       0    seq
    ...................................................uuuguacaauauuaauauuuaaac............................   2       0    seq
    ....................................................uuguacaauauuaauauuuaa..............................   1       0    seq
    ....................................................uuguacaauauuaauauuuaaa.............................   8       0    seq
    ....................................................uuguacaauauuaauauuuaaac............................   2       0    seq
    ....................................................uuguacaauauuaauauuuaaaA............................   1       1    seq
    ....................................................uuguacaauauuaauauuuaaaca...........................   1       0    seq
    .....................................................uguacaauauuaauauuuaa..............................   1       0    seq
    .....................................................uguacaauauuaauauuuaaa.............................   1       0    seq
    .....................................................uguacaauauuaauauuuaaac............................   3       0    seq
```

miRBase precursor : dsi-mir-9369
Total read count : 3065
dsi-miR-9369-5p read count: 290
dsi-miR-9369-3p read count: 2775
remaining reads : 0

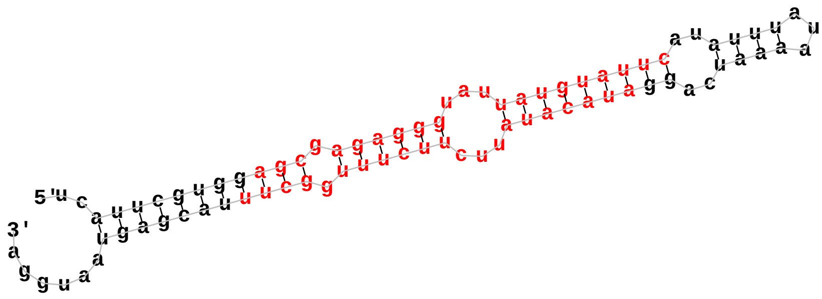

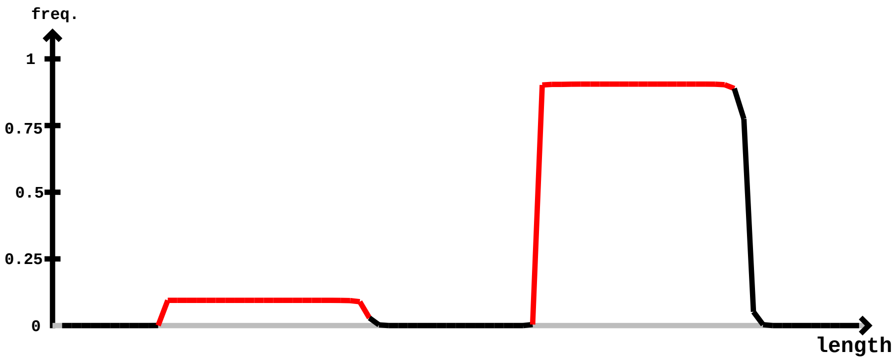


```
                                   dsi-miR-9369-5p
                                                                      dsi-miR-9369-3p
5'- ucauucguggagcgagaggguauuauguauucauauuuauaaaaucaggauacauauucuucuuuggcuuuacgaguaaugga   -3'   exp
    ..((((((((((.((((((...((((((((((..((((...))))..))))))))))...)))))).))))))))))......    reads   mm   sample
    .........Cagcgagaggguauuauguauu....................................................    1       1    seq
    ..........agcgagaggguauuaugu.......................................................    1       0    seq
    ..........agcgagagggAauuaugua......................................................    1       1    seq
    ..........agcgagaggguauuaugua......................................................    1       0    seq
    ..........agcgagagggGauuauguau.....................................................    1       1    seq
    ..........agcgagaggguauuauguau.....................................................    11      0    seq
    ..........agcgagagggAauuauguau.....................................................    1       1    seq
    ..........agcgagaggUuauuauguauu....................................................    1       1    seq
    ..........agcgagagggGauuauguauu....................................................    3       1    seq
    ..........agcgagaggguauuauguauu....................................................    181     0    seq
    ..........agcgagagggGauuauguauuc...................................................    1       1    seq
    ..........agcgagaUgguauuauguauuc...................................................    1       1    seq
    ..........agcgagaggguauuauguauuU...................................................    5       1    seq
    ..........agcgagaggguauuauguauuG...................................................    1       1    seq
    ..........agcgagagggAauuauguauuc...................................................    2       1    seq
    ..........agcgagaggguauuauguauuA...................................................    3       1    seq
    ..........agcgagaggguauuauguauuc...................................................    67      0    seq
    ..........agcgagaggguauuauguauuca..................................................    8       0    seq
    ................................................Aauacauauucuucuuuggcuu.............    2       1    seq
    ................................................Cauacauauucuucuuuggcuu.............    1       1    seq
    ................................................Cauacauauucuucuuuggcuuu............    1       1    seq
    ................................................gauacauauucuucuuuggcuuu............    1       0    seq
    ................................................Aauacauauucuucuuuggcuuu............    6       1    seq
    ................................................Aauacauauucuucuuuggcuuua...........    1       1    seq
    .................................................auacauauucuucuuugA................    1       1    seq
    .................................................auacauauucuucuuuggc...............    4       0    seq
    .................................................auacauauucuucuuuggA...............    2       1    seq
    .................................................auacauauucuucuuuggcA..............    3       1    seq
    .................................................auacauauucuucuuuggcu..............    39      0    seq
    .................................................auacauauucuuUuuuggcu..............    1       1    seq
    .................................................auacauauucuucuuuggAuu.............    1       1    seq
    .................................................auacauGuucuucuuuggcuu.............    1       1    seq
    .................................................auacauauucuucuuuggcuA.............    1       1    seq
    .................................................auaAauauucuucuuuggcuu.............    1       1    seq
```

```
          dsi-miR-9369-5p
                                                 dsi-miR-9369-3p
ucauucguggagcgagaggguauuauguauucauauuuauaaaaucaggauacauauucuucuuuggcuuuacgaguaaugga

.................................................auacauauuUuucuuuggcuu.............    3     1    seq
.................................................auacauauucuuUuuuggcuu.............    1     1    seq
.................................................auaUauauucuucuuuggcuu.............    1     1    seq
.................................................auacauauucuucuuuggcuu.............    338   0    seq
.................................................auacauauucuucuuuggcuAu............    1     1    seq
.................................................auacauGuucuucuuuggcuuu............    1     1    seq
.................................................auaUauauucuucuuuggcuuu............    1     1    seq
.................................................auacauauucuucuuuggcuCu............    1     1    seq
.................................................auacauauucuuAuuuggcuuu............    2     1    seq
.................................................auacauauucuucuuuggcuuG............    2     1    seq
.................................................auGcauauucuucuuuggcuuu............    1     1    seq
.................................................auacauauucuuUuuuggcuuu............    2     1    seq
.................................................auacauauuAuucuuuggcuuu............    1     1    seq
.................................................auacauauucuucuuuggUuuu............    3     1    seq
.................................................auacauauucuucuuuggcuuA............    9     1    seq
.................................................auacauauucuucuuuggcuuC............    2     1    seq
.................................................auaAauauucuucuuuggcuuu............    2     1    seq
.................................................auacauauucuucuuuggcuuu............    2157  0    seq
.................................................Guacauauucuucuuuggcuuu............    1     1    seq
.................................................auacauCuucuucuuuggcuuu............    1     1    seq
.................................................auacauauuUuucuuuggcuuu............    4     1    seq
.................................................auacauauucuucuuuggGuuu............    5     1    seq
.................................................auacauauucuuGuuuggcuuu............    1     1    seq
.................................................aCacauauucuucuuuggcuuu............    1     1    seq
.................................................auacauauucuucuuuggAuuu............    2     1    seq
.................................................auacauauucuucCuuggcuuu............    2     1    seq
.................................................auacaCauucuucuuuggcuuu............    1     1    seq
.................................................auacauauAcuucuuuggcuuu............    1     1    seq
.................................................auacauauucuucuuuggcuuuG...........    1     1    seq
.................................................auacauauucuucuuuggcuuua...........    40    0    seq
.................................................auacauauucuucuuuggcuuuU...........    101   1    seq
.................................................auacauauucuucuuuggcuuuaA..........    10    1    seq
..................................................uacauauucuucuuuggcuuu............    4     0    seq
..................................................uacauauucuucuuuggcuuua...........    3     0    seq
....................................................Aauauucuucuuuggcuuua...........    2     1    seq
.....................................................auauucuucuuuggcuuu............    1     0    seq
```

miRBase precursor : dsi-mir-973
Total read count : 389
dsi-miR-973-5p read count : 4
dsi-miR-973-3p read count : 385
remaining reads : 0

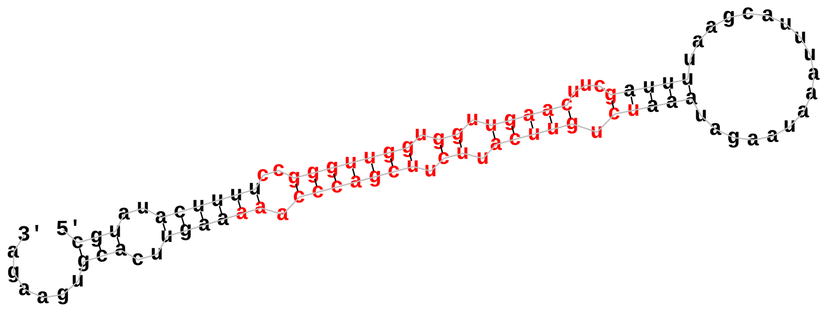

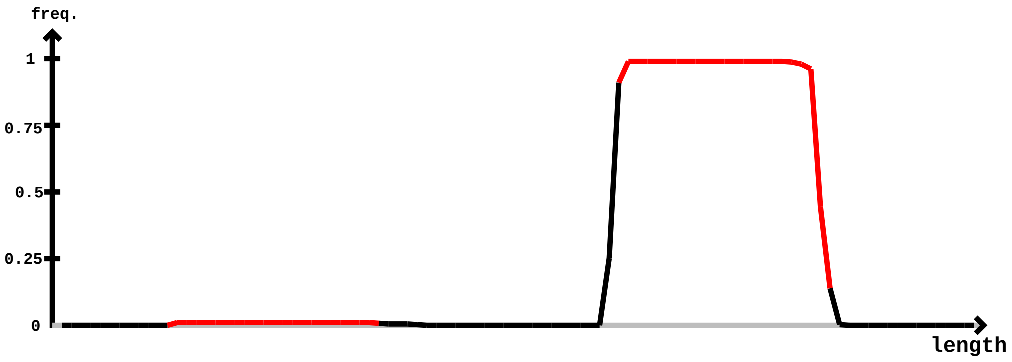


```
                                                          dsi-miR-973-3p
              dsi-miR-973-5p
5'- cguauacuuuccggguuggugguugaacuucgauuuuaagcauuuaaauaagauaaaucuguucauucuucgacccaaaaaguucacgugaaga  -3'   exp
    (((..((((((..(((((((.((.(((((...(((((.................))))).))))).)).))))))).))))))..)))......   reads    mm    sample
    ..........ccggguuggugguugaacuuc...............................................................   1        0     seq
    ..........ccggguuggugguugaacuucg..............................................................   1        0     seq
    ..........ccggguuggugguugaacuucgauu...........................................................   1        0     seq
    ..........ccggguuggugguugaacuucgauuu..........................................................   1        0     seq
    .......................................................aaucuguucauucuucgac....................   1        0     seq
    .......................................................aaucuguucauucuucgacc...................   3        0     seq
    .......................................................aaucuguucauucuucgaccc..................   4        0     seq
    .......................................................aaucuguuAauucuucgaccca.................   1        1     seq
    .......................................................aaucuguucauucuucgaccca.................   80       0     seq
    .......................................................aaucuguucauucuucgacccU.................   1        1     seq
    .......................................................aaucuguucauucuucgaUcca.................   1        1     seq
    .......................................................aaucuguucauucuucgacccaU................   1        1     seq
    .......................................................aaucuguucauucuucgacccaa................   5        0     seq
    .......................................................aaucuguucauucuucgacccaaa...............   1        0     seq
    ........................................................aucuguucauucuucgaccc..................   3        0     seq
    ........................................................aucuguucauucuucgaccca.................   117      0     seq
    ........................................................aucuguuAauucuucgaccca.................   1        1     seq
    ........................................................aucuguucauucuucgacccaa................   87       0     seq
    ........................................................aucuguucauucuucgacccaU................   7        1     seq
    ........................................................aucuguucauucuucgacccCa................   1        1     seq
    ........................................................aucuguucauucuucgacccaC................   5        1     seq
    ........................................................aucuguucauucuCcgacccaa................   1        1     seq
    ........................................................aucuguucauucuucgacccaaa...............   34       0     seq
    .........................................................ucuguucauucuucgacccaa................   12       0     seq
    .........................................................ucuguucauucuucgacccaaa...............   18       0     seq
    .........................................................ucuguucauucuucgacccaaaa..............   1        0     seq
```

miRBase precursor : dsi-mir-974
Total read count : 1926
dsi-miR-974-5p read count : 1862
dsi-miR-974-3p read count : 64
remaining reads : 0

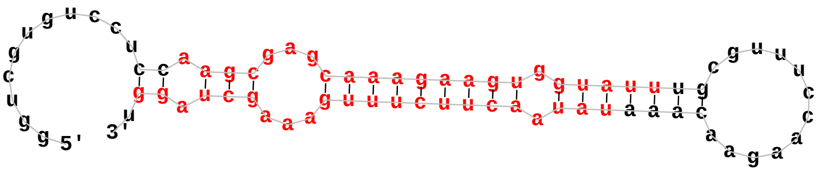

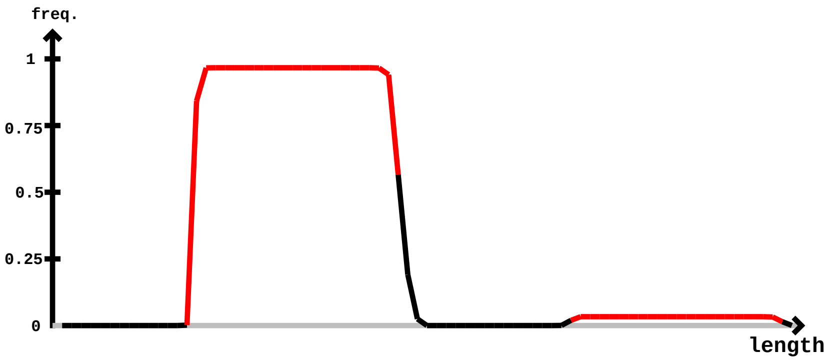


```
                                                                   dsi-miR-974-3p
                 dsi-miR-974-5p
5'- ggucguguccuccaagcgagcaaagaagugguauuugcguuuccaagaacaaauauaacuucuuugaaagcuaggu   -3'    exp
    ((......)).((.(((...((((((((.(((((((............))))))).)))))))))...))).)).    reads      mm         sample
    ............Aaagcgagcaaagaagugguau..........................................    1          1          seq
    ............Aaagcgagcaaagaagugguauu.........................................    1          1          seq
    ............Aaagcgagcaaagaagugguauuu........................................    1          1          seq
    .............aagcgagcaaagaaguggu............................................    1          0          seq
    .............aagcgagcaUagaaguggua...........................................    1          1          seq
    .............aagcgagcaaagaaguggua...........................................    40         0          seq
    .............aagcgagcaaagaagugguU...........................................    2          1          seq
    .............aagcgagcaaagaaguggGau..........................................    1          1          seq
    .............aagcgagcaaagaagugguau..........................................    689        0          seq
    .............aagcgagcaaaUaagugguau..........................................    1          1          seq
    .............aGgcgagcaaagaagugguau..........................................    1          1          seq
    .............aagcgagcaaagaagugUuau..........................................    1          1          seq
    .............aagcgagcaaagaagCgguau..........................................    1          1          seq
    .............aagcgagcaaagaaguggAau..........................................    2          1          seq
    .............aagcgagcGaagaagugguau..........................................    1          1          seq
    .............aagcgagcaGagaagugguau..........................................    2          1          seq
    .............aagcgagcaaagaagugguaA..........................................    4          1          seq
    .............aagcgagcaaagaagGgguau..........................................    1          1          seq
    .............aagcgagcaaagaagugguUu..........................................    1          1          seq
    .............aGgcgagcaaagaagugguauu.........................................    1          1          seq
    .............aagcgagcaaagaagugguUuu.........................................    1          1          seq
    .............aagcgagcaaagaagugguauu.........................................    641        0          seq
    .............aagcgagcaaagaagAgguauu.........................................    1          1          seq
    .............aagcgagcaaagaaguggAauu.........................................    1          1          seq
    .............aagcgagcaaagaagugguauA.........................................    3          1          seq
    .............aagcgagcaaagaaCugguauu.........................................    1          1          seq
    .............aagcgGgcaaagaagugguauu.........................................    1          1          seq
    .............aagcgagcaaagaagGgguauu.........................................    1          1          seq
    .............aagcgagcaaagaagugguGuu.........................................    1          1          seq
    .............aagcgagcaaagaagugguauuA........................................    16         1          seq
    .............aagcgagcaaagaagugguauAu........................................    1          1          seq
    .............aagcgagcaaagaagugguauuu........................................    168        0          seq
    .............aagGgagcaaagaagugguauuu........................................    1          1          seq
    .............aagcgagcaaagaaguggGauuu........................................    1          1          seq
```

```
                                                     dsi-miR-974-3p
             dsi-miR-974-5p
ggucguguccuccaagcgagcaaagaagugguauuugcguuuccaagaacaaauauaacuucuuugaaagcuaggu

.............aagcgagcaaagaagugguaAuu........................................   1     1     seq
.............aagcgagcaaagaagugguauuuA.......................................   4     1     seq
.............aagcgagcaaagaagugguauuuU.......................................   26    1     seq
..............agcgagcaaagaaguggu............................................   1     0     seq
..............agcgagcaaagaagugguU...........................................   1     1     seq
..............agcgagcaaagaaguggua...........................................   4     0     seq
..............agcgagcaaagaagugguaA..........................................   1     1     seq
..............agcgagcaaagaagugguau..........................................   13    0     seq
..............agcgagcaaagaagugguUu..........................................   2     1     seq
..............agcgagcaaagaagugguauu.........................................   67    0     seq
..............agcgagcaaagaagugguauA.........................................   1     1     seq
..............agcAagcaaagaagugguauu.........................................   1     1     seq
..............agcgagcaaagaagugguauuA........................................   2     1     seq
..............agcgagcaaagaagugguauuu........................................   123   0     seq
..............agcAagcaaagaagugguauuu........................................   1     1     seq
..............agcgagcaaagaaUugguauuu........................................   1     1     seq
..............agcgagcaaagaaguggGauuu........................................   2     1     seq
..............agcgagcaaagaagugguUuuu........................................   1     1     seq
..............agcgagcaaagaagugguauuug.......................................   2     0     seq
..............agcgagcaaagaagugguauuuA.......................................   1     1     seq
..............agcgagcaaagaagugguauuuU.......................................   15    1     seq
...............gcgagcaaagaagugguauuu........................................   1     0     seq
...................................................aauauaacuucuuugaaagcuag..   1     0     seq
....................................................auauaacuucuuugaaagcua...   1     0     seq
....................................................auauaacuucuuugaaagcuag..   33    0     seq
....................................................auauaacuucuuugaaagcuagg.   2     0     seq
....................................................auauaacuucuuugaaagcuagA.   1     1     seq
.....................................................uauaacuucuuugaaagcua...   1     0     seq
.....................................................uauaacuucuuugaaagcuagg.   24    0     seq
.....................................................uauaacuucuuugaaagcuaggA   1     1     seq
```

miRBase precursor : dsi-mir-2499
Total read count : 5963
dsi-miR-2499-5p read count: 5449
dsi-miR-2499-3p read count: 514
remaining reads : 0

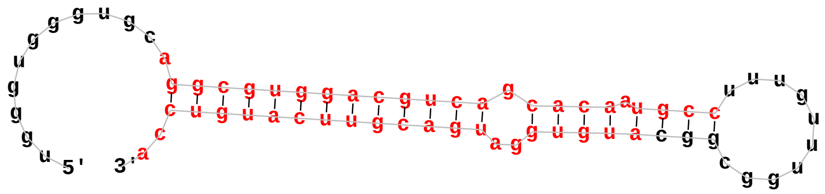

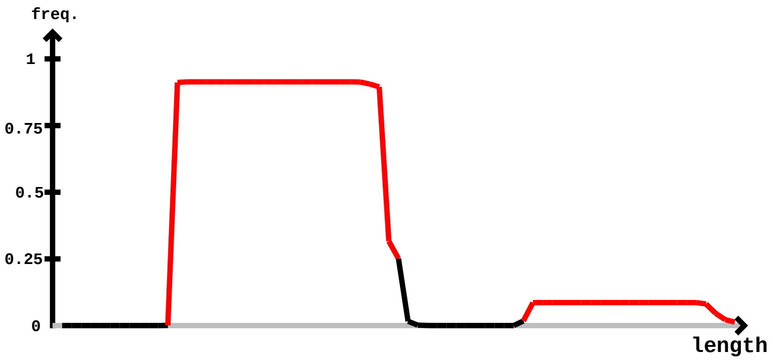


```
                                                dsi-miR-2499-3p
          dsi-miR-2499-5p
5'- ugggugggugcaggcguggacgucagcacaaugccuuuguuuggcggcauguggaugacguucaugucca   -3'    exp
    ............(((((((((((((.((((.((((..........))))))))..)))))))))))))..   reads      mm         sample
    ..........caggcguggacgucagcacaaug.....................................   2          0          seq
    ..........caggcguggacgucagcacaaugc....................................   1          0          seq
    ...........aggcguggacgucagcaca........................................   3          0          seq
    ...........aggcguggacgucagcacaU.......................................   2          1          seq
    ...........aggcguggacgucagcacaa.......................................   43         0          seq
    ...........aggcguggacgucagcacaUu......................................   2          1          seq
    ...........aggcguggacgucagcacaaA......................................   1          1          seq
    ...........aggcguggacgucaAcacaau......................................   1          1          seq
    ...........aggcguggacgucagcacaau......................................   61         0          seq
    ...........aggcguggacgucagcaGaaug.....................................   1          1          seq
    ...........aggcgCggacgucagcacaaug.....................................   2          1          seq
    ...........aggcguggacgucagcacaauU.....................................   51         1          seq
    ...........aAgcguggacgucagcacaaug.....................................   3          1          seq
    ...........aggcguggacguAagcacaaug.....................................   2          1          seq
    ...........aggcguggacgucagAacaaug.....................................   2          1          seq
    ...........aUgcguggacgucagcacaaug.....................................   1          1          seq
    ...........Gggcguggacgucagcacaaug.....................................   3          1          seq
    ...........aggGguggacgucagcacaaug.....................................   1          1          seq
    ...........aggcguggacgucagcacGaug.....................................   1          1          seq
    ...........aggcguggacgucagcacaaAg.....................................   1          1          seq
    ...........aggcguggacgucagcacaaug.....................................   3352       0          seq
    ...........aggcguggacguUagcacaaug.....................................   1          1          seq
    ...........aggcguggacgucagcaUaaug.....................................   1          1          seq
    ...........agAcguggacgucagcacaaug.....................................   3          1          seq
    ...........aggcguggacgucagcacaauA.....................................   6          1          seq
    ...........aggcguggaAgucagcacaaug.....................................   3          1          seq
    ...........aggcguggacgCcagcacaaug.....................................   3          1          seq
    ...........aggcguggacgucagcacCaug.....................................   1          1          seq
    ...........aggAguggacgucagcacaaug.....................................   1          1          seq
    ...........aggcgGggacgucagcacaaug.....................................   3          1          seq
    ...........aggcguggacgucagcUcaaug.....................................   1          1          seq
    ...........aggcguggacgucCgcacaaug.....................................   1          1          seq
    ...........aggcguggacgucagcacUaug.....................................   1          1          seq
    ...........aggcguggacgucagcaAaaug.....................................   1          1          seq
```

dsi-miR-2499-3p

dsi-miR-2499-5p

ugggugggugcaggcguggacgucagcacaaugccuuuguuuggcggcauguggaugacguucaugucca

...........aggcgugCacgucagcacaaug.....................................	1	1	seq
...........aUgcguggacgucagcacaaugc....................................	1	1	seq
...........aggcguggacgucagcacaaugc....................................	278	0	seq
...........aggAguggacgucagcacaaugc....................................	1	1	seq
...........aggcguggacgucagcacaaGgc....................................	1	1	seq
...........aggcgGggacgucagcacaaugc....................................	1	1	seq
...........aggcguggacgucagcacaaugA....................................	4	1	seq
...........aggcguggacgucagcacaauCc....................................	1	1	seq
...........aggcguggacgucagcacaaugU....................................	95	1	seq
...........aggcguggacAucagcacaaugc....................................	1	1	seq
...........aggcguggacgucagcacaauUc....................................	1	1	seq
...........aggcgAggacgucagcacaaugcc...................................	1	1	seq
...........aggcguggacguUagcacaaugcc...................................	2	1	seq
...........aggcguggacgCcagcacaaugcc...................................	1	1	seq
...........aggcguggacgucagcacaaugcA...................................	7	1	seq
...........aggcguggacgucagcacaaugcc...................................	1336	0	seq
...........aggcguggaAgucagcacaaugcc...................................	2	1	seq
...........aggcguggacgucagcacaauCcc...................................	1	1	seq
...........aggcguggacgucagcCcaaugcc...................................	1	1	seq
...........Cggcguggacgucagcacaaugcc...................................	1	1	seq
...........aggcguggacgucagcacaaAgcc...................................	1	1	seq
...........aggcguggacguAagcacaaugcc...................................	2	1	seq
...........aUgcguggacgucagcacaaugcc...................................	1	1	seq
...........aggcguggacgucagcacaaugcG...................................	1	1	seq
...........aggcUuggacgucagcacaaugcc...................................	1	1	seq
...........aggcguggacgucagcacaaugcU...................................	33	1	seq
...........aggAguggacgucagcacaaugcc...................................	1	1	seq
...........aggcguggacgucagcacaaugcAu..................................	1	1	seq
...........aggcguggacgucagcacaaugcUu..................................	6	1	seq
...........aggcguggacgucagcacaaugccu..................................	53	0	seq
...........aggcguggacgucagcacaaGgccu..................................	1	1	seq
...........aggcguggacgucagcacaaugccA..................................	25	1	seq
...........aggcguggacgucagcacaaugcUuu.................................	5	1	seq
...........aggcguggacgucagcacaaugccuu.................................	3	0	seq
...........aggcguggacgucagcacaaugccAu.................................	1	1	seq
...........aggcguggacgucagcacaaugcUuuu................................	1	1	seq
............ggcguggacgucagcacaaugc....................................	3	0	seq
............ggcgAggacgucagcacaaugc....................................	1	1	seq
............ggcguggacgucagcacaaugcc...................................	10	0	seq
....cauguggaugacguucau.....	1	0	seq
....cauguggaugacguucaug....	15	0	seq
....cauguggaugacguucauU....	1	1	seq
....cauguggaugacguucaugu...	27	0	seq
....cauguggaugacguucaugA...	1	1	seq
....cauguggaugacguucauguU..	2	1	seq
....cauguggaugacguucauguc..	24	0	seq
....cauguggaugacguucaugucc.	12	0	seq
....cauguggaugacguucauguUc.	1	1	seq
....cauguggaugacguucaugucca	20	0	seq
....aAguggaugacguucaug....	1	1	seq
....auguggaugacguucaug....	12	0	seq
....auguggaugaUguucaugu...	1	1	seq
....auAuggaugacguucaugu...	1	1	seq
....auguggaugacguucaugu...	163	0	seq
....auguCgaugacguucaugu...	1	1	seq
....augGggaugacguucaugu...	1	1	seq
....auguggaugacguucauUu...	10	1	seq
....auguggaugGcguucaugu...	1	1	seq
....auguggaugacguucaugA...	1	1	seq
....auguggaugacgAucaugu...	2	1	seq
....auguggaugacguucauguU..	95	1	seq
....auguggaugacguucauguA..	4	1	seq
....auguggaugacguucauguc..	17	0	seq
....auguggaugacguucaugucA.	1	1	seq
....auguggaugacguucaugucc.	32	0	seq
....auguggaugacguucGugucc.	1	1	seq
....auguggaugacguucaugucU.	8	1	seq
....auguggaugacguucauguccU	1	1	seq
....auguggaugacguucaugucca	55	0	seq

dsi-miR-2499-3p

dsi-miR-2499-5p

ugggugggugcaggcguggacgucagcacaaugccuuuguuuggcggcauguggaugacguucaugucca

....uguggaugacguucaugucca 2 0 seq

miRBase precursor : dsi-mir-4966-1
Total read count : 439
dsi-miR-4966-5p read count: 367
dsi-miR-4966-3p read count: 72
remaining reads : 0

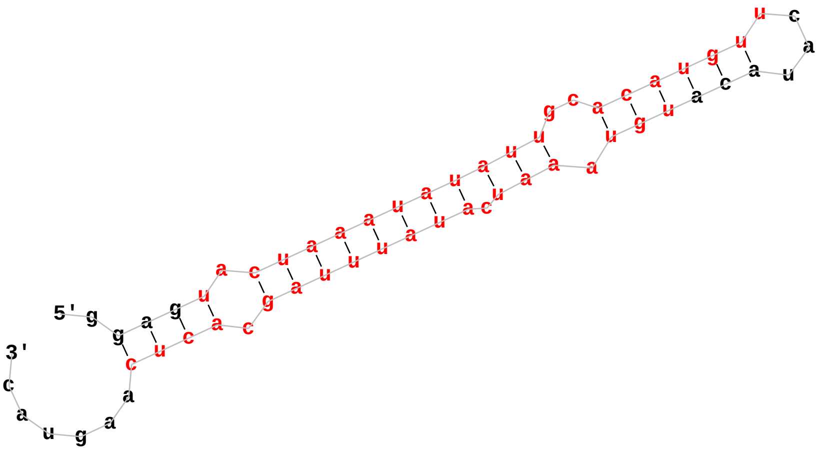

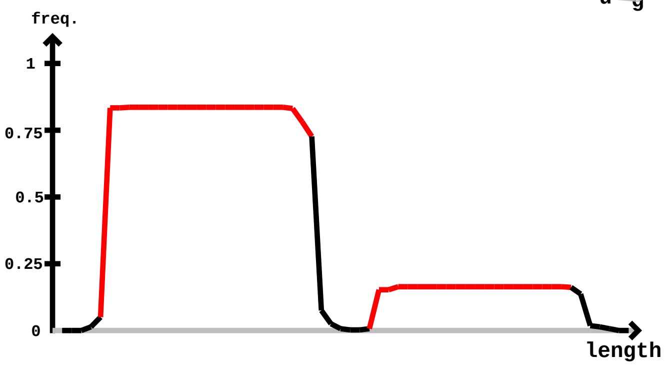


```
                                  dsi-miR-4966-3p
        dsi-miR-4966-5p
5'- ggaguacuaaauauauugcacauguucauacauguaaaucauauuuagcacucaaguac   -3'    exp
    .((((.(((((((((((..((((((....)))))).))).)))))))).))))......    reads      mm         sample
    ..aguacuaaauauauugcacaug...................................    5          0          seq
    ..aAuacuaaauauauugcacauguu.................................    1          1          seq
    ...guacuaaauauauugcacaug...................................    6          0          seq
    ...guacuaaauauauugcacaugu..................................    8          0          seq
    ...Auacuaaauauauugcacauguu.................................    2          1          seq
    ....uacuaaauauauugcacaA....................................    2          1          seq
    ....uacuaaauauauugcacaug...................................    11         0          seq
    ....uacuaaauauauugcacaugu..................................    15         0          seq
    ....uacuaaauauauugcacauUu..................................    1          1          seq
    ....uacuaaauauauugcacauguA.................................    3          1          seq
    ....uacuaaauauauugcacauUuu.................................    1          1          seq
    ....uacuaaauauauugcacaAguu.................................    1          1          seq
    ....uacuaaauauauugAacauguu.................................    1          1          seq
    ....uGcuaaauauauugcacauguu.................................    1          1          seq
    ....uacuaaauauauugcacauguu.................................    272        0          seq
    ....uacAaaauauauugcacauguu.................................    1          1          seq
    ....uacuCaauauauugcacauguu.................................    1          1          seq
    ....uacuaaauauGuugcacauguu.................................    1          1          seq
    ....uacuaaauauauugcacauguuA................................    8          1          seq
    ....uacuaaauauauugcacauguuc................................    4          0          seq
    ....uacuaaauauauugcacauguuU................................    10         1          seq
    ....uacuaaauauauugcacauguuAa...............................    3          1          seq
    ....uacuaaauauauugcacauguuUa...............................    1          1          seq
    ....uacuaaauauauugcacauguuca...............................    4          0          seq
    ....uacuaaauauauugcacauguucau..............................    2          0          seq
    ....uacuaaauauauugcacauguucaua.............................    1          0          seq
    ......cuaaauauauugcacauguu.................................    1          0          seq
    ..............................cauguaaaucauauuuagcacu.......    1          0          seq
    ...............................auguaaaucauauuuagcacuca.....    1          0          seq
    ...............................auguaaaucauauuuagcacucaaA...    1          1          seq
    ................................uguaaaucauauuuagcacuc......    10         0          seq
    ................................uguaaaucauauuuGgcacuc......    1          1          seq
    ................................uguaaaucauauuuagAacuca.....    1          1          seq
    ................................uguaaaucauauuuagcaUuca.....    2          1          seq
```

```
                                dsi-miR-4966-3p
    dsi-miR-4966-5p
ggaguacuaaauauauugcacauguucauacauguaaaucauauuuagcacucaaguac

................................uguaaaucauauuuagcacuca.....    48    0    seq
................................uguaaaucauauuuagcacucaa....    2     0    seq
..................................uaaaucauauuuagcacucaag...    1     0    seq
..................................uaaaucauauuuagcacucaaA...    1     1    seq
..................................uaaaucauauuuagcacucaagu..    3     0    seq
```

miRBase precursor : dsi-mir-4966-2
Total read count : 732
dsi-miR-4966-5p read count: 657
dsi-miR-4966-3p read count: 72
remaining reads : 3

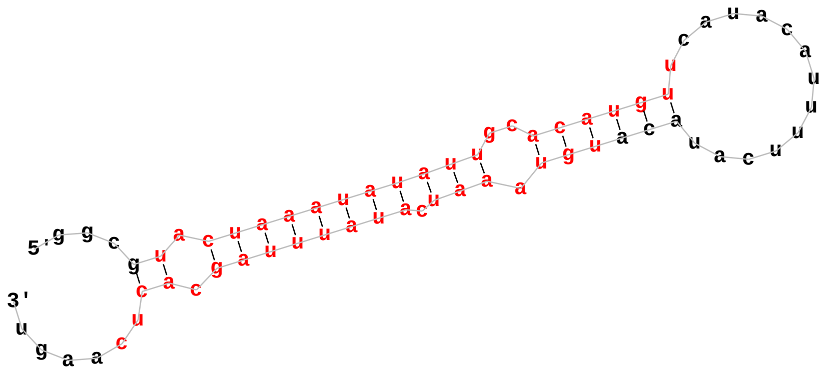

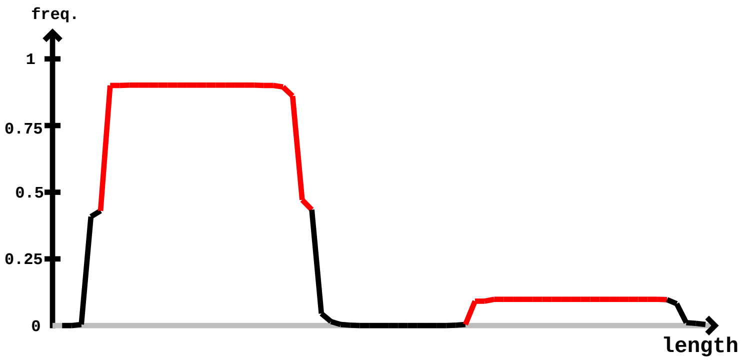


```
                                                     dsi-miR-4966-3p
        dsi-miR-4966-5p
5'- ggcguacuaaauauauugcacauguucauacauuuucauacauguaaaucauauuuagcacucaagu   -3'    exp
    ((.((.(((((((((((..((((((..............)))))).))).)))))))).))))....   reads     mm        sample
    .Acguacuaaauauauugcacau............................................   3         1         seq
    ..cguacuaaauauauugca...............................................   1         0         seq
    ..cguacuaaauauauugcaca.............................................   4         0         seq
    ..cguacuaaauauauugcacau............................................   20        0         seq
    ..cguacuaaauauaAugcacaug...........................................   1         1         seq
    ..cguacuaaauauauugcacauA...........................................   1         1         seq
    ..cguacuaaauaAauugcacaug...........................................   1         1         seq
    ..cguacuaaauauauugcacaAg...........................................   1         1         seq
    ..cguacuaaauauauugcacauU...........................................   2         1         seq
    ..cguacuaaauauauugcacaug...........................................   262       0         seq
    ..cguacuaaauauauugcacaugA..........................................   1         1         seq
    ..cguacuaaauauauugcacaugu..........................................   2         0         seq
    ...guacuaaauauauugcacaug...........................................   6         0         seq
    ...guacuaaauauauugcacaugu..........................................   8         0         seq
    ...Auacuaaauauauugcacauguu.........................................   2         1         seq
    ....uacuaaauauauugcacaA............................................   2         1         seq
    ....uacuaaauauauugcacaug...........................................   11        0         seq
    ....uacuaaauauauugcacaugu..........................................   15        0         seq
    ....uacuaaauauauugcacauUu..........................................   1         1         seq
    ....uacuaaauauauugcacauguu.........................................   272       0         seq
    ....uacuaaauauauugAacauguu.........................................   1         1         seq
    ....uacuaaauauauugcacauUuu.........................................   1         1         seq
    ....uGcuaaauauauugcacauguu.........................................   1         1         seq
    ....uacuaaauauauugcacaAguu.........................................   1         1         seq
    ....uacuaaauauGuugcacauguu.........................................   1         1         seq
    ....uacAaaauauauugcacauguu.........................................   1         1         seq
    ....uacuaaauauauugcacauguA.........................................   3         1         seq
    ....uacuCaauauauugcacauguu.........................................   1         1         seq
    ....uacuaaauauauugcacauguuA........................................   8         1         seq
    ....uacuaaauauauugcacauguuU........................................   10        1         seq
    ....uacuaaauauauugcacauguuc........................................   4         0         seq
    ....uacuaaauauauugcacauguuUa.......................................   1         1         seq
    ....uacuaaauauauugcacauguuca.......................................   4         0         seq
    ....uacuaaauauauugcacauguuAa.......................................   3         1         seq
```

```
                                         dsi-miR-4966-3p
    dsi-miR-4966-5p
ggcguacuaaaauauauugcacauguucauacauuuucauacauguaaaucauauuuagcacucaagu

....uacuaaaauauauugcacauguucau.....................................    2           0           seq
....uacuaaaauauauugcacauguucaua....................................    1           0           seq
......cuaaaauauauugcacauguu........................................    1           0           seq
........................................cauguaaaucauauuuagcacu.....    1           0           seq
.........................................auguaaaucauauuuagcacuca...    1           0           seq
.........................................auguaaaucauauuuagcacucaaA.    1           1           seq
..........................................uguaaaucauauuuagcacuc....    10          0           seq
..........................................uguaaaucauauuuGgcacuc....    1           1           seq
..........................................uguaaaucauauuuagAacuca...    1           1           seq
..........................................uguaaaucauauuuagcaUuca...    2           1           seq
..........................................uguaaaucauauuuagcacuca...    48          0           seq
..........................................uguaaaucauauuuagcacucaa..    2           0           seq
............................................uaaaucauauuuagcacucaaA.    1           1           seq
............................................uaaaucauauuuagcacucaag.    1           0           seq
............................................uaaaucauauuuagcacucaagu    3           0           seq
```

miRBase precursor : dsi-mir-4966-3
Total read count : 2206
dsi-miR-4966-5p read count : 367
dsi-miR-4966-3-3p read count : 1839
remaining reads : 0

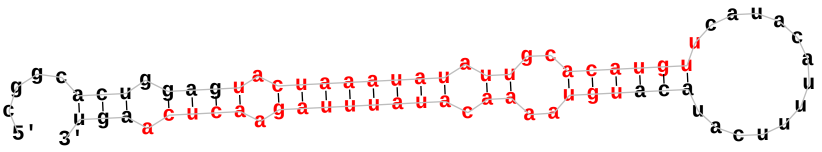

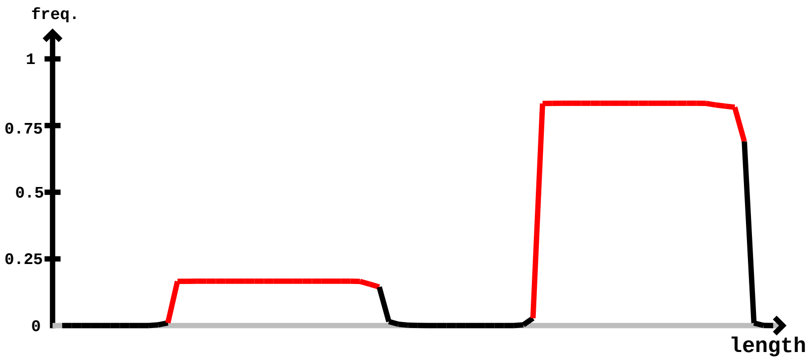


```
                                                  dsi-miR-4966-3-3p
              dsi-miR-4966-5p
5'- cggcacuggaguacuaaaauauauugcacauguucauacauuuucauacauguaaaacauauuuagaacucaagu   -3'    exp
    ....(((.((((.((((((((.((..((((((.............))))))..)).)))))))).)))).)))   reads      mm         sample
    .........aguacuaaaauauauugcacaug.........................................   5          0          seq
    .........aAuacuaaaauauauugcacauguu.......................................   1          1          seq
    ..........guacuaaaauauauugcacaug.........................................   6          0          seq
    ..........guacuaaaauauauugcacaugu........................................   8          0          seq
    ..........Auacuaaaauauauugcacauguu.......................................   2          1          seq
    ...........uacuaaaauauauugcacaA..........................................   2          1          seq
    ...........uacuaaaauauauugcacaug.........................................   11         0          seq
    ...........uacuaaaauauauugcacauUu........................................   1          1          seq
    ...........uacuaaaauauauugcacaugu........................................   15         0          seq
    ...........uacuCaauauauugcacauguu........................................   1          1          seq
    ...........uacuaaaauauauugcacaAguu.......................................   1          1          seq
    ...........uacuaaaauauauugcacauguu.......................................   272        0          seq
    ...........uGcuaaaauauauugcacauguu.......................................   1          1          seq
    ...........uacuaaaauauauugAacauguu.......................................   1          1          seq
    ...........uacAaaauauauugcacauguu........................................   1          1          seq
    ...........uacuaaaauauauugcacauUuu.......................................   1          1          seq
    ...........uacuaaaauauGuugcacauguu.......................................   1          1          seq
    ...........uacuaaaauauauugcacauguA.......................................   3          1          seq
    ...........uacuaaaauauauugcacauguuc......................................   4          0          seq
    ...........uacuaaaauauauugcacauguuA......................................   8          1          seq
    ...........uacuaaaauauauugcacauguuU......................................   10         1          seq
    ...........uacuaaaauauauugcacauguuAa.....................................   3          1          seq
    ...........uacuaaaauauauugcacauguuca.....................................   4          0          seq
    ...........uacuaaaauauauugcacauguuUa.....................................   1          1          seq
    ...........uacuaaaauauauugcacauguucau....................................   2          0          seq
    ...........uacuaaaauauauugcacauguucaua...................................   1          0          seq
    .............cuaaaauauauugcacauguu.......................................   1          0          seq
    ...............................................cauguaaaacauauuuagaacu....   6          0          seq
    ................................................auguaaaacauauuuaga.......   1          0          seq
    ................................................auguaaaacauauuuagaa......   5          0          seq
    ................................................auguaaaacauauuuagaacuc...   18         0          seq
    ................................................auguaaaacauauuuagaacuca..   29         0          seq
    ................................................auguaaaacauauuuagaGcucaa.   2          1          seq
    .................................................uguaaaacauauuuagaa......   8          0          seq
```

```
                                                  dsi-miR-4966-3-3p
           dsi-miR-4966-5p
cggcacuggaguacuaaaauauauugcacauguucauacauuuucauacauguaaaacauauuuagaacucaagu

..................................................uguaaaacauauuuagaac......    10      0       seq
..................................................uguaaaacauauuuagaacu.....    3       0       seq
..................................................uguaaaacauauuuagaaGuc....    1       1       seq
..................................................uguaaaCcauauuuagaacuc....    1       1       seq
..................................................uguGaaacauauuuagaacuc....    1       1       seq
..................................................uguaaaacauauuuagaacuU....    1       1       seq
..................................................uguaaaacauauuuagaacuA....    1       1       seq
..................................................uguaaaacauGuuuagaacuc....    1       1       seq
..................................................uguaaaacauauuuagaacuc....    258     0       seq
..................................................uguaaaaAauauuuagaacuc....    1       1       seq
..................................................uCuaaaacauauuuagaacuca...    1       1       seq
..................................................uguUaaacauauuuagaacuca...    1       1       seq
..................................................Gguaaaacauauuuagaacuca...    1       1       seq
..................................................uguaaaacauauuuagGacuca...    1       1       seq
..................................................uguaaUacauauuuagaacuca...    1       1       seq
..................................................uguaaaacauauuuagaGcuca...    1       1       seq
..................................................uguaaGacauauuuagaacuca...    1       1       seq
..................................................uguaaaacauauuuagaaGuca...    2       1       seq
..................................................uguaaaacauauuuagaacucC...    1       1       seq
..................................................uguGaaacauauuuagaacuca...    2       1       seq
..................................................uguaaaacauauuuagaacuGa...    1       1       seq
..................................................uguaaaacauauuuagaacucU...    5       1       seq
..................................................uguaaaacauGuuuagaacuca...    1       1       seq
..................................................uguaaaacauauuuagaacuca...    1443    0       seq
..................................................uguaaaacauauuuagaaUuca...    3       1       seq
..................................................uguaaaacauauuuGgaacuca...    1       1       seq
..................................................uguaaaacauUuuuagaacuca...    1       1       seq
..................................................uguaaaUcauauuuagaacuca...    1       1       seq
..................................................uguaaaacauauuuaCaacuca...    2       1       seq
..................................................uAuaaaacauauuuagaacuca...    1       1       seq
..................................................uguaaaacauauuuagaaAuca...    1       1       seq
..................................................uguaaaacauauuuagaacucaU..    11      1       seq
..................................................uguaaaacauauuuagaacucaa..    6       0       seq
..................................................uguaaaacauauuuagaacucaaA.    1       1       seq
...................................................guaaaacauauuuagaacucaU..    1       1       seq
....................................................uaaaacauauuuagaacuca...    1       0       seq
```

miRBase precursor : dsi-mir-975
Total read count : 19201
dsi-miR-975-5p read count : 14125
dsi-miR-975-3p read count : 5070
remaining reads : 6

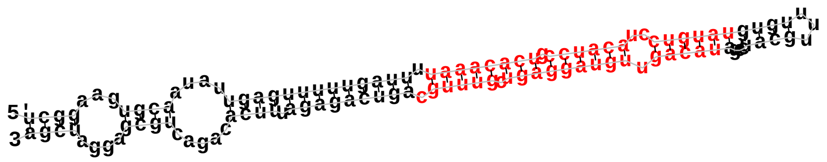

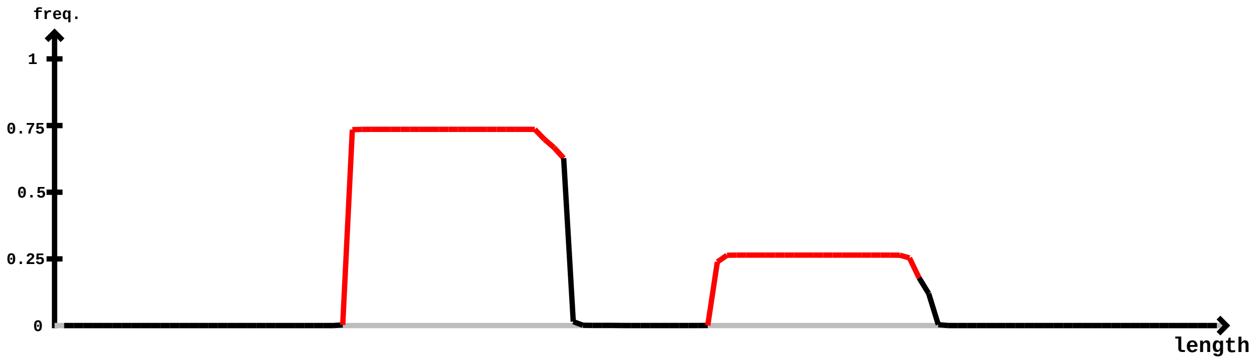


```
                                  dsi-miR-975-5p
                                                                       dsi-miR-975-3p
5'- ucggaagugcaauauugaguuuuugauuuuaaacacugccuacauccuguauguguuuugcauccgauacaguuguaggagucguuugcagucagagauuucacagacugcgaggaucga   -3'    exp
    ((((...((((....((((((((((((.((((((((.((((((..((((((((((...))))...)))))).)))))))))).))))).))))))))).))))).....))))....))))    reads      mm         sa
    ............................uuaaacacugccuacauccu........................................................................    1          0          se
    ............................Auaaacacugccuacauccug.......................................................................    2          1          se
    ............................uuaaacacugccuacauccug.......................................................................    13         0          se
    ............................uuaaacacugccuacauccugu......................................................................    16         0          se
    ............................Auaaacacugccuacauccugu......................................................................    1          1          se
    ............................uuaaacacugccuacauccugua.....................................................................    4          0          se
    ............................Cuaaacacugccuacauccuguau....................................................................    3          1          se
    ............................uuaaacacugccuacauccuguaA....................................................................    3          1          se
    ............................Auaaacacugccuacauccuguau....................................................................    3          1          se
    .............................uaaacacugccuacauccA........................................................................    1          1          se
    .............................uaaacacugccuacauccu........................................................................    2          0          se
    .............................uaaacGcugccuacauccug.......................................................................    1          1          se
    .............................uaaacacugccuacauccug.......................................................................    645        0          se
    .............................uUaacacugccuacauccug.......................................................................    1          1          se
    .............................uaaacacugccuacauccuA.......................................................................    4          1          se
    .............................uaaacacugccuacauccAg.......................................................................    1          1          se
    .............................uaaacacuAccuacauccug.......................................................................    1          1          se
    .............................uaaacacugcGuacauccug.......................................................................    1          1          se
    .............................uaaacaAugccuacauccug.......................................................................    1          1          se
    .............................uaaacacugccuacaucUug.......................................................................    1          1          se
    .............................uaaacacugccuacauAcug.......................................................................    2          1          se
    .............................uaaacacugccuacauccuU.......................................................................    44         1          se
    .............................uaaacacugccuaAauccug.......................................................................    1          1          se
    .............................uaaacacugccuacauccuAu......................................................................    5          1          se
    .............................uaaGcacugccuacauccugu......................................................................    1          1          se
    .............................uaaacacugccuacauccugC......................................................................    1          1          se
    .............................uaaacacugccuacauccugA......................................................................    12         1          se
    .............................uaaacacugccuacauccugu......................................................................    537        0          se
    .............................uaaacGcugccuacauccugu......................................................................    2          1          se
    .............................uaaacacugccuaAauccugu......................................................................    1          1          se
    .............................uaaacaUugccuacauccugu......................................................................    1          1          se
    .............................uaaacacugccuacauccuUu......................................................................    15         1          se
    .............................uaaacacugccuacauccugua.....................................................................    660        0          se
    .............................uaaacacugGcuacauccugua.....................................................................    1          1          se
```

```
                                                 dsi-miR-975-5p
                                                                                       dsi-miR-975-3p
ucggaagugcaauauugaguuuuugauuuuaaacacugccuacauccuguauguguuuugcauccgauacaguuguaggagucguuugcagucagagauuucacagacugcgaggaucga

.............................uaaacacugccuacauccugCa.....................................................................   1       1       se
.............................uaaacacugccuacauUcugua.....................................................................   1       1       se
.............................uaaacacugccuacauccuguC.....................................................................   1       1       se
.............................uaaacacugccuacauccugAa.....................................................................   5       1       se
.............................uaaacacugccuacauccuguU.....................................................................   85      1       se
.............................uaaacacugcUuacauccugua.....................................................................   1       1       se
.............................uaaacacugccuacauccuguG.....................................................................   1       1       se
.............................uaaacUcugccuacauccugua.....................................................................   1       1       se
.............................uaaacacugccuacauAcuguau....................................................................   3       1       se
.............................uaaacacugccuacauccuAuau....................................................................   2       1       se
.............................Aaaacacugccuacauccuguau....................................................................   2       1       se
.............................uGaacacugccuacauccuguau....................................................................   4       1       se
.............................uaaacaUugccuacauccuguau....................................................................   4       1       se
.............................uaCacacugccuacauccuguau....................................................................   1       1       se
.............................uaaacacugccuacauccugAau....................................................................   3       1       se
.............................uaGacacugccuacauccuguau....................................................................   5       1       se
.............................uaaacGcugccuacauccuguau....................................................................   3       1       se
.............................uaaacacugccuacauccugCau....................................................................   5       1       se
.............................uaaacacugccuacauccAguau....................................................................   3       1       se
.............................uaaacacugUcuacauccuguau....................................................................   2       1       se
.............................uaaacacAgccuacauccuguau....................................................................   2       1       se
.............................uaaacacugccuacaGccuguau....................................................................   2       1       se
.............................uaaacacugccuacauccCguau....................................................................   1       1       se
.............................uaaUcacugccuacauccuguau....................................................................   2       1       se
.............................uaaacacugccuacauccuguUu....................................................................   47      1       se
.............................uaaacacugccuacauccuguaA....................................................................   160     1       se
.............................uaaGcacugccuacauccuguau....................................................................   8       1       se
.............................uaUacacugccuacauccuguau....................................................................   1       1       se
.............................uaaacacugcGuacauccuguau....................................................................   3       1       se
.............................uUaacacugccuacauccuguau....................................................................   2       1       se
.............................uaaacacugccuacauccuguau....................................................................   11386   0       se
.............................uaaacaAugccuacauccuguau....................................................................   2       1       se
.............................uaaacacugccuacauccuguCu....................................................................   2       1       se
.............................uaaacacugAcuacauccuguau....................................................................   4       1       se
.............................uCaacacugccuacauccuguau....................................................................   1       1       se
.............................uaaacacuCccuacauccuguau....................................................................   1       1       se
.............................uaaacacugccuacauccuCuau....................................................................   1       1       se
.............................uaaacacugccuacaAccuguau....................................................................   1       1       se
.............................uaaacUcugccuacauccuguau....................................................................   3       1       se
.............................uaaacacugccuacauUcuguau....................................................................   10      1       se
.............................uaaCcacugccuacauccuguau....................................................................   2       1       se
.............................uaaacacugccuaGauccuguau....................................................................   3       1       se
.............................uaaaAacugccuacauccuguau....................................................................   8       1       se
.............................uaaacacugccuacauccuUuau....................................................................   1       1       se
.............................uaaacacugccuacauccuguaC....................................................................   5       1       se
.............................uaaacacugccuaAauccuguau....................................................................   5       1       se
.............................uaaacacugccuacaucUuguau....................................................................   1       1       se
.............................uaaacacugccuacauccugGau....................................................................   6       1       se
.............................uaaacacugcAuacauccuguau....................................................................   6       1       se
.............................uaaacacugccuUcauccuguau....................................................................   1       1       se
.............................uaaacacugccuacauccuguaG....................................................................   4       1       se
.............................uaaacacugccCacauccuguau....................................................................   2       1       se
.............................uaaacacGgccuacauccuguau....................................................................   2       1       se
.............................uaaacacugccuCcauccuguau....................................................................   1       1       se
.............................uaaacacugccuacCuccuguau....................................................................   1       1       se
.............................uaaacacuAccuacauccuguau....................................................................   5       1       se
.............................uaaacacugccuacauccuguGu....................................................................   7       1       se
.............................uaaacacugccuacauccGguau....................................................................   5       1       se
.............................uaaacacugccuacGuccuguau....................................................................   1       1       se
.............................uaaacacugccuacauccuguaug...................................................................   18      0       se
.............................uaaacacugccuacauccuguauU...................................................................   166     1       se
.............................uaaacacugccuacauccuguauA...................................................................   72      1       se
.............................uaaacacugccuacauccuguaugA..................................................................   1       1       se
.............................uaaacacugccuacauccuguauUu..................................................................   10      1       se
.............................uaaacacugccuacauccuguauguguu...............................................................   8       0       se
.............................uaaacacugccuacauccuguauUuguuu..............................................................   1       1       se
.............................uaaacacugccuacauccuguauguguuu..............................................................   3       0       se
..............................aaacacugccuacauccug.......................................................................   1       0       se
..............................aaacacugccuacauccuguU.....................................................................   1       1       se
```

```
                             dsi-miR-975-5p
                                                                   dsi-miR-975-3p
ucggaagugcaauauugaguuuuugauuuuaaacacugccuacauccuguauguguuuugcauccgauacaguuguaggagucguuugcagucagagauuucacagacugcgaggaucga

..............................Uaacacugccuacauccugua.....................................................................   1      1      se
..............................Uaacacugccuacauccuguau....................................................................   1      1      se
..............................aaacacugccuacauccuguau....................................................................   10     0      se
..............................aaacacugccuacauccuguauU...................................................................   5      1      se
...............................aacacugccuacauccugu......................................................................   1      0      se
...............................aacacugccuacauccuguau....................................................................   4      0      se
..........................................................ugcauccgauacaguuguaggagucguuu.................................   1      0      se
..............................................................uccgauacaguuguaggagucguuugc...............................   1      0      se
.................................................................gauacaguuguaggagucguuugca..............................   2      0      se
..................................................................auacaguuguaggagucguu..................................   1      0      se
..................................................................Cuacaguuguaggagucguuu.................................   2      1      se
..................................................................Cuacaguuguaggagucguuugca..............................   1      1      se
...................................................................uacaguuguaggagucgu...................................   1      0      se
...................................................................uacaguuguaggagucguu..................................   4      0      se
...................................................................uacaguuguaggagucguuu.................................   188    0      se
...................................................................uacaguuguaggagucguuG.................................   1      1      se
...................................................................uacaguuguaggagucguuug................................   1404   0      se
...................................................................uacaguuguaggaCucguuug................................   2      1      se
...................................................................uacaguuguaggagucguuuA................................   2      1      se
...................................................................uacaguuguaggaguAguuug................................   1      1      se
...................................................................uacaguuguaggagGcguuug................................   2      1      se
...................................................................uacGguuguaggagucguuug................................   1      1      se
...................................................................uacaguuUuaggagucguuug................................   1      1      se
...................................................................uacaguuguaggagucguuuU................................   8      1      se
...................................................................uaAaguuguaggagucguuug................................   1      1      se
...................................................................uacaguuguaggagAcguuug................................   2      1      se
...................................................................uacaguuguaggagucguGug................................   1      1      se
...................................................................uacaguuguaggagucguuugG...............................   1      1      se
...................................................................uacaguuguaggagAcguuugc...............................   2      1      se
...................................................................uacGguuguaggagucguuugc...............................   1      1      se
...................................................................uacaguuguagUagucguuugc...............................   1      1      se
...................................................................uacaguuguaggagucguuugA...............................   30     1      se
...................................................................uacaguuguaggagGcguuugc...............................   1      1      se
...................................................................uacagGuguaggagucguuugc...............................   1      1      se
...................................................................uacaguuguaggagucguuugU...............................   47     1      se
...................................................................uacaguuguagAagucguuugc...............................   1      1      se
...................................................................uacaguuguaggagucguuugc...............................   977    0      se
...................................................................uacaUuuguaggagucguuugc...............................   1      1      se
...................................................................uacaguuguaggagucguuuUc...............................   1      1      se
...................................................................uaGaguuguaggagucguuugc...............................   1      1      se
...................................................................uacaguuguaggagucCuuugc...............................   1      1      se
...................................................................uUcaguuguaggagucguuugca..............................   1      1      se
...................................................................uacaguuguaggagGcguuugca..............................   3      1      se
...................................................................Aacaguuguaggagucguuugca..............................   1      1      se
...................................................................uacaUuuguaggagucguuugca..............................   3      1      se
...................................................................uacaguuguaggagucguuugAa..............................   2      1      se
...................................................................uaAaguuguaggagucguuugca..............................   1      1      se
...................................................................uacaguuguaggGgucguuugca..............................   1      1      se
...................................................................uacaguuguaggagucguGugca..............................   1      1      se
...................................................................uacaguuguaggagAcguuugca..............................   3      1      se
...................................................................uacaguuguaggagucAuuugca..............................   1      1      se
...................................................................uacaguugAaggagucguuugca..............................   1      1      se
...................................................................uacaguCguaggagucguuugca..............................   1      1      se
...................................................................uacaguuguaggagucguuugUa..............................   2      1      se
...................................................................uacaguuguaggagCcguuugca..............................   1      1      se
...................................................................uacaguuguaggagucguuuAca..............................   1      1      se
...................................................................uGcaguuguaggagucguuugca..............................   1      1      se
...................................................................uacaguuguaggagucguuugGa..............................   1      1      se
...................................................................uaGaguuguaggagucguuugca..............................   1      1      se
...................................................................uacaguuguaggagucguuugca..............................   1789   0      se
...................................................................uacaguugGaggagucguuugca..............................   1      1      se
...................................................................uacagCuguaggagucguuugca..............................   1      1      se
...................................................................uacaguuguaggagucgCuugca..............................   1      1      se
...................................................................uacGguuguaggagucguuugca..............................   1      1      se
...................................................................uacaguuguaggagucguuugcU..............................   7      1      se
...................................................................uacaguuguaUgagucguuugca..............................   1      1      se
...................................................................uacaguuguaggagucguuugcaA.............................   15     1      se
...................................................................uacaguuguaggagucguuugcaC.............................   1      1      se
...................................................................uacaguuguaggagucguuugcaU.............................   43     1      se
```

```
                                             dsi-miR-975-5p
                                                                                dsi-miR-975-3p
ucggaagugcaauauugaguuuuugauuuuaaacacugccuacauccuguauguguuuugcauccgauacaguuguaggagucguuugcagucagagauuucacagacugcgaggaucga

...................................................................uacaguuguaggagucguuugcaUu............................    1     1    se
....................................................................acaguuguaggagucguuu.................................    3     0    se
....................................................................acaguuguaggagucguuuA................................    1     1    se
....................................................................acaguuguaggagucguuug................................    8     0    se
....................................................................acaguuguaggagucguuugU...............................    2     1    se
....................................................................acaguuguaggagucguuugc...............................    55    0    se
....................................................................Gcaguuguaggagucguuugc...............................    1     1    se
....................................................................acaguuguaggagucguuCgca..............................    1     1    se
....................................................................acGguuguaggagucguuugca..............................    1     1    se
....................................................................acaAuuguaggagucguuugca..............................    1     1    se
....................................................................acaguuguaggagucguuugca..............................    395   0    se
....................................................................acaguuguaggagCcguuugca..............................    2     1    se
....................................................................acagGuguaggagucguuugca..............................    1     1    se
....................................................................acaguuguaggaguAguuugca..............................    1     1    se
....................................................................acaguuguaggagucguuugcU..............................    1     1    se
....................................................................acaguGguaggagucguuugca..............................    1     1    se
....................................................................acaguuguaggagGcguuugca..............................    1     1    se
....................................................................acaguuguaggagAcguuugca..............................    2     1    se
....................................................................acaguuguaggagucguuugcaU.............................    2     1    se
....................................................................acaguuguaggagucguuugcaA.............................    2     1    se
.....................................................................caguuguaggagucguuug................................    2     0    se
.....................................................................caguuguaggagucguuugc...............................    2     0    se
.....................................................................caguuguaggagucguuugca..............................    4     0    se
.....................................................................caguuguaggagucguuugcag.............................    2     0    se
.....................................................................caguuguaggagucguuugcagA............................    1     1    se
```

miRBase precursor : dsi-mir-976
Total read count : 282
dsi-miR-976-5p read count : 21
dsi-miR-976-3p read count : 260
remaining reads : 1

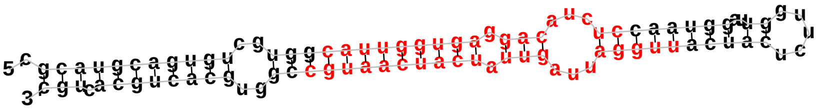

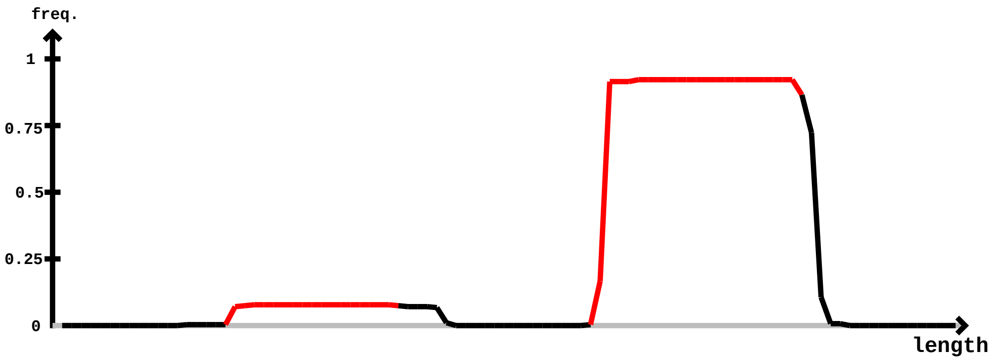

dsi-miR-976-3p

dsi-miR-976-5p

5'- cgcaugcagugucgugg**cauuggugaggacaucuc**caauggauugguucucauca**uuggauuaguuaucaucaaugc**cggugcacugcacugc -3'	exp		
.(((((((((((..((((((((((((.(((...((((((((..((.....))))))))))...))).))))))))))))..)))))))).)))	reads	mm	sample
............cguggcauuggugaggacaucu...	1	0	seq
.................cauuggugaggacaucuU..	1	1	seq
.................cauuggugaggacaucuccaa...	1	0	seq
.................cauuggugaggacaucuccaau..	16	0	seq
.................cauuggugaggacaucuccaauU...	1	1	seq
..................auuggugaggacaucuccaaug...	1	0	seq
...................uuggugaggacaucuccaaug...	1	0	seq
....auuggauuaguuaucaucaaug.................	1	0	seq
....uuggauuaguuaucaucaaug.................	3	0	seq
....uuggauuaguuaucaucaaugc................	18	0	seq
....uuggauuaguuaucaucaaugU................	1	1	seq
....uuggauuaguuaucaucaaugcc...............	19	0	seq
....uuggauuaguuaucaucaaugccU..............	1	1	seq
....uuggauuaguuaucaucaaugccA..............	4	1	seq
....uggauuaguuaucaucaaug.................	12	0	seq
....uggauuaguuaucaucaaugc................	19	0	seq
....uggauuaguuaucaucaaugU................	2	1	seq
....uggauuaguuaucaucaaugcc...............	154	0	seq
....uggauuaguuaucaucaaugcU...............	1	1	seq
....uggauuaguuaucaucaaugccg..............	1	0	seq
....uggauuaguuaucaucaaugccU..............	2	1	seq
....uggauuaguuaucaucaaugccA..............	20	1	seq
....auuaguuaucaucaaugccggu............	2	0	seq

miRBase precursor : dsi-mir-977
Total read count : 1055
dsi-miR-977-5p read count : 15
dsi-miR-977-3p read count : 1039
remaining reads : 1

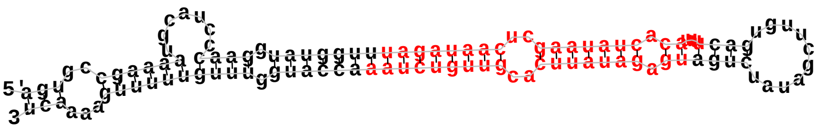

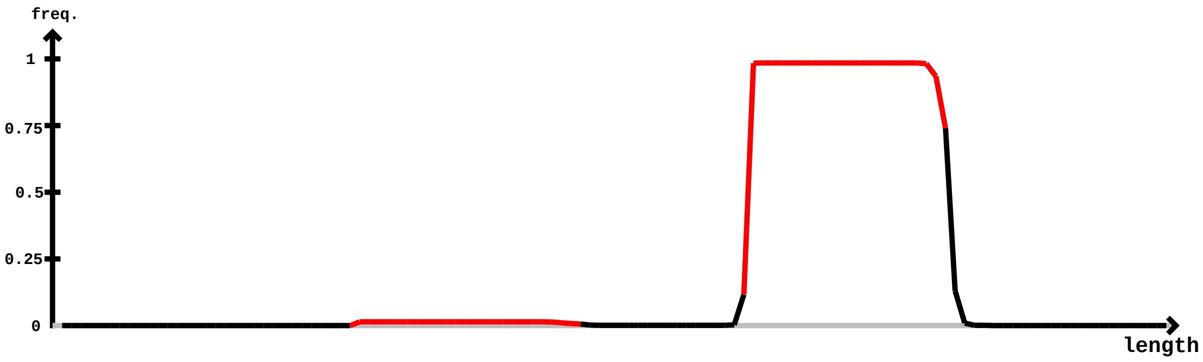


```
                                                                     dsi-miR-977-3p
                              dsi-miR-977-5p
5'- agugccgaaaaugcaucccaagguaugguuuagauaacucgaauaucacauuuucaguguucgauaucugaugagauauucacguugucuaaaccaugguuuguuuuugaaaacu  -3'   exp
    .((((.......))))..((((.(((((((((((((((..(((((((.((...((((..........)))))).))))))).)))))))))))))))).))))............   reads   mm   sample
    ..............................uagauaacucgaauaucaca.................................................................   1       0    seq
    ..............................uagauaacucgaauaucacau................................................................   3       0    seq
    ..............................uagauaacucgaauaucacauu...............................................................   4       0    seq
    ..............................uagauaacucgaauaucacauuu..............................................................   2       0    seq
    ..............................uagauaacucgaauaucacauuuu.............................................................   4       0    seq
    ..............................uagauaacucgaauaucacauuuuc............................................................   1       0    seq
    ....................................................uucaguguucgauaucuga............................................   1       0    seq
    .....................................................................Aaugagauauucacguugucua........................   2       1    seq
    ......................................................................augagauauucacguuguc..........................   1       0    seq
    ......................................................................augagauauucacguuguA..........................   1       1    seq
    ......................................................................augagauauucacguugucu.........................   4       0    seq
    ......................................................................augagauauucacguugucuU........................   2       1    seq
    ......................................................................augagauauucacguugucua........................   26      0    seq
    ......................................................................augagauauucacguugucuaa.......................   64      0    seq
    ......................................................................augagauauucacguugucuaaa......................   19      0    seq
    ......................................................................Gugagauauucacguugucuaaa......................   1       1    seq
    ......................................................................augagauauucacguugucuaaaA.....................   1       1    seq
    ......................................................................augagauauucacguugucuaaacAa...................   1       1    seq
    .......................................................................ugagauauucacguuguc..........................   1       0    seq
    .......................................................................ugagauauucacguugucA.........................   3       1    seq
    .......................................................................ugagauauucacguugucu.........................   43      0    seq
    .......................................................................ugagauauucacguugucua........................   167     0    seq
    .......................................................................ugagauauucacguuguUua........................   1       1    seq
    .......................................................................ugagauauucacguugucuU........................   5       1    seq
    .......................................................................ugagauauucacUuugucua........................   1       1    seq
    .......................................................................ugagauUuucacguugucuaa.......................   1       1    seq
    .......................................................................ugagauauucacguugucuaa.......................   558     0    seq
    .......................................................................ugagauauucacguugucuaU.......................   11      1    seq
    .......................................................................ugaUauauucacguugucuaa.......................   2       1    seq
    .......................................................................ugagauauucacguugucAaa.......................   1       1    seq
    .......................................................................ugaAauauucacguugucuaa.......................   1       1    seq
    .......................................................................ugagauauuAacguugucuaa.......................   1       1    seq
    .......................................................................ugagauauucacUuugucuaa.......................   2       1    seq
    .......................................................................ugagauauucacguugucuaG.......................   1       1    seq
```

```
                                                                       dsi-miR-977-3p
                              dsi-miR-977-5p
agugccgaaaaugcaucccaagguaugguuuagauaacucgaauaucacauuuucaguguucgauaucugaugagauauucacguugucuaaaccaugguuuguuuuugaaaacu

.......................................................................ugagauauAcacguugucuaa.......................    1     1     seq
.......................................................................ugagauauucacguugucGaa.......................    1     1     seq
.......................................................................ugagauauuAacguugucuaaa......................    1     1     seq
.......................................................................ugagauauucacguugucuaCa......................    1     1     seq
.......................................................................ugagauauucacguugucuaaU......................    2     1     seq
.......................................................................ugagauauucacguugucuaaa......................    103   0     seq
.......................................................................ugagauauucacguugucuaaaA.....................    8     1     seq
........................................................................gagauauucacguugucua........................    1     0     seq
```

miRBase precursor : dsi-mir-978a
Total read count : 513
dsi-miR-978a-5p read count: 6
dsi-miR-978a-3p read count: 501
remaining reads : 6

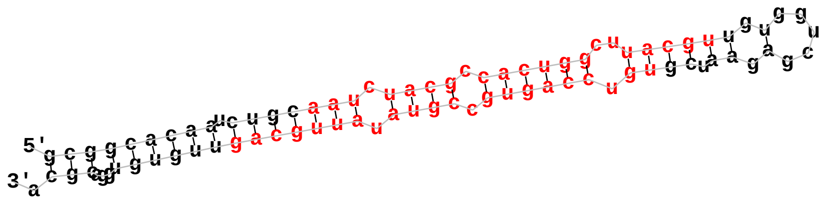

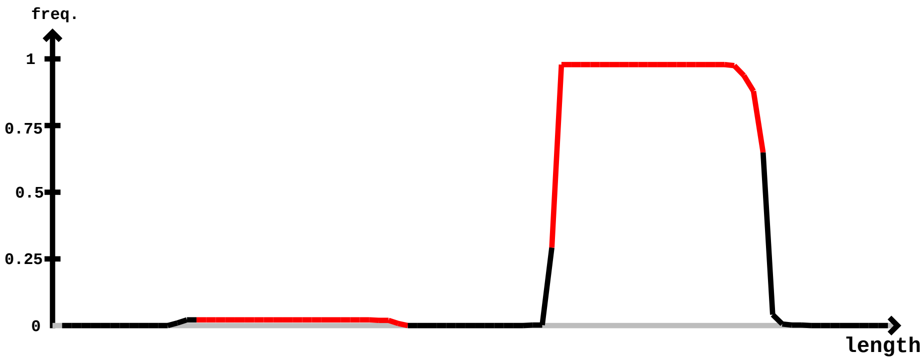

dsi-miR-978a-5p

dsi-miR-978a-3p

```
5'- gcggcacaaucugcaaucuacgccacuggcuuacguuguggucgagaaucguguccagugccguauauugcaguuguguggacgca   -3'    exp
    ((((((((.(((((((.((((.((((((..((((((.(.....).)).)))).)))))).)))).)))))))))))))...))).   reads      mm        sample
    ...........ugcaaucuacgccacuggcuu......................................................   1          0         seq
    ...........ugcaaucuacgccacuggcuuac....................................................   2          0         seq
    ...........ugcaaucuacgccacuggcuuacA...................................................   1          1         seq
    ...........ugcaaucuacgccacuggcuuacg...................................................   1          0         seq
    ............gcaaucuacgccacuggcuuac....................................................   4          0         seq
    ............gcaaucuacgccacuggcuuacg...................................................   2          0         seq
    ................................................Gcguguccagugccguauauu.................   1          1         seq
    ..................................................guguccagugccguauUuu.................   1          1         seq
    ..................................................guguccagugccguauauuU................   2          1         seq
    ..................................................guguccagugccguauauug................   16         0         seq
    ..................................................guguccagugccguauauugc...............   27         0         seq
    ..................................................guguccagugccguauauugca..............   81         0         seq
    ..................................................guguccagugccguauauugcaU.............   2          1         seq
    ..................................................guguccagugccguauauugcaA.............   1          1         seq
    ..................................................guguccagugccguauauugcag.............   17         0         seq
    ..................................................guguccagugccguauauugcagu............   1          0         seq
    ..................................................guguccagugccguauauugcagA............   1          1         seq
    ...................................................uguccagugccguauauug................   1          0         seq
    ...................................................uguccagugccguauauugc...............   3          0         seq
    ...................................................uguccagugccguauGuugca..............   1          1         seq
    ...................................................uguccagugccguauauugca..............   36         0         seq
    ...................................................uguccagugccguauauugcaA.............   3          1         seq
    ...................................................uguccagugccguauauuAcag.............   1          1         seq
    ...................................................uguccagugccguauauugcaU.............   1          1         seq
    ...................................................uguccUgugccguauauugcag.............   1          1         seq
    ...................................................uguccagugccguauauugcag.............   286        0         seq
    ...................................................uguccagugccguauauugcagu............   11         0         seq
    ...................................................uguccagugccguauauugcagA............   4          1         seq
    ...................................................uCuccagugccguauauugcagu............   1          1         seq
    ...................................................uguccagugccguauauugcaguu...........   2          0         seq
    ...................................................uguccagugccguauauugcaguuAu.........   1          1         seq
```

miRBase precursor : dvi-mir-973
Total read count : 1156
dvi-miR-973-5p read count : 69
dvi-miR-973-3p read count : 1087
remaining reads : 0

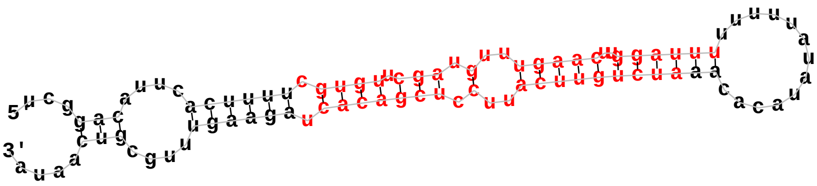

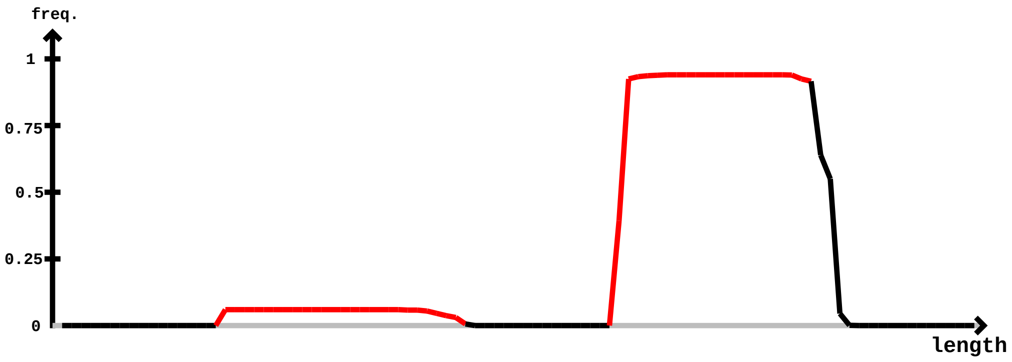


```
                                                            dvi-miR-973-3p
                    dvi-miR-973-5p
5'- ucggacauucacuuuucguguucgauguuugaacuuggauuuuuuuuauauacacaaaucuguucauucccucgacacuagaaguuugcgucaaua  -3'   exp
    ...(((....((((((.((((.(((.(..(((((..((((((..............)))))))))))..).))))))).))))))....)))....   reads   mm   sample
    ................cguguucgauguuugaacu.............................................................   1       0    dv1
    ................cguguucgauguuugaacuugg..........................................................   4       0    dv1
    ................cguguucgauguuugaacuugga.........................................................   3       0    dv1
    ................cguguucgauguuugaacuuggau........................................................   3       0    dv1
    ................cguguucgauguuugaacuuggauu.......................................................   9       0    dv1
    ................cguguucgCuguuugaacuuggauu.......................................................   1       1    dv1
    ................cguguucgauguuugaacuuggauuu......................................................   2       0    dv1
    .........................................................aucuguucauucccucgaca...................   7       0    dv1
    .........................................................aucuguucauucccucgacC...................   3       1    dv1
    .........................................................aucuguucauucccucgacCc..................   1       1    dv1
    .........................................................aucuguucauucccucgacac..................   3       0    dv1
    .........................................................aucuguucauucccucgacCcu.................   4       1    dv1
    .........................................................aucuguucauucccucgCcacu.................   4       1    dv1
    .........................................................aucuguucauucccucUacacu.................   2       1    dv1
    .........................................................aucuguucauucccucgacacu.................   103     0    dv1
    .........................................................aucuguucauucccucgacacC.................   1       1    dv1
    .........................................................aucuguucGuucccucgacacu.................   1       1    dv1
    .........................................................aucuguucauucccucgCcacua................   2       1    dv1
    .........................................................aucuguucauucccucgacacua................   23      0    dv1
    .........................................................aucuguucauucccucgacacuC................   3       1    dv1
    .........................................................aucugCucauucccucgacacuag...............   2       1    dv1
    .........................................................aucuguucauucccucgacacuaU...............   2       1    dv1
    .........................................................aucuguucauucccucgacacuag...............   78      0    dv1
    .........................................................aucuguucauucccucgacCcuag...............   1       1    dv1
    .........................................................aucuguucaCucccucgacacuag...............   1       1    dv1
    .........................................................aucugGucauucccucgacacuag...............   1       1    dv1
    .........................................................Gucuguucauucccucgacacuag...............   1       1    dv1
    .........................................................aucugAucauucccucgacacuag...............   1       1    dv1
    .........................................................aucuguucauucccucgCcacuag...............   1       1    dv1
    .........................................................aucuguucauucccucgacacuCg...............   3       1    dv1
    .........................................................aucuguucauucccucgacacuagU..............   1       1    dv1
    ..........................................................ucuguucauucccucgaca...................   2       0    dv1
    ..........................................................ucuguucauucccucgacac..................   3       0    dv1
    ..........................................................ucuguucauucccucgacCcu.................   2       1    dv1
```

```
                                                         dvi-miR-973-3p
                dvi-miR-973-5p
ucggacauucacuuuucguguucgauguuugaacuuggauuuuuuuuauauacacaaaucuguucauuccucgacacuagaaguuugcgucaaua

..........................................................ucuguucauuAcucgacacu.................   1     1    dv1
..........................................................ucuguucauuccucgacacu.................   57    0    dv1
..........................................................ucuguucauuccucgCcacu.................   3     1    dv1
..........................................................ucuguucauuccucgacacuC................   3     1    dv1
..........................................................ucuguucauuccucgacacua................   20    0    dv1
..........................................................ucCguucauuccucgacacua................   1     1    dv1
..........................................................ucuguucauuccucgCcacua................   1     1    dv1
..........................................................ucuguCcauuccucgacacua................   1     1    dv1
..........................................................ucuguucauuccucgacacuag...............   159   0    dv1
..........................................................ucugCucauuccucgacacuag...............   1     1    dv1
..........................................................ucuguucauuccucgacacuaA...............   1     1    dv1
..........................................................ucuguucUuuccucgacacuag...............   1     1    dv1
..........................................................ucCguucauuccucgacacuag...............   3     1    dv1
..........................................................ucuguucauuccucgCcacuag...............   2     1    dv1
..........................................................ucuguucauuccucgacCcuag...............   4     1    dv1
..........................................................ucuguucauuccucCacacuag...............   1     1    dv1
..........................................................uUuguucauuccucgacacuag...............   1     1    dv1
..........................................................ucuguucauuccucgacacuaU...............   1     1    dv1
..........................................................ucuUuucauuccucgacacuag...............   1     1    dv1
..........................................................ucuguucauuccucgacacuCg...............   12    1    dv1
..........................................................Gcuguucauuccucgacacuag...............   1     1    dv1
..........................................................ucuguucauuccCcgacacuag...............   1     1    dv1
..........................................................ucuguucGuuccucgacacuag...............   1     1    dv1
..........................................................ucuguucauuccucgacacuagU..............   19    1    dv1
...........................................................cuguucauuccucgacacuaU...............   1     1    dv1
...........................................................Auguucauuccucgacacuag...............   1     1    dv1
...........................................................cuguucauuccucgacacuag...............   2     0    dv1
...........................................................cuguucauuccucgacacuagU..............   2     1    dv1
...........................................................cuguucauuccucgacacuagC..............   1     1    dv1
............................................................uguucauuccucgacacua................   1     0    dv1
............................................................uguucauuccucgacacuag...............   1     0    dv1
..............................................................uucauuccucgacacuag...............   2     0    dv1

................cguguucgauguuugaacuug..........................................................   2     0    dv9
................cguguucgauguuugaacuuUg.........................................................   1     1    dv9
................cguguucgauguuugaacuugg.........................................................   3     0    dv9
................cguguucgauguuugaacuugC.........................................................   1     1    dv9
................cguguucgauguuugCacuugga........................................................   1     1    dv9
................cguguucgauguuugaacuugga........................................................   5     0    dv9
................Aguguucgauguuugaacuuggau.......................................................   1     1    dv9
................cguguucgauguuugaacuuggau.......................................................   2     0    dv9
................cguguucgauguuugaacuugUauu......................................................   1     1    dv9
................Aguguucgauguuugaacuuggauu......................................................   1     1    dv9
................cguguucgauguuugGacuuggauu......................................................   1     1    dv9
................cguguucgauguuugaacuuggauu......................................................   11    0    dv9
................cguguucgauguuugaacuuggCuu......................................................   1     1    dv9
................cguguucgauguuugaacuugUauuu.....................................................   1     1    dv9
................cguguucgauguuugaacuuggauuu.....................................................   4     0    dv9
.........................................................aucuguucauuccucgac....................   1     0    dv9
.........................................................aucuguucauuccucgaca...................   3     0    dv9
.........................................................aucuguucauuccucgacC...................   1     1    dv9
.........................................................aucuguucauuccucgacac..................   3     0    dv9
.........................................................aucuguucauuccucUacacu.................   1     1    dv9
.........................................................aucuguucauuccucgacacu.................   67    0    dv9
.........................................................aucuguucauuccucgCcacu.................   5     1    dv9
.........................................................aucuguucauuccucgacCcu.................   2     1    dv9
.........................................................aucuguCcauuccucgacacu.................   1     1    dv9
.........................................................aucuguucauuccucgacCcua................   1     1    dv9
.........................................................aucuguucauuccucgacacuC................   2     1    dv9
.........................................................aucuguucauuccucgacacua................   17    0    dv9
.........................................................aucuguucauuccucgacacuU................   1     1    dv9
.........................................................aucuguucauuccucgacCcuag...............   1     1    dv9
.........................................................aucuguucauuccucgacacuCg...............   5     1    dv9
.........................................................aCcuguucauuccucgacacuag...............   1     1    dv9
.........................................................aucuguucauuccucgacacuag...............   74    0    dv9
.........................................................aucuguucauuccucgCcacuag...............   2     1    dv9
.........................................................aucuguucauuccucUacacuag...............   1     1    dv9
.........................................................aucuguucauuccucgacacuUg...............   1     1    dv9
.........................................................aucuguucauuccucgacacuaC...............   1     1    dv9
```

```
                                                         dvi-miR-973-3p
                dvi-miR-973-5p
ucggacauucacuuuucguguucgauguuugaacuuggauuuuuuuuauauacacaaaucuguucauuccucgacacuagaaguuugcgucaaua

.........................................................aucuguucauuccucgacacuagU..............    10     1     dv9
.........................................................aucuguucauuccucgacacuagaC.............    1      1     dv9
..........................................................ucuguucauuccucgaca...................    1      0     dv9
..........................................................ucuguucauuccucgacCcu.................    3      1     dv9
..........................................................uUuguucauuccucgacacu.................    1      1     dv9
..........................................................ucuguucauuccucgacacu.................    57     0     dv9
..........................................................ucuguucCuuccucgacacu.................    1      1     dv9
..........................................................ucuguucauuccucgacacua................    23     0     dv9
..........................................................ucuguucaCuccucgacacua................    1      1     dv9
..........................................................ucuguucauuccucgacacuC................    1      1     dv9
..........................................................ucuguuUauuccucgacacuag...............    1      1     dv9
..........................................................ucuguucauuccucgacCcuag...............    6      1     dv9
..........................................................ucugGucauuccucgacacuag...............    1      1     dv9
..........................................................ucuguucauuccucgacacuaU...............    1      1     dv9
..........................................................ucuguucauuccucgacacuag...............    175    0     dv9
..........................................................ucuguucauuccucAacacuag...............    1      1     dv9
..........................................................ucuguucauCccucgacacuag...............    1      1     dv9
..........................................................uAuguucauuccucgacacuag...............    1      1     dv9
..........................................................ucuguucauuccucgCcacuag...............    1      1     dv9
..........................................................ucuguucCuuccucgacacuag...............    1      1     dv9
..........................................................ucuguucauuccucgacacuaC...............    1      1     dv9
..........................................................ucuCuucauuccucgacacuag...............    1      1     dv9
..........................................................ucuguucauuccucUacacuag...............    1      1     dv9
..........................................................ucuUuucauuccucgacacuag...............    1      1     dv9
..........................................................Gcuguucauuccucgacacuag...............    1      1     dv9
..........................................................ucuguucauuccucgacacuCg...............    9      1     dv9
..........................................................ucuguucauuccucgacacuagU..............    17     1     dv9
...........................................................cuguucauuccucgCcacu.................    1      1     dv9
...........................................................cuguucauuccucgacacuag...............    1      0     dv9
...........................................................cuguucauuccuUgacacuag...............    1      1     dv9
............................................................uguucauuccucgacacua................    1      0     dv9
.............................................................guucauuccucgacacuag...............    2      0     dv9

................cguguucgauguuugaacu............................................................    1      0     arg
................cguguucgauguuugaacuug..........................................................    2      0     arg
................cguguucgauguuugaacuugg.........................................................    1      0     arg
................cguguucgauguuugaacuugga........................................................    1      0     arg
................cguguucgauguuugaacuuggau.......................................................    2      0     arg
................cguguucgauguuugaacuuggauu......................................................    3      0     arg
.........................................................aucuguucauuccucgacacu.................    2      0     arg
.........................................................aucuguucauuccucgacacuagU..............    1      1     arg
..........................................................ucuguucauuccucgacacu.................    1      0     arg
..........................................................ucuguucauuccucgacacuag...............    3      0     arg
............................................................uguucauuccucgacacua................    1      0     arg
```

miRBase precursor : dvi-mir-974-1
Total read count : 1672
dvi-miR-974-1-5p read count514
dvi-miR-974-1-3p read count1158
remaining reads : 0

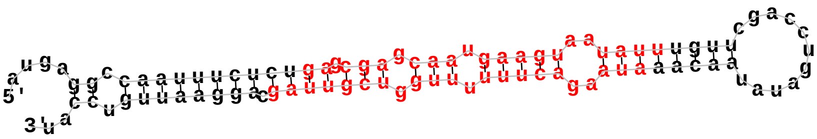

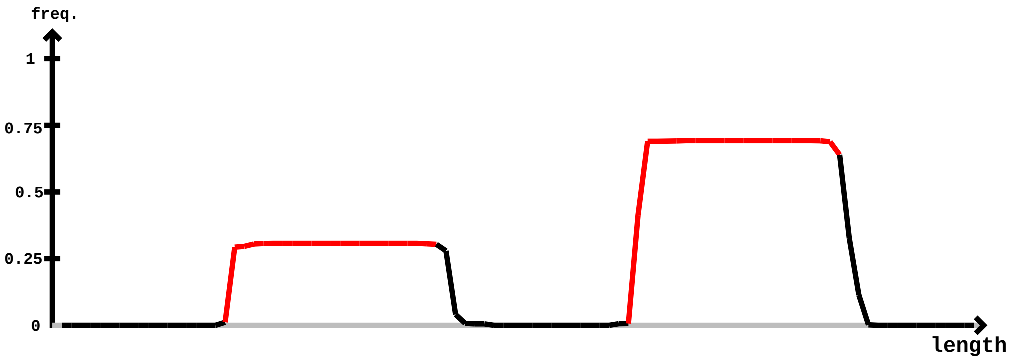


```
                                                                dvi-miR-974-1-3p
                   dvi-miR-974-1-5p
5'- augaggccaauuucucugagcgagcaaugaaguaauauuuguucgaccugauauaacaaauaagacuuuuuuggucguuagcaggaauuguccau  -3'   exp
    ....((.((((((((((((.(((.(((.(((((..((((((((...........))))))))..))))).))).))))))).)))))))).))..   reads   mm   sample
    ................ugagcgagcaaugaaguaauauu........................................................   1       0    dv1
    ................ugagcgagcaaugaaguaauauuC.......................................................   3       1    dv1
    ................ugagcgagcaaugaaguaauauuu.......................................................   1       0    dv1
    .................gagcgagcaaugaaguaauC..........................................................   1       1    dv1
    .................gagcgagcaaugaaguaaua..........................................................   1       0    dv1
    .................gagcgagcaaugaaguaauau.........................................................   1       0    dv1
    .................gagcgagcaaugaaguaauauu........................................................   3       0    dv1
    .................gagcgagcaaugaaguaauauuC.......................................................   34      1    dv1
    .................Uagcgagcaaugaaguaauauuu.......................................................   5       1    dv1
    .................gagcgagcaaugaaguaauGuuu.......................................................   1       1    dv1
    .................gagcgagcaaugaaguCauauuu.......................................................   1       1    dv1
    .................gGgcgagcaaugaaguaauauuu.......................................................   1       1    dv1
    .................gagcgagcaaugaagAaauauuu.......................................................   1       1    dv1
    .................gagcgagcaaugaaguaauauuu.......................................................   89      0    dv1
    .................gagcgagcaaugaaguaauCuuu.......................................................   1       1    dv1
    .................gagcgagcaaugaCguaauauuu.......................................................   2       1    dv1
    .................gagcgagcaaugaaguaauauuuC......................................................   23      1    dv1
    ..................agcgagcaaugaaguaauauuu.......................................................   2       0    dv1
    ...................gcgagcaaugaaguaauauuuguCc...................................................   5       1    dv1
    ....................cgagcaaugaaguaauauuuguCc...................................................   1       1    dv1
    .........................................................aaauaagacuuuuuuggucguu................   1       0    dv1
    .........................................................aaauaagacuuuuuuggucguuC...............   1       1    dv1
    ..........................................................aauaagacuuuuuuggucguua...............   1       0    dv1
    ...........................................................auaagacuuuuuuggucgu.................   1       0    dv1
    ...........................................................auaagacuuuuuuggucguuC...............   2       1    dv1
    ...........................................................auaagacuuuuuuggucguua...............   39      0    dv1
    ...........................................................auaagacuuuuuugUucguuag..............   3       1    dv1
    ...........................................................auaagacuuuuuGggucguuag..............   1       1    dv1
    ...........................................................auaagacuuuuuuggucguuaU..............   11      1    dv1
    ...........................................................auaagacuuuuuuggucguuCg..............   4       1    dv1
    ...........................................................auaagacuuuuuuggucguuag..............   202     0    dv1
    ...........................................................auaagacuCuuuuggucguuag..............   2       1    dv1
    ...........................................................auaagaUuuuuuuggucguuag..............   1       1    dv1
    ...........................................................auaagGcuuuuuuggucguuag..............   2       1    dv1
```

```
                                                                             dvi-miR-974-1-3p
                 dvi-miR-974-1-5p
augaggccaauuucucugagcgagcaaugaaguaauauuuguucgaccugauauaacaaauaagacuuuuuuggucguuagcaggaauuguccau

...........................................................auaagacuuuuuuggucUuuag..............    6     1    dv1
...........................................................auaagacuuuuuuUgucguuag..............    2     1    dv1
...........................................................auaagacuuuuuuCgucguuag..............    1     1    dv1
...........................................................auaagacuuuuuuggucguuaUc.............    4     1    dv1
...........................................................auaagacuuuuuuggucguuagA.............    5     1    dv1
...........................................................auaagacuuuuuuggucguuagc.............    32    0    dv1
...........................................................auaagacuuuuuugUucguuagc.............    1     1    dv1
...........................................................auaagacuuuuuuggucguuagU.............    1     1    dv1
...........................................................auaagacuuuuuuggucguuagcC............    1     1    dv1
............................................................uaagacuuuuuuggucguuC...............    1     1    dv1
............................................................uaagacuuuuuuggucguua...............    2     0    dv1
............................................................uaagacuuuuuuggucguuCg..............    1     1    dv1
............................................................uaagacuuuuuuggucguuag..............    36    0    dv1
............................................................uaagacuuuuuugUucguuag..............    1     1    dv1
............................................................uaagacuuuuuuggucguuaU..............    1     1    dv1
............................................................uaagacuuuuuuggCcguuag..............    1     1    dv1
............................................................uaagacuuuuuuggucguuagU.............    3     1    dv1
............................................................uaagacuuuuuuggucUuuagc.............    1     1    dv1
............................................................uaagacuuuuuuggucguuagA.............    5     1    dv1
............................................................uaagacuuuuuuggucguuagc.............    31    0    dv1
............................................................uaagacuuuuuugUucguuagc.............    2     1    dv1
............................................................uaagacuuuuuuggucguuaUc.............    5     1    dv1
............................................................uaagacuuuuuuggucguuagca............    1     0    dv1
............................................................uaagacuuuuuuggucguuagcU............    2     1    dv1
............................................................uaagacuuuuuuggucguuagcC............    54    1    dv1
............................................................uaagacuuuuuuggucguuagcag...........    1     0    dv1
............................................................uaagacuuuuuuggucguuagcaU...........    2     1    dv1
..............................................................agacuuuuuuggucguuag..............    1     0    dv1
................................................................acuuuuuuggucguuagc.............    2     0    dv1

................ugagcgagcaaugaaguaauauu........................................................    4     0    dv9
................ugagcgagcaaugaaguaauauuC.......................................................    3     1    dv9
.................gagcgagcaaugaaguaauC..........................................................    1     1    dv9
.................gagcgagcaaugaaguaauau.........................................................    2     0    dv9
.................gagcgagcaaugaaguaauauu........................................................    5     0    dv9
.................Uagcgagcaaugaaguaauauu........................................................    1     1    dv9
.................gagcgagcaaugaaguaaCauuu.......................................................    1     1    dv9
.................gagcgagcaaugaaguaCuauuu.......................................................    1     1    dv9
.................gagcgagcaaugCaguaauauuu.......................................................    1     1    dv9
.................gagcgagcaaugaaguaauauuC.......................................................    19    1    dv9
.................gagcgGgcaaugaaguaauauuu.......................................................    1     1    dv9
.................gagcgagcaauAaaguaauauuu.......................................................    1     1    dv9
.................Aagcgagcaaugaaguaauauuu.......................................................    1     1    dv9
.................gagcgagcaaugaaguaauauuu.......................................................    129   0    dv9
.................gagcgagcaaugaCguaauauuu.......................................................    1     1    dv9
.................Uagcgagcaaugaaguaauauuu.......................................................    7     1    dv9
.................gagcgagcCaugaaguaauauuu.......................................................    1     1    dv9
.................gagcgagcaaugaaguaauaAuu.......................................................    1     1    dv9
.................gagcgagcaaugaaguaauauuuC......................................................    21    1    dv9
..................agcgagcaaugaaguaauauuu.......................................................    2     0    dv9
...................gcgagcaaugaaguaauauuug......................................................    1     0    dv9
...................gcgagcaaugaaguaauauuuguCc...................................................    3     1    dv9
....................cgagcaaugaaguaauauuu.......................................................    1     0    dv9
....................cgagcaaugaaguaauauuuC......................................................    1     1    dv9
.....................gagcaaugaaguaGuauuu.......................................................    1     1    dv9
.........................................................aaauaagacuuuuuuggucguu................    2     0    dv9
.........................................................aaauaagacuuuuuuggucguuC...............    2     1    dv9
.........................................................aaauaagacuuuuuuggucguuag..............    1     0    dv9
...........................................................auaagacuuuuuuggucguC................    1     1    dv9
...........................................................auaagacuuuuuuggucguuC...............    1     1    dv9
...........................................................auaagacuuuuuuggucguua...............    27    0    dv9
...........................................................auaagacuuuuuuggucUuua...............    1     1    dv9
...........................................................auaagacuuuuuuggucguuCg..............    2     1    dv9
...........................................................auaagacuuCuuuggucguuag..............    1     1    dv9
...........................................................Cuaagacuuuuuuggucguuag..............    1     1    dv9
...........................................................auaagacuuuuuuggucUuuag..............    2     1    dv9
...........................................................auaagacuuuuuuCgucguuag..............    1     1    dv9
...........................................................auaagacuuuuuugCucguuag..............    1     1    dv9
...........................................................auaagacuuuuCuggucguuag..............    2     1    dv9
```

```
                                                           dvi-miR-974-1-3p
                 dvi-miR-974-1-5p
augaggccaauuucucugagcgagcaaugaaguaauauuuguucgaccugauauaacaaauaagacuuuuuuggucguuagcaggaauuguccau

...........................................................auaagacuuuuuuggucguuag..............    141    0    dv9
...........................................................auaagacuuuuuuggucguuaA..............    1      1    dv9
...........................................................auaagacuuuuuugUucguuag..............    3      1    dv9
...........................................................auaagacuuuuuuggucguuaU..............    10     1    dv9
...........................................................auaagacuuuuuuggucguuaC..............    1      1    dv9
...........................................................auaagacuuuuuuggucguuagc.............    41     0    dv9
...........................................................auaagacuuuuuuggucguuGgc.............    1      1    dv9
...........................................................auaagacuuuuuuggucguuagU.............    1      1    dv9
...........................................................auaagacuuuuuuggucguuaUc.............    4      1    dv9
...........................................................auaagacuuuuuuggucguuCgc.............    1      1    dv9
...........................................................auaagacuuuuuuggucguuagA.............    2      1    dv9
...........................................................auaagacuuuuuuUgucguuagc.............    1      1    dv9
...........................................................auaagacuuuuuuggucguuagcC............    1      1    dv9
............................................................uaagacuuuuuuggucguua...............    1      0    dv9
............................................................uaagacuuuuuuggucguuCg..............    1      1    dv9
............................................................uaagacuuuuuuggucguuaC..............    1      1    dv9
............................................................uaagacuuuuuuggucguuaU..............    1      1    dv9
............................................................uaagacuuuuuuggucguuag..............    23     0    dv9
............................................................uaagGcuuuuuuggucguuag..............    1      1    dv9
............................................................uaagacuuuuuuUgucguuag..............    1      1    dv9
............................................................uaagacuuuuuuggucguuaUc.............    3      1    dv9
............................................................uaagacuuuuuuggucUuuagc.............    1      1    dv9
............................................................uaagacuuuuuuggCcguuagc.............    1      1    dv9
............................................................uaagacuuuuuuggucguuagA.............    2      1    dv9
............................................................uaagacuuuuuuggucguuCgc.............    1      1    dv9
............................................................uaagacuuuuuuggucguuaCc.............    1      1    dv9
............................................................uaagacuuuuuuggucguuagc.............    51     0    dv9
............................................................uaagacuuuuuuggucguuagcU............    3      1    dv9
............................................................uaagacuuuuuuggucguuagcC............    81     1    dv9
...............................................................gacuuuuuuggucguuagc.............    1      0    dv9

................ugagcgagcaaugaaguaauauu........................................................    4      0    arg
................ugagcgagcaaugaaguGauauu........................................................    1      1    arg
................ugagcgagcaaugaaguaauauuC.......................................................    2      1    arg
.................gagcgagcaaugaaguaauauu........................................................    22     0    arg
.................gagcgagcaaugaaguCauauuu.......................................................    1      1    arg
.................gagcgagcaGugaaguaauauuu.......................................................    1      1    arg
.................gagcgagcaaugaaguaauauuu.......................................................    66     0    arg
.................gagcCagcaaugaaguaauauuu.......................................................    2      1    arg
.................gagcgagcaaugaaguaauauuA.......................................................    1      1    arg
.................gagcgagcaaugaagAaauauuu.......................................................    1      1    arg
.................Aagcgagcaaugaaguaauauuu.......................................................    1      1    arg
.................gagcgagcaaugaaguaauauuC.......................................................    11     1    arg
.................gagcgagcaaugaaguaauauuug......................................................    1      0    arg
.................gagcgagcaaugaaguaauauuuC......................................................    6      1    arg
..................agcgagcaaugaaguaauauuu.......................................................    1      0    arg
...................gcgagcaaugaaguaauauuug......................................................    3      0    arg
...................gcgagcaaugaaguaauauuugu.....................................................    3      0    arg
.........................................................aaauaagacuuuuuuggucguu................    2      0    arg
.........................................................aaauaagacuuuuuuggucguua...............    1      0    arg
...........................................................auaagacuuuuuuggucguua...............    3      0    arg
...........................................................auaagacuuuuuuggucguuaU..............    3      1    arg
...........................................................auaagacuuuuuuggucguuag..............    35     0    arg
...........................................................auaagacuuuuuuggucguuagA.............    1      1    arg
...........................................................aCaagacuuuuuuggucguuagc.............    1      1    arg
...........................................................auaagacuuuuuuggucguuagc.............    62     0    arg
...........................................................auaagacuuuuuugCucguuagc.............    1      1    arg
...........................................................auaagacuuuuuugUucguuagc.............    1      1    arg
...........................................................auaagacuuuuuuggucguuagcC............    3      1    arg
............................................................uaagacuuuuuuggucUuuag..............    1      1    arg
............................................................uaagacuuuuuuggucguuag..............    12     0    arg
............................................................uaagacuuuuuugCucguuagc.............    1      1    arg
............................................................uaagacuuuuuuggucguuagc.............    71     0    arg
............................................................uaagacuuuuuuggucguuaUc.............    5      1    arg
............................................................uaagacuuuuuuggucUuuagc.............    1      1    arg
............................................................uaagacCuuuuuggucguuagc.............    1      1    arg
............................................................uaagacuuuuuuggucguuagA.............    5      1    arg
............................................................uaagacuuuuuuggucguuaCc.............    1      1    arg
............................................................uaagacuuuuuuggucguuagG.............    2      1    arg
```

```
                                                            dvi-miR-974-1-3p
                 dvi-miR-974-1-5p
augaggccaauuucucugagcgagcaaugaaguaauauuuguucgaccugauauaacaaauaagacuuuuuuggucguuagcaggaauuguccau

............................................................uaagacuuuuuuggucguuagcC............    39    1    arg
............................................................uaagacuuuuuuggucguuagcU............    2     1    arg
```

miRBase precursor : dvi-mir-974-2
Total read count : 12824
dvi-miR-974-2-5p read count9740
dvi-miR-974-2-3p read count3082
remaining reads : 2

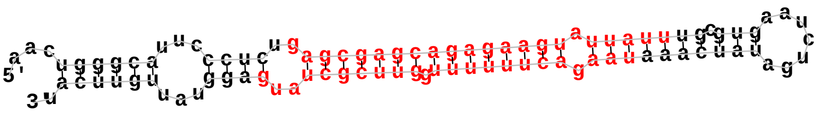

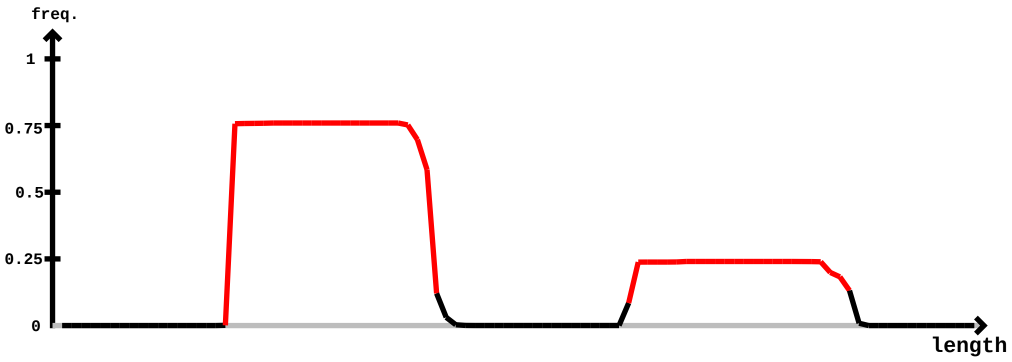

dvi-miR-974-2-3p

dvi-miR-974-2-5p

```
5'- aacugggcauucccucugagcgagcagagaaguauuauuugcgugaaucugauaucaaauaagacuuuuuugguucgcuaugagguauuguucau  -3'    exp
    ...((((((...((((..(((((((((((((((.(((((((.(((.......)))))))))).)))))))).)))))))..))))...)))))).     reads      mm         sample
    ................ugagcgagcagagaaguauu...........................................................     1          0          arg
    ................Cgagcgagcagagaaguauuau.........................................................     5          1          arg
    ................ugagcgagcagagaaguauuauu........................................................     1          0          arg
    ................Cgagcgagcagagaaguauuauu........................................................     2          1          arg
    .................gagcgagcagagaaguau............................................................     72         0          arg
    .................gagcgagcagagaagCau............................................................     1          1          arg
    .................gagcgGgcagagaaguau............................................................     1          1          arg
    .................gagcgagcagagaaguCu............................................................     7          1          arg
    .................gagcgagcagGgaaguau............................................................     1          1          arg
    .................gagcgagUagagaaguau............................................................     1          1          arg
    .................Aagcgagcagagaaguauu...........................................................     1          1          arg
    .................gagAgagcagagaaguauu...........................................................     1          1          arg
    .................gagUgagcagagaaguauu...........................................................     3          1          arg
    .................gagcgagcagagCaguauu...........................................................     1          1          arg
    .................gagcgagcagCgaaguauu...........................................................     1          1          arg
    .................gagcgagcagagaagAauu...........................................................     1          1          arg
    .................gagcgagcagagaaguaAu...........................................................     1          1          arg
    .................gagcgagcagagaaguaCu...........................................................     1          1          arg
    .................gagcgagcagagaaguaGu...........................................................     1          1          arg
    .................gagcgagcagagaaguUuu...........................................................     2          1          arg
    .................gagcAagcagagaaguauu...........................................................     1          1          arg
    .................gagcgagcagagaaguauC...........................................................     8          1          arg
    .................gagcgagcagagaaguauu...........................................................     522        0          arg
    .................gagcgagcagagaUguauu...........................................................     1          1          arg
    .................gagcgagcagagaGguauu...........................................................     1          1          arg
    .................gagcgagcagagaagCauu...........................................................     3          1          arg
    .................gagcgagcagagaaguGuu...........................................................     1          1          arg
    .................gagcgagcagagUaguauu...........................................................     1          1          arg
    .................gagcgagcagagGaguauu...........................................................     1          1          arg
    .................gagcgagcagagaagGauu...........................................................     4          1          arg
    .................gGgcgagcagagaaguauu...........................................................     1          1          arg
    .................gagcgagcagGgaaguauu...........................................................     1          1          arg
    .................gaAcgagcagagaaguauua..........................................................     1          1          arg
    .................gagcgagcagagUaguauua..........................................................     3          1          arg
```

```
                                                           dvi-miR-974-2-3p
                 dvi-miR-974-2-5p
aacugggcauucccucugagcgagcagagaaguauuauuugcgugaaucugauaucaaauaagacuuuuuugguucgcuaugagguauuguucau

.................gagAgagcagagaaguauua..........................................................    1       1       arg
.................gagcgagcagagaaguauua..........................................................    208     0       arg
.................gaCcgagcagagaaguauua..........................................................    1       1       arg
.................Aagcgagcagagaaguauua..........................................................    1       1       arg
.................gagcgagcagagaaguauuG..........................................................    1       1       arg
.................gagcgGgcagagaaguauua..........................................................    1       1       arg
.................gagcgagUagagaaguauua..........................................................    1       1       arg
.................gagcgCgcagagaaguauua..........................................................    1       1       arg
.................gagcgagcagagaaguUuua..........................................................    1       1       arg
.................gagcgagcagagaaguauuU..........................................................    40      1       arg
.................gagcgagcagagaaguauuC..........................................................    397     1       arg
.................gagcgagcagagaagCauua..........................................................    3       1       arg
.................gagcgagcagagaagAauua..........................................................    1       1       arg
.................gagcgagcagagaaguaGua..........................................................    1       1       arg
.................gagcgagcagagaaguauCau.........................................................    7       1       arg
.................gagcgagcagagaaguUuuau.........................................................    3       1       arg
.................gagAgagcagagaaguauuau.........................................................    3       1       arg
.................gagcgagUagagaaguauuau.........................................................    6       1       arg
.................gagcgagAagagaaguauuau.........................................................    1       1       arg
.................gagcgGgcagagaaguauuau.........................................................    7       1       arg
.................gaCcgagcagagaaguauuau.........................................................    3       1       arg
.................gagcgagcagagGaguauuau.........................................................    9       1       arg
.................gagcgagcagagaaguauGau.........................................................    2       1       arg
.................gagcgagcagGgaaguauuau.........................................................    9       1       arg
.................gagcgagcagagaGguauuau.........................................................    6       1       arg
.................gagcgagcCgagaaguauuau.........................................................    2       1       arg
.................gagcgagcagagaaguaCuau.........................................................    6       1       arg
.................gaAcgagcagagaaguauuau.........................................................    4       1       arg
.................gagcgCgcagagaaguauuau.........................................................    1       1       arg
.................Aagcgagcagagaaguauuau.........................................................    2       1       arg
.................gagcgagcagagaagGauuau.........................................................    19      1       arg
.................gagcgagcagagaaguauuCu.........................................................    5       1       arg
.................gagcgagcUgagaaguauuau.........................................................    2       1       arg
.................gagcgagcaUagaaguauuau.........................................................    1       1       arg
.................gagcgaUcagagaaguauuau.........................................................    2       1       arg
.................gagcgagcagUgaaguauuau.........................................................    1       1       arg
.................gagcgagcagagaagAauuau.........................................................    5       1       arg
.................gagUgagcagagaaguauuau.........................................................    11      1       arg
.................gagcgaAcagagaaguauuau.........................................................    1       1       arg
.................gagcgagcagagaaguauuaC.........................................................    4       1       arg
.................gagcgagcagagCaguauuau.........................................................    5       1       arg
.................gagcgagcagaUaaguauuau.........................................................    1       1       arg
.................gagcgaCcagagaaguauuau.........................................................    1       1       arg
.................gagcgagcagagaaguauuaG.........................................................    3       1       arg
.................Uagcgagcagagaaguauuau.........................................................    4       1       arg
.................gagcgagcagagaagCauuau.........................................................    16      1       arg
.................gagcgagcaCagaaguauuau.........................................................    1       1       arg
.................gagcgagcagCgaaguauuau.........................................................    1       1       arg
.................gagcgagcagagaaguauuGu.........................................................    3       1       arg
.................gagcgagcagagaaguCuuau.........................................................    1       1       arg
.................gUgcgagcagagaaguauuau.........................................................    1       1       arg
.................Cagcgagcagagaaguauuau.........................................................    2       1       arg
.................gagcgagcagagaUguauuau.........................................................    4       1       arg
.................gagcCagcagagaaguauuau.........................................................    1       1       arg
.................gagcgagcagagaaguGuuau.........................................................    7       1       arg
.................gagcUagcagagaaguauuau.........................................................    1       1       arg
.................gGgcgagcagagaaguauuau.........................................................    7       1       arg
.................gagcgUgcagagaaguauuau.........................................................    4       1       arg
.................gagcgagcagagaaguauuau.........................................................    3737    0       arg
.................gagcgagcagagaCguauuau.........................................................    5       1       arg
.................gagcgagcagagaaguauuUu.........................................................    1       1       arg
.................gagcgagcagagaaguaGuau.........................................................    1       1       arg
.................gagcgagcGgagaaguauuau.........................................................    9       1       arg
.................gagcgagcagagaaguauuaA.........................................................    19      1       arg
.................gagcgagGagagaaguauuau.........................................................    1       1       arg
.................gagcgagcagagaaAuauuau.........................................................    2       1       arg
.................gagcgagcagagUaguauuau.........................................................    13      1       arg
.................gagcAagcagagaaguauuau.........................................................    6       1       arg
.................gagcgagcagagaaguauuauC........................................................    265     1       arg
```

```
                 dvi-miR-974-2-5p                          dvi-miR-974-2-3p
aacugggcauucccucugagcgagcagagaaguauuauuugcgugaaucugauaucaaauaagacuuuuuugguucgcuaugagguauuguucau

.................gagcgagcagagaaguauuauu........................................................   127   0   arg
.................gagcgGgcagagaaguauuauuu.......................................................   1     1   arg
.................gagcgagcagagaaguauuauuC.......................................................   44    1   arg
.................gagGgagcagagaaguauuauuu.......................................................   1     1   arg
.................gagcgagcagagCaguauuauuu.......................................................   1     1   arg
.................gagcgagcagagaaguauuauCu.......................................................   3     1   arg
.................gagcgagcagagaagGauuauuu.......................................................   2     1   arg
.................gagcgagcagagaaguauuGuuu.......................................................   1     1   arg
.................gagcgagcagagaaguauuCuuu.......................................................   1     1   arg
.................gagcgagcagagaaguauuauuu.......................................................   139   0   arg
.................gagcgagcagagaaguCuuauuu.......................................................   1     1   arg
.................gagUgagcagagaaguauuauuu.......................................................   1     1   arg
.................gagcgagcagagaaguauuauuuC......................................................   12    1   arg
.................gagcgagcagagaaguauuauuuCc.....................................................   2     1   arg
..................agcgagcagagaaguauu...........................................................   1     0   arg
..................agcgagcagagaaguauuau.........................................................   4     0   arg
...................gcgagcagagaaguauuau.........................................................   2     0   arg
.....................gagcagagaaguauuauC........................................................   1     1   arg
..........................................................auaagacuuuuuugguucg..................   1     0   arg
..........................................................auaagacuuuuuugguucgc.................   1     0   arg
..........................................................auaagacuuuuuugguucUcu................   1     1   arg
..........................................................aCaagacuuuuuugguucgcu................   1     1   arg
..........................................................auaagacuuuuuugguucgcu................   93    0   arg
..........................................................auaagacuuuuuugguucgcua...............   13    0   arg
..........................................................auaagacuuuuuugguucgcuC...............   1     1   arg
..........................................................auaagacuuuuuugguucgcuU...............   2     1   arg
..........................................................auaagacuuuuuugguuUgcuau..............   1     1   arg
..........................................................auaagacuuuuuugguucgcuUu..............   1     1   arg
..........................................................aCaagacuuuuuugguucgcuau..............   1     1   arg
..........................................................auaagacuuuuuugguucgcuau..............   34    0   arg
..........................................................auaagacuuuuuugguucgcuaug.............   56    0   arg
..........................................................auaagacuuuuuugguucgcuaugU............   8     1   arg
...........................................................uaagacuuuuuugguucgcu................   22    0   arg
...........................................................uaagacuuuuuuAguucgcu................   1     1   arg
...........................................................uaagacuuuuuugguucgcua...............   8     0   arg
...........................................................uaagacuuuuuugguuAgcua...............   1     1   arg
...........................................................uaagacuuuuuugguucgcuU...............   1     1   arg
...........................................................uaagacuuuuuuUguucgcuau..............   1     1   arg
...........................................................uaagacuuuuuugguGcgcuau..............   1     1   arg
...........................................................uaagacuuuuuugguucgcuau..............   55    0   arg
...........................................................uaagacuuuuuugguucgcuauC.............   4     1   arg
...........................................................uaagacuuuuuugguucgcuaug.............   143   0   arg
...........................................................uaagacuuuuuugAuucgcuaug.............   1     1   arg
...........................................................uaagacuuuuuugguucgcuauU.............   5     1   arg
...........................................................uaagacuCuuuugguucgcuaug.............   1     1   arg
...........................................................uaagacuuuuuugguucgcuCug.............   1     1   arg
...........................................................uaGgacuuuuuugguucgcuaug.............   1     1   arg
...........................................................uaagacuuuuuugguucgcAaug.............   1     1   arg
...........................................................uaagacuuuuuugUuucgcuaug.............   1     1   arg
...........................................................uaagacuuuuuugguucgcuauA.............   1     1   arg
...........................................................uaagacuuuuuugguucgcuaugU............   33    1   arg
...........................................................uaagacuuuuuugguucgcuauga............   1     0   arg
............................................................Uagacuuuuuugguucgcuaug.............   1     1   arg
...............................................................acuuuuuugguucgcuaugU............   1     1   arg
................................................................cuuuuuugguucgcuaug.............   4     0   arg
................................................................cuuuuuugguucUcuaug.............   1     1   arg
................................................................cAuuuuugguucgcuaug.............   1     1   arg

................ugagcgagcagagaaguauuauC........................................................   1     1   dv9
.................gagcgagcagagaaguCu............................................................   1     1   dv9
.................gagcgagcagagaaguau............................................................   4     0   dv9
.................gagcgagcagagaaguauC...........................................................   5     1   dv9
.................gagcgagcagagCaguauu...........................................................   1     1   dv9
.................gGgcgagcagagaaguauu...........................................................   3     1   dv9
.................gagcgagcagagaaguauu...........................................................   71    0   dv9
.................Uagcgagcagagaaguauu...........................................................   4     1   dv9
.................gagcgagcagagaaguauua..........................................................   169   0   dv9
.................gagcgagcagagaGguauua..........................................................   1     1   dv9
.................gagcgagcagagaaguGuua..........................................................   1     1   dv9
```

dvi-miR-974-2-3p

dvi-miR-974-2-5p

aacugggcauucccucugagcgagcagagaaguauuauuugcgugaaucugauaucaaauaagacuuuuuugguucgcuaugagguauuguucau

.................Uagcgagcagagaaguauua.. 8 1 dv9
.................gagcgagcagagaaguauCa.. 1 1 dv9
.................Aagcgagcagagaaguauua.. 1 1 dv9
.................gagcgaUcagagaaguauua.. 2 1 dv9
.................gagcgagcagagaaguaCua.. 1 1 dv9
.................gagcgagcGgagaaguauua.. 1 1 dv9
.................gagcgagcagagaaguauuU.. 4 1 dv9
.................gagcgagcagagaagAauua.. 1 1 dv9
.................gagcgagcagagaaguauuC.. 235 1 dv9
.................gagcCagcagagaaguauua.. 2 1 dv9
.................gagUgagcagagaaguauua.. 1 1 dv9
.................gaCcgagcagagaaguauua.. 1 1 dv9
.................gagcgagcagagaaguCuua.. 1 1 dv9
.................gagcgagcagagaaguauuG.. 2 1 dv9
.................gagcgagcagagaCguauua.. 1 1 dv9
.................gagcgagcagagaCguauuau... 6 1 dv9
.................gagcgagcagagGaguauuau... 2 1 dv9
.................gagcgagcagagaaguauuCu... 10 1 dv9
.................Cagcgagcagagaaguauuau... 1 1 dv9
.................gagcgagcagagaagCauuau... 2 1 dv9
.................gagcgagcagagaaguauuau... 1081 0 dv9
.................gagcgaCcagagaaguauuau... 1 1 dv9
.................gagcgagcagagaaguauuaC... 6 1 dv9
.................gagcgagcagGgaaguauuau... 1 1 dv9
.................gagcgagcGgagaaguauuau... 2 1 dv9
.................gagcgagcaUagaaguauuau... 1 1 dv9
.................gagcgagcagCgaaguauuau... 3 1 dv9
.................gagcgagcagagaaCuauuau... 1 1 dv9
.................gagcgagcagagaaguauuaG... 1 1 dv9
.................Uagcgagcagagaaguauuau... 65 1 dv9
.................gagcgagcagagaaguCuuau... 8 1 dv9
.................gCgcgagcagagaaguauuau... 3 1 dv9
.................gagcgGgcagagaaguauuau... 3 1 dv9
.................gagcgagcagagCaguauuau... 10 1 dv9
.................gagcgagcagagaUguauuau... 1 1 dv9
.................gagcgagcagUgaaguauuau... 1 1 dv9
.................gagcgagcagagaGguauuau... 2 1 dv9
.................gagcgagcagagaaguGuuau... 1 1 dv9
.................gagcgagcagagaaguauuaA... 12 1 dv9
.................gagcgagUagagaaguauuau... 1 1 dv9
.................gagcgagcagagaaguaCuau... 1 1 dv9
.................Aagcgagcagagaaguauuau... 2 1 dv9
.................gagcAagcagagaaguauuau... 1 1 dv9
.................gagcgagcagagaagAauuau... 2 1 dv9
.................gagcgaUcagagaaguauuau... 1 1 dv9
.................gagcgagcagagaaguauuauu.. 37 0 dv9
.................gagcgagcagagaaguCuuauu.. 1 1 dv9
.................gagcgagcagagaaguauuaCu.. 1 1 dv9
.................gagcgagcagagaaguauuauC.. 408 1 dv9
.................gagcgagcagagaaguaCuauu.. 1 1 dv9
.................gagcgagcagagaaguauuauG.. 2 1 dv9
.................gagcgagcaUagaaguauuauu.. 1 1 dv9
.................Uagcgagcagagaaguauuauu.. 3 1 dv9
.................gagcgagcaUagaaguauuauuu... 1 1 dv9
.................gagcgagcagagaaguauuCuuu... 1 1 dv9
.................gagcgagcagagaaAuauuauuu... 1 1 dv9
.................Uagcgagcagagaaguauuauuu... 5 1 dv9
.................gagcgagcagagaaguGuuauuu... 1 1 dv9
.................gagcgagcagagaaguauuauuC... 31 1 dv9
.................gCgcgagcagagaaguauuauuu... 1 1 dv9
.................gagcgagcagagaaguauuauuu... 61 0 dv9
.................gagcgagcagagaaguauAauuu... 1 1 dv9
.................gagcgagcagagaaguauuauuuC.. 12 1 dv9
.................gagcgagcagagaaguauuauuuCc... 1 1 dv9
..................agcgagcagagaaguauuau... 3 0 dv9
..................agcgagcagagaaguauuauC.. 1 1 dv9
...................gcgagcagagaaguauuauu.. 1 0 dv9
...................gcgagcagagaaguauuauC.. 1 1 dv9
....................cgagcagagaaguauuau... 1 0 dv9

dvi-miR-974-2-3p

dvi-miR-974-2-5p

aacugggcauucccucugagcgagcagagaaguauuauuugcgugaaucugauaucaaauaagacuuuuuugguucgcuaugagguauuguucau

....................cgagcagagaaguauuauC..	1	1	dv9
....................cgagcagagaaguauuauuuCc...	1	1	dv9
.....................gagcagagaaguauuauC..	9	1	dv9
....ugcgugaaucugauaucaaa....................................	2	0	dv9
....auaagacuuuuuugguuc...................	1	0	dv9
....auaagacuuuuuugguucgc.................	3	0	dv9
....auaagacuuuuuugUuucgcu................	1	1	dv9
....auaagacuuuuuugguucUcu................	2	1	dv9
....auaagacuuuuuugguucgcA................	1	1	dv9
....auaagacCuuuuugguucgcu................	1	1	dv9
....auaagacuuuuuugguuUgcu................	1	1	dv9
....auaagacuuuuuugguucgcC................	1	1	dv9
....auaagacuuuuuugguucgcu................	125	0	dv9
....auaagacuuuuuugguucUcua...............	3	1	dv9
....auaagacuuuuuugguucgcuC...............	14	1	dv9
....auaagacuuuuuugguucgcua...............	8	0	dv9
....auaagacuuuuuugguucgcuU...............	2	1	dv9
....Cuaagacuuuuuugguucgcuau..............	2	1	dv9
....auaGgacuuuuuugguucgcuau..............	1	1	dv9
....auaagacuuuuuugguucUcuau..............	7	1	dv9
....auaagacuuuuuugguucgcuCu..............	4	1	dv9
....auaagacuuGuuugguucgcuau..............	1	1	dv9
....auaagacuuuuuugguucgcuau..............	101	0	dv9
....auaagacuuuuuuggAucgcuau..............	1	1	dv9
....auaagacuuuuuugUuucgcuaug.............	3	1	dv9
....auaagacuuuuuugguucgcuCug.............	4	1	dv9
....auaagacuuuuuuUguucgcuaug.............	3	1	dv9
....auaagacuuuuuugguucgcuaug.............	106	0	dv9
....auaagacuuuuuugguucgcGaug.............	1	1	dv9
....auaaUacuuuuuugguucgcuaug.............	1	1	dv9
....auaagacuuuuuugguucgcuauC.............	6	1	dv9
....auaagacuuuuuugguucgcuauU.............	2	1	dv9
....auaagacuuuuuugguucUcuaug.............	1	1	dv9
....auaagacuuuuuugguucgcuaGg.............	1	1	dv9
....auaagacCuuuuugguucgcuaug.............	1	1	dv9
....auaagacuuuuuugguucgcuaugU............	9	1	dv9
....uaagacuuuuuugguucgc.................	1	0	dv9
....uaagacuuuuuugguucUcu................	1	1	dv9
....uaagacuuuuuugUuucgcu................	2	1	dv9
....uaagacuuuuuugguucgcu................	52	0	dv9
....uaagacuuuuuugguucgcua...............	56	0	dv9
....uaagacuuuuCugguucgcua...............	1	1	dv9
....uaagacuuuuuugguucgcuC...............	10	1	dv9
....uaagacuuuuuCgguucgcua...............	1	1	dv9
....uaagacuuuuuugguuUgcua...............	1	1	dv9
....uaagacuuuuuugguucgcuau..............	169	0	dv9
....uaagacuuuuuugCuucgcuau..............	1	1	dv9
....uaagacuuuuCugguucgcuau..............	1	1	dv9
....uaagacuuuuuugguucgcuUu..............	1	1	dv9
....uaagacuuuuuugguucgcuaC..............	2	1	dv9
....uaagacuuuuuugUuucgcuau..............	1	1	dv9
....uaagacCuuuuugguucgcuau..............	1	1	dv9
....uaagacuuuuuugguucgcuCu..............	1	1	dv9
....uaagacuuuuuugguucgcuaG..............	1	1	dv9
....uaagacuuuuuugguucgcuaug.............	505	0	dv9
....uaagacuuuuuugCuucgcuaug.............	1	1	dv9
....uaagacuuuuuugguucgcuGug.............	1	1	dv9
....uaGgacuuuuuugguucgcuaug.............	1	1	dv9
....uaagacuuuuuugguucgcuCug.............	14	1	dv9
....uaagacuuuuuugguuAgcuaug.............	1	1	dv9
....uaagacuuuuuCgguucgcuaug.............	1	1	dv9
....uaagacuuuuuugguucgcuUug.............	3	1	dv9
....uaagacuuuuuugguucgcuauC.............	26	1	dv9
....uaagaAuuuuuugguucgcuaug.............	1	1	dv9
....uaagacGuuuuugguucgcuaug.............	1	1	dv9
....uaaUacuuuuuugguucgcuaug.............	1	1	dv9
....uaagacuuuuuuUguucgcuaug.............	3	1	dv9
....uaagacuuCuuugguucgcuaug.............	1	1	dv9
....uaagacuuuuuugguucUcuaug.............	7	1	dv9

dvi-miR-974-2-3p

dvi-miR-974-2-5p

aacugggcauucccucugagcgagcagagaaguauuauuugcgugaaucugauaucaaauaagacuuuuuugguucgcuaugagguauuguucau

....uaagacuuuuuugguucgcuauA.............	1	1	dv9
....uaagacuuuuuugUuucgcuaug.............	2	1	dv9
....uaagacuuuuuugguucgAuaug.............	1	1	dv9
....uaagacuuuuCugguucgcuaug.............	1	1	dv9
....uaagacuuuuuugguucgcuauU.............	12	1	dv9
....uaagacuuuuuugguucgcuaAg.............	1	1	dv9
....uaagacCuuuuugguucgcuaug.............	1	1	dv9
....uaagacuuuuuugguucCcuaug.............	1	1	dv9
....uaagaUuuuuuugguucgcuaug.............	1	1	dv9
....uaagacuuuuuugguucUcuauga............	1	1	dv9
....uaagacuuuuuugguucgcuauga............	1	0	dv9
....uaagacuuuuuugguucgcuaugU............	23	1	dv9
....acuuuuuugguucgcuaug.............	3	0	dv9
....acuuuuuugguucgcuauU.............	1	1	dv9
....Auuuuuugguucgcuaug.............	1	1	dv9
....cuuuuuugguucgcuauC.............	1	1	dv9
....cuuuuuugguucgcuaug.............	6	0	dv9
....cuuuuuugguucgcuaugU............	1	1	dv9
................Cgagcgagcagagaaguauu...	1	1	dv1
.................gagcgagcagagGaguau..	1	1	dv1
.................gagcgagcagagaaguau..	4	0	dv1
.................gagcgagcagagaaguCu..	1	1	dv1
.................gagcgaUcagagaaguauu...	1	1	dv1
.................gagcgagcUgagaaguauu...	2	1	dv1
.................gagcgagcagagaaguauu...	53	0	dv1
.................gagcgagcagagaaguauC...	3	1	dv1
.................Uagcgagcagagaaguauu...	3	1	dv1
.................gagcgagcagagaagCauua..	2	1	dv1
.................gagcgagcagagaaguauuU..	5	1	dv1
.................gagcgagcagagaaguCuua..	1	1	dv1
.................gagcgagcagagaaguauuC..	179	1	dv1
.................gagcgagcGgagaaguauua..	1	1	dv1
.................gagcgagcagagaaguauua..	142	0	dv1
.................gaCcgagcagagaaguauua..	1	1	dv1
.................gagcgagcagagaGguauua..	1	1	dv1
.................gagcgGgcagagaaguauua..	2	1	dv1
.................gagcgagcagagaCguauua..	2	1	dv1
.................Uagcgagcagagaaguauua..	6	1	dv1
.................gagcgagcagagaaguauCa..	2	1	dv1
.................gagcgagcagagaagCauuau...	1	1	dv1
.................gagcgagcagagCaguauuau...	7	1	dv1
.................gagcgagcagagaaguauuaC...	1	1	dv1
.................gagcgagcaUagaaguauuau...	2	1	dv1
.................gagcgagcagagaagGauuau...	1	1	dv1
.................gagcgagcagagaaguGuuau...	1	1	dv1
.................gagcgagcagagaaguauuaA...	7	1	dv1
.................gagcgagcagagaGguauuau...	1	1	dv1
.................gagcgagcagagaaguauuCu...	1	1	dv1
.................gagcgagcagagaaguUuuau...	1	1	dv1
.................gagcgagcGgagaaguauuau...	1	1	dv1
.................gagcgagcagagaaUuauuau...	1	1	dv1
.................gagcgagcagCgaaguauuau...	1	1	dv1
.................gagcgaUcagagaaguauuau...	1	1	dv1
.................gagcgUgcagagaaguauuau...	1	1	dv1
.................gagcgagcagagaaguauuau...	645	0	dv1
.................gagcgGgcagagaaguauuau...	1	1	dv1
.................gagcgagcagagaaguCuuau...	5	1	dv1
.................Uagcgagcagagaaguauuau...	33	1	dv1
.................gaUcgagcagagaaguauuau...	1	1	dv1
.................gagcgagcagagaCguauuau...	8	1	dv1
.................gagcgCgcagagaaguauuau...	2	1	dv1
.................gagcgagcagagaaguaGuau...	1	1	dv1
.................gagcgagcagGgaaguauuau...	1	1	dv1
.................Aagcgagcagagaaguauuau...	1	1	dv1
.................gagcgagcagagaaguauuaG...	1	1	dv1
.................gagcgagcagagaaguauuauu..	14	0	dv1
.................gagcgagcagagaaguauuauC..	266	1	dv1
.................gagcUagcagagaaguauuauu..	1	1	dv1

dvi-miR-974-2-3p

dvi-miR-974-2-5p

aacugggcauucccucugagcgagcagagaaguauuauuugcgugaaucugauaucaaauaagacuuuuuugguucgcuaugagguauuguucau

.................gagcgaCcagagaaguauuauu..	2	1	dv1
.................gagcgagcagagUaguauuauuu...	1	1	dv1
.................Uagcgagcagagaaguauuauuu...	1	1	dv1
.................gagUgagcagagaaguauuauuu...	2	1	dv1
.................gCgcgagcagagaaguauuauuu...	1	1	dv1
.................gagcgagcagagaaguauuauuu...	39	0	dv1
.................gagcgagcagagaaguauuauuC...	15	1	dv1
.................gagcgagcagagaaguauuauuuC..	8	1	dv1
..................agcgagcagagaaguauuau...	1	0	dv1
...................gcgagcagagaaguauuC..	1	1	dv1
....................cgagcagagaaguauuauC..	3	1	dv1
.....................gagcagagaaguauuauC..	2	1	dv1
....Cauaagacuuuuuugguucgcuau..............	1	1	dv1
....auaagacuuuuuugguuc...................	2	0	dv1
....auaagacuuuuuugguucg..................	1	0	dv1
....auaagacuuuuuugguucgc.................	3	0	dv1
....auaagacuuuuuugguucgcC................	1	1	dv1
....auaagacuuuuuugguucUcu................	4	1	dv1
....auaagacuuuuuuggCucgcu................	1	1	dv1
....auaagacuuuuuugUuucgcu................	2	1	dv1
....auaagacuuCuuugguucgcu................	1	1	dv1
....auaagacuuuuuuUguucgcu................	2	1	dv1
....aGaagacuuuuuugguucgcu................	1	1	dv1
....auaagacuuuuuugguucgcu................	143	0	dv1
....auaagacCuuuuugguucgcu................	1	1	dv1
....auaagacuuuuuugguucgcuC...............	12	1	dv1
....auaagacuuuuuugguucgcua...............	22	0	dv1
....auaagacuuuuuugguucgcuU...............	6	1	dv1
....auaagacuuuuuugguucgcuCu..............	3	1	dv1
....auaagacuuuuuugguucUcuau..............	3	1	dv1
....auaagacuuuuuugguucgcuau..............	114	0	dv1
....auaagacuuuuuugguucCcuau..............	1	1	dv1
....auaagacuCuuuugguucgcuau..............	1	1	dv1
....auaagacuuuuuuUguucgcuau..............	1	1	dv1
....auaagacuuuuuugguucgcuUu..............	1	1	dv1
....auaagCcuuuuuugguucgcuau..............	1	1	dv1
....auaagacuuuuuugguCcgcuau..............	1	1	dv1
....auaagaAuuuuuugguucgcuau..............	1	1	dv1
....auaagCcuuuuuugguucgcuaug.............	1	1	dv1
....auaagacuuuuuGgguucgcuaug.............	1	1	dv1
....auaagacuuuuuugUuucgcuaug.............	4	1	dv1
....auaagacuuuuuugguucgcuCug.............	3	1	dv1
....auaagacuuuuuugguucgcuauA.............	1	1	dv1
....auaagacuuuuuugguucUcuaug.............	1	1	dv1
....auaagacuuuuuugguucgcuauU.............	5	1	dv1
....auaagacuuuuuugguucgcAaug.............	1	1	dv1
....auaagacuuuuuugCuucgcuaug.............	2	1	dv1
....auaagacuuuuuuUguucgcuaug.............	1	1	dv1
....auaagacuuuuuugguucgcuaug.............	91	0	dv1
....auaagacuuuuuugguucgcuauC.............	4	1	dv1
....auaagacuuuuuugguucCcuaug.............	1	1	dv1
....auaagacuuuuuugguucgcuaugU............	4	1	dv1
....uaagacuuuuuugguucgc.................	1	0	dv1
....uaagacuuuuuugguucgcC................	1	1	dv1
....uaagacuuuuuugguucgcu................	52	0	dv1
....uaagacuuuuuugguucgcA................	1	1	dv1
....uaagacuuuuuugguucgcua...............	39	0	dv1
....uaagacuuuuuugguucUcua...............	1	1	dv1
....uaagacuuuuuugguucgcuC...............	9	1	dv1
....uaagacuuuuuugguucUcuau..............	2	1	dv1
....uaagacuuuuuugUuucgcuau..............	2	1	dv1
....uaagacuuuuuugguucgcuau..............	132	0	dv1
....Caagacuuuuuugguucgcuau..............	1	1	dv1
....uaagacuuuuuuggCucgcuau..............	1	1	dv1
....uaagacuuuuuugguucgcuCu..............	1	1	dv1
....uaagacuuuuuugguucgcuaA..............	1	1	dv1
....uaagacuuuuuugguucgcuUu..............	2	1	dv1
....uaagacuuuuuugguucgcuaug.............	426	0	dv1
....uaagacuuCuuugguucgcuaug.............	2	1	dv1

```
                                                           dvi-miR-974-2-3p
                 dvi-miR-974-2-5p
aacugggcauucccucugagcgagcagagaaguauuauuugcgugaaucugauaucaaauaagacuuuuuugguucgcuaugagguauuguucau

...........................................................uaagacuuuuuuggGucgcuaug.............    1    1    dv1
...........................................................uaagacuuuuuuAguucgcuaug.............    1    1    dv1
...........................................................Gaagacuuuuuugguucgcuaug.............    1    1    dv1
...........................................................uaGgacuuuuuugguucgcuaug.............    1    1    dv1
...........................................................uaagacuuuuuugguucgcGaug.............    1    1    dv1
...........................................................uaagacuuuuuugguuAgcuaug.............    2    1    dv1
...........................................................uaagacuuuuuuggCucgcuaug.............    2    1    dv1
...........................................................uaagacuuuuuugguucCcuaug.............    1    1    dv1
...........................................................uaagacuuuuuuUguucgcuaug.............    1    1    dv1
...........................................................uaagacuuuuuugguuUgcuaug.............    1    1    dv1
...........................................................uaagacuuuuuugguucgcuauU.............    11   1    dv1
...........................................................Caagacuuuuuugguucgcuaug.............    2    1    dv1
...........................................................uaagacuuuuuugguucgcuCug.............    9    1    dv1
...........................................................uaagacuuuuuugguucgcuUug.............    1    1    dv1
...........................................................uaagacuuuuuugguucgcuauC.............    22   1    dv1
...........................................................uaagacuuuuuugUuucgcuaug.............    2    1    dv1
...........................................................uaagacuuuuuugguucUcuaug.............    7    1    dv1
...........................................................uaagacuuuuuugguucgcuGug.............    2    1    dv1
...........................................................uaagacuuuuuugguucgcuauga............    2    0    dv1
...........................................................uaagacuuuuuugguucgcuaugU............    26   1    dv1
............................................................aagacuuuuuugguucgcuaug.............    1    0    dv1
...............................................................acuuuuuugguucgcuaug.............    1    0    dv1
................................................................Auuuuuugguucgcuaug.............    1    1    dv1
................................................................cuuuuuugguucgcuaug.............    5    0    dv1
................................................................cuuuuuugguucgcuaugU............    1    1    dv1
.................................................................uuuuuugguucgcuaugU............    1    1    dv1
```

miRBase precursor : dvi-mir-9692
Total read count : 21332
dvi-miR-9692-5p read count : 9380
dvi-miR-9692-3p read count : 11929
remaining reads : 23

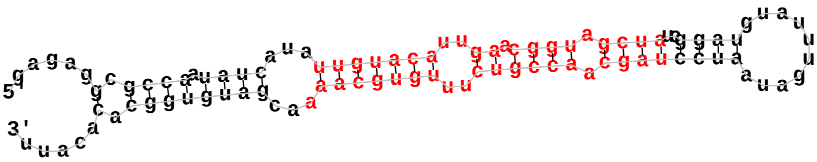

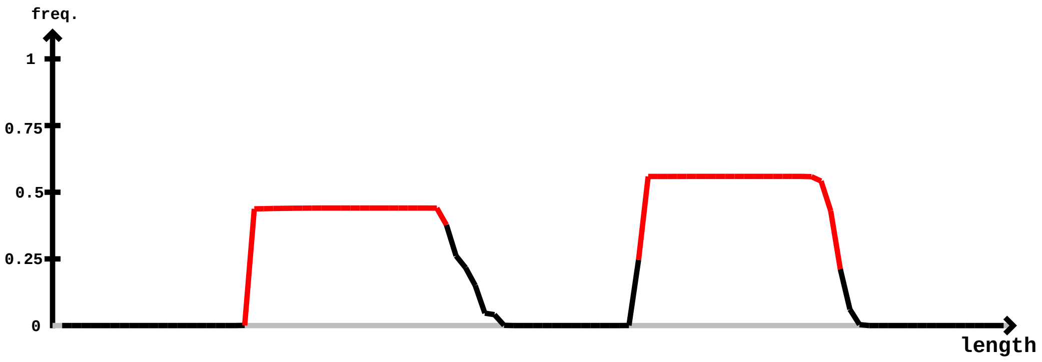


```
                    dvi-miR-9692-5p
                                                    dvi-miR-9692-3p
5'- gagaggcgccaauaucauauuguacauugaacgguagcuaucggauguauuugauaauccuagcaaccgucuuugugcaaaacgauguggcacacauu   -3'    exp
    .....(.((((.((((...(((((((..((.((((.((((..((((..........)))))))).))))))..)))))))...)))))))).).....    reads      mm         sample
    .................uauuguacauugaacgguagcu...........................................................    1          0          dv9
    ..................Cuuguacauugaacgguagcua..........................................................    8          1          dv9
    ..................Cuuguacauugaacgguagcuauc........................................................    1          1          dv9
    ..................Cuuguacauugaacgguagcuaucg.......................................................    2          1          dv9
    ..................Cuuguacauugaacgguagcuaucgga.....................................................    1          1          dv9
    ...................uuguacauugaacgguagc............................................................    2          0          dv9
    ...................uuguacaCugaacgguagcu...........................................................    1          1          dv9
    ...................uuguacauugaaAgguagcu...........................................................    1          1          dv9
    ...................Guguacauugaacgguagcu...........................................................    1          1          dv9
    ...................uuguacauugaacgUuagcu...........................................................    1          1          dv9
    ...................Cuguacauugaacgguagcu...........................................................    1          1          dv9
    ...................uuCuacauugaacgguagcu...........................................................    1          1          dv9
    ...................uuguacauugaacgguaUcu...........................................................    1          1          dv9
    ...................uuguacauugaacgAuagcu...........................................................    1          1          dv9
    ...................uuguacauugaacgguagcC...........................................................    12         1          dv9
    ...................uuguacauugaacgCuagcu...........................................................    1          1          dv9
    ...................uuguacauugaacgguCgcu...........................................................    2          1          dv9
    ...................uuguacauugaacgguagcu...........................................................    502        0          dv9
    ...................uugGacauugaacgguagcu...........................................................    1          1          dv9
    ...................uuguacauuUaacgguagcu...........................................................    1          1          dv9
    ...................uuguacauugaacgguGgcu...........................................................    3          1          dv9
    ...................uugAacauugaacgguagcu...........................................................    1          1          dv9
    ...................uuguacauugCacgguagcu...........................................................    1          1          dv9
    ...................uuguacauugaacUguagcu...........................................................    1          1          dv9
    ...................uuguacauugaacggCagcua..........................................................    1          1          dv9
    ...................uuguacauugaacgguagcuC..........................................................    105        1          dv9
    ...................Cuguacauugaacgguagcua..........................................................    1          1          dv9
    ...................uuguacauugaacgguCgcua..........................................................    1          1          dv9
    ...................uuguacauugaCcgguagcua..........................................................    2          1          dv9
    ...................Auguacauugaacgguagcua..........................................................    1          1          dv9
    ...................uuguacauugaacgguagcuG..........................................................    1          1          dv9
    ...................uuguacauugaacgguGgcua..........................................................    1          1          dv9
    ...................uuUuacauugaacgguagcua..........................................................    3          1          dv9
    ...................uuCuacauugaacgguagcua..........................................................    2          1          dv9
```

```
                   dvi-miR-9692-5p
                                                          dvi-miR-9692-3p
gagaggcgccaauaucauauuguacauugaacgguagcuaucggauguauuugauaauccuagcaaccgucuuugugcaaaacgauguggcacacauu

...................uuguacauugaacgguagcua..........................................................   653   0   dv9
...................uuguacauugaacgCuagcua..........................................................   1     1   dv9
...................uuguacauugaaUgguagcua..........................................................   1     1   dv9
...................uuguacauugaacUguagcua..........................................................   1     1   dv9
...................uuguacauugaacCguagcua..........................................................   1     1   dv9
...................uCguacauugaacgguagcua..........................................................   1     1   dv9
...................uuguacauugaacgguagcuU..........................................................   25    1   dv9
...................uugCacauugaacgguagcua..........................................................   2     1   dv9
...................uuguacaCugaacgguagcua..........................................................   1     1   dv9
...................uuguacauugGacgguagcua..........................................................   3     1   dv9
...................uuguacauugaacAguagcuau.........................................................   1     1   dv9
...................uuguacauCgaacgguagcuau.........................................................   1     1   dv9
...................uCguacauugaacgguagcuau.........................................................   1     1   dv9
...................uuguacauugaacgguagcuCu.........................................................   16    1   dv9
...................uuguacauugaacgguagcuau.........................................................   496   0   dv9
...................uuguacauugaacgguagcuUu.........................................................   5     1   dv9
...................uuguacauugaaAgguagcuau.........................................................   2     1   dv9
...................uuUuacauugaacgguagcuau.........................................................   1     1   dv9
...................uuguacauugaacCguagcuau.........................................................   1     1   dv9
...................uuguacauugaacgguagcuaC.........................................................   2     1   dv9
...................uuguacauuUaacgguagcuau.........................................................   1     1   dv9
...................uuguacauugaacgguagcuaA.........................................................   1     1   dv9
...................uuguacauugaacgguagcuaG.........................................................   1     1   dv9
...................uuguacauugaacgguagcuGu.........................................................   1     1   dv9
...................uuguacGuugaacgguagcuau.........................................................   2     1   dv9
...................uuguacauugaacgguCgcuau.........................................................   2     1   dv9
...................uuguacaCugaacgguagcuau.........................................................   1     1   dv9
...................uugGacauugaacgguagcuau.........................................................   1     1   dv9
...................uuguacauugaaAgguagcuauc........................................................   1     1   dv9
...................uuguacauugaacgguaCcuauc........................................................   1     1   dv9
...................uuguacauugaacgguagcuaAc........................................................   1     1   dv9
...................uuguacauugaacgguCgcuauc........................................................   6     1   dv9
...................uuguacauugaacgguagcuUuc........................................................   1     1   dv9
...................uuguacauugaaUgguagcuauc........................................................   1     1   dv9
...................uuguacauugaacUguagcuauc........................................................   1     1   dv9
...................uuguacCuugaacgguagcuauc........................................................   1     1   dv9
...................uuguacauugaacgguagcuauU........................................................   7     1   dv9
...................uuguacauugaacgguagcuCuc........................................................   18    1   dv9
...................uuCuacauugaacgguagcuauc........................................................   2     1   dv9
...................uuguacaCugaacgguagcuauc........................................................   2     1   dv9
...................uuguacauugaacgAuagcuauc........................................................   2     1   dv9
...................uuguacauugaacgguagcuauA........................................................   4     1   dv9
...................Cuguacauugaacgguagcuauc........................................................   2     1   dv9
...................uuguacauuCaacgguagcuauc........................................................   1     1   dv9
...................uuguacauugaacgguagcuaCc........................................................   1     1   dv9
...................uuguacauugaacCguagcuauc........................................................   1     1   dv9
...................uuguacauCgaacgguagcuauc........................................................   2     1   dv9
...................uCguacauugaacgguagcuauc........................................................   1     1   dv9
...................uuguacauugaacgUuagcuauc........................................................   2     1   dv9
...................uuguacauugaacgguagcuauc........................................................   768   0   dv9
...................uuguacauugaacggCagcuaucg.......................................................   1     1   dv9
...................uuguacauugaacgguagcuaucA.......................................................   12    1   dv9
...................Cuguacauugaacgguagcuaucg.......................................................   2     1   dv9
...................uuguacGuugaacgguagcuaucg.......................................................   2     1   dv9
...................uuguacauugaacgguagcuCucg.......................................................   4     1   dv9
...................uuguacauugaacgguCgcuaucg.......................................................   4     1   dv9
...................uuguacauugaacgguagcuaucC.......................................................   728   1   dv9
...................uuguacauugaacgguagcuauUg.......................................................   2     1   dv9
...................uuguacauugaacgguagcuGucg.......................................................   1     1   dv9
...................uuguacauugaacgguagcuaAcg.......................................................   1     1   dv9
...................uuguacauugaacgguagcuaucg.......................................................   408   0   dv9
...................uuguacauugaacgguagUuaucg.......................................................   1     1   dv9
...................uugGacauugaacgguagcuaucg.......................................................   1     1   dv9
...................uuguacauugGacgguagcuaucg.......................................................   1     1   dv9
...................uuguacauugaacgguagcuaCcg.......................................................   2     1   dv9
...................uuguacauugaacUguagcuaucg.......................................................   2     1   dv9
...................uuguacauugaacgguagcuaucU.......................................................   89    1   dv9
...................uGguacauugaacgguagcuaucg.......................................................   1     1   dv9
...................uuguacauugaaAgguagcuaucg.......................................................   1     1   dv9
```

```
                   dvi-miR-9692-5p
                                                            dvi-miR-9692-3p
gagaggcgccaauaucauauuguacauugaacgguagcuaucggauguauuugauaauccuagcaaccgucuuugugcaaaacgauguggcacacauu

...................uuguacauCgaacgguagcuaucg.......................................................  2    1    dv9
...................uuguacauugaaUgguagcuaucg.......................................................  1    1    dv9
...................uuUuacauugaacgguagcuaucg.......................................................  2    1    dv9
...................uuguacauugaacgguagcuaucgC......................................................  2    1    dv9
...................uuguacauugaacgguagcuaucgA......................................................  6    1    dv9
...................uuguacauugaacgguaCcuaucgg......................................................  1    1    dv9
...................uuguacauugaacgguagcuaucAg......................................................  5    1    dv9
...................uuguacauugaacgguagcuaucUg......................................................  5    1    dv9
...................uuguacauugaacgguagcuGucgg......................................................  1    1    dv9
...................uuguacauugaacgguagcuUucgg......................................................  1    1    dv9
...................uuguacauugaacgguaUcuaucgg......................................................  2    1    dv9
...................uuguacauugaacgguagcuaucgg......................................................  45   0    dv9
...................uuguacauugaacggGagcuaucgg......................................................  1    1    dv9
...................uuguacauugaacgguagcuaucgU......................................................  3    1    dv9
...................uuguacauugaacAguagcuaucgga.....................................................  1    1    dv9
...................uuguacauugaCcgguagcuaucgga.....................................................  1    1    dv9
...................uuguaAauugaacgguagcuaucgga.....................................................  1    1    dv9
...................uuguacauugaacgguagcuaucggG.....................................................  2    1    dv9
...................Cuguacauugaacgguagcuaucgga.....................................................  2    1    dv9
...................uuguacauugaacgguagcuaAcgga.....................................................  3    1    dv9
...................uugCacauugaacgguagcuaucgga.....................................................  1    1    dv9
...................uuguacauuAaacgguagcuaucgga.....................................................  2    1    dv9
...................uuguacauugaacgguagcuaCcgga.....................................................  1    1    dv9
...................uuguacauugaacgguagcuaucggC.....................................................  22   1    dv9
...................uuguacauugaacgguagcuCucgga.....................................................  8    1    dv9
...................uuguacauugaacgguagcuaucggU.....................................................  57   1    dv9
...................uuguacauugaacgguaAcuaucgga.....................................................  1    1    dv9
...................uuguacauugaacgguagcuaucgUa.....................................................  8    1    dv9
...................uuguacauugaacgguagcuaucgga.....................................................  324  0    dv9
...................uuguacauugaUcgguagcuaucgga.....................................................  1    1    dv9
...................uuguacauuUaacgguagcuaucgga.....................................................  1    1    dv9
...................uuguacauugaacgguagcuaucUga.....................................................  13   1    dv9
...................uuguacauugaacgUuagcuaucgga.....................................................  4    1    dv9
...................uuguacauugaacgguCgcuaucgga.....................................................  3    1    dv9
...................uuguacauugaacggCagcuaucgga.....................................................  1    1    dv9
...................uuguacauugaacgguagcuaucggaC....................................................  2    1    dv9
...................uuguacauugaacgguagcuaucggaA....................................................  3    1    dv9
....................uguacauugaacgguagcu...........................................................  1    0    dv9
....................uguacauugaacgguagcua..........................................................  1    0    dv9
....................uUuacauugaacgguagcuauc........................................................  1    1    dv9
....................uUuacauugaacgguagcuaucg.......................................................  1    1    dv9
....................uguacauugaacgguagcuaucggC.....................................................  1    1    dv9
....................uUuacauugaacgguagcuaucgga.....................................................  1    1    dv9
.....................guacauugaacgguagcu...........................................................  2    0    dv9
.....................Cuacauugaacgguagcua..........................................................  1    1    dv9
.....................guacauugaacgguagcuau.........................................................  1    0    dv9
.....................Uuacauugaacgguagcuauc........................................................  5    1    dv9
.....................guacauugaacgguagcuaucC.......................................................  3    1    dv9
......................uacauugaacgguagcua..........................................................  1    0    dv9
......................uacauugaacgguagcuUu.........................................................  1    1    dv9
......................uacauugaacgguagcuauc........................................................  1    0    dv9
......................uacauugaacgguagcuaucgga.....................................................  2    0    dv9
.......................acauugaacgguagcuau.........................................................  1    0    dv9
.......................Ccauugaacgguagcuaucg.......................................................  1    1    dv9
.......................acauugaacgguagcuaucg.......................................................  3    0    dv9
.......................acauugaacgguagcuaucgga.....................................................  2    0    dv9
.........................auugaacgguagcuaucC.......................................................  2    1    dv9
.........................auugaacgguagcuaucg.......................................................  1    0    dv9
..........................uugaacgguagcuaucgga.....................................................  3    0    dv9
............................gaacgguagcuaucggaA....................................................  1    1    dv9
.......................................................Gauccuagcaaccgucuuugugcaa..................  1    1    dv9
..........................................................Ucuagcaaccgucuuugugcaa..................  1    1    dv9
..........................................................Ucuagcaaccgucuuugugcaaa.................  1    1    dv9
...........................................................cuagcaaccgucuuugug.....................  6    0    dv9
...........................................................Auagcaaccgucuuugugc....................  10   1    dv9
...........................................................cuagUaaccgucuuugugc....................  2    1    dv9
...........................................................cGagcaaccgucuuugugc....................  1    1    dv9
...........................................................cuagcaaccgucuuugCgc....................  1    1    dv9
...........................................................cuagcaaccgucuuuguCc....................  2    1    dv9
```

```
                   dvi-miR-9692-5p
                                                            dvi-miR-9692-3p
gagaggcgccaauaucauauuguacauugaacgguagcuaucggauguauuugauaauccuagcaaccgucuuugugcaaaacgauguggcacacauu

...........................................................cuagcaaccguAuuugugc....................   1          1          dv9
...........................................................cuagcaaccgucuuugugc....................   165        0          dv9
...........................................................cuagcaaccguUuuugugc....................   1          1          dv9
...........................................................cuagcaaccgucuuCgugca...................   3          1          dv9
...........................................................cuagcaaccgucuuuUugca...................   2          1          dv9
...........................................................cGagcaaccgucuuugugca...................   1          1          dv9
...........................................................cuaAcaaccgucuuugugca...................   1          1          dv9
...........................................................cuagcaaccguUuuugugca...................   1          1          dv9
...........................................................cuCgcaaccgucuuugugca...................   1          1          dv9
...........................................................cuagcaaccgucuCugugca...................   1          1          dv9
...........................................................cuagcaaccgucuuugugcC...................   49         1          dv9
...........................................................cuagcaaccUucuuugugca...................   2          1          dv9
...........................................................cuagAaaccgucuuugugca...................   2          1          dv9
...........................................................cuagcaaccgucuuugugcG...................   1          1          dv9
...........................................................cuaUcaaccgucuuugugca...................   1          1          dv9
...........................................................cuGgcaaccgucuuugugca...................   1          1          dv9
...........................................................cuagcaaccgCcuuugugca...................   2          1          dv9
...........................................................cuagcaaccgucuuugugca...................   663        0          dv9
...........................................................Auagcaaccgucuuugugca...................   35         1          dv9
...........................................................cuaCcaaccgucuuugugca...................   3          1          dv9
...........................................................cuagcCaccgucuuugugca...................   2          1          dv9
...........................................................cuagcaaccgucuuugCgca...................   1          1          dv9
...........................................................cuagcaaccgucuuugAgca...................   1          1          dv9
...........................................................cuagcaCccgucuuugugca...................   1          1          dv9
...........................................................Uuagcaaccgucuuugugca...................   3          1          dv9
...........................................................cuagcaaccgucuuuguUca...................   1          1          dv9
...........................................................cuagcaaccgucuuugugUa...................   3          1          dv9
...........................................................cuagcaaccgGcuuugugcaa..................   1          1          dv9
...........................................................cuagcaaccgucuuugugcaC..................   50         1          dv9
...........................................................Guagcaaccgucuuugugcaa..................   2          1          dv9
...........................................................cuagcaaAcgucuuugugcaa..................   2          1          dv9
...........................................................cuagcaaccgucCuugugcaa..................   4          1          dv9
...........................................................Auagcaaccgucuuugugcaa..................   51         1          dv9
...........................................................cuagcaaccgucuCugugcaa..................   1          1          dv9
...........................................................cuagcaacUgucuuugugcaa..................   1          1          dv9
...........................................................cuagcaaccgucuuuUugcaa..................   2          1          dv9
...........................................................cuagcGaccgucuuugugcaa..................   2          1          dv9
...........................................................cuaCcaaccgucuuugugcaa..................   1          1          dv9
...........................................................cuagcaaccgucuuugugcCa..................   21         1          dv9
...........................................................cuagcaaccUucuuugugcaa..................   1          1          dv9
...........................................................cuagcaaccgucuuugugcaG..................   7          1          dv9
...........................................................cuagcaGccgucuuugugcaa..................   3          1          dv9
...........................................................cuagcaaccgucuuugugcGa..................   2          1          dv9
...........................................................cuagcaaUcgucuuugugcaa..................   1          1          dv9
...........................................................cuagcaaccgucuuugugcaa..................   1207       0          dv9
...........................................................cuagcaaccgucuuugugcaU..................   3          1          dv9
...........................................................cuagcaaccgucuuugugAaa..................   1          1          dv9
...........................................................cuagcaaccgucuuuguUcaa..................   1          1          dv9
...........................................................cuagUaaccgucuuugugcaa..................   1          1          dv9
...........................................................cuCgcaaccgucuuugugcaa..................   1          1          dv9
...........................................................cuagcaaccgCcuuugugcaa..................   3          1          dv9
...........................................................cuagcaUccgucuuugugcaa..................   2          1          dv9
...........................................................cuagcaaccgucuuAgugcaa..................   1          1          dv9
...........................................................cuagcaaccgucuuugCgcaa..................   3          1          dv9
...........................................................cuagcaaccgucCuugugcaaa.................   1          1          dv9
...........................................................cuagcaaccCucuuugugcaaa.................   1          1          dv9
...........................................................cuaUcaaccgucuuugugcaaa.................   1          1          dv9
...........................................................Auagcaaccgucuuugugcaaa.................   16         1          dv9
...........................................................cuagcaacUgucuuugugcaaa.................   1          1          dv9
...........................................................cuagcaaccgucuuugugcaaC.................   12         1          dv9
...........................................................cuagcaGccgucuuugugcaaa.................   1          1          dv9
...........................................................cuagcaaccgucuuugugcaaa.................   320        0          dv9
...........................................................cuagcaaccgucuuugugcCaa.................   8          1          dv9
...........................................................cuagcaaccgucuuugCgcaaa.................   2          1          dv9
...........................................................cuagcaaccgucuuugugcaaU.................   3          1          dv9
...........................................................cuagcaaccgucuuugugcaUa.................   1          1          dv9
...........................................................cuagcaaccgucuuugugUaaa.................   1          1          dv9
...........................................................cuagcaaccgucuuugugcaCa.................   19         1          dv9
...........................................................cuagcaaccgucuuugugcaaaU................   2          1          dv9
```

dvi-miR-9692-5p

dvi-miR-9692-3p

gagaggcgccaauaucauauuguacauugaacgguagcuaucggauguauuugauaauccuagcaaccgucuuugugcaaaacgauguggcacacauu			
....cuagcaaccgucuuugugcaaCa................	9	1	dv9
....cuagcaaccgucuuugugcaaUa................	1	1	dv9
....cuagcaaccgucuuugugcaCaa................	6	1	dv9
....cuagcaaccgucuuugCgcaaaa................	2	1	dv9
....cuagcaaccgucCuugugcaaaa................	1	1	dv9
....cuagcaaccgucuuugugcaaaC................	1	1	dv9
....cuagcaaccgucuuugugcCaaa................	1	1	dv9
....cGagcaaccgucuuugugcaaaa................	1	1	dv9
....cuagcaaccgucuuugugcaaaa................	121	0	dv9
....Auagcaaccgucuuugugcaaaa................	6	1	dv9
....cuagcaaccgucuuugugcaaaaA...............	2	1	dv9
....cuagcaaccgucuuugugcaaaaU...............	9	1	dv9
....uagcaaccgucuuugugc....................	22	0	dv9
....uagcaaccgucuuuguCc....................	1	1	dv9
....uagcaaccgucuuugCgca...................	2	1	dv9
....uagcaaccgucuuugugcG...................	4	1	dv9
....uagcaGccgucuuugugca...................	2	1	dv9
....uagcaaccgucCuugugca...................	3	1	dv9
....uagcaaccgucuuugAgca...................	1	1	dv9
....uagcaaccgCcuuugugca...................	2	1	dv9
....uaUcaaccgucuuugugca...................	1	1	dv9
....uagUaaccgucuuugugca...................	1	1	dv9
....uagcaaccgucuuuCugca...................	1	1	dv9
....uagcaaccgucuuuguAca...................	1	1	dv9
....uagcGaccgucuuugugca...................	2	1	dv9
....uagcaaccgucuuugugca...................	399	0	dv9
....Gagcaaccgucuuugugca...................	1	1	dv9
....uagcaaccgucuuugugcC...................	36	1	dv9
....uagcaaccgucuCugugca...................	1	1	dv9
....uCgcaaccgucuuugugca...................	1	1	dv9
....uagcaaccgucuuugugcaG..................	1	1	dv9
....uagcaaccgucuuugugcCa..................	21	1	dv9
....uagcaaccgucuuuguUcaa..................	2	1	dv9
....uagcaaccgucuuugGgcaa..................	3	1	dv9
....uagcaacUgucuuugugcaa..................	1	1	dv9
....uagcaaccgucuCugugcaa..................	2	1	dv9
....uagcaaccgucuuugugcaa..................	1063	0	dv9
....uagUaaccgucuuugugcaa..................	1	1	dv9
....uagcaGccgucuuugugcaa..................	2	1	dv9
....uagcaaccgucuuuguCcaa..................	1	1	dv9
....uagcaaccgucuuuUugcaa..................	1	1	dv9
....uagcaaccgucGuugugcaa..................	2	1	dv9
....uagcaaccgucuuGgugcaa..................	1	1	dv9
....uagcaaccgCcuuugugcaa..................	1	1	dv9
....uUgcaaccgucuuugugcaa..................	1	1	dv9
....Gagcaaccgucuuugugcaa..................	1	1	dv9
....uagcGaccgucuuugugcaa..................	2	1	dv9
....uagcaaccgucuuuCugcaa..................	1	1	dv9
....uagcaaccgucuuugugAaa..................	1	1	dv9
....uagcaaccgucuuugugcGa..................	2	1	dv9
....uagcaaccgucuuugugUaa..................	2	1	dv9
....uagcaaccgucuAugugcaa..................	2	1	dv9
....uagcaaccgucuuugugcaC..................	32	1	dv9
....uagcaaccgucuuugugcUa..................	1	1	dv9
....uagcaaUcgucuuugugcaa..................	1	1	dv9
....uaUcaaccgucuuugugcaa..................	5	1	dv9
....uagcaaccgucuuugugcaU..................	3	1	dv9
....uagcaaccgucuuuguAcaa..................	2	1	dv9
....uagcaaccguUuuugugcaa..................	1	1	dv9
....uagcaaccgucCuugugcaa..................	4	1	dv9
....uagcaaccgucuuugugGaa..................	1	1	dv9
....uagcaaccgucuCugugcaaa.................	2	1	dv9
....uagcaacUgucuuugugcaaa.................	1	1	dv9
....uagcaacAgucuuugugcaaa.................	1	1	dv9
....uagcaaccgucuuugCgcaaa.................	1	1	dv9
....uagcaaAcgucuuugugcaaa.................	1	1	dv9
....uagcaGccgucuuugugcaaa.................	3	1	dv9
....uaCcaaccgucuuugugcaaa.................	1	1	dv9
....uagcaaccgucuuugugcaaC.................	39	1	dv9

```
                   dvi-miR-9692-5p
                                                            dvi-miR-9692-3p
gagaggcgccaauaucauauuguacauugaacgguagcuaucggauguauuugauaauccuagcaaccgucuuugugcaaaacgauguggcacacauu

............................................................uagcaaccgucuuugugcCaa..................   17     1     dv9
............................................................uagcaaUcgucuuugugcaaa.................   1      1     dv9
............................................................uagcaaccgucuuugugcaaU.................   3      1     dv9
............................................................uagcaaccgucuuugugcaaG.................   3      1     dv9
............................................................uagcaaccgCcuuugugcaaa.................   1      1     dv9
............................................................uagcGaccgucuuugugcaaa.................   1      1     dv9
............................................................uagcaaccgucuuuguUcaaa.................   2      1     dv9
............................................................uagcaaccgucuuugugcaGa.................   1      1     dv9
............................................................uagcaaccgucuuugugcaaa.................   1029   0     dv9
............................................................uagcaaccgucuuugGgcaaa.................   1      1     dv9
............................................................uagcaCccgucuuugugcaaa.................   2      1     dv9
............................................................uCgcaaccgucuuugugcaaa.................   2      1     dv9
............................................................uagcaaccgucuuugugcaCa.................   47     1     dv9
............................................................uaUcaaccgucuuugugcaaa.................   2      1     dv9
............................................................uagcaaccgucuuuCugcaaa.................   5      1     dv9
............................................................uagUaaccgucuuugugcaaa.................   2      1     dv9
............................................................uagcaaccgucuuuguCcaaa.................   2      1     dv9
............................................................uagcaaccgucuuCgugcaaa.................   1      1     dv9
............................................................uagcCaccgucuuugugcaaa.................   1      1     dv9
............................................................uGgcaaccgucuuugugcaaa.................   2      1     dv9
............................................................uagcaaccgucuuugugUaaa.................   1      1     dv9
............................................................uagcaaccgucuuuUugcaaa.................   1      1     dv9
............................................................uagAaaccgucuuugugcaaa.................   1      1     dv9
............................................................Cagcaaccgucuuugugcaaa.................   1      1     dv9
............................................................uagUaaccgucuuugugcaaaa................   1      1     dv9
............................................................uagcGaccgucuuugugcaaaa................   2      1     dv9
............................................................Aagcaaccgucuuugugcaaaa................   1      1     dv9
............................................................uagcaaccgucuuugugcCaaa................   8      1     dv9
............................................................uagcaaAcgucuuugugcaaaa................   1      1     dv9
............................................................uagcaaccgucuuugugcaaGa................   2      1     dv9
............................................................uagcaaccgucCuugugcaaaa................   1      1     dv9
............................................................uagcaaccgucuuugugcaaaa................   473    0     dv9
............................................................uagcaaccgucuuugugcGaaa................   1      1     dv9
............................................................uagcaaccgucuuugugcaaaC................   12     1     dv9
............................................................uagcaaccgucuuuCugcaaaa................   1      1     dv9
............................................................uagcaaccgucuuugugcaaaU................   1      1     dv9
............................................................uagcaaccgucuuugugcaaCa................   19     1     dv9
............................................................uagcaaccgucuuugCgcaaaa................   1      1     dv9
............................................................uagcaaccUucuuugugcaaaa................   1      1     dv9
............................................................uagcaaccgucuuuguUcaaaa................   1      1     dv9
............................................................Cagcaaccgucuuugugcaaaa................   1      1     dv9
............................................................uagcaaccgucuuugugcaCaa................   32     1     dv9
............................................................uagcaaccgucuuugugcUaaa................   1      1     dv9
............................................................uagcaGccgucuuugugcaaaa................   1      1     dv9
............................................................uagcaaccgucuuugugcaaaaU...............   1      1     dv9
............................................................uagcaaccgucuuugugcaaaaA...............   24     1     dv9
............................................................uagcaaccgucuuugugcaaaUc...............   2      1     dv9
...............................................................caaccgucuuugugcaaa.................   1      0     dv9
...............................................................caaccgucuuugugcCaa.................   1      1     dv9
................................................................aaccgucuuugugcaaaaU...............   1      1     dv9

...................uuguacauugaacgguagc............................................................   3      0     arg
...................uuguacauugaacgAuagcu...........................................................   2      1     arg
...................uuguacauugaacgguagcu...........................................................   147    0     arg
...................uuguacauugaacgguagcC...........................................................   1      1     arg
...................uuguacauCgaacgguagcu...........................................................   1      1     arg
...................uugCacauugaacgguagcu...........................................................   1      1     arg
...................uuguacauugaacgguaUcu...........................................................   1      1     arg
...................uGguacauugaacgguagcu...........................................................   1      1     arg
...................uuguGcauugaacgguagcua..........................................................   1      1     arg
...................uuguacauugaacgguagcua..........................................................   272    0     arg
...................uuguacauugaacCguagcua..........................................................   1      1     arg
...................uuguacauugaacgguagcuU..........................................................   8      1     arg
...................uuguacauugGacgguagcua..........................................................   1      1     arg
...................uuguacauugaacgguagcuC..........................................................   7      1     arg
...................uuUuacauugaacgguagcua..........................................................   1      1     arg
...................uuguacauugaaAgguagcua..........................................................   1      1     arg
...................uuguacauugaacggCagcua..........................................................   2      1     arg
...................uuguacauugaacgguagcuau.........................................................   37     0     arg
```

dvi-miR-9692-5p

dvi-miR-9692-3p

gagaggcgccaauaucauauuguacauugaacgguagcuaucggauguauuugauaauccuagcaaccgucuuugugcaaaacgauguggcacacauu

...................uuguacauugaacgguagcuCu...	1	1	arg
...................uuguacauugaacggCagcuau...	1	1	arg
...................uuguacaCugaacgguagcuau...	1	1	arg
...................uuguacauugaacgguagcuaA...	2	1	arg
...................uuguacauugaacgguagcuGuc..	1	1	arg
...................uuguacauugaacgguagcuauc..	78	0	arg
...................uuguacauugaacgguagcuUuc..	1	1	arg
...................uuguacauugaacgguagcuauU..	1	1	arg
...................Cuguacauugaacgguagcuauc..	1	1	arg
...................uuCuacauugaacgguagcuauc..	1	1	arg
...................uuguacauugaacgguagcCaucg...	1	1	arg
...................uuguacauugaacgguagcuaucC...	27	1	arg
...................uuguacauugaacggGagcuaucg...	1	1	arg
...................uuguacauugaacgguagcuaucg...	81	0	arg
...................uuguacGuugaacgguagcuaucg...	1	1	arg
...................uuguacauugaacgguagcuaucU...	16	1	arg
...................uuguacauugaacgguagcuGucgg..	1	1	arg
...................uuguacauugaacgguagcuaucgg..	1	0	arg
...................uuguacauugaacgguagcuaucgU..	1	1	arg
...................uuguacauugaacgguagcuauUgga...	1	1	arg
...................uuguacauugaacgguagcuaucggU...	12	1	arg
...................uuguacauugaacgguagcuaucgga...	50	0	arg
...................uuguacauugaacgguagcuaucgUa...	2	1	arg
...................uuguacauugaacAguagcuaucgga...	1	1	arg
...................uuguacauugaacgguagcuaucggUu..	2	1	arg
....................uUuacauugaacgguagcuau...	1	1	arg
......................uacauugaacgguagcuaucggaA..	1	1	arg
.......................acauugaacgguagcuaucg...	1	0	arg
.........................auugaacgguagcuaucgga...	2	0	arg
....cuagcaaccgucuuugug.....................	3	0	arg
....cuagcaaccgucuuuguCc....................	1	1	arg
....cuagcaaccgucuuugugc....................	11	0	arg
....cuagcaaccgucuuugugcU...................	1	1	arg
....cGagcaaccgucuuugugca...................	1	1	arg
....cuagcaaccgucuuugugcC...................	3	1	arg
....cuagcaaccgucuuugCgca...................	1	1	arg
....cuagcaaccgucuuugugca...................	68	0	arg
....cuagcaaccguUuuugugca...................	1	1	arg
....cuagcaaccgucuuugugcaC..................	1	1	arg
....cuagcaaccgucuuugugcaa..................	33	0	arg
....cuagcaaccgucuuugugcaaG.................	1	1	arg
....cuagcaaccgucuuGgugcaaa.................	1	1	arg
....cuagcGaccgucuuugugcaaa.................	1	1	arg
....cuagcaaccgCcuuugugcaaa.................	1	1	arg
....cuagcaaccgucuuugugUaaa.................	1	1	arg
....cuagcaaccgucuuugugcaaa.................	97	0	arg
....cuagcaaccguUuuugugcaaaa................	1	1	arg
....cuagcaaccgucuuugugcaaaa................	19	0	arg
....cuagcaaccgucuuugugcaaaaA...............	1	1	arg
....uagcaaccgucuuugugc....................	4	0	arg
....uagcaaccgucuuugugcC...................	2	1	arg
....uagcaaccgucuuugCgca...................	1	1	arg
....uagcaaccgucuuuCugca...................	1	1	arg
....uagcaaccgucuuugugca...................	31	0	arg
....uagcaaccgucuuugugcaC..................	1	1	arg
....uagcaaccgucuuugugcaa..................	44	0	arg
....uagcaaccgucuuuAugcaa..................	1	1	arg
....uagcaaccgucuuugugcCa..................	1	1	arg
....uagcaaccgucuuugugcaGa.................	2	1	arg
....uagcaaccgucuuugugcaaa.................	205	0	arg
....uagcaaccgucuuuguCcaaa.................	2	1	arg
....uagcaacAgucuuugugcaaa.................	1	1	arg
....uagcaaccgucuuugugCaa..................	1	1	arg
....uagcaaccgucuuugugcaaaa................	42	0	arg
....uagcaaccgucuuugugcaaaaA...............	1	1	arg

...................uuguacauugaacgguag...	1	0	dv1
...................uuguacauugaacgguagc..	2	0	dv1
...................uCguacauugaacgguagcu...	1	1	dv1

```
                   dvi-miR-9692-5p
                                                           dvi-miR-9692-3p
gagaggcgccaauaucauauuguacauugaacgguagcuaucggauguauuugauaauccuagcaaccgucuuugugcaaaacgauguggcacacauu

...................uuguacauugCacgguagcu...........................................................  1     1   dv1
...................uuguacauugaacggCagcu...........................................................  2     1   dv1
...................uuguacauugaacgguUgcu...........................................................  1     1   dv1
...................uuguacauugaacgguCgcu...........................................................  6     1   dv1
...................uuguacGuugaacgguagcu...........................................................  1     1   dv1
...................uuguacauugaacgguGgcu...........................................................  1     1   dv1
...................uuguaAauugaacgguagcu...........................................................  1     1   dv1
...................uuguacauugaacgAuagcu...........................................................  1     1   dv1
...................uAguacauugaacgguagcu...........................................................  1     1   dv1
...................uugCacauugaacgguagcu...........................................................  1     1   dv1
...................uuguacauugaacAguagcu...........................................................  2     1   dv1
...................uuguacauugaUcgguagcu...........................................................  1     1   dv1
...................uuguaUauugaacgguagcu...........................................................  1     1   dv1
...................uuguacauugaaUgguagcu...........................................................  1     1   dv1
...................Cuguacauugaacgguagcu...........................................................  1     1   dv1
...................uuguacauCgaacgguagcu...........................................................  2     1   dv1
...................uuguacauugaacggAagcu...........................................................  1     1   dv1
...................uuUuacauugaacgguagcu...........................................................  1     1   dv1
...................uuguacauugaacUguagcu...........................................................  4     1   dv1
...................uuguacauugaacgguagcG...........................................................  1     1   dv1
...................uuguacauugaacgguagcu...........................................................  607   0   dv1
...................uuguacauugaacCguagcu...........................................................  1     1   dv1
...................uuguacauugaacgguagcC...........................................................  8     1   dv1
...................uuguacauugaacgguaUcu...........................................................  1     1   dv1
...................uuguacauugaGcgguagcu...........................................................  2     1   dv1
...................uuguacauugaacgUuagcu...........................................................  1     1   dv1
...................uuguacauugaGcgguagcua..........................................................  2     1   dv1
...................uuguacauCgaacgguagcua..........................................................  2     1   dv1
...................uuguacauugaCcgguagcua..........................................................  1     1   dv1
...................uuguacauugGacgguagcua..........................................................  3     1   dv1
...................uuguacauugaacgguagcua..........................................................  1182  0   dv1
...................uuguacauugaacCguagcua..........................................................  1     1   dv1
...................uuguacauugaaUgguagcua..........................................................  1     1   dv1
...................uuguacauugaacgguagcuU..........................................................  32    1   dv1
...................uuguacauuCaacgguagcua..........................................................  1     1   dv1
...................uuguacauugaacgguaUcua..........................................................  3     1   dv1
...................uuguGcauugaacgguagcua..........................................................  2     1   dv1
...................uuguacauugaacgguCgcua..........................................................  9     1   dv1
...................uuguacauuUaacgguagcua..........................................................  1     1   dv1
...................uuUuacauugaacgguagcua..........................................................  5     1   dv1
...................uGguacauugaacgguagcua..........................................................  1     1   dv1
...................uuguaUauugaacgguagcua..........................................................  1     1   dv1
...................uuguacauugaacgguagcuG..........................................................  2     1   dv1
...................uuguacauugaacgguagUua..........................................................  1     1   dv1
...................uuguacauugaacggCagcua..........................................................  3     1   dv1
...................uuguacauugaacgUuagcua..........................................................  5     1   dv1
...................uuguacGuugaacgguagcua..........................................................  1     1   dv1
...................uuCuacauugaacgguagcua..........................................................  3     1   dv1
...................uuguacauugaacgCuagcua..........................................................  1     1   dv1
...................uuguacauugaacUguagcua..........................................................  1     1   dv1
...................uuguacauugCacgguagcua..........................................................  1     1   dv1
...................uCguacauugaacgguagcua..........................................................  3     1   dv1
...................uuguacauugaacgguagcuC..........................................................  85    1   dv1
...................uuguacauugaacgguagcCa..........................................................  1     1   dv1
...................uuguacaCugaacgguagcua..........................................................  1     1   dv1
...................Guguacauugaacgguagcua..........................................................  1     1   dv1
...................uuguacauugaacUguagcuau.........................................................  1     1   dv1
...................uuguacauugaacgguagcuaA.........................................................  4     1   dv1
...................uuguacauugaacgguagcuaC.........................................................  2     1   dv1
...................uuguacauugaGcgguagcuau.........................................................  1     1   dv1
...................uuguacauugaacgguGgcuau.........................................................  1     1   dv1
...................uuguacauugaacggCagcuau.........................................................  1     1   dv1
...................uuguacauugaacgUuagcuau.........................................................  1     1   dv1
...................uuguacauugaacgguagcuau.........................................................  348   0   dv1
...................uuguacauugaacgguaUcuau.........................................................  1     1   dv1
...................Cuguacauugaacgguagcuau.........................................................  1     1   dv1
...................Guguacauugaacgguagcuau.........................................................  2     1   dv1
...................uuguacauugGacgguagcuau.........................................................  1     1   dv1
...................uuguacauugCacgguagcuau.........................................................  1     1   dv1
```

```
                   dvi-miR-9692-5p
                                                            dvi-miR-9692-3p
gagaggcgccaauaucauauuguacauugaacgguagcuaucggauguauuugauaauccuagcaaccgucuuugugcaaaacgauguggcacacauu

...................uuguacauugaacgguagcuUu.........................................................   1     1   dv1
...................uuguacauugaacgguagcuCu.........................................................   9     1   dv1
...................uuguacauugaacggCagcuauc........................................................   1     1   dv1
...................uuguacauugaacgUuagcuauc........................................................   1     1   dv1
...................uuguacauugaacUguagcuauc........................................................   2     1   dv1
...................uuguacauugaaAgguagcuauc........................................................   1     1   dv1
...................uuguacauugaacgAuagcuauc........................................................   1     1   dv1
...................uuguacauugaacgguagcuauA........................................................   1     1   dv1
...................uuguacauCgaacgguagcuauc........................................................   1     1   dv1
...................uuguacauuAaacgguagcuauc........................................................   1     1   dv1
...................uuguacauugaacgguagcuauU........................................................   12    1   dv1
...................uuguacauugaacgguaUcuauc........................................................   1     1   dv1
...................Cuguacauugaacgguagcuauc........................................................   2     1   dv1
...................uuguacauugaacgguagcuCuc........................................................   4     1   dv1
...................uuUuacauugaacgguagcuauc........................................................   1     1   dv1
...................uuguacauugaaUgguagcuauc........................................................   1     1   dv1
...................uuguacauugaacgguagcuauc........................................................   455   0   dv1
...................uuguacauugaacgguagcuaCc........................................................   1     1   dv1
...................uuguacauugaacgguGgcuauc........................................................   2     1   dv1
...................uuguacauugaacgguCgcuauc........................................................   1     1   dv1
...................uuguacauugaacggCagcuaucg.......................................................   1     1   dv1
...................uuguacauugaacgUuagcuaucg.......................................................   1     1   dv1
...................uuguacauugaacggAagcuaucg.......................................................   1     1   dv1
...................uuguacauugaacgguagcuauUg.......................................................   1     1   dv1
...................Guguacauugaacgguagcuaucg.......................................................   2     1   dv1
...................uuguacauugaacgguagUuaucg.......................................................   1     1   dv1
...................uuguacauugaacgguagcuaucA.......................................................   8     1   dv1
...................uuCuacauugaacgguagcuaucg.......................................................   2     1   dv1
...................uuguacauugaacgguUgcuaucg.......................................................   1     1   dv1
...................uugCacauugaacgguagcuaucg.......................................................   1     1   dv1
...................uuguacauugaacgguagcuGucg.......................................................   1     1   dv1
...................uuguacauugaacgguagcuaucC.......................................................   490   1   dv1
...................uuguacauugaacgguagcuaucU.......................................................   40    1   dv1
...................uuUuacauugaacgguagcuaucg.......................................................   2     1   dv1
...................uuguacauugaacgguagcuaucg.......................................................   241   0   dv1
...................uuguacauugaacgguGgcuaucg.......................................................   1     1   dv1
...................uuguacauugaGcgguagcuaucg.......................................................   2     1   dv1
...................uuguacauugaacgguagcuCucg.......................................................   2     1   dv1
...................uuguacauugaacgguagcuCucgg......................................................   1     1   dv1
...................uuguacauugaacgguagcuauUgg......................................................   1     1   dv1
...................uuguacauugaacgguagcuaucgC......................................................   2     1   dv1
...................uuguacauugaacgguagcuaucgg......................................................   35    0   dv1
...................uuguacauugaacgguagcuaucgU......................................................   1     1   dv1
...................Cuguacauugaacgguagcuaucgg......................................................   1     1   dv1
...................uuguacauugaacgguagcuUucgg......................................................   1     1   dv1
...................uuguacauugaacgguagcuaucAg......................................................   1     1   dv1
...................uuguacauugaacgguaAcuaucgga.....................................................   1     1   dv1
...................uuguacauugaacgguagcuaucggC.....................................................   15    1   dv1
...................uuguacauugaacgguagcuaucgUa.....................................................   5     1   dv1
...................uCguacauugaacgguagcuaucgga.....................................................   2     1   dv1
...................uuguacauugaacgguagcuaucggU.....................................................   22    1   dv1
...................uuguacauCgaacgguagcuaucgga.....................................................   1     1   dv1
...................uuguacauugaacgguagcuUucgga.....................................................   1     1   dv1
...................uuguacauugaacUguagcuaucgga.....................................................   1     1   dv1
...................uuguacauugaacgguagcuGucgga.....................................................   1     1   dv1
...................uuguacauugaGcgguagcuaucgga.....................................................   1     1   dv1
...................uuguacauugaacgguCgcuaucgga.....................................................   3     1   dv1
...................uuguacGuugaacgguagcuaucgga.....................................................   1     1   dv1
...................uuguacauugaacgguagcuCucgga.....................................................   6     1   dv1
...................uuguacauugaacgCuagcuaucgga.....................................................   1     1   dv1
...................uuguacauugaacgguagcuauUgga.....................................................   1     1   dv1
...................uuguacauugaacgUuagcuaucgga.....................................................   1     1   dv1
...................uuguacauugaacgguagcuaucUga.....................................................   11    1   dv1
...................uuguacauugaacgguagcuaucgga.....................................................   240   0   dv1
...................uuUuacauugaacgguagcuaucgga.....................................................   1     1   dv1
...................uuguacauugaacgguagcuaucggaC....................................................   3     1   dv1
...................uuguacauugaacgguagcuaucggau....................................................   2     0   dv1
...................uuguacauugaacgguagcuaucggaA....................................................   7     1   dv1
....................uUuacauugaacgguagcu...........................................................   1     1   dv1
```

dvi-miR-9692-5p

dvi-miR-9692-3p

gagaggcgccaauaucauauuguacauugaacgguagcuaucggauguauuugauaauccuagcaaccgucuuugugcaaaacgauguggcacacauu

....................uguacauugaacgguagcuC..	1	1	dv1
....................uguacauugaacgguagcuauc..	1	0	dv1
....................uUuacauugaacgguagcuauc..	1	1	dv1
....................uguacauugaacgguagcuaucgU..	1	1	dv1
.....................Uuacauugaacgguagcua..	3	1	dv1
.....................Uuacauugaacgguagcuaucg...	1	1	dv1
.....................guacauugaacgguagcuaucC...	1	1	dv1
.....................guacauugaacgguagcuaucggUu..	1	1	dv1
......................uacauugaacgguagcuau...	1	0	dv1
......................uacauugaacgguagcuauc..	1	0	dv1
......................uacauugaacgguagcuaucg...	1	0	dv1
.......................acauugaacgguagcuau...	2	0	dv1
.......................acauugaacgguagcuaucC...	1	1	dv1
.......................acauugaacgguagcuaucg...	1	0	dv1
........................cauugaacgguagcuauc..	3	0	dv1
........................cauugaacgguagcuaucg...	2	0	dv1
........................Aauugaacgguagcuaucg...	1	1	dv1
....ccuagcaaccgucuuugugcaa..................	2	0	dv1
....Ucuagcaaccgucuuugugcaaaa................	1	1	dv1
....cuagcaaccgucuuugug.....................	10	0	dv1
....Auagcaaccgucuuugug.....................	1	1	dv1
....Auagcaaccgucuuugugc....................	3	1	dv1
....cuagcaaccgucuuugugc....................	112	0	dv1
....cCagcaaccgucuuugugc....................	1	1	dv1
....cuagcaaccgucuuuguUc....................	1	1	dv1
....cuagcaaccgucCuugugc....................	1	1	dv1
....cuagcaaccgCcuuugugc....................	1	1	dv1
....cuagcaaccgucuuugCgc....................	1	1	dv1
....cuagcUaccgucuuugugc....................	1	1	dv1
....cuagcaacAgucuuugugc....................	1	1	dv1
....cuagcaaccgucuuugAgca...................	1	1	dv1
....Guagcaaccgucuuugugca...................	1	1	dv1
....cuagUaaccgucuuugugca...................	1	1	dv1
....cuagcaaccgucuuugugAa...................	2	1	dv1
....cuagcCaccgucuuugugca...................	1	1	dv1
....cuagcaaccgucuuugugcC...................	36	1	dv1
....cuagcaaccgCcuuugugca...................	2	1	dv1
....Auagcaaccgucuuugugca...................	26	1	dv1
....cuagcaaccgGcuuugugca...................	1	1	dv1
....cuaUcaaccgucuuugugca...................	1	1	dv1
....cuagcaaccgucuGugugca...................	1	1	dv1
....cuagcaaccgucuuuUugca...................	1	1	dv1
....cuagcaaccgucCuugugca...................	3	1	dv1
....cuagcGaccgucuuugugca...................	2	1	dv1
....cuagcaaccgucuuuguUca...................	2	1	dv1
....cuGgcaaccgucuuugugca...................	1	1	dv1
....cuagcaaccgucuuugugca...................	517	0	dv1
....cuaCcaaccgucuuugugcaa..................	2	1	dv1
....cuagcaGccgucuuugugcaa..................	1	1	dv1
....cuagcaaccUucuuugugcaa..................	1	1	dv1
....cuagcaaccgucuuugCgcaa..................	1	1	dv1
....cuagcaaccgucuuugugcaa..................	857	0	dv1
....cuagcaaccgucuCugugcaa..................	1	1	dv1
....cuaUcaaccgucuuugugcaa..................	3	1	dv1
....cuagcaaccgucuuugugcaC..................	27	1	dv1
....cuagcaaccgucuuugugUaa..................	1	1	dv1
....cuagcaaccgucuuuUugcaa..................	2	1	dv1
....cuagcaaccgucCuugugcaa..................	3	1	dv1
....cuCgcaaccgucuuugugcaa..................	1	1	dv1
....cuagcaaAcgucuuugugcaa..................	1	1	dv1
....cuagcaaccgucuuugugAaa..................	1	1	dv1
....cuagcaaccguUuuugugcaa..................	2	1	dv1
....cuagcaaccgucuuuguCcaa..................	1	1	dv1
....Auagcaaccgucuuugugcaa..................	30	1	dv1
....cuagcaaccgucuuugugcaG..................	2	1	dv1
....cCagcaaccgucuuugugcaa..................	1	1	dv1
....cuagcaaccgucuuuguUcaa..................	11	1	dv1
....cuagcaaccgCcuuugugcaa..................	1	1	dv1
....cuagcaaccgucuuuCugcaa..................	3	1	dv1

```
                   dvi-miR-9692-5p
                                                           dvi-miR-9692-3p
gagaggcgccaauaucauauuguacauugaacgguagcuaucggauguauuugauaaucccuagcaaccgucuuugugcaaaacgauguggcacacauu

..........................................................cuagcaaUcgucuuugugcaa...................   1      1      dv1
..........................................................cuagcaaccgucuuugugcGa...................   1      1      dv1
..........................................................cuagcaaccgucuuugugcCa...................   13     1      dv1
..........................................................cuagcaaccgucuuCgugcaa...................   4      1      dv1
..........................................................cuagcCaccgucuuugugcaa...................   2      1      dv1
..........................................................Guagcaaccgucuuugugcaa...................   1      1      dv1
..........................................................cuagcaaccgucuuugugcCaa..................   4      1      dv1
..........................................................cuagcaaccgucuuuAugcaaa..................   1      1      dv1
..........................................................cuagcaaccgucuuuCugcaaa..................   1      1      dv1
..........................................................cuagcaaccgucuuugugcaaG..................   1      1      dv1
..........................................................cuagcaaccgucuuugugcaaa..................   258    0      dv1
..........................................................cuagcaaccgucuuugugcaUa..................   1      1      dv1
..........................................................cuagcaaccgucuuuUugcaaa..................   3      1      dv1
..........................................................cuagcaaccgucuuugugcaaC..................   13     1      dv1
..........................................................cuagcaaccgucuuugCgcaaa..................   1      1      dv1
..........................................................cGagcaaccgucuuugugcaaa..................   1      1      dv1
..........................................................cuagcCaccgucuuugugcaaa..................   1      1      dv1
..........................................................cuagcaaccgucuuugugcaaU..................   1      1      dv1
..........................................................cuagcaaccgucuuugugcaCa..................   10     1      dv1
..........................................................cuagcaaccgucuuugugcUaa..................   1      1      dv1
..........................................................cuCgcaaccgucuuugugcaaa..................   1      1      dv1
..........................................................cuagcaaccgucGuugugcaaa..................   1      1      dv1
..........................................................Auagcaaccgucuuugugcaaa..................   11     1      dv1
..........................................................Uuagcaaccgucuuugugcaaa..................   1      1      dv1
..........................................................cuagcaaccAucuuugugcaaaa.................   1      1      dv1
..........................................................cuagcaaccgucuuugugcaaaG.................   3      1      dv1
..........................................................cuagcaaccgucuuugugcaaaU.................   1      1      dv1
..........................................................cuagcaaccgucuuuguCcaaaa.................   1      1      dv1
..........................................................cuagcaaccgucuuugugcaaaC.................   2      1      dv1
..........................................................cuagcaaccgucuuugugcCaaa.................   1      1      dv1
..........................................................Auagcaaccgucuuugugcaaaa.................   1      1      dv1
..........................................................cuagcaaccgucuuugugcaaaa.................   57     0      dv1
..........................................................cuagcaaccgucuuuAugcaaaa.................   2      1      dv1
..........................................................cuGgcaaccgucuuugugcaaaa.................   1      1      dv1
..........................................................cuagcaaccgucuuugugcaaCa.................   7      1      dv1
..........................................................cuagcaaccgucuuugugcaCaa.................   3      1      dv1
..........................................................cuagcaaccgucuuugugcaaaaU................   8      1      dv1
..........................................................cuagcaaccgucuuugugcaaaac................   1      0      dv1
..........................................................cuagcaaccgucuuugugcaaaaA................   3      1      dv1
...........................................................uagcaaccAucuuugugc.....................   1      1      dv1
...........................................................uagcaaccgucuuugugc.....................   7      0      dv1
...........................................................uagcaaccgucuuugugcG....................   2      1      dv1
...........................................................uGgcaaccgucuuugugca....................   1      1      dv1
...........................................................uagcaaccgucuuGgugca....................   1      1      dv1
...........................................................uagcaaccgucuCugugca....................   1      1      dv1
...........................................................uagcaaccgucuuuguUca....................   2      1      dv1
...........................................................uagcaaccgucuuuUugca....................   4      1      dv1
...........................................................uagcaaccgucuuugugAa....................   1      1      dv1
...........................................................uagcaaccgucuuugugca....................   380    0      dv1
...........................................................uagcaaccgucuuugCgca....................   1      1      dv1
...........................................................Gagcaaccgucuuugugca....................   1      1      dv1
...........................................................uagcaGccgucuuugugca....................   1      1      dv1
...........................................................uagcaaccgucCuugugca....................   1      1      dv1
...........................................................uagcaaccgucuuugugcC....................   35     1      dv1
...........................................................uagAaaccgucuuugugca....................   1      1      dv1
...........................................................uagcaaccgucuuuUugcaa...................   3      1      dv1
...........................................................uagcaaccgucuuugugcCa...................   12     1      dv1
...........................................................uagcaaccgucuuugCgcaa...................   2      1      dv1
...........................................................uagcaacAgucuuugugcaa...................   1      1      dv1
...........................................................uagcaaccguAuuugugcaa...................   1      1      dv1
...........................................................uagcaaccguUuuugugcaa...................   1      1      dv1
...........................................................uagcaaccgucuuuAugcaa...................   1      1      dv1
...........................................................uagcaaccgCcuuugugcaa...................   1      1      dv1
...........................................................Cagcaaccgucuuugugcaa...................   4      1      dv1
...........................................................uagcaaccgucuuuguUcaa...................   3      1      dv1
...........................................................uagcaaccgucCuugugcaa...................   2      1      dv1
...........................................................uagcaaccgucuCugugcaa...................   1      1      dv1
...........................................................uagUaaccgucuuugugcaa...................   1      1      dv1
...........................................................uagcaaccgucuuuguCcaa...................   1      1      dv1
```

```
                   dvi-miR-9692-5p
                                                            dvi-miR-9692-3p
gagaggcgccaauaucauauuguacauugaacgguagcuaucggauguauuugauaauccuagcaaccgucuuugugcaaaacgauguggcacacauu

............................................................uagcaaccgucGuugugcaa..................    1      1      dv1
............................................................uagcaaccgucuuugugcaC..................    24     1      dv1
............................................................uaCcaaccgucuuugugcaa..................    2      1      dv1
............................................................uagcaaccgucuuugugcaa..................    984    0      dv1
............................................................uaUcaaccgucuuugugcaa..................    1      1      dv1
............................................................uagcaaccgucuGugugcaa..................    1      1      dv1
............................................................Gagcaaccgucuuugugcaa..................    5      1      dv1
............................................................uagcaaccgucuuuCugcaa..................    4      1      dv1
............................................................uagcaaccgGcuuugugcaa..................    3      1      dv1
............................................................uagAaaccgucuuugugcaa..................    1      1      dv1
............................................................uagcGaccgucuuugugcaa..................    2      1      dv1
............................................................uagcaGccgucuuugugcaa..................    1      1      dv1
............................................................uaUcaaccgucuuugugcaaa.................    2      1      dv1
............................................................uagcaGccgucuuugugcaaa.................    2      1      dv1
............................................................uagcaaccgucuuugugcaaa.................    920    0      dv1
............................................................uCgcaaccgucuuugugcaaa.................    1      1      dv1
............................................................uagcaaccgucuuugugcaaC.................    19     1      dv1
............................................................uagcaaccgucuuugugcaCa.................    42     1      dv1
............................................................uagcaaccgucuuuguCcaaa.................    2      1      dv1
............................................................uagcaCccgucuuugugcaaa.................    1      1      dv1
............................................................uagcaaccgucCuugugcaaa.................    3      1      dv1
............................................................uagcCaccgucuuugugcaaa.................    1      1      dv1
............................................................uagcaaccgucuuugugcCaa.................    16     1      dv1
............................................................uagcaaccgucuCugugcaaa.................    1      1      dv1
............................................................uagcaaccgucuuuguUcaaa.................    1      1      dv1
............................................................uagcaaccgGcuuugugcaaa.................    2      1      dv1
............................................................uagcaaccgucuuugugcaaG.................    1      1      dv1
............................................................uagcaaccgucuuugCgcaaa.................    1      1      dv1
............................................................uagcaaccgucuuuguAcaaa.................    2      1      dv1
............................................................uGgcaaccgucuuugugcaaa.................    1      1      dv1
............................................................uagcaaccgucuuugugcaGa.................    1      1      dv1
............................................................uagcaaccgucuuuUugcaaa.................    1      1      dv1
............................................................uagcGaccgucuuugugcaaa.................    1      1      dv1
............................................................uagcaaccgucuuCgugcaaa.................    1      1      dv1
............................................................uagcaaccgucuuugugcaaU.................    2      1      dv1
............................................................uagcaaccgucuuugGgcaaa.................    1      1      dv1
............................................................uagcaaccgucuuugugcUaa.................    1      1      dv1
............................................................uagcaaccgucuuugugUaaa.................    2      1      dv1
............................................................uagcaacUgucuuugugcaaa.................    1      1      dv1
............................................................uagcaaccgucuuugugcaCaa................    10     1      dv1
............................................................uagcaaccgucuuugugcaaaC................    9      1      dv1
............................................................uagcaaccgucuuugugcGaaa................    2      1      dv1
............................................................uagcUaccgucuuugugcaaaa................    1      1      dv1
............................................................uagcaaccgucCuugugcaaaa................    1      1      dv1
............................................................uagcaaccgucuuugugcaUaa................    1      1      dv1
............................................................uaUcaaccgucuuugugcaaaa................    1      1      dv1
............................................................uagcaaccgucuuCgugcaaaa................    1      1      dv1
............................................................uagcaaccgucuuugugcaaCa................    15     1      dv1
............................................................uagcaaccgucuuugugcaaaa................    315    0      dv1
............................................................uagcaaccgucuuugCgcaaaa................    2      1      dv1
............................................................uagcaaccgucuuugugUaaaa................    1      1      dv1
............................................................uagcaaccgucuuugugcaaaU................    1      1      dv1
............................................................uagcaaccgucuuuguUcaaaa................    1      1      dv1
............................................................uagcaaccgucuuugugcCaaa................    1      1      dv1
............................................................uagcaaccgucuuugugcaaaaU...............    5      1      dv1
............................................................uagcaaccgucuuugugcaaaaA...............    17     1      dv1
............................................................uagcaaccgucuuugugcaaaaG...............    1      1      dv1
............................................................uagcaaccgucuuugugcaaaUc...............    1      1      dv1
.............................................................agcaaccgucuuugugcaa..................    1      0      dv1
```

miRBase precursor : dvi-mir-9693
Total read count : 14951
dvi-miR-9693-5p read count : 90
dvi-miR-9693-3p read count : 13964
remaining reads : 897

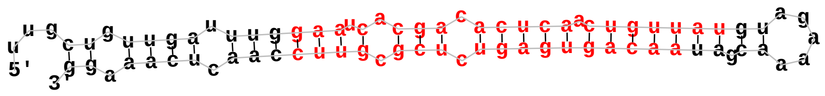

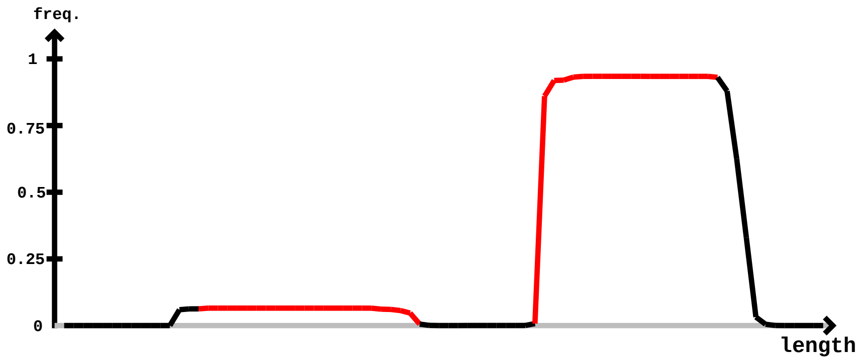


```
                                                                  dvi-miR-9693-3p
                 dvi-miR-9693-5p
5'- uugcuguugauuuggaaucacgacacucaacuguuauguagaaaacgauaacagugagucucgcguuccaacucaaagg   -3'    exp
    ...((.((((.((((((.(.(((.(((((.(((((((((.....)).))))))))))).))).))))))).)))))).     reads       mm          sample
    ..........uuggaaucacgacacucaac............................................     1           0           dv1
    ..........uuggaaucacgacacucaCcu...........................................     1           1           dv1
    ..........uuggaaucacgacacucCacu...........................................     1           1           dv1
    ..........uuggaaucacgacacucaacu...........................................     32          0           dv1
    ..........uuggaaucacUacacucaacu...........................................     1           1           dv1
    ..........uuggaaucacgacCcucaacu...........................................     2           1           dv1
    ..........uuggaaucacgacacucaacug..........................................     5           0           dv1
    ..........uuggaaucacgacacucaacuC..........................................     3           1           dv1
    ..........uuggaaucacgacacucaacuU..........................................     1           1           dv1
    ..........uuggaaucacgacacucaacugu.........................................     19          0           dv1
    ..........uuggaaucacgacacucaacuCu.........................................     1           1           dv1
    ..........uuggaaucacgacacucCacuguu........................................     1           1           dv1
    ..........uuggaaucacgacacucaacuguC........................................     5           1           dv1
    ..........uuggaaucacgacacucaacuguu........................................     31          0           dv1
    ..........uuggaaucacgacacucaCcuguua.......................................     1           1           dv1
    ..........uuggaaucacgacacucaacuguuU.......................................     6           1           dv1
    ..........uuggaaucacgacacucaacuguuC.......................................     209         1           dv1
    ..........uuggaaucacgacacucaacuguua.......................................     6           0           dv1
    ..........uuggaaucacgacacucaacuguuCu......................................     18          1           dv1
    ..........uuggaaucacgacacucaacuguuauC.....................................     1           1           dv1
    ...........uggaaucacgacacucaacu...........................................     1           0           dv1
    ...........uggaaucacgacacucaacug..........................................     2           0           dv1
    ...........uggaaucacgacacucaacuguu........................................     2           0           dv1
    ...........uggaaucacgacacucCacuguu........................................     1           1           dv1
    ...........uggaaucacgacacucaacuguua.......................................     2           0           dv1
    ...........uggaaucacgacacucaacuguuC.......................................     5           1           dv1
    ............ggaaucacgacacucaacuguua.......................................     1           0           dv1
    .............gaaucacgacacucaacuguua.......................................     1           0           dv1
    .............gaaucacgacacucaacuguuau......................................     7           0           dv1
    .............gaaucacgacacucaCcuguuau......................................     1           1           dv1
    .............gaaucacgacacucaacuguuauC.....................................     6           1           dv1
    .............................................uaacagugagucucgcguuc..........     4           0           dv1
    .............................................uaacagugagucucgcguucc.........     6           0           dv1
    .............................................Caacagugagucucgcguucc.........     3           1           dv1
```

dvi-miR-9693-3p

dvi-miR-9693-5p

uugcuguugauuuggaaucacgacacucaacuguuauguagaaaacgauaacagugagucucgcguuccaacucaaagg

....Caacagugagucucgcguucca.........	3	1	dv1
....uaacagugagucucgcguuccC.........	2	1	dv1
....uaacagugagucucgcguucca.........	3	0	dv1
....Caacagugagucucgcguuccaa........	1	1	dv1
....aacagugagucucgcguu............	11	0	dv1
....aacagugagucucgcguC............	2	1	dv1
....aacagugagucucgcguuc...........	179	0	dv1
....aacagugaUucucgcguuc...........	1	1	dv1
....aacagugagucucgcguuA...........	1	1	dv1
....aGcagugagucucgcguuc...........	1	1	dv1
....aacagugagucucgcguCc...........	1	1	dv1
....aacaUugagucucgcguuc...........	1	1	dv1
....aacagugagucCcgcguuc...........	1	1	dv1
....aacagugagucucgcUuucc..........	2	1	dv1
....aacagGgagucucgcguucc..........	1	1	dv1
....aacagugagCcucgcguucc..........	1	1	dv1
....aacagugagucuUgcguucc..........	1	1	dv1
....aacagugagucucgcgGucc..........	1	1	dv1
....aacagugagAcucgcguucc..........	1	1	dv1
....aacagugagucucgcguucc..........	820	0	dv1
....aacagugagucucgcguuUc..........	2	1	dv1
....aacagugagucucgcgCucc..........	1	1	dv1
....aacaguCagucucgcguucc..........	1	1	dv1
....aacCgugagucucgcguucc..........	1	1	dv1
....aacagCgagucucgcguucc..........	1	1	dv1
....aacagugagucucgcCuucc..........	3	1	dv1
....aacaCugagucucgcguucc..........	2	1	dv1
....aacagugagucucgcguucU..........	2	1	dv1
....aacagugagucCcgcguucc..........	5	1	dv1
....aacaguUagucucgcguucc..........	2	1	dv1
....aacagugagucucgUguucc..........	1	1	dv1
....aacagugagucucUcguucc..........	3	1	dv1
....aCcagugagucucgcguucc..........	1	1	dv1
....aGcagugagucucgcguucc..........	2	1	dv1
....aacagugaAucucgcguucc..........	1	1	dv1
....Cacagugagucucgcguucc..........	2	1	dv1
....aacagugagucucgcguGcc..........	1	1	dv1
....aacagugagucucCcguucc..........	1	1	dv1
....aacagugaUucucgcguucc..........	2	1	dv1
....aacagugagucucgcguuccC.........	68	1	dv1
....Gacagugagucucgcguucca.........	1	1	dv1
....aacagugagucucgcgGucca.........	2	1	dv1
....aacCgugagucucgcguucca.........	1	1	dv1
....aacagugagucucAcguucca.........	1	1	dv1
....aacaguAagucucgcguucca.........	1	1	dv1
....aacagugagucucgAguucca.........	1	1	dv1
....aacagugaUucucgcguucca.........	1	1	dv1
....aacagugGgucucgcguucca.........	1	1	dv1
....aacagugagCcucgcguucca.........	3	1	dv1
....aacagugagucucgcguuccU.........	13	1	dv1
....aacagugagucucgcguucUa.........	1	1	dv1
....aacagGgagucucgcguucca.........	1	1	dv1
....aacaguUagucucgcguucca.........	2	1	dv1
....aacagugagucucgcguCcca.........	1	1	dv1
....aacaguCagucucgcguucca.........	1	1	dv1
....aacagugagucCcgcguucca.........	1	1	dv1
....aGcagugagucucgcguucca.........	2	1	dv1
....aacagugagucucgcguucca.........	516	0	dv1
....aacagugagucucgcguuccaC........	45	1	dv1
....aacagugagucucgcgGuccaa........	2	1	dv1
....aacaCugagucucgcguuccaa........	2	1	dv1
....aacagugagucucgcCuuccaa........	2	1	dv1
....aacaguUagucucgcguuccaa........	1	1	dv1
....aacagugagucucgcguucUaa........	2	1	dv1
....Gacagugagucucgcguuccaa........	2	1	dv1
....aacagugaguAucgcguuccaa........	1	1	dv1
....aacagugaCucucgcguuccaa........	1	1	dv1
....aacagugagucucgcguCccaa........	2	1	dv1
....aacagugagucucCcguuccaa........	1	1	dv1

dvi-miR-9693-3p

dvi-miR-9693-5p

uugcuguugauuuggaaucacgacacucaacuguuauguagaaaacgauaacagugagucucgcguucccaacucaaagg

....aacagugagucucgcguuccaa.........	924	0	dv1
....aacagCgagucucgcguuccaa.........	2	1	dv1
....aacagugagucucgcgCuccaa.........	2	1	dv1
....aacagugagucucgcguGccaa.........	1	1	dv1
....aacagugagucucgcguuccCa.........	81	1	dv1
....aCcagugagucucgcguuccaa.........	1	1	dv1
....aacagugagucucgcguuccGa.........	2	1	dv1
....aacaUugagucucgcguuccaa.........	1	1	dv1
....aacaAugagucucgcguuccaa.........	1	1	dv1
....aacagugagucucUcguuccaa.........	1	1	dv1
....aacagugaUucucgcguuccaa.........	3	1	dv1
....aacagugGgucucgcguuccaa.........	4	1	dv1
....aacagugagucuUgcguuccaa.........	1	1	dv1
....aacagugCgucucgcguuccaa.........	1	1	dv1
....aacagugagucCcgcguuccaa.........	1	1	dv1
....aacagugagucucgcguuccUa.........	3	1	dv1
....aacagugagucucgcguuccaU.........	25	1	dv1
....aacagugaAucucgcguuccaa.........	2	1	dv1
....aacagugagucucgcUuuccaa.........	1	1	dv1
....aacagugagucucgGguuccaa.........	1	1	dv1
....aacagGgagucucgcguuccaa.........	1	1	dv1
....aacagugagucuAgcguuccaa.........	1	1	dv1
....Cacagugagucucgcguuccaa.........	3	1	dv1
....aaAagugagucucgcguuccaa.........	1	1	dv1
....aacagAgagucucgcguuccaa.........	1	1	dv1
....aacagugagucucgcguuccaCc........	1	1	dv1
....Cacagugagucucgcguuccaac........	1	1	dv1
....aacagugagucucgcguuccaaU........	43	1	dv1
....aacagugagucucgcguuccaaA........	25	1	dv1
....aacagugagucucgcguuccaaG........	1	1	dv1
....aacagugagucucgcguuccaUc........	1	1	dv1
....aacagugagucucgcguuccaac........	6	0	dv1
....aacagugagucucgcguuccaacu.......	1	0	dv1
....aacagugagucucgcguuccCacu.......	1	1	dv1
....aacagugagucucgcguuccaUcu.......	1	1	dv1
....aacagugagucucgcguuccaaUu.......	1	1	dv1
....aacagugagucucgcguuccaaAu.......	1	1	dv1
....acagugagucucgcguuc............	6	0	dv1
....acagugagucucgGguucc...........	1	1	dv1
....acagugagucucgcguucc...........	19	0	dv1
....acagugagucucgcguucU...........	2	1	dv1
....acagugagucucgcguuccC..........	2	1	dv1
....acagugagucucgcguucca..........	59	0	dv1
....acagugagucucgcguuUcaa.........	1	1	dv1
....acagugagucucUcguuccaa.........	2	1	dv1
....acagugagucucgcguuccaU.........	8	1	dv1
....acagugagucucgcguuccaC.........	3	1	dv1
....acagugagucucgcguuccaG.........	1	1	dv1
....acagCgagucucgcguuccaa.........	1	1	dv1
....acagugagucucgcguuccaa.........	56	0	dv1
....acagugagucucgcguuccCa.........	10	1	dv1
....acagugagucucgcguuccaac........	4	0	dv1
....acagugagucucCcguuccaac........	3	1	dv1
....acagugagucucgcguuccaUc........	1	1	dv1
....acagugagucucgcguuccaaA........	4	1	dv1
....acagugagucucgcguuccaaU........	31	1	dv1
....acagugagucucgcguuccaacC.......	2	1	dv1
....acagugagucucgcguuccaaUu.......	6	1	dv1
....acagugagucucgcguuccaacu.......	1	0	dv1
....cagugagucucgcguucca..........	1	0	dv1
....cagugagucucgcguuccaa.........	2	0	dv1
....caUugagucucgcguuccaacu.......	1	1	dv1
....cagugagucucgcguuccaacu.......	2	0	dv1
....agugagucucgcguuAca..........	1	1	dv1
....agugagucucgcguuccC..........	2	1	dv1
....agugagucucgcguucca..........	15	0	dv1
....agugagucucgcguuccaa.........	2	0	dv1
....agugagucucgcguuccaaU........	1	1	dv1
....agugagucucgcguuccaacu.......	1	0	dv1

```
                                                  dvi-miR-9693-3p
              dvi-miR-9693-5p
uugcuguugauuuggaaucacgacacucaacuguuauguagaaaacgauaacagugagucucgcguucccaacucaaagg

.....................................................gugagucucgcguuccCa........	1	1	dv1
.....................................................Uugagucucgcguuccaa........	1	1	dv1
.....................................................gugagucucgcguuccaa........	14	0	dv1
......................................................ugagucucgcguuccaacu......	1	0	dv1

...........uuggaaucacgacacucaacu...............................................	2	0	arg
...........uuggaaucacgacacucaacuC..............................................	1	1	arg
...........uuggaaucacgacacucaacug..............................................	1	0	arg
...........uuggaaucaUgacacucaacugu.............................................	1	1	arg
...........uuggaaucacgacacucaacugu.............................................	22	0	arg
...........uuggaaucacgacacucaacuguu............................................	6	0	arg
...........uuggaaucacgacacucaacuguuC...........................................	18	1	arg
...........uuggaaucacgacacucaacuguuU...........................................	1	1	arg
...........uuggaaucacgacacucaacuguuCu..........................................	3	1	arg
............uggaaucacgacacucaacugu.............................................	1	0	arg
............uggaaucacgacacucaacuguu............................................	1	0	arg
............uggaaucacgacacucaacuguua...........................................	2	0	arg
............uggaaucacgacacucaacuguuC...........................................	2	1	arg
..............gaaucacgacacucaacuguuau..........................................	1	0	arg
................................................uaacagugagucucgcguuc...........	4	0	arg
................................................Caacagugagucucgcguucc..........	2	1	arg
................................................uaacagugagucucgUguucc..........	1	1	arg
................................................uaacagugagucucgcguucc..........	14	0	arg
................................................uaacagugagucucgcguucca.........	6	0	arg
................................................uaacagugagucucgcguuccC.........	1	1	arg
................................................Caacagugagucucgcguucca.........	3	1	arg
................................................uaacagugagucucgcguuccU.........	1	1	arg
................................................uaacagugagucCcgcguucca.........	1	1	arg
.................................................aacagugagAcucgcguu............	1	1	arg
.................................................aacagugagucucgcgGu............	1	1	arg
.................................................aacagugagucucgcguu............	13	0	arg
.................................................aacagugagucucgcguC............	5	1	arg
.................................................aacagugagucuAgcguuc...........	1	1	arg
.................................................aacagugagucCcgcguuc...........	1	1	arg
.................................................aacagugagCcucgcguuc...........	1	1	arg
.................................................aacagugagucucgcguuU...........	1	1	arg
.................................................aacaguAagucucgcguuc...........	1	1	arg
.................................................aacagugGgucucgcguuc...........	1	1	arg
.................................................aacagGgagucucgcguuc...........	2	1	arg
.................................................aacagugagucucgcAuuc...........	1	1	arg
.................................................aacagugaguUucgcguuc...........	1	1	arg
.................................................aacagugagucucAcguuc...........	1	1	arg
.................................................aacagugagucucgcguuc...........	300	0	arg
.................................................aacagugagucucgUguuc...........	1	1	arg
.................................................aacaguUagucucgcguuc...........	1	1	arg
.................................................aacaUugagucucgcguuc...........	1	1	arg
.................................................aGcagugagucucgcguuc...........	1	1	arg
.................................................aacagugaAucucgcguuc...........	2	1	arg
.................................................aacagugagucucUcguuc...........	1	1	arg
.................................................Gacagugagucucgcguucc..........	4	1	arg
.................................................aacagugagucucgcguucA..........	1	1	arg
.................................................aacagugagucucgcguucG..........	1	1	arg
.................................................aacaAugagucucgcguucc..........	2	1	arg
.................................................aacagugagucCcgcguucc..........	1	1	arg
.................................................aacagugagucucgcUuucc..........	5	1	arg
.................................................aacagugagucuAgcguucc..........	1	1	arg
.................................................aacagugGgucucgcguucc..........	2	1	arg
.................................................aacaguAagucucgcguucc..........	4	1	arg
.................................................aGcagugagucucgcguucc..........	3	1	arg
.................................................aacagugagAcucgcguucc..........	2	1	arg
.................................................aacagugaguUucgcguucc..........	1	1	arg
.................................................aacagugagucuUgcguucc..........	1	1	arg
.................................................aacagugagucucgcgCucc..........	2	1	arg
.................................................aacagugUgucucgcguucc..........	1	1	arg
.................................................aacagugagucucgcguGcc..........	1	1	arg
.................................................aacagugagucucgcguucc..........	1241	0	arg
.................................................aacagugagucucCcguucc..........	4	1	arg
.................................................aacagugagucucgcgGucc..........	6	1	arg
.................................................aacagGgagucucgcguucc..........	1	1	arg
```

dvi-miR-9693-3p

dvi-miR-9693-5p

uugcuguugauuuggaaucacgacacucaacuguuauguagaaaacgauaacagugagucucgcguucccaacucaaagg

....aUcagugagucucgcguucc...........	3	1	arg
....aacagugagucucgcCuucc...........	2	1	arg
....aacagugagucucAcguucc...........	4	1	arg
....aacagugagucucgcguucU...........	4	1	arg
....aaUagugagucucgcguucc...........	1	1	arg
....aacagugagucucgAguucc...........	1	1	arg
....aacagugagucucgcguuUc...........	5	1	arg
....aacagugagGcucgcguucc...........	1	1	arg
....aacagugagucucUcguucc...........	3	1	arg
....aacagugagucucgcguuAc...........	2	1	arg
....aacagugaAucucgcguucc...........	1	1	arg
....aacagugagucucgcguAcca..........	1	1	arg
....aacagugagucuUgcguucca..........	2	1	arg
....aacagugagucucgcCuucca..........	1	1	arg
....aacagugagucucgcgCucca..........	3	1	arg
....aacGgugagucucgcguucca..........	3	1	arg
....aacagugagucCcgcguucca..........	4	1	arg
....aacagugagucucgcguuccC..........	31	1	arg
....aacagugagCcucgcguucca..........	1	1	arg
....aacagugagucucgcguuccG..........	2	1	arg
....aacagugagucucgcguCcca..........	2	1	arg
....aacagugagucucgcguucUa..........	5	1	arg
....Uacagugagucucgcguucca..........	1	1	arg
....aacaCugagucucgcguucca..........	3	1	arg
....Gacagugagucucgcguucca..........	2	1	arg
....aacagugagucucgcUuucca..........	2	1	arg
....aacagugagucucgcguuUca..........	2	1	arg
....aacagugagucucgcgAucca..........	1	1	arg
....aacagugagucucgcguucca..........	1445	0	arg
....aacagugagGcucgcguucca..........	2	1	arg
....aacagGgagucucgcguucca..........	2	1	arg
....aacagugagucucgcgGucca..........	4	1	arg
....aaAagugagucucgcguucca..........	1	1	arg
....aacagugagucucgcAuucca..........	1	1	arg
....aacagugagucucgUguucca..........	3	1	arg
....aacagugagucucgcguuccU..........	28	1	arg
....aacagugagucucgAguucca..........	2	1	arg
....aGcagugagucucgcguucca..........	4	1	arg
....aacagCgagucucgcguucca..........	4	1	arg
....aacagugaguUucgcguucca..........	1	1	arg
....aacagugGgucucgcguucca..........	5	1	arg
....aUcagugagucucgcguucca..........	3	1	arg
....aacaAugagucucgcguucca..........	2	1	arg
....aaUagugagucucgcguucca..........	8	1	arg
....aacagugagucucAcguucca..........	1	1	arg
....aacaguAagucucgcguucca..........	4	1	arg
....aacGgugagucucgcguuccaa.........	3	1	arg
....aacagugagucucgcgCuccaa.........	1	1	arg
....aacagugagucucgcguucUaa.........	1	1	arg
....aacagugagucucgcUuuccaa.........	1	1	arg
....aacagugagucucgcguuccaU.........	7	1	arg
....aacagugagucucgcguCccaa.........	3	1	arg
....aacagCgagucucgcguuccaa.........	3	1	arg
....aacagugagucCcgcguuccaa.........	1	1	arg
....aacagugagucucUcguuccaa.........	1	1	arg
....aacagugagucucgcgAuccaa.........	1	1	arg
....aacagugagucucgcguuccUa.........	2	1	arg
....aacagugagucucgcAuuccaa.........	1	1	arg
....aacagugCgucucgcguuccaa.........	1	1	arg
....aacagugagucucgcguucAaa.........	2	1	arg
....aCcagugagucucgcguuccaa.........	1	1	arg
....aacaguAagucucgcguuccaa.........	1	1	arg
....aacagGgagucucgcguuccaa.........	2	1	arg
....aacagugagucucgcguuccCa.........	11	1	arg
....aacagugagucucgcCuuccaa.........	2	1	arg
....aacagugagucucgcguuUcaa.........	1	1	arg
....aacagugaguAucgcguuccaa.........	1	1	arg
....aacagugagucucgcguuccaC.........	3	1	arg
....Uacagugagucucgcguuccaa.........	1	1	arg

```
                                                 dvi-miR-9693-3p
              dvi-miR-9693-5p
uugcuguugauuuggaaucacgacacucaacuguuauguagaaaacgauaacagugagucucgcguucccaacucaaagg

.................................................aacagugGgucucgcguuccaa.........    1        1        arg
.................................................aacagugagucucgAguuccaa.........    1        1        arg
.................................................aacagugagucucgcguuccaa.........    700      0        arg
.................................................aacUgugagucucgcguuccaa.........    1        1        arg
.................................................aacagugagCcucgcguuccaa.........    2        1        arg
.................................................aacagugagucucgcguuAcaa.........    1        1        arg
.................................................aacagugagucuAgcguuccaa.........    2        1        arg
.................................................aacagugagucucgcguuccGa.........    1        1        arg
.................................................aacagugagucuUgcguuccaa.........    1        1        arg
.................................................aacagugagucucgcgGuccaa.........    3        1        arg
.................................................aacagugagGcucgcguuccaa.........    1        1        arg
.................................................aacagugagucucgcguuccaUc........    1        1        arg
.................................................aacagugagucucgcguuccaaU........    8        1        arg
.................................................aacagugagucucgcguuccaaA........    34       1        arg
.................................................aacagugagucucgcguGccaac........    1        1        arg
.................................................aacagugagucucgcguuccaacC.......    1        1        arg
.................................................aacagugagucucgcguuccaaUu.......    2        1        arg
..................................................acagugagucucgcguuc............    7        0        arg
..................................................acagugagucucgcguucU...........    1        1        arg
..................................................acagugagucucgcguucc...........    41       0        arg
..................................................acagugaCucucgcguucc...........    1        1        arg
..................................................acagugaguAucgcguucc...........    1        1        arg
..................................................acGgugagucucgcguucc...........    1        1        arg
..................................................acagugagAcucgcguucca..........    1        1        arg
..................................................acagugGgucucgcguucca..........    1        1        arg
..................................................acagugagucucgcguGcca..........    2        1        arg
..................................................acagugagucucgcguuccC..........    2        1        arg
..................................................acagugagucucgcguuccU..........    1        1        arg
..................................................acagugagucucgcguucca..........    126      0        arg
..................................................acagugagucuUgcguucca..........    2        1        arg
..................................................acagugagucucgUguucca..........    1        1        arg
..................................................acagugagucucgcguuUca..........    1        1        arg
..................................................aUagugagucucgcguucca..........    1        1        arg
..................................................acagugagucucgcguuccaa.........    23       0        arg
..................................................acagugagucucgcguuccCa.........    1        1        arg
..................................................acagugagucucgcUuuccaa.........    2        1        arg
..................................................acagugaUucucgcguuccaa.........    1        1        arg
..................................................acagugagucucgcguuccaaU........    9        1        arg
..................................................acagugagucucgcguuccaac........    3        0        arg
..................................................acagugagucucgcguuccaacu.......    1        0        arg
...................................................cagugagucucgcguuccaacu.......    1        0        arg
....................................................agGgagucucgcguucca..........    1        1        arg
....................................................agugagucucgcCuucca..........    1        1        arg
....................................................agugagucucgcguucca..........    91       0        arg
....................................................agugagucucUcguucca..........    1        1        arg
....................................................agugagucucgcguucUa..........    1        1        arg
....................................................agugagucucgcguuccU..........    1        1        arg
....................................................aguAagucucgcguucca..........    1        1        arg
....................................................agugagucucgcguuccaa.........    2        0        arg
....................................................agugagucucgcguuccaacu.......    2        0        arg
.....................................................gugagucucgcguuccaU.........    2        1        arg
.....................................................gugagucucgcguuccaa.........    5        0        arg
.....................................................gugagucucgcguuccaaU........    1        1        arg

...........uuggaaucacgacacucaac.................................................    2        0        dv9
...........uuggaaucacgacacucaacu................................................    15       0        dv9
...........uuggaaucacgacacucaacug...............................................    3        0        dv9
...........uCggaaucacgacacucaacugu..............................................    1        1        dv9
...........uuggaaucacgacacucaacugu..............................................    17       0        dv9
...........uuggaaucacgacacucGacuguu.............................................    1        1        dv9
...........uuggaaucacgacacucaacuguC.............................................    4        1        dv9
...........uuggaaucacgacacucCacuguu.............................................    1        1        dv9
...........uuggaaucacgacacucaacuguu.............................................    75       0        dv9
...........uuggaaucacgacacucaacuUuu.............................................    1        1        dv9
...........uuggaaucacgacacucaCcuguu.............................................    2        1        dv9
...........uuggaaucacgacacuAaacuguua............................................    1        1        dv9
...........uuggUaucacgacacucaacuguua............................................    1        1        dv9
...........uuggaaucacgacacucaacuguua............................................    7        0        dv9
...........uuggaaucacgacacucaacuguuC............................................    330      1        dv9
```

```
                                                  dvi-miR-9693-3p
                   dvi-miR-9693-5p
uugcuguugauuuggaaucacgacacucaacuguuauguagaaaacgauaacagugagucucgcguuccaacucaaagg

...........uuggaaucacgacacucaacuguuU...........................................    3     1    dv9
...........uuggaaucacgacacucaacuguuCu..........................................    24    1    dv9
............uggaaucacgacacucaacuguu............................................    5     0    dv9
............uggaaucacgacacucaacuguC............................................    2     1    dv9
............uggaaucacgacacucaacuguua...........................................    9     0    dv9
............uggaaucacgacacucaacuguuC...........................................    10    1    dv9
............ugUaaucacgacacucaacuguua...........................................    1     1    dv9
............uggaaucacgacacucaacuguCa...........................................    1     1    dv9
.............ggaaucacgacacucaacuguuau..........................................    2     0    dv9
..............gaaucacgacacucaacuguu............................................    1     0    dv9
..............gaaucacgacacucaacuguua...........................................    2     0    dv9
..............Uaaucacgacacucaacuguua...........................................    1     1    dv9
..............gaaucacgacacucaacuguuau..........................................    15    0    dv9
..............Uaaucacgacacucaacuguuau..........................................    1     1    dv9
..............gaaucacgacCcucaacuguuau..........................................    1     1    dv9
..............gaaucacgacacucaacuguuauC.........................................    3     1    dv9
.........................................aaaacgauaacagugaguc...................    3     0    dv9
...............................................auaacagugagucucAcguuc...........    2     1    dv9
................................................uaacagugagucucgcguuc...........    7     0    dv9
................................................Aaacagugagucucgcguucc..........    1     1    dv9
................................................uaacagugagucucgcguucc..........    16    0    dv9
................................................Caacagugagucucgcguucc..........    5     1    dv9
................................................uaacagugagucucgcguucca.........    10    0    dv9
................................................uaacagugagucucgcguuccG.........    1     1    dv9
................................................Caacagugagucucgcguucca.........    5     1    dv9
................................................uaacagugagucucgcguuccC.........    2     1    dv9
................................................Caacagugagucucgcguuccaa........    3     1    dv9
.................................................aacagugagucucgcguu............    19    0    dv9
.................................................aacagugagucucgcguC............    2     1    dv9
.................................................aacagugagucucgcguCc...........    4     1    dv9
.................................................aacagugaguUucgcguuc...........    1     1    dv9
.................................................aacaCugagucucgcguuc...........    1     1    dv9
.................................................aacagugagucCcgcguuc...........    1     1    dv9
.................................................aacagugagucucUcguuc...........    1     1    dv9
.................................................aacagugagucucgcguuc...........    218   0    dv9
.................................................aacagugCgucucgcguuc...........    1     1    dv9
.................................................aaUagugagucucgcguuc...........    2     1    dv9
.................................................aacagugaUucucgcguuc...........    1     1    dv9
.................................................aacagugagucucgcgCuc...........    1     1    dv9
.................................................aacagugagucucgcguuU...........    1     1    dv9
.................................................aacGgugagucucgcguucc..........    2     1    dv9
.................................................aacagugagucucgcguGcc..........    1     1    dv9
.................................................aacagugagucuAgcguucc..........    2     1    dv9
.................................................aacaCugagucucgcguucc..........    1     1    dv9
.................................................aacagugCgucucgcguucc..........    1     1    dv9
.................................................aacagugagucucgcUuucc..........    3     1    dv9
.................................................aacagugagucucgcgCucc..........    4     1    dv9
.................................................aacagugagucucgcguucc..........    1415  0    dv9
.................................................aacagugagucucgcguuAc..........    2     1    dv9
.................................................aacagugagucucgcguucU..........    2     1    dv9
.................................................aacagugagCcucgcguucc..........    2     1    dv9
.................................................aacagugagucucgcAuucc..........    1     1    dv9
.................................................Cacagugagucucgcguucc..........    4     1    dv9
.................................................aacagugagucucgUguucc..........    1     1    dv9
.................................................aacagugagucucgcguCcc..........    1     1    dv9
.................................................aacagugagucCcgcguucc..........    2     1    dv9
.................................................aaUagugagucucgcguucc..........    1     1    dv9
.................................................aacagugaUucucgcguucc..........    1     1    dv9
.................................................aacagugagucucUcguucc..........    2     1    dv9
.................................................aacagugGgucucgcguucc..........    2     1    dv9
.................................................aacagugagucucgcgGucc..........    1     1    dv9
.................................................aacagugagucuUgcguucc..........    1     1    dv9
.................................................aacagCgagucucgcguucc..........    2     1    dv9
.................................................aacagugagucucAcguucc..........    1     1    dv9
.................................................aacaAugagucucgcguucc..........    1     1    dv9
.................................................aacaguAagucucgcguucc..........    1     1    dv9
.................................................aacagugagucucgcCuucc..........    4     1    dv9
.................................................Uacagugagucucgcguucc..........    1     1    dv9
.................................................aacagugagucucCcguucc..........    2     1    dv9
```

```
                                                 dvi-miR-9693-3p
              dvi-miR-9693-5p
uugcuguugauuuggaaucacgacacucaacuguuauguagaaaacgauaacagugagucucgcguucccaacucaaagg

.................................................aacagugagucucgcgAucc...........	1	1	dv9
.................................................aacagugaCucucgcguucc...........	3	1	dv9
.................................................Cacagugagucucgcguucca..........	1	1	dv9
.................................................aUcagugagucucgcguucca..........	2	1	dv9
.................................................aacagugagucAcgcguucca..........	1	1	dv9
.................................................aacagugagucucUcguucca..........	2	1	dv9
.................................................aacagugaguUucgcguucca..........	3	1	dv9
.................................................aacagugagucucgcAuucca..........	1	1	dv9
.................................................aGcagugagucucgcguucca..........	1	1	dv9
.................................................aacagugagucGcgcguucca..........	2	1	dv9
.................................................aacagugagucucgcguCcca..........	2	1	dv9
.................................................aacagugagucucgcgCucca..........	4	1	dv9
.................................................aacagugagucucgcguuAca..........	1	1	dv9
.................................................aacaUugagucucgcguucca..........	1	1	dv9
.................................................aacagugagucCcgcguucca..........	1	1	dv9
.................................................aacagugagucucgcguuUca..........	1	1	dv9
.................................................aacCgugagucucgcguucca..........	1	1	dv9
.................................................aaAagugagucucgcguucca..........	1	1	dv9
.................................................aacagugagucuAgcguucca..........	2	1	dv9
.................................................aacagugagucucAcguucca..........	2	1	dv9
.................................................aacagugagAcucgcguucca..........	1	1	dv9
.................................................aacagugaCucucgcguucca..........	2	1	dv9
.................................................aacaguCagucucgcguucca..........	2	1	dv9
.................................................aacagugaUucucgcguucca..........	2	1	dv9
.................................................Gacagugagucucgcguucca..........	3	1	dv9
.................................................aacagugagucucgcUuucca..........	2	1	dv9
.................................................aacagugagucucgUguucca..........	8	1	dv9
.................................................aacagugagucucgcCuucca..........	1	1	dv9
.................................................aacagugGgucucgcguucca..........	4	1	dv9
.................................................aacagugagucucgcguuccG..........	5	1	dv9
.................................................aacagugagucucgcguuccU..........	14	1	dv9
.................................................aacagugagucucgcguGcca..........	1	1	dv9
.................................................aaUagugagucucgcguucca..........	4	1	dv9
.................................................aacagugagucucgcguucUa..........	5	1	dv9
.................................................aacagugagucucgcguucca..........	1426	0	dv9
.................................................aacagugagCcucgcguucca..........	4	1	dv9
.................................................aacagugagucuGgcguucca..........	1	1	dv9
.................................................aacagugUgucucgcguucca..........	1	1	dv9
.................................................aacGgugagucucgcguucca..........	1	1	dv9
.................................................aacagugagucucgcguuccC..........	145	1	dv9
.................................................aacagCgagucucgcguucca..........	2	1	dv9
.................................................aacagugagucucgcguucGa..........	1	1	dv9
.................................................aacagugagucuUgcguucca..........	1	1	dv9
.................................................aacaguUagucucgcguucca..........	1	1	dv9
.................................................aacagugGgucucgcguuccaa.........	2	1	dv9
.................................................aacagugagucucgcguuccaU.........	60	1	dv9
.................................................aacagugaUucucgcguuccaa.........	2	1	dv9
.................................................aacagugagucucUcguuccaa.........	6	1	dv9
.................................................aacagugagCcucgcguuccaa.........	2	1	dv9
.................................................aacagugagucucgcguucAaa.........	1	1	dv9
.................................................aacagugagucucgcCuuccaa.........	2	1	dv9
.................................................aacaguAagucucgcguuccaa.........	1	1	dv9
.................................................Uacagugagucucgcguuccaa.........	1	1	dv9
.................................................aacagugagucCcgcguuccaa.........	3	1	dv9
.................................................aacagugagucucgcguCccaa.........	2	1	dv9
.................................................aacagugaguUucgcguuccaa.........	2	1	dv9
.................................................aacaguUagucucgcguuccaa.........	1	1	dv9
.................................................aacagugagucucgcgAuccaa.........	2	1	dv9
.................................................aacagugagucucgcguuccUa.........	8	1	dv9
.................................................aacagugagucucgcAuuccaa.........	1	1	dv9
.................................................aacagugagucucgcgCuccaa.........	4	1	dv9
.................................................aacaguCagucucgcguuccaa.........	2	1	dv9
.................................................aacagugagucucgcguuccaC.........	99	1	dv9
.................................................aacagugagucuUgcguuccaa.........	2	1	dv9
.................................................aacagugagucucgGguuccaa.........	1	1	dv9
.................................................Cacagugagucucgcguuccaa.........	7	1	dv9
.................................................aacagugagucucAcguuccaa.........	1	1	dv9
.................................................aacagCgagucucgcguuccaa.........	2	1	dv9
.................................................aacagugUgucucgcguuccaa.........	1	1	dv9
```

```
                                             dvi-miR-9693-3p
              dvi-miR-9693-5p
uugcuguugauuuggaaucacgacacucaacuguuauguagaaaacgauaacagugagucucgcguuccaacucaaagg

.................................................aacagugagucucgcguuccGa........    1      1      dv9
.................................................aacaCugagucucgcguuccaa........    2      1      dv9
.................................................aacagugaguAucgcguuccaa........    1      1      dv9
.................................................aacagugagucuAgcguuccaa........    1      1      dv9
.................................................aacagGgagucucgcguuccaa........    1      1      dv9
.................................................aacagugagucucgcUuuccaa........    2      1      dv9
.................................................aacagugCgucucgcguuccaa........    3      1      dv9
.................................................aaUagugagucucgcguuccaa........    1      1      dv9
.................................................aacagugagucucgcguuccCa........    185    1      dv9
.................................................aacagugagucucgcguuccaa........    1831   0      dv9
.................................................aacaUugagucucgcguuccaa........    7      1      dv9
.................................................aacagugagucucgcguuccaG........    2      1      dv9
.................................................aacagugagucucgcguuAcaa........    1      1      dv9
.................................................aGcagugagucucgcguuccaa........    2      1      dv9
.................................................aUcagugagucucgcguuccaa........    1      1      dv9
.................................................aacagugaCucucgcguuccaa........    1      1      dv9
.................................................aacagugagucucgcguAccaa........    1      1      dv9
.................................................aacagugagucucgcgGuccaa........    1      1      dv9
.................................................aacGgugagucucgcguuccaa........    3      1      dv9
.................................................Gacagugagucucgcguuccaa........    1      1      dv9
.................................................aacagugagucucgAguuccaa........    1      1      dv9
.................................................aacagugagucucgcguuccCac.......    3      1      dv9
.................................................aacagugagucucgcguuccaUc.......    2      1      dv9
.................................................aacagugagucucgcguuccaac.......    21     0      dv9
.................................................aacagugagucucgcguuccaaU.......    61     1      dv9
.................................................aacagugagucucgcguuccaaG.......    1      1      dv9
.................................................aacagugagucucgcguuccaCc.......    1      1      dv9
.................................................aacagugagucucgcguuccaaA.......    61     1      dv9
.................................................aacagugagucucgcguuccaacC......    6      1      dv9
.................................................aacagugagucucgcguuccaacA......    1      1      dv9
.................................................aacagugagucucgcguuccaaAu......    1      1      dv9
.................................................aacagugagucucgcguuccaacu......    1      0      dv9
.................................................aacagugagucucgcguuccaaUu......    4      1      dv9
.................................................aacagugagucucgcguuccaaAuc.....    1      1      dv9
.................................................aacagugagucucgcguuccaaUuc.....    3      1      dv9
..................................................acagugagucucgcguuc...........    3      0      dv9
..................................................acagugagucucgcguucc..........    47     0      dv9
..................................................acagugagucucgcguucca.........    126    0      dv9
..................................................acagugagucucgcguCcca.........    1      1      dv9
..................................................acagugagucucgcguuccU.........    2      1      dv9
..................................................acagugagucucgcguuccC.........    8      1      dv9
..................................................acagugagucucgcguuccaa........    123    0      dv9
..................................................acagugagucucgcguCccaa........    1      1      dv9
..................................................acagugagucucgcguuccCa........    10     1      dv9
..................................................acagugagucucgcguuccaU........    4      1      dv9
..................................................acagugagucucgcguuccaC........    6      1      dv9
..................................................Ccagugagucucgcguuccaa........    1      1      dv9
..................................................acagugaUucucgcguuccaa........    1      1      dv9
..................................................acagugagucucgcguuccCac.......    1      1      dv9
..................................................acagugagucucgcguuccaac.......    10     0      dv9
..................................................acagugagucucgcguuccaaU.......    54     1      dv9
..................................................acagugagucucgcguuccaUc.......    3      1      dv9
..................................................acagugagucucgcguuccaCc.......    1      1      dv9
..................................................acagugagucucgcguuccaaA.......    4      1      dv9
..................................................acagugagucucgcguuccaacu......    6      0      dv9
..................................................acagugagucucgcguuccaacA......    1      1      dv9
..................................................acagugagucucgcguuccaacC......    5      1      dv9
..................................................acagugagucucgcguuccaaUu......    6      1      dv9
..................................................acagugagucucgcguuccaacCc.....    1      1      dv9
..................................................acagugagucucgcguuccaacuU.....    1      1      dv9
...................................................cagugagucucgcguuccC.........    1      1      dv9
...................................................cagugagucucgcguucca.........    2      0      dv9
...................................................cagugaUucucgcguuccaac.......    1      1      dv9
...................................................cagugagucucgcguuccCacu......    1      1      dv9
...................................................cagugagucucgcguuccaacu......    8      0      dv9
....................................................agugagucucgcguuccC.........    2      1      dv9
....................................................agugagucucgcguucca.........    34     0      dv9
....................................................agugaguUucgcguucca.........    1      1      dv9
....................................................agugGgucucgcguucca.........    1      1      dv9
```

```
                                                    dvi-miR-9693-3p
              dvi-miR-9693-5p
uugcuguugauuuggaaucacgacacucaacuguuauguagaaaacgauaacagugagucucgcguuccaacucaaagg

....................................................agugagucucgcguuUca.........    1    1    dv9
....................................................agugagucuUgcguuccaa........    1    1    dv9
....................................................agugagucucgcguuccaU........    2    1    dv9
....................................................agugagucucgcguuccaa........    2    0    dv9
....................................................agugagucucgcguuccaac.......    1    0    dv9
....................................................agugagucucgcguuccaacu......    1    0    dv9
.....................................................gugagucucgcguuccaa........    13   0    dv9
.....................................................gugagucucgcguuccCa........    2    1    dv9
.....................................................Uugagucucgcguuccaa........    2    1    dv9
......................................................ugagucucgcguuccaaA.......    2    1    dv9
```

miRBase precursor : dvi-mir-975
Total read count : 41976
dvi-miR-975-5p read count : 4350
dvi-miR-975-3p read count : 37626
remaining reads : 0

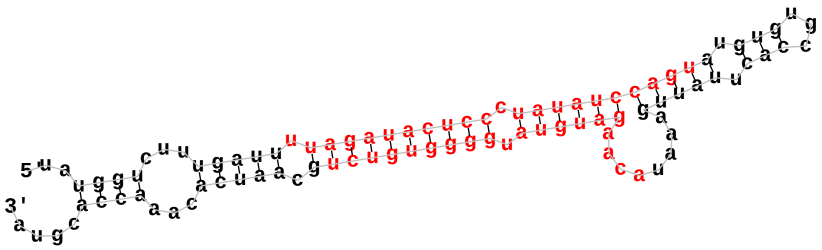

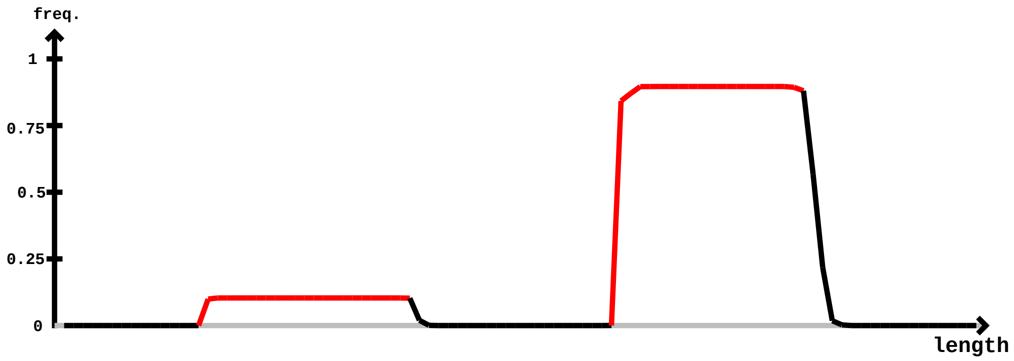


```
              dvi-miR-975-5p
                                          dvi-miR-975-3p
5'- uauggucuuugauuuuagauacucccuauauccaguaugugugccacuuauugaaauacaagauguauggggugucugcaaucacaaaccacgua  -3'   exp
    ..((((...(((((.((((((((((.((((((((((.(((...))).)))))........)))))).))))))))))).)))))...))))....   reads   mm   sample
    ..............uuagauacucccuauauccag............................................................   1       0    arg
    ..............Auagauacucccuauauccagu...........................................................   1       1    arg
    ..............uuagauacucccuauauccagu...........................................................   140     0    arg
    ..............uuagGuacucccuauauccagu...........................................................   1       1    arg
    ..............uuagauacucccuauauccaguC..........................................................   3       1    arg
    ..............uuagauacucccuauauccaguU..........................................................   9       1    arg
    ...............uagauaUucccuauauccagu...........................................................   1       1    arg
    ...............uagauacucccuauauccagu...........................................................   3       0    arg
    ...............uagauacucccuauauccaguU..........................................................   1       1    arg
    ................agauacucccuauauccaguau.........................................................   2       0    arg
    .......................................................Cacaagauguauggggugucu...................   2       1    arg
    ......................................................uacaagauguauggggugucug...................   2       0    arg
    .......................................................Cacaagauguauggggugucug..................   2       1    arg
    ........................................................acaagauguauggggugu.....................   76      0    arg
    ........................................................acaaAauguauggggugu.....................   1       1    arg
    ........................................................acaagauguauggAgugu.....................   1       1    arg
    ........................................................acaGgauguauggggugu.....................   1       1    arg
    ........................................................acaagGuguaugggguguc....................   1       1    arg
    ........................................................acaagaugGaugggguguc....................   1       1    arg
    ........................................................acaagauguauggggguguU....................   6       1    arg
    ........................................................acaagauguauggggugCc....................   1       1    arg
    ........................................................acaagauguauggggugGc....................   1       1    arg
    ........................................................acaagauguaugggguguc....................   287     0    arg
    ........................................................acaagauguauggggCguc....................   3       1    arg
    ........................................................acaagCuguaugggguguc....................   1       1    arg
    ........................................................acaagauguauggAguguc....................   1       1    arg
    ........................................................aUaagauguaugggguguc....................   1       1    arg
    ........................................................acaagauguGugggguguc....................   1       1    arg
    ........................................................acaagauguauggggugAcu...................   1       1    arg
    ........................................................acaagauguaugggCugucu...................   2       1    arg
    ........................................................acaaCauguauggggugucu...................   2       1    arg
    ........................................................acCagauguauggggugucu...................   1       1    arg
    ........................................................acaagaCguauggggugucu...................   6       1    arg
    ........................................................acaaAauguauggggugucu...................   4       1    arg
```

dvi-miR-975-5p

dvi-miR-975-3p

uauggucuuugauuuuagauacucccuauauccaguaugugugccacuuauugaaauacaagauguauggggugucugcaaucacaaaccacgua

....acaagauguaugggguCucu..................	2	1	arg
....acaGgauguauggggugucu..................	6	1	arg
....acUagauguauggggugucu..................	1	1	arg
....acaagauguaugCggugucu..................	4	1	arg
....acaagauguauggggugucG..................	2	1	arg
....acaagauguauggAgugucu..................	2	1	arg
....acaagauguaAggggugucu..................	4	1	arg
....acaagauCuauggggugucu..................	2	1	arg
....aAaagauguauggggugucu..................	7	1	arg
....acaagauguauggggugCcu..................	14	1	arg
....acaagauguaugAggugucu..................	7	1	arg
....acaagauguaCggggugucu..................	8	1	arg
....acaagauguaugggguAucu..................	5	1	arg
....Ccaagauguauggggugucu..................	1	1	arg
....acaagauguaugUggugucu..................	10	1	arg
....aUaagauguauggggugucu..................	15	1	arg
....acaagauguauggUgugucu..................	7	1	arg
....acaagauguauggCgugucu..................	4	1	arg
....acaagaugAauggggugucu..................	1	1	arg
....acaagauguauggggGgucu..................	26	1	arg
....acaagauguaugggguguAu..................	6	1	arg
....acaagauguauUgggugucu..................	2	1	arg
....acaagauguaugggguguUu..................	6	1	arg
....acaagauguauggggugucC..................	155	1	arg
....acaagauguaugggUugucu..................	2	1	arg
....Gcaagauguauggggugucu..................	5	1	arg
....acaagaugCauggggugucu..................	7	1	arg
....acaagCuguauggggugucu..................	1	1	arg
....acaagauguauggggAgucu..................	11	1	arg
....acaagauguCuggggugucu..................	1	1	arg
....acaagauguaugggguUucu..................	3	1	arg
....acaagaGguauggggugucu..................	1	1	arg
....acaagUuguauggggugucu..................	1	1	arg
....acaagauguauggggCgucu..................	38	1	arg
....acaagauguUuggggugucu..................	4	1	arg
....acaagaugGauggggugucu..................	10	1	arg
....acaagauguauggggugucu..................	6738	0	arg
....acGagauguauggggugucu..................	9	1	arg
....acaagauguGuggggugucu..................	8	1	arg
....acaagauguaugggAugucu..................	5	1	arg
....acaagauguauCgggugucu..................	2	1	arg
....acaUgauguauggggugucu..................	3	1	arg
....acaagauAuauggggugucu..................	1	1	arg
....acaagauguauggggugucA..................	10	1	arg
....acaagauguauAgggugucu..................	4	1	arg
....acaagauguaGggggugucu..................	2	1	arg
....acaaUauguauggggugucu..................	2	1	arg
....acaagauUuauggggugucu..................	1	1	arg
....acaagauguauggggugGcu..................	10	1	arg
....acaagGuguauggggugucu..................	8	1	arg
....acCagauguauggggugucug.................	2	1	arg
....acaagauUuauggggugucug.................	1	1	arg
....acaagauguauAgggugucug.................	8	1	arg
....acaagauguaugggguguUug.................	8	1	arg
....acaagCuguauggggugucug.................	2	1	arg
....acaagGuguauggggugucug.................	11	1	arg
....acaagauguaugggUugucug.................	3	1	arg
....acaagauguaugCggugucug.................	2	1	arg
....acGagauguauggggugucug.................	10	1	arg
....acaagauguauggggGgucug.................	19	1	arg
....acaagauguauCgggugucug.................	1	1	arg
....acaagauguauggCgugucug.................	1	1	arg
....acaagaugCauggggugucug.................	6	1	arg
....acaagauguauUgggugucug.................	2	1	arg
....acaagauguaugggguCucug.................	14	1	arg
....acaagauguaugggAugucug.................	5	1	arg
....acaagauguauggggugGcug.................	14	1	arg
....Ucaagauguauggggugucug.................	2	1	arg
....acaagaugGauggggugucug.................	8	1	arg

dvi-miR-975-5p

dvi-miR-975-3p

uauggucuuugauuuuagauacucccuauauccaguaugugugccacuuauugaaauacaagauguauggggugucugcaaucacaaaccacgua

....acaagauguauggggugucuU.................	56	1	arg
....acaaAauguauggggugucug.................	2	1	arg
....acaagauAuauggggugucug.................	3	1	arg
....acaagauguGuggggugucug.................	3	1	arg
....acaagauguauggggugucCg.................	6	1	arg
....acaagauguUuggggugucug.................	3	1	arg
....acaagauguaugggguUucug.................	7	1	arg
....acaagauguaugggguguAug.................	6	1	arg
....acaagauguaugggguAucug.................	6	1	arg
....acaagauguaCggggugucug.................	7	1	arg
....acaGgauguauggggugucug.................	15	1	arg
....aUaagauguauggggugucug.................	8	1	arg
....acaagauguauggggugucug.................	6606	0	arg
....acaagauguaugUggugucug.................	8	1	arg
....acaagauguauggAgugucug.................	3	1	arg
....acaagauguauggggugucuC.................	35	1	arg
....acaagauguaAggggugucug.................	1	1	arg
....Gcaagauguauggggugucug.................	11	1	arg
....acaagauguCuggggugucug.................	1	1	arg
....acaagauguauggggAgucug.................	5	1	arg
....acaagauguauggggugucuA.................	6	1	arg
....acaagauguauggggugucAg.................	1	1	arg
....acaagauguauggUgugucug.................	3	1	arg
....acaagauguaugAggugucug.................	2	1	arg
....acaagaugAauggggugucug.................	1	1	arg
....acaaCauguauggggugucug.................	3	1	arg
....acaagauguaugggCugucug.................	2	1	arg
....acaagauguauggggugCcug.................	16	1	arg
....acaagUuguauggggugucug.................	1	1	arg
....acaagaCguauggggugucug.................	5	1	arg
....acaagauguauggggCgucug.................	22	1	arg
....acaagauCuauggggugucug.................	2	1	arg
....acaagauguauggggugucGg.................	2	1	arg
....aAaagauguauggggugucug.................	2	1	arg
....acaagauguauggggugAcug.................	1	1	arg
....acaagauguaugggguguGug.................	2	1	arg
....acGagauguauggggugucugc................	3	1	arg
....acaagaugAauggggugucugc................	1	1	arg
....acaagauguaugUggugucugc................	1	1	arg
....acaagauguauggggugGcugc................	3	1	arg
....acaagauguauggggugucuUc................	30	1	arg
....acaagauguaugAggugucugc................	1	1	arg
....Gcaagauguauggggugucugc................	6	1	arg
....acaagauAuauggggugucugc................	4	1	arg
....acaagaGguauggggugucugc................	1	1	arg
....acaagGuguauggggugucugc................	3	1	arg
....acaagauguauggAgugucugc................	2	1	arg
....acaagauguaugggguUucugc................	2	1	arg
....acaagauguauggUgugucugc................	2	1	arg
....acaagauguauggggugucCgc................	4	1	arg
....acaagauguauggggAgucugc................	3	1	arg
....acaagauguauggggugCcugc................	4	1	arg
....acaagaugGauggggugucugc................	3	1	arg
....acaagauguauggggCgucugc................	9	1	arg
....acaagauguauggggugucuCc................	4	1	arg
....acaagauguaugggguAucugc................	1	1	arg
....acaagauguGuggggugucugc................	1	1	arg
....acaagauguauggggugucugG................	1	1	arg
....acaagaugCauggggugucugc................	3	1	arg
....acaagauguauggggugucugU................	905	1	arg
....acaagauguauggggugucGgc................	3	1	arg
....acaagauguauggggugucugA................	22	1	arg
....acaagauguaugggAugucugc................	3	1	arg
....aUaagauguauggggugucugc................	2	1	arg
....aAaagauguauggggugucugc................	2	1	arg
....acaagauguUuggggugucugc................	4	1	arg
....acaagauguaugggguguAugc................	2	1	arg
....acaagauguaCggggugucugc................	3	1	arg
....acaagauguauggggGgucugc................	8	1	arg

dvi-miR-975-5p

dvi-miR-975-3p

uauggucuuugauuuuagauacucccuauauccaguaugugugccacuuauugaaauacaagauguauggggugucugcaaucacaaaccacgua

....acaaCauguauggggugucugc................	1	1	arg
....acaagauguaugggguCucugc................	6	1	arg
....acaagauguauggggugucugc................	2113	0	arg
....acaagauguaugggCugucugc................	2	1	arg
....acaagauguauggggugucuAc................	2	1	arg
....acCagauguauggggugucugc................	1	1	arg
....acaGgauguauggggugucugc................	5	1	arg
....acaagauguauggggugucugcU...............	17	1	arg
....acaagauguauggggugucugcG...............	1	1	arg
....acaagauguauggggugucugAa...............	1	1	arg
....acaagauguauggggugucugUa...............	1	1	arg
....acaagauguauggggugucugcC...............	32	1	arg
....acaagauguauggggugucugca...............	2	0	arg
....acaagauguauggggugucugUaa..............	1	1	arg
....caagauguaugggguguc...................	32	0	arg
....caagauguaugggguguG...................	1	1	arg
....caagauguauggggugCcu..................	1	1	arg
....cGagauguauggggugucu..................	1	1	arg
....caagauguauggggugucC..................	3	1	arg
....caagauguauggggCgucu..................	1	1	arg
....caagauguauggggugucu..................	178	0	arg
....caagaCguauggggugucu..................	1	1	arg
....caagGuguauggggugucu..................	1	1	arg
....caagauguauggggugucA..................	1	1	arg
....caagauAuauggggugucu..................	1	1	arg
....caagauCuauggggugucu..................	1	1	arg
....caagauguauggggGgucu..................	1	1	arg
....Aaagauguauggggugucu..................	1	1	arg
....caagauguauggggugGcug.................	1	1	arg
....caagauguauggggCgucug.................	1	1	arg
....caagauguauggggugucug.................	72	0	arg
....caagauguauggggugucuC.................	2	1	arg
....caagaGguauggggugucug.................	1	1	arg
....caagauguauggggugucuA.................	1	1	arg
....caagauguauggggugucuU.................	1	1	arg
....caagauguauggggCgucugc................	1	1	arg
....caagauguauggggugucugU................	10	1	arg
....caagauguauggggugucugc................	165	0	arg
....caagauguaugggguUucugc................	1	1	arg
....caagauguauggggugucugG................	1	1	arg
....caagaugCauggggugucugc................	1	1	arg
....caagauguauggggugucGgc................	1	1	arg
....caGgauguauggggugucugc................	2	1	arg
....caagauguauggggGgucugc................	1	1	arg
....caagauguauggggugucuCc................	1	1	arg
....caagauguauggggugucCgc................	1	1	arg
....caagauguauggggugucuUc................	2	1	arg
....caagauguauggggugucugcC...............	2	1	arg
....caagauguauggggugucugcU...............	2	1	arg
....caagauguauggggugucugca...............	1	0	arg
....aagauguauggggAgucu..................	1	1	arg
....aagauguauggggugucC..................	1	1	arg
....aagauguauggggugucu..................	76	0	arg
....aagauguauggggugucuU.................	2	1	arg
....aagGuguauggggugucug.................	1	1	arg
....aagauguauggggCgucug.................	1	1	arg
....aagauguauggggugucCg.................	1	1	arg
....aagauguauggggugucug.................	73	0	arg
....aagauguauUgggugucugc................	1	1	arg
....aagauUuauggggugucugc................	1	1	arg
....aagauguauggggugucugU................	17	1	arg
....aagauguaugggguAucugc................	1	1	arg
....aagauguauggggugucugc................	232	0	arg
....aagauguaugggguCucugc................	2	1	arg
....aagauguaugggUugucugc................	1	1	arg
....aagauguauggggguguAugc................	2	1	arg
....aagaugCauggggugucugc................	3	1	arg
....aagauguauggggugucCgc................	2	1	arg
....aagauguauggggugucugA................	1	1	arg

```
              dvi-miR-975-5p
                                          dvi-miR-975-3p
uauggucuuugauuuuagauacucccuauauccaguaugugugccacuuauugaaauacaagauguauggggugucugcaaucacaaaccacgua

...........................................................aagauguauggggugucugG................    1      1      arg
...........................................................aagauguauggggugucGgc................    1      1      arg
...........................................................aagauguauggggugCcugca...............    1      1      arg
...........................................................aagUuguauggggugucugca...............    1      1      arg
...........................................................aagauguauggggugucuAca...............    1      1      arg
...........................................................aagauguauggggugucugAa...............    1      1      arg
...........................................................aagauguauggggCgucugca...............    1      1      arg
...........................................................aagauguauggggugucuCca...............    1      1      arg
...........................................................aagauguauggggugucugcC...............    12     1      arg
...........................................................aagauguauggggugucugca...............    113    0      arg
...........................................................aagauguauggggugucugcU...............    6      1      arg
...........................................................aagauguauggggGgucugca...............    1      1      arg
...........................................................aagauguauggggugucCgca...............    1      1      arg
...........................................................aagauguauggggugucugcaC..............    2      1      arg
...........................................................aagauguauggggugucugcaU..............    6      1      arg
...........................................................aagauguauggggugucugcaa..............    14     0      arg
...........................................................aagauguauggggugucugcaau.............    1      0      arg
............................................................agauguauggggugucug.................    2      0      arg
............................................................agauguauggggugucugc................    7      0      arg
.............................................................gauguauggggugucugc................    3      0      arg

..............uuagauacucccuauaucc..............................................................    1      0      dv9
..............uuagauacucccuauaucca.............................................................    1      0      dv9
..............uuagauacucccuauaucAag............................................................    1      1      dv9
..............uuagauacucccuGuauccag............................................................    1      1      dv9
..............uuagauacucccuauaCccag............................................................    1      1      dv9
..............uuagauacucccuauauccag............................................................    11     0      dv9
..............uuagauacucccuauauccaUu...........................................................    8      1      dv9
..............uGagauacucccuauauccagu...........................................................    3      1      dv9
..............uuagauacuUccuauauccagu...........................................................    2      1      dv9
..............Guagauacucccuauauccagu...........................................................    1      1      dv9
..............uuagauacucccuauaucUagu...........................................................    1      1      dv9
..............uuagauacucccuauauccGgu...........................................................    1      1      dv9
..............uuagauacucccuauauccUgu...........................................................    1      1      dv9
..............uuaUauacucccuauauccagu...........................................................    1      1      dv9
..............uuagauacucccuauauccagu...........................................................    1556   0      dv9
..............uuGgauacucccuauauccagu...........................................................    3      1      dv9
..............uCagauacucccuauauccagu...........................................................    1      1      dv9
..............Cuagauacucccuauauccagu...........................................................    1      1      dv9
..............uuagauacCcccuauauccagu...........................................................    3      1      dv9
..............uuagauacucccuaCauccagu...........................................................    1      1      dv9
..............uuagauGcucccuauauccagu...........................................................    1      1      dv9
..............uuagauacucccuauauccaCu...........................................................    3      1      dv9
..............uuagauacucccuauauccagG...........................................................    2      1      dv9
..............uuagauacucccuauaucAagu...........................................................    1      1      dv9
..............uuagauacuccUuauauccagu...........................................................    1      1      dv9
..............uuagaGacucccuauauccagu...........................................................    1      1      dv9
..............uuagGuacucccuauauccagu...........................................................    1      1      dv9
..............uuagauaAucccuauauccagu...........................................................    1      1      dv9
..............uuagauacucccuauCuccagu...........................................................    22     1      dv9
..............uuagauacucccuauauccagC...........................................................    4      1      dv9
..............uuagauacucccuauauccCgu...........................................................    47     1      dv9
..............uuagauacucccuCuauccagu...........................................................    6      1      dv9
..............uuagauacucccuGuauccagu...........................................................    1      1      dv9
..............uuagauacucAcuauauccagu...........................................................    1      1      dv9
..............uuagaCacucccuauauccagu...........................................................    3      1      dv9
..............uuagauacucccCauauccagu...........................................................    1      1      dv9
..............uuagauacucccGauauccagu...........................................................    1      1      dv9
..............uuagauacucccuauauccaguU..........................................................    11     1      dv9
..............uuagauacucccuauauccaguC..........................................................    417    1      dv9
..............uuagauacucccuauauccaguCu.........................................................    19     1      dv9
..............uuagauacucccuauauccaguaA.........................................................    1      1      dv9
...............uagauCcucccuauauccagu...........................................................    1      1      dv9
...............uagauacucccuauauccCgu...........................................................    2      1      dv9
...............uagauUcucccuauauccagu...........................................................    1      1      dv9
...............uagauacucccuauauccagu...........................................................    66     0      dv9
...............uagauacucccuauCuccagu...........................................................    1      1      dv9
...............uagauacucccuauaCccagu...........................................................    1      1      dv9
...............uagauacucccuauauccaguC..........................................................    12     1      dv9
```

dvi-miR-975-5p

dvi-miR-975-3p

uauggucuuugauuuuagauacucccuauauccaguaugugugccacuuauugaaauacaagauguauggggugucugcaaucacaaaccacgua

...............uagauacucccuauauccaguaA...	1	1	dv9
.................gauacucccuauauccagu...	1	0	dv9
.................gauacucccuauauccUgu...	1	1	dv9
..................auacucccuauauccaguC..	1	1	dv9
....uacaagauguauggggugucu..................	1	0	dv9
....Cacaagauguauggggugucu..................	1	1	dv9
....uacaagauguauggggugucug.................	1	0	dv9
....Cacaagauguauggggugucugc................	1	1	dv9
....uacaagauguauggggugucuCc................	1	1	dv9
....acaagauguauggggGgu....................	1	1	dv9
....acaagauguauggggugu....................	21	0	dv9
....acaagauguauggCguguc...................	1	1	dv9
....acaagauguaugggguguc...................	88	0	dv9
....acaagauguaugAgguguc...................	1	1	dv9
....acaagauguaugggguguA...................	2	1	dv9
....acaagauguaugggguguU...................	1	1	dv9
....acaagaugCaugggguguc...................	1	1	dv9
....acaagauguaugggguUucu..................	3	1	dv9
....aUaagauguauggggugucu..................	4	1	dv9
....acaagauguauggCgugucu..................	2	1	dv9
....acaagauguaugggguguAu..................	1	1	dv9
....acaagauguaGggggugucu..................	1	1	dv9
....acaagauguauCgggugucu..................	1	1	dv9
....acaagauguauAgggugucu..................	3	1	dv9
....acaagauguauggggugucC..................	181	1	dv9
....acaagauguauggUgugucu..................	7	1	dv9
....Ucaagauguauggggugucu..................	1	1	dv9
....acaagauguaugUggugucu..................	4	1	dv9
....acaagCuguauggggugucu..................	1	1	dv9
....acaagauguauggggAgucu..................	1	1	dv9
....acaagaugCauggggugucu..................	4	1	dv9
....acaagauguauggggCgucu..................	11	1	dv9
....acaagauguGuggggugucu..................	1	1	dv9
....acaagauguaCggggugucu..................	1	1	dv9
....acaagauguauggggugucu..................	2970	0	dv9
....acaagUuguauggggugucu..................	1	1	dv9
....acaagauguaugggguCucu..................	2	1	dv9
....acaagauUuauggggugucu..................	4	1	dv9
....acaagauguauUgggugucu..................	1	1	dv9
....Ccaagauguauggggugucu..................	11	1	dv9
....acaagauguaugggUugucu..................	6	1	dv9
....Gcaagauguauggggugucu..................	3	1	dv9
....acaagauguaugggguguUu..................	1	1	dv9
....acaagauguaugggguAucu..................	3	1	dv9
....acaagauguaugAggugucu..................	1	1	dv9
....acaaAauguauggggugucu..................	1	1	dv9
....acaCgauguauggggugucu..................	2	1	dv9
....acaagauguaugggCugucu..................	1	1	dv9
....acGagauguauggggugucu..................	1	1	dv9
....acaagauguaugCggugucu..................	5	1	dv9
....acaagauguauggAgugucu..................	3	1	dv9
....aAaagauguauggggugucu..................	1	1	dv9
....acaagGuguauggggugucu..................	7	1	dv9
....acaagauguaugggAugucu..................	2	1	dv9
....acaagauguauggggugucA..................	1	1	dv9
....acaagauguauggggugCcu..................	9	1	dv9
....acaagaCguauggggugucu..................	3	1	dv9
....acaGgauguauggggugucu..................	4	1	dv9
....acaagauguauggggGgucu..................	2	1	dv9
....acaagauguGuggggugucug.................	6	1	dv9
....Ccaagauguauggggugucug.................	10	1	dv9
....acaagauguauggggGgucug.................	4	1	dv9
....acaagauguauggggugucCg.................	3	1	dv9
....acGagauguauggggugucug.................	4	1	dv9
....acaagauguauggggugGcug.................	1	1	dv9
....acaagauguaugggguguAug.................	2	1	dv9
....acaagauguaugggUugucug.................	11	1	dv9
....acaagauguauggggugucug.................	4186	0	dv9
....acaagaCguauggggugucug.................	2	1	dv9

dvi-miR-975-5p

dvi-miR-975-3p

uauggucuuugauuuuagauacucccuauauccaguaugugugccacuuauugaaauacaagauguauggggugucugcaaucacaaaccacgua

....acaagauguauggggAgucug.................	6	1	dv9
....acaagauguauCgggugucug.................	2	1	dv9
....acaagauguaugggguAucug.................	2	1	dv9
....acaaCauguauggggugucug.................	2	1	dv9
....acaagauguauggggugucuC.................	70	1	dv9
....acaaUauguauggggugucug.................	2	1	dv9
....acaagauguauggggugucAg.................	1	1	dv9
....acaagauguauggggugucuA.................	16	1	dv9
....acaagauguauggCgugucug.................	1	1	dv9
....aAaagauguauggggugucug.................	3	1	dv9
....acaagauguaAggggugucug.................	1	1	dv9
....acaagauguaugggguUucug.................	3	1	dv9
....acaagauguaugAggugucug.................	2	1	dv9
....acCagauguauggggugucug.................	1	1	dv9
....acaagGuguauggggugucug.................	6	1	dv9
....aUaagauguauggggugucug.................	5	1	dv9
....acaagauguauUgggugucug.................	2	1	dv9
....acaagaugGauggggugucug.................	1	1	dv9
....acaagauguauggUgugucug.................	5	1	dv9
....acaagauguauggggCgucug.................	8	1	dv9
....Gcaagauguauggggugucug.................	5	1	dv9
....acaagauCuauggggugucug.................	3	1	dv9
....acaagauguauggggugCcug.................	10	1	dv9
....acaagauguaugggCugucug.................	3	1	dv9
....acaagaugCauggggugucug.................	4	1	dv9
....acaagauguaugCggugucug.................	1	1	dv9
....acaagauguaCggggugucug.................	3	1	dv9
....acaagauguauggggugucuU.................	47	1	dv9
....acaagauguCuggggugucug.................	1	1	dv9
....acaagauUuauggggugucug.................	3	1	dv9
....acaagauguaugUggugucug.................	6	1	dv9
....acaagCuguauggggugucug.................	2	1	dv9
....acaagauguauAgggugucug.................	1	1	dv9
....acaagauguaugggAugucug.................	1	1	dv9
....acaagauguaugggguguUug.................	3	1	dv9
....acaUgauguauggggugucug.................	2	1	dv9
....acaagauguaugggguCucug.................	15	1	dv9
....acaagaGguauggggugucug.................	1	1	dv9
....acaGgauguauggggugucug.................	8	1	dv9
....acaagauAuauggggugucug.................	1	1	dv9
....acaagauguaugggguguGug.................	1	1	dv9
....acaagauguauggCgugucugc................	1	1	dv9
....Ccaagauguauggggugucugc................	3	1	dv9
....acaagauAuauggggugucugc................	1	1	dv9
....acaagauguaugggguAucugc................	1	1	dv9
....acaagauguauggggugucuAc................	6	1	dv9
....acaagauguaugCggugucugc................	1	1	dv9
....acaagauguaCggggugucugc................	2	1	dv9
....acaagauguauggggugucuUc................	32	1	dv9
....acaagauguauggggugucugG................	2	1	dv9
....acaagauguauggAgugucugc................	1	1	dv9
....acaagauguauUgggugucugc................	1	1	dv9
....acaagCuguauggggugucugc................	2	1	dv9
....acaagauguGuggggugucugc................	5	1	dv9
....aUaagauguauggggugucugc................	3	1	dv9
....acaagauguaugggguguUugc................	1	1	dv9
....acaagauguaugggCugucugc................	1	1	dv9
....acaagauUuauggggugucugc................	1	1	dv9
....acaagauguauggggugucugc................	1976	0	dv9
....acaagauguauggggugucGgc................	1	1	dv9
....acaagauguaugggguUucugc................	4	1	dv9
....acaagauguaugUggugucugc................	3	1	dv9
....acaagaugGauggggugucugc................	2	1	dv9
....acaagGuguauggggugucugc................	1	1	dv9
....acaagauguauggggGgucugc................	3	1	dv9
....acaagauCuauggggugucugc................	2	1	dv9
....acaagauguUuggggugucugc................	2	1	dv9
....acGagauguauggggugucugc................	2	1	dv9
....acaagaCguauggggugucugc................	1	1	dv9

dvi-miR-975-5p

dvi-miR-975-3p

uauggucuuugauuuuagauacucccuauauccaguaugugugccacuuauugaaauacaagauguauggggugucugcaaucacaaaccacgua

....acaagauguauggggCgucugc................	6	1	dv9
....acaagauguauggggugucuCc................	36	1	dv9
....Gcaagauguauggggugucugc................	1	1	dv9
....acaagauguauggggugucugA................	51	1	dv9
....acaagauguaugggguCucugc................	7	1	dv9
....acaagauguaAggggugucugc................	1	1	dv9
....acaGgauguauggggugucugc................	5	1	dv9
....acaagauguauggggugAcugc................	3	1	dv9
....acaagauguauggggugucCgc................	3	1	dv9
....acaagaugCauggggugucugc................	3	1	dv9
....acaagauguaugAggugucugc................	1	1	dv9
....acaagauguCuggggugucugc................	1	1	dv9
....acaagauguaugggUugucugc................	3	1	dv9
....acaaUauguauggggugucugc................	4	1	dv9
....acaagauguauggggugucugU................	428	1	dv9
....acaagauguauggggugCcugc................	2	1	dv9
....acaagauguauggggAgucugc................	1	1	dv9
....acaagauguauggUgugucugc................	2	1	dv9
....acaagauguauggggugucugcU...............	38	1	dv9
....acaagauguauggggugucuCca...............	1	1	dv9
....acaGgauguauggggugucugca...............	1	1	dv9
....acaagauguauggggugucugAa...............	1	1	dv9
....acaagauguauggggugucugUa...............	1	1	dv9
....acaagauguauggggugucugca...............	15	0	dv9
....acaagauguauggggugucugcG...............	3	1	dv9
....acaagauguauggggugucCgca...............	1	1	dv9
....acaagauguauggggugucugcC...............	137	1	dv9
....caagauguaugggguguc...................	16	0	dv9
....caagauguauggggugucu..................	62	0	dv9
....Aaagauguauggggugucu..................	6	1	dv9
....caagauguauggggAgucu..................	1	1	dv9
....caagauguauggggCgucu..................	1	1	dv9
....caagauguauggggugucC..................	5	1	dv9
....caagauguaugggguUucu..................	1	1	dv9
....caagauguauggggugucug.................	87	0	dv9
....caagauAuauggggugucug.................	1	1	dv9
....caagauguauggggugucuU.................	4	1	dv9
....caagauguaugggUugucug.................	1	1	dv9
....caagauguauggggugucuC.................	6	1	dv9
....Aaagauguauggggugucug.................	9	1	dv9
....caagauguaugggguUucug.................	1	1	dv9
....caagauguauggggugucugc................	189	0	dv9
....caagauguauggggCgucugc................	1	1	dv9
....caagauguauggggAgucugc................	1	1	dv9
....Gaagauguauggggugucugc................	1	1	dv9
....caagaCguauggggugucugc................	1	1	dv9
....caagauguauggggugucugU................	9	1	dv9
....Aaagauguauggggugucugc................	11	1	dv9
....caGgauguauggggugucugc................	1	1	dv9
....caagauguaugggguAucugc................	1	1	dv9
....caagauguauggggugucuUc................	1	1	dv9
....caagauguauggggugucugA................	1	1	dv9
....caagauguauggggugucCgc................	1	1	dv9
....caagauguauggggugucuCc................	1	1	dv9
....caagauguaugggguguUugc................	1	1	dv9
....caagauguauggggugucugcU...............	2	1	dv9
....caagauguauggggugucugca...............	12	0	dv9
....caagauguauggggugucugcC...............	7	1	dv9
....aagauguauggggugucC..................	1	1	dv9
....aagauguauggggugucu..................	23	0	dv9
....aagauguauggggugucug.................	37	0	dv9
....aagauguauggggugucuC.................	1	1	dv9
....aagauguaugggUugucugc................	1	1	dv9
....aagauguauggggugucugc................	74	0	dv9
....aagauguaugggguguAugc................	2	1	dv9
....aagauguauggggugucugU................	6	1	dv9
....aagauguauggggugucuUc................	2	1	dv9
....aagauguauggUgugucugc................	3	1	dv9
....aagauguauggggugCcugc................	1	1	dv9

```
              dvi-miR-975-5p
                                           dvi-miR-975-3p
uauggucuuugauuuuagauacucccuauauccaguaugugugccacuuauugaaauacaagauguauggggugucugcaaucacaaaccacgua

...........................................................aagauguauggggugucugcC...............    20    1    dv9
...........................................................aCgauguauggggugucugca...............    2     1    dv9
...........................................................aagauguaugggguCucugca...............    1     1    dv9
...........................................................aagauguauggggugucCgca...............    1     1    dv9
...........................................................aagauguauggggugucugcG...............    1     1    dv9
...........................................................aagauguauggggugucugca...............    82    0    dv9
...........................................................aagauguauggggugucugcaC..............    4     1    dv9
...........................................................aagauguauggggugucugcaa..............    62    0    dv9
...........................................................aagauguauggggugucugcaU..............    3     1    dv9
............................................................aCauguauggggugucug.................    1     1    dv9
.............................................................gauguauggggugucugc................    4     0    dv9

.............Cuuagauacucccuauauccagu...........................................................    4     1    dv1
..............uuagauacucccuauaucc..............................................................    1     0    dv1
..............uuagauaAucccuauaucca.............................................................    1     1    dv1
..............uuagauacucccuauaucca.............................................................    1     0    dv1
..............uuagauacucccuauauccCg............................................................    1     1    dv1
..............uuagauacucccuauauccag............................................................    17    0    dv1
..............uuagauacucccuauauccaU............................................................    1     1    dv1
..............uuagauacucccuauaCccagu...........................................................    2     1    dv1
..............uuagauacucccuauCuccagu...........................................................    13    1    dv1
..............uuGgauacucccuauauccagu...........................................................    3     1    dv1
..............uuagauacucccuauauccagu...........................................................    1401  0    dv1
..............uuagauacucccuCuauccagu...........................................................    9     1    dv1
..............Guagauacucccuauauccagu...........................................................    1     1    dv1
..............uuagauacucccuauauccCgu...........................................................    40    1    dv1
..............uuagauaAucccuauauccagu...........................................................    1     1    dv1
..............uuagauacucccuauauccaUu...........................................................    6     1    dv1
..............uuagauUcucccuauauccagu...........................................................    1     1    dv1
..............uuagauacucccuauauccUgu...........................................................    2     1    dv1
..............uuagauacucccuauauccagC...........................................................    2     1    dv1
..............uuagauacucccuauauccagA...........................................................    1     1    dv1
..............uuagauacucccuauGuccagu...........................................................    1     1    dv1
..............uuagCuacucccuauauccagu...........................................................    1     1    dv1
..............uuagauacucccuauaucUagu...........................................................    3     1    dv1
..............uuagauacuUccuauauccagu...........................................................    2     1    dv1
..............uuagauacucccuauauccaCu...........................................................    1     1    dv1
..............uuagauacAcccuauauccagu...........................................................    1     1    dv1
..............uuagauacuccUuauauccagu...........................................................    2     1    dv1
..............uuagauacuccAuauauccagu...........................................................    1     1    dv1
..............uuagUuacucccuauauccagu...........................................................    1     1    dv1
..............uuagauGcucccuauauccagu...........................................................    3     1    dv1
..............uuagauacucccuauauccGgu...........................................................    1     1    dv1
..............uGagauacucccuauauccagu...........................................................    1     1    dv1
..............uuagauacucAcuauauccagu...........................................................    2     1    dv1
..............uuagauacucccuauauccagua..........................................................    1     0    dv1
..............uuagauacucccuauauccaguU..........................................................    6     1    dv1
..............uuagauacucccuauauccaguC..........................................................    327   1    dv1
..............uuagauacucccuauCuccagua..........................................................    1     1    dv1
..............uuagauacucccuauauccaguCu.........................................................    10    1    dv1
...............uagauacucccuauauccagu...........................................................    72    0    dv1
...............uagauacucccuauauccagC...........................................................    1     1    dv1
...............Gagauacucccuauauccagu...........................................................    1     1    dv1
...............uagauacucccuauCuccagu...........................................................    1     1    dv1
...............uagauacucccuauauccaguC..........................................................    6     1    dv1
.................gauacucccuauauccCgu...........................................................    1     1    dv1
...................uacucccuauauccaguC..........................................................    1     1    dv1
........................................................uacaagauguauggggugucu..................    1     0    dv1
........................................................Aacaagauguauggggugucu..................    1     1    dv1
........................................................Cacaagauguauggggugucu..................    2     1    dv1
........................................................Cacaagauguauggggugucug.................    2     1    dv1
.........................................................acaagauguauggggugu....................    25    0    dv1
.........................................................acaagauguGugggguguc...................    1     1    dv1
.........................................................acaUgauguaugggguguc...................    1     1    dv1
.........................................................acaagGuguaugggguguc...................    2     1    dv1
.........................................................acaagauguauggUguguc...................    1     1    dv1
.........................................................acaagauguaugggguguc...................    72    0    dv1
.........................................................acaagauCuauggggugucu..................    1     1    dv1
.........................................................acaagauguaugUggugucu..................    4     1    dv1
```

```
             dvi-miR-975-5p
                                                         dvi-miR-975-3p
uauggucuuugauuuuagauacucccuauauccaguaugugugccacuuauugaaauacaagauguauggggugucugcaaucacaaaccacgua

.........................................................acaagauguaugggUugucu..................   3      1     dv1
.........................................................acaagCuguauggggugucu..................   1      1     dv1
.........................................................Gcaagauguauggggugucu..................   3      1     dv1
.........................................................acaagauguauggggCgucu..................   12     1     dv1
.........................................................acaagauguaCggggugucu..................   1      1     dv1
.........................................................acaCgauguauggggugucu..................   2      1     dv1
.........................................................acaagauguauggCgugucu..................   1      1     dv1
.........................................................acaagauguauggggugucA..................   2      1     dv1
.........................................................acaaCauguauggggugucu..................   1      1     dv1
.........................................................acaaUauguauggggugucu..................   1      1     dv1
.........................................................acaagauguauCgggugucu..................   2      1     dv1
.........................................................acaagauguauggggugucC..................   129    1     dv1
.........................................................acaagauguauAgggugucu..................   1      1     dv1
.........................................................aUaagauguauggggugucu..................   3      1     dv1
.........................................................acaagauguauggggGgucu..................   4      1     dv1
.........................................................aAaagauguauggggugucu..................   2      1     dv1
.........................................................acaagauguauggggugugAu..................   1      1     dv1
.........................................................acaagauUuauggggugucu..................   1      1     dv1
.........................................................acaagaCguauggggugucu..................   1      1     dv1
.........................................................acCagauguauggggugucu..................   2      1     dv1
.........................................................acaagaugCauggggugucu..................   2      1     dv1
.........................................................acaagauguauggAgugucu..................   2      1     dv1
.........................................................acaagaugGauggggugucu..................   2      1     dv1
.........................................................acaagauguCuggggugucu..................   1      1     dv1
.........................................................acaGgauguauggggugucu..................   4      1     dv1
.........................................................acGagauguauggggugucu..................   2      1     dv1
.........................................................acaagauguaugCggugucu..................   3      1     dv1
.........................................................acaagauguaugggguUucu..................   4      1     dv1
.........................................................acaagauguauggggAgucu..................   3      1     dv1
.........................................................acaagauguauggUgugucu..................   7      1     dv1
.........................................................acaagauAuauggggugucu..................   1      1     dv1
.........................................................acaagauguGuggggugucu..................   2      1     dv1
.........................................................acaagauguauggggugucu..................   2002   0     dv1
.........................................................acaagGuguauggggugucu..................   3      1     dv1
.........................................................acaagauguauggggGgucug.................   3      1     dv1
.........................................................acaagauguaugCggugucug.................   1      1     dv1
.........................................................acaagCuguauggggugucug.................   2      1     dv1
.........................................................acaagauUuauggggugucug.................   3      1     dv1
.........................................................acaagauguaugggAugucug.................   3      1     dv1
.........................................................acaagaugCauggggugucug.................   1      1     dv1
.........................................................acaagauguauggggAgucug.................   4      1     dv1
.........................................................acGagauguauggggugucug.................   1      1     dv1
.........................................................aAaagauguauggggugucug.................   1      1     dv1
.........................................................acaagauguaugggguAucug.................   1      1     dv1
.........................................................acaagauguauggAgugucug.................   1      1     dv1
.........................................................acaagauguauAgggugucug.................   3      1     dv1
.........................................................acaagauAuauggggugucug.................   1      1     dv1
.........................................................acaagauguUuggggugucug.................   2      1     dv1
.........................................................acaagauguauggggugugUug.................   1      1     dv1
.........................................................acaGgauguauggggugucug.................   5      1     dv1
.........................................................acaagaCguauggggugucug.................   2      1     dv1
.........................................................acaaUauguauggggugucug.................   2      1     dv1
.........................................................acaagaGguauggggugucug.................   1      1     dv1
.........................................................acaUgauguauggggugucug.................   1      1     dv1
.........................................................acaagauguauggggugCcug.................   3      1     dv1
.........................................................acaagauguaugggguUucug.................   3      1     dv1
.........................................................acaagauguauggggugucCg.................   5      1     dv1
.........................................................acaagauguauggggugucug.................   2684   0     dv1
.........................................................acaagauguauggggugucuU.................   14     1     dv1
.........................................................acaagauguauggUgugucug.................   4      1     dv1
.........................................................acaagauguauggggugucGg.................   1      1     dv1
.........................................................acaagauguaugUggugucug.................   3      1     dv1
.........................................................acaagauguauggggugucuC.................   18     1     dv1
.........................................................acCagauguauggggugucug.................   2      1     dv1
.........................................................Gcaagauguauggggugucug.................   7      1     dv1
.........................................................acaagauguaCggggugucug.................   4      1     dv1
.........................................................acaagauguaugggUugucug.................   5      1     dv1
.........................................................acaagGuguauggggugucug.................   4      1     dv1
.........................................................acaagauguGuggggugucug.................   3      1     dv1
```

dvi-miR-975-5p

dvi-miR-975-3p

uauggucuuugauuuuagauacucccuauauccaguaugugugccacuuauugaaauacaagauguauggggugucugcaaucacaaaccacgua

....Ucaagauguauggggugucug.................	1	1	dv1
....acaagauguauggggugGcug.................	2	1	dv1
....acaagauguauggggugAcug.................	1	1	dv1
....acaagauguauggggCgucug.................	10	1	dv1
....acaagauguaugggguCucug.................	1	1	dv1
....acaagauguaugggCugucug.................	2	1	dv1
....Ccaagauguauggggugucug.................	1	1	dv1
....aUaagauguauggggugucug.................	4	1	dv1
....acaagauguCuggggugucug.................	1	1	dv1
....acaagauguaugUggugucugc................	4	1	dv1
....acaagGuguauggggugucugc................	1	1	dv1
....acaagauguaugggCugucugc................	1	1	dv1
....acaagauguauggggugucuUc................	24	1	dv1
....acaagauguauggggugCcugc................	3	1	dv1
....acaagauguaugggguCucugc................	2	1	dv1
....acaagauguauggggugucuCc................	9	1	dv1
....acGagauguauggggugucugc................	1	1	dv1
....acaagauguauggggugucugA................	22	1	dv1
....acaagauguauCgggugucugc................	2	1	dv1
....acaagauguauggggugucugc................	1249	0	dv1
....acaagauguauggggugGcugc................	1	1	dv1
....acaagauguaugggguAucugc................	1	1	dv1
....acaagauCuauggggugucugc................	1	1	dv1
....acaagauAuauggggugucugc................	1	1	dv1
....acaagauguaugggUugucugc................	2	1	dv1
....acaagauguGuggggugucugc................	1	1	dv1
....aAaagauguauggggugucugc................	1	1	dv1
....acaagauguauggggCgucugc................	3	1	dv1
....acaUgauguauggggugucugc................	1	1	dv1
....acaagaugCauggggugucugc................	1	1	dv1
....acaagauguaugAggugucugc................	1	1	dv1
....acaagauguauAgggugucugc................	1	1	dv1
....acaagauguauggggAgucugc................	1	1	dv1
....acCagauguauggggugucugc................	1	1	dv1
....acaagaCguauggggugucugc................	1	1	dv1
....acaCgauguauggggugucugc................	1	1	dv1
....acaagauguauggUgugucugc................	2	1	dv1
....acaagCuguauggggugucugc................	1	1	dv1
....acaagauguaugggguUucugc................	2	1	dv1
....acaagauguauggCgugucugc................	2	1	dv1
....acaagauUuauggggugucugc................	2	1	dv1
....acaagauguauggggGgucugc................	1	1	dv1
....acaagauguauggAgugucugc................	2	1	dv1
....acaagauguauggggugucugU................	284	1	dv1
....acaagauguauggggugucugcU...............	13	1	dv1
....acaagauguauggggugucugUa...............	3	1	dv1
....acaagauguauggggugucugca...............	3	0	dv1
....acaagauguauggggugucugcC...............	51	1	dv1
....acaagauguauggggugucugUaa..............	1	1	dv1
....caagauguaugggguguc...................	9	0	dv1
....Aaagauguaugggguguc...................	1	1	dv1
....caagauguauggggugucC..................	7	1	dv1
....caagaugGauggggugucu..................	1	1	dv1
....caagauguauggggugucu..................	38	0	dv1
....Aaagauguauggggugucu..................	1	1	dv1
....caagauguauggggugucug.................	37	0	dv1
....caagCuguauggggugucug.................	1	1	dv1
....Aaagauguauggggugucug.................	5	1	dv1
....caagauguauggggugucuU.................	5	1	dv1
....caagauguaugggguAucugc................	1	1	dv1
....caagauguauggggugucuUc................	3	1	dv1
....caagauguaugUggugucugc................	1	1	dv1
....caGgauguauggggugucugc................	1	1	dv1
....caagauguauggUgugucugc................	1	1	dv1
....caagauguauggggugucugU................	5	1	dv1
....caagaCguauggggugucugc................	1	1	dv1
....caagauguauggggCgucugc................	1	1	dv1
....caagauguauggggugucugc................	111	0	dv1
....Aaagauguauggggugucugc................	9	1	dv1

dvi-miR-975-5p

dvi-miR-975-3p

uauggucuuugauuuuagauacucccuauauccaguaugugugccacuuauugaaauacaagauguauggggugucugcaaucacaaaccacgua

....caagauguauggCgugucugca...............	1	1	dv1
....caagauguauggggugucugcC...............	1	1	dv1
....caagauguauggggugucugca...............	2	0	dv1
....caagauguauggggugucugcaa..............	1	0	dv1
....aagauguauggggugucu..................	14	0	dv1
....aagauguauggggugucug.................	16	0	dv1
....aagauguauggggugucuUc................	1	1	dv1
....aagauguauggggugucugc................	65	0	dv1
....aagauguauggggugucugcC...............	4	1	dv1
....aagauguauggggugucuUca...............	1	1	dv1
....aagauguauggggugucugca...............	44	0	dv1
....aagauguGuggggugucugca...............	1	1	dv1
....aagauguauggggugucugcU...............	4	1	dv1
....aagauguauggggugucugcaC..............	1	1	dv1
....aagGuguauggggugucugcaa..............	1	1	dv1
....aagauguauggggugucugcaU..............	2	1	dv1
....aagauguauggggugucugcCa..............	1	1	dv1
....aagauguauggggugucugcaa..............	11	0	dv1
....agauguauggggugucug.................	1	0	dv1

miRBase precursor : dvi-mir-976
Total read count : 4717
dvi-miR-976-5p read count : 102
dvi-miR-976-3p read count : 4591
remaining reads : 24

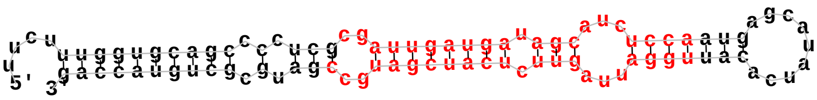

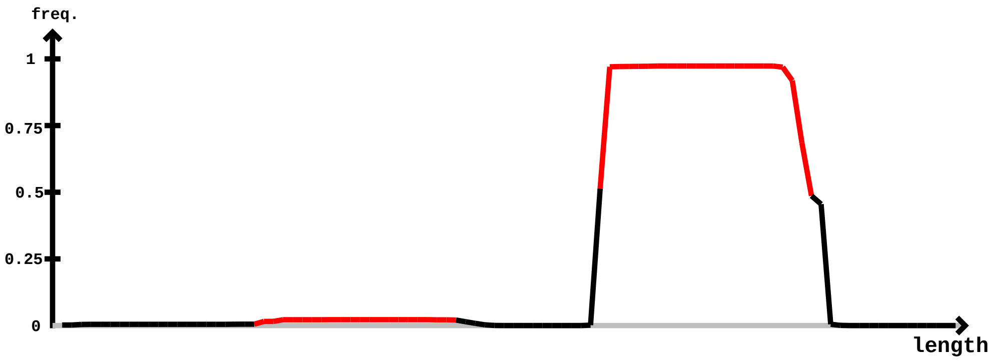


```
                                                                dvi-miR-976-3p
                       dvi-miR-976-5p
5'- uucuuuggugcagccccucgcgauugaugauagcaucuccaaugagcauaucacauuggauuaguucucaucgaugccgaugcgcuguaccag  -3'  exp
    .....((((((((.(.(((..((((((((.(((...(((((((.........)))))))...))).))))))))..))).).)))))))).   reads  mm  sample
    uucuuuggugcagccccucg.........................................................................   4      0   arg
    .ucuuuggugcagccccucg.........................................................................   6      0   arg
    ..cuuuggugcGgccccucg.........................................................................   1      1   arg
    .................ucgcgauugaugauagcaucu.......................................................   1      0   arg
    ....................cgauugaugauagcaucucc.....................................................   2      0   arg
    ....................cgauugaugauagcaucuccaa...................................................   1      0   arg
    ....................cgauugaugauagcaucuccaau..................................................   2      0   arg
    ......................auugaugauagcaucuccaaug.................................................   5      0   arg
    .......................................................uuggauuaguucucaucga...................   3      0   arg
    .......................................................uuggauuaguucucaucgau..................   42     0   arg
    .......................................................uuggauuaguucucaucUaug.................   2      1   arg
    .......................................................uuggauuaguucucaucgGug.................   1      1   arg
    .......................................................uuggauuaguucucaucgaCg.................   1      1   arg
    .......................................................uuggauuaguucucUucgaug.................   1      1   arg
    .......................................................uuggauuaguucucaucgauU.................   1      1   arg
    .......................................................uuggauuaguucucaucgaug.................   72     0   arg
    .......................................................uugAauuaguucucaucgaug.................   1      1   arg
    .......................................................uuggauuaguucucaucgauC.................   7      1   arg
    .......................................................Cuggauuaguucucaucgaug.................   1      1   arg
    .......................................................uuggauuagCucucaucgaugc................   1      1   arg
    .......................................................uuggauCaguucucaucgaugc................   1      1   arg
    .......................................................Cuggauuaguucucaucgaugc................   1      1   arg
    .......................................................uuggauuaguucucaucgaugU................   1      1   arg
    .......................................................uuggauuaguucucaucgaCgc................   1      1   arg
    .......................................................uuggauuaguucucaucgaugc................   48     0   arg
    .......................................................uuggauuaguucucaucgaugcc...............   3      0   arg
    .......................................................uuggauuaguucucaucgaugUc...............   1      1   arg
    .......................................................uuggCuuaguucucaucgaugcc...............   1      1   arg
    .......................................................uuggauuaguucucaucgaugcU...............   1      1   arg
    ........................................................uggauuaguucucaucgaug.................   1      0   arg
    ........................................................uggauuaguucucaucgaugc................   1      0   arg
    ........................................................uggauuaguucucaucgaugcc...............   4      0   arg
    ........................................................uggauuaguucucaucgaugcUg..............   1      1   arg
    ........................................................uggauuaguucucaucgUugccg..............   2      1   arg
```

```
                                                        dvi-miR-976-3p
                    dvi-miR-976-5p
uucuuuggugcagccccucgcgauugaugauagcaucuccaaugagcauaucacauuggauuaguucucaucgaugccgaugcgcuguaccag

........................................................ugCauuaguucucaucgaugccg..............   1     1    arg
........................................................uggauuaguucucaucgaugccg..............   124   0    arg
........................................................uggauuaguucucaucgGugccg..............   1     1    arg
........................................................uggauuaguucucauUgaugccg..............   1     1    arg
........................................................uggauuaguucucaucgaugccA..............   1     1    arg
........................................................uUgauuaguucucaucgaugccg..............   1     1    arg
........................................................uggaGuaguucucaucgaugccg..............   1     1    arg
........................................................uggauuaguucucaucgaugccgU.............   1     1    arg
........................................................uggauuaguucucaucgaugccgUu............   1     1    arg

uucuuuggugcagccccucg.........................................................................   5     0    dv9
.ucuuuggugcagccccucg.........................................................................   4     0    dv9
..cuuuggugcagccccucgcg.......................................................................   1     0    dv9
.................ucgcgauugaugauagcaucu.......................................................   1     0    dv9
..................cgcgauugaugauagcaucucca....................................................   1     0    dv9
....................cgauugaugauagcaucu.......................................................   1     0    dv9
....................cgUuugaugauagcaucucca....................................................   1     1    dv9
....................cgauugaugauagcaucucca....................................................   18    0    dv9
....................Agauugaugauagcaucucca....................................................   1     1    dv9
....................cgauugaugauagcaucuccC....................................................   1     1    dv9
....................cgauugaugauagcaucuccaU...................................................   2     1    dv9
....................cgauuCaugauagcaucuccaa...................................................   1     1    dv9
....................cgauugaugauagcaucuccaa...................................................   13    0    dv9
....................cgauugaugauagcaucuccaau..................................................   8     0    dv9
......................auugaugauagcaucucca....................................................   1     0    dv9
......................auugaugauagcaucuccC....................................................   1     1    dv9
......................auugaugauagcGucuccaa...................................................   1     1    dv9
......................auugaugauagcaucuccaa...................................................   1     0    dv9
......................auugaugauagcaucuccaau..................................................   3     0    dv9
......................auugaugauagcaucuccCau..................................................   1     1    dv9
......................auugaugauagcaucuccaaug.................................................   2     0    dv9
......................auugaugauagcaucuccCaug.................................................   1     1    dv9
......................auugaugauagcaucuccaauC.................................................   1     1    dv9
...........................ugauagcaucuccaauga................................................   1     0    dv9
......................................................Cuuggauuaguucucaucgaugc................   1     1    dv9
......................................................Cuuggauuaguucucaucgaugcc...............   2     1    dv9
.......................................................uuggauuaguucucaucga...................   7     0    dv9
.......................................................uuggauuaguucuAaucgau..................   1     1    dv9
.......................................................uuggauuaguucucaucgCu..................   3     1    dv9
.......................................................uuggauuaguucucaucgUu..................   1     1    dv9
.......................................................uuggauuaguucucaucgau..................   89    0    dv9
.......................................................uuggauuaguucucaucgauC.................   21    1    dv9
.......................................................uuggauuaCuucucaucgaug.................   1     1    dv9
.......................................................uuggauuaguucucCucgaug.................   1     1    dv9
.......................................................uuggauuaguucuAaucgaug.................   1     1    dv9
.......................................................uuggaGuaguucucaucgaug.................   1     1    dv9
.......................................................uuggaCuaguucucaucgaug.................   1     1    dv9
.......................................................uuggauuaguucucaucgGug.................   2     1    dv9
.......................................................uuggGuuaguucucaucgaug.................   1     1    dv9
.......................................................uuggauuaguucucaucUaug.................   11    1    dv9
.......................................................uuggauuaguucucaucgCug.................   17    1    dv9
.......................................................uuggauuaguucucaucgauU.................   4     1    dv9
.......................................................uuggauuaguucucaucgauA.................   3     1    dv9
.......................................................uugAauuaguucucaucgaug.................   1     1    dv9
.......................................................uuAgauuaguucucaucgaug.................   1     1    dv9
.......................................................uuggauuaguucucauAgaug.................   1     1    dv9
.......................................................uuggauCaguucucaucgaug.................   1     1    dv9
.......................................................uuggauuaguucucaucgaug.................   533   0    dv9
.......................................................uuggauuaguucucaucgCugc................   10    1    dv9
.......................................................uuggauuaguucucaucgauUc................   11    1    dv9
.......................................................uuggauuaguucucaucgaugc................   509   0    dv9
.......................................................uuggauuaguucucGucgaugc................   1     1    dv9
.......................................................uuggauuaguucucaucgaugU................   23    1    dv9
.......................................................uuggauuaguucucaCcgaugc................   1     1    dv9
.......................................................uuggauuaguucCcaucgaugc................   4     1    dv9
.......................................................uuggauCaguucucaucgaugc................   1     1    dv9
.......................................................uuggGuuaguucucaucgaugc................   1     1    dv9
.......................................................uuggauuaguucucaucgauCc................   1     1    dv9
.......................................................uCggauuaguucucaucgaugc................   1     1    dv9
```

dvi-miR-976-3p

dvi-miR-976-5p

uucuuuggugcagccccucgcgauugaugauagcaucuccaaugagcauaucacauuggauuaguucucaucgaugccgaugcgcuguaccag

....uuggauuaguucucaucCaugc................	2	1	dv9
....uuggauuaguucucaucUaugc................	2	1	dv9
....uuggauuaguucucaucgauAc................	1	1	dv9
....uugUauuaguucucaucgaugc................	1	1	dv9
....Guggauuaguucucaucgaugc................	1	1	dv9
....uuggauuCguucucaucgaugc................	1	1	dv9
....uuggaCuaguucucaucgaugc................	4	1	dv9
....uuggauuaguCcucaucgaugc................	2	1	dv9
....uuggauuaguucucCucgaugc................	1	1	dv9
....uuggauuaguucucaucgaugcc...............	62	0	dv9
....uuggauuaguucucaucgaugcA...............	4	1	dv9
....uuggauuaguucucaucgaCgcc...............	1	1	dv9
....uuggauuaguucucaucgCugcc...............	2	1	dv9
....uuggauuaguucucaucgaugUc...............	4	1	dv9
....uuggauuaguucucaucgaugcU...............	5	1	dv9
....uuggauuaguucucaucgauCcc...............	1	1	dv9
....uuggauuaguucucCucgaugcc...............	2	1	dv9
....uuggauuaguucucaucgaugccU..............	4	1	dv9
....uuggauuaguucucaucgaugccA..............	1	1	dv9
....uuggauuaguucucaucgaugccg..............	5	0	dv9
....Cuggauuaguucucaucgaugccg..............	7	1	dv9
....uuggauuaguucucaucgaugccC..............	17	1	dv9
....uggauuaguucucaucga...................	1	0	dv9
....uggauuaguucucaucgau..................	1	0	dv9
....uggauuaguucCcaucgaug.................	1	1	dv9
....uggauuaguucucaucgaug.................	2	0	dv9
....uggauuaguucucaucgaugc................	15	0	dv9
....uggauuaguucucaucgaugA................	1	1	dv9
....uggauuaguucucaucgauUcc...............	1	1	dv9
....uggauuaguucucaucgaugcc...............	12	0	dv9
....uggauuaguucucaucgaugcU...............	1	1	dv9
....uggauuaguucucCucgaugccg..............	3	1	dv9
....uggauuaguucucaucgUugccg..............	1	1	dv9
....uggauCaguucucaucgaugccg..............	1	1	dv9
....uggaCuaguucucaucgaugccg..............	3	1	dv9
....uggauuaguucucaCcgaugccg..............	2	1	dv9
....uggauuaguucucaucgaugUcg..............	4	1	dv9
....uggauuaguucucGucgaugccg..............	1	1	dv9
....uggauuaguCcucaucgaugccg..............	3	1	dv9
....uggauuaguucucaucgaugccA..............	1	1	dv9
....uggauuaguAcucaucgaugccg..............	1	1	dv9
....uggauuaguucucaucgaugcAg..............	1	1	dv9
....uggauuagCucucaucgaugccg..............	2	1	dv9
....uggauuaguucucaucgaugccU..............	7	1	dv9
....uggauuaguucAcaucgaugccg..............	1	1	dv9
....uggauuaCuucucaucgaugccg..............	2	1	dv9
....uggGuuaguucucaucgaugccg..............	1	1	dv9
....uggauuaguucucaucgaugAcg..............	3	1	dv9
....uggauuaUuucucaucgaugccg..............	1	1	dv9
....uggauuaguucucaucgCugccg..............	17	1	dv9
....uggauuGguucucaucgaugccg..............	1	1	dv9
....uggauuaguucuAaucgaugccg..............	2	1	dv9
....uggauuaguuUucaucgaugccg..............	5	1	dv9
....uggauuaguucucaGcgaugccg..............	2	1	dv9
....uggauuaguucucaucAaugccg..............	1	1	dv9
....uggauuaguucucaucgauUccg..............	4	1	dv9
....ugUauuaguucucaucgaugccg..............	1	1	dv9
....uggauuaguucucaucgauCccg..............	1	1	dv9
....uggauuaguucucaucgaCgccg..............	1	1	dv9
....uUgauuaguucucaucgaugccg..............	1	1	dv9
....uggauuaguucucaucgGugccg..............	3	1	dv9
....uggCuuaguucucaucgaugccg..............	1	1	dv9
....uggauuaguucuUaucgaugccg..............	7	1	dv9
....uggauuaguucucaucgaugGcg..............	1	1	dv9
....Cggauuaguucucaucgaugccg..............	1	1	dv9
....uggauuaguucucaucgaugccC..............	3	1	dv9
....uggauuaAuucucaucgaugccg..............	1	1	dv9
....uggauuaguucCcaucgaugccg..............	2	1	dv9
....uggauuaguucucauUgaugccg..............	1	1	dv9

```
                                                        dvi-miR-976-3p
                    dvi-miR-976-5p
uucuuuggugcagccccucgcgauugaugauagcaucuccaaugagcauaucacauuggauuaguucucaucgaugccgaugcgcuguaccag

........................................................uggauuaguucucaucgaugccg..............    1276    0    dv9
........................................................uggauuaguucucaucUaugccg..............    1       1    dv9
........................................................ugCauuaguucucaucgaugccg..............    4       1    dv9
........................................................uggauuaguucucaucgaugccgU.............    9       1    dv9
........................................................uggauuaguucucaucgaugccgUu............    1       1    dv9
.........................................................Ugauuaguucucaucgaugccg..............    1       1    dv9
.........................................................ggauuaguucucaucgaugccg..............    2       0    dv9
..........................................................gauuaguucucaucgaugccg..............    2       0    dv9
...........................................................auuaguucucaucgaugccg..............    1       0    dv9
............................................................uuaguucucaucgaugccg..............    1       0    dv9

uucuuuggugcagccccucg.........................................................................    1       0    dv1
....................cgauugaugauagcaucucc.....................................................    2       0    dv1
....................cgauugaugauagcaucucca....................................................    4       0    dv1
....................cgauugaugauagcaucuccaa...................................................    6       0    dv1
....................cgauugaugauagcaucuccCa...................................................    1       1    dv1
....................cgauugaugauagcaucuccCau..................................................    2       1    dv1
....................cgauugaugauagcaucuccaau..................................................    3       0    dv1
.....................gauugaugauagcaucuccaC...................................................    1       1    dv1
......................auugaugauagcaucucca....................................................    1       0    dv1
......................auugaugauagcaucuccCa...................................................    1       1    dv1
......................auugaugauagcaucuccaa...................................................    1       0    dv1
......................auugaugauagcaucuccaau..................................................    7       0    dv1
......................auugaugauagcaucuccaaug.................................................    2       0    dv1
......................auugaugauagcaucuccaaugU................................................    1       1    dv1
......................................................Cuuggauuaguucucaucgaug.................    2       1    dv1
......................................................auuggauuaguucucaucgaugc................    1       0    dv1
......................................................Cuuggauuaguucucaucgaugc................    1       1    dv1
.......................................................uuggauuaguucucaucg....................    1       0    dv1
.......................................................uuggauuaguucucaucga...................    6       0    dv1
.......................................................uuggauuaguucucaucgCu..................    3       1    dv1
.......................................................uuggauuaguucucaucgau..................    95      0    dv1
.......................................................uuggauuaguucucaucgCug.................    16      1    dv1
.......................................................uuggauuaguucucCucgaug.................    1       1    dv1
.......................................................uuggauuaguucucaCcgaug.................    1       1    dv1
.......................................................uuggauuaguucCcaucgaug.................    1       1    dv1
.......................................................uuggaCuaguucucaucgaug.................    1       1    dv1
.......................................................uuggauCaguucucaucgaug.................    1       1    dv1
.......................................................uuggauuaguucucaucUaug.................    8       1    dv1
.......................................................uuggauuaguucucaucgauC.................    10      1    dv1
.......................................................uuggauuaguucucaucgauU.................    4       1    dv1
.......................................................uuggauuaguucuUaucgaug.................    1       1    dv1
.......................................................uuggauuaguucucaucgaug.................    355     0    dv1
.......................................................uuggGuuaguucucaucgaug.................    4       1    dv1
.......................................................uuggauuagCucucaucgaug.................    1       1    dv1
.......................................................uuggauuaguucucaucgaugc................    228     0    dv1
.......................................................uuggauuaguucucaucgCugc................    4       1    dv1
.......................................................uuggauuaguucucaucgaugU................    24      1    dv1
.......................................................uuggauuaguucucCucgaugc................    2       1    dv1
.......................................................uuggauuaguucucaucgauCc................    1       1    dv1
.......................................................uuggauuagCucucaucgaugc................    2       1    dv1
.......................................................uuggauuaguucCcaucgaugc................    1       1    dv1
.......................................................uuggauuaguucucaucgaugA................    1       1    dv1
.......................................................uuggauuGguucucaucgaugc................    1       1    dv1
.......................................................uuggauuUguucucaucgaugc................    1       1    dv1
.......................................................uuggauuaguucucaucgaCgc................    3       1    dv1
.......................................................uuggauuaguucucaucUaugc................    2       1    dv1
.......................................................Cuggauuaguucucaucgaugc................    3       1    dv1
.......................................................uuggauuaguucucaucgauUc................    6       1    dv1
.......................................................uuggauuaguucucaucgaugcU...............    5       1    dv1
.......................................................uuggauuaguucucaucgaugcc...............    24      0    dv1
.......................................................Cuggauuaguucucaucgaugcc...............    1       1    dv1
.......................................................uuggauuaguucucaucgaugUc...............    1       1    dv1
.......................................................uuggauuaguucucaucgaugcA...............    3       1    dv1
.......................................................uuggauuaguucucaucgauUcc...............    1       1    dv1
.......................................................uuggauuaguucucaucgaugccg..............    4       0    dv1
.......................................................uuggauuaguucucaucgaugccC..............    3       1    dv1
........................................................uggauuaguucucaucga...................    2       0    dv1
........................................................uggauuaguucucaucgau..................    3       0    dv1
```

```
                                                        dvi-miR-976-3p
                    dvi-miR-976-5p
uucuuuggugcagccccucgcgauugaugauagcaucuccaaugagcauaucacauuggauuaguucucaucgaugccgaugcgcuguaccag

........................................................uggauuaguucucaucgaug.................    2          0          dv1
........................................................uggauuaguucucaucgaugc................    7          0          dv1
........................................................uggauuaguucucaucgaugU................    1          1          dv1
........................................................uggauuaguucucaucgaugcc...............    6          0          dv1
........................................................uggauuaguucucaucgaugccg..............    535        0          dv1
........................................................uggauuaguCcucaucgaugccg..............    1          1          dv1
........................................................uggauuaguucucUucgaugccg..............    1          1          dv1
........................................................uggauuaguucCcaucgaugccg..............    1          1          dv1
........................................................uggauuaguucucaucgaugUcg..............    1          1          dv1
........................................................uggauuaguucucaucgaugccU..............    4          1          dv1
........................................................uggauuaguucucaucgaCgccg..............    2          1          dv1
........................................................uggauuaguucuGaucgaugccg..............    1          1          dv1
........................................................uggCuuaguucucaucgaugccg..............    1          1          dv1
........................................................uggauuaguucucaucgCugccg..............    3          1          dv1
........................................................uggauuaUuucucaucgaugccg..............    3          1          dv1
........................................................uggauuaguucuAaucgaugccg..............    1          1          dv1
........................................................uggauuaguucucaucgaugcUg..............    1          1          dv1
........................................................uggauuagCucucaucgaugccg..............    3          1          dv1
........................................................ugUauuaguucucaucgaugccg..............    1          1          dv1
........................................................uggauuaguuAucaucgaugccg..............    1          1          dv1
........................................................uggaGuaguucucaucgaugccg..............    1          1          dv1
........................................................uggauuaguucucaucgaugccgU.............    9          1          dv1
........................................................uggauuaguucucaucgaugccgUu............    2          1          dv1
.........................................................ggauuaguucucaucgaugccg..............    1          0          dv1
...........................................................auuaguucucaucgaugccg..............    1          0          dv1
............................................................uuaguucucaucgaugccg..............    1          0          dv1
.............................................................uaguucucaucgaugccg..............    4          0          dv1
```

miRBase precursor : dvi-mir-977
Total read count : 3095
dvi-miR-977-5p read count : 230
dvi-miR-977-3p read count : 2865
remaining reads : 0

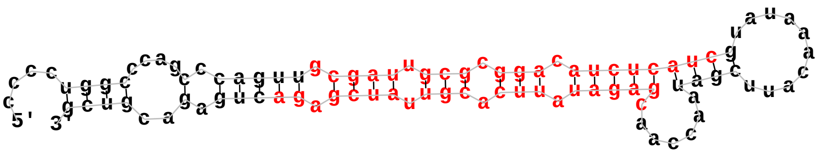

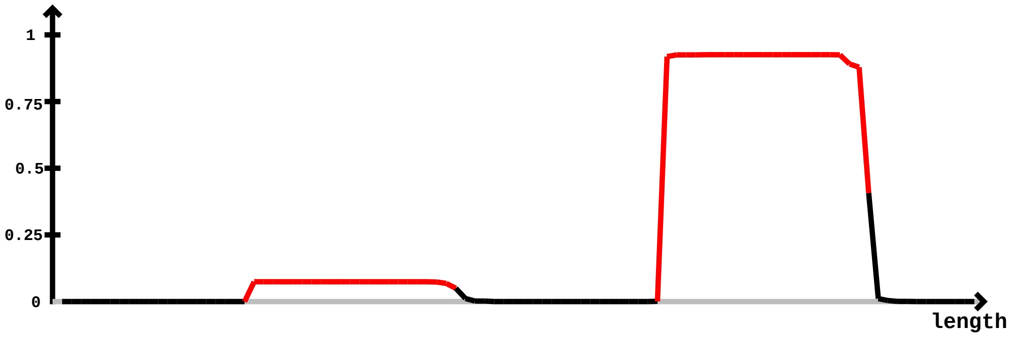


```
                                                                                dvi-miR-977-3p
                                    dvi-miR-977-5p
5'- ccccuggcccagcccaguugcgauugcgcggacaucucaucguauaaacauucgauaaccaacgagauauucacguuaucgagacugagacgucg  -3'   exp
    .....(((...)))(((((.((((.(((.(((.(((((((((..........)))).......))))).))).))).)))).)))))........   reads   mm   sample
    ...................gcgauugcgcggacaucuc.........................................................   2       0    dv1
    ...................gcgauugcgcggacaucuca........................................................   7       0    dv1
    ...................Ucgauugcgcggacaucucau.......................................................   1       1    dv1
    ...................gcgauugcgcggacaucucau.......................................................   23      0    dv1
    ...................gcgauugcgcggacCucucau.......................................................   1       1    dv1
    ...................gcgauugcgcggCcaucucau.......................................................   2       1    dv1
    ...................gcgauugcgcggacaucucCuc......................................................   2       1    dv1
    ...................Ucgauugcgcggacaucucauc......................................................   3       1    dv1
    ...................gcgauugcgcggacaucucauc......................................................   43      0    dv1
    ...................gcgauugcgcggacaucucaucU.....................................................   1       1    dv1
    ...................gcgauugcgcggacaucucaucg.....................................................   4       0    dv1
    ...................gcgauugcgcggacaucucaucA.....................................................   1       1    dv1
    ...................gcgauugcgcggacaucucaucgua...................................................   4       0    dv1
    .............................................................acgagauauucacguuaucgaga...........   1       0    dv1
    ..............................................................cgagauauucacguuauc...............   1       0    dv1
    ..............................................................cgagaCauucacguuaucg..............   1       1    dv1
    ..............................................................cgagauauucacguuauUg..............   1       1    dv1
    ..............................................................Agagauauucacguuaucg..............   5       1    dv1
    ..............................................................cgagauauucacguuaucg..............   46      0    dv1
    ..............................................................cgagauauucacguuaucga.............   20      0    dv1
    ..............................................................cgagauauucacguCaucgag............   1       1    dv1
    ..............................................................cgagauauucGcguuaucgag............   1       1    dv1
    ..............................................................cgagauauucacguuaucgaU............   3       1    dv1
    ..............................................................cgagGuauucacguuaucgag............   1       1    dv1
    ..............................................................cgagauauucacguuaucgag............   514     0    dv1
    ..............................................................cgagauauAcacguuaucgag............   1       1    dv1
    ..............................................................Agagauauucacguuaucgag............   18      1    dv1
    ..............................................................cgagauauucacguuaucgaA............   1       1    dv1
    ..............................................................cgagauauucacguuaucUag............   10      1    dv1
    ..............................................................cgagauauucacgCuaucgag............   3       1    dv1
    ..............................................................cgagauauucacguuaucgCg............   26      1    dv1
    ..............................................................cgagauauucacguuaucgaC............   3       1    dv1
    ..............................................................cgagauauucacguuaucgUg............   2       1    dv1
    ..............................................................cgagauauucacguuCucgag............   5       1    dv1
```

```
                                                              dvi-miR-977-3p
                   dvi-miR-977-5p
ccccuggcccagcccaguugcgauugcgcggacaucucaucguauaaacauucgauaaccaacgagauauucacguuaucgagacugagacgucg

..............................................................Ugagauauucacguuaucgag............    1      1     dv1
..............................................................cgagauauucacguuaucAag............    1      1     dv1
..............................................................cgagauauucacgCuaucgaga...........    1      1     dv1
..............................................................cgagauauucacguuauAgaga...........    1      1     dv1
..............................................................cgagauaCucacguuaucgaga...........    1      1     dv1
..............................................................cgagauauucacguuaucgagC...........    4      1     dv1
..............................................................Agagauauucacguuaucgaga...........    12     1     dv1
..............................................................Ugagauauucacguuaucgaga...........    1      1     dv1
..............................................................cgagauauucacguuaucgaga...........    298    0     dv1
..............................................................cgagauauucacguuaucAaga...........    2      1     dv1
..............................................................cgagGuauucacguuaucgaga...........    1      1     dv1
..............................................................cgagauauucacguuCucgaga...........    1      1     dv1
..............................................................cgaUauauucacguuaucgaga...........    1      1     dv1
..............................................................cUagauauucacguuaucgaga...........    2      1     dv1
..............................................................cgagauauucacguuaucgagU...........    34     1     dv1
..............................................................cgGgauauucacguuaucgaga...........    1      1     dv1
..............................................................cgagauauucacguuaucgCga...........    4      1     dv1
..............................................................cgagauauucacguuaucgaUa...........    4      1     dv1
..............................................................cgagauGuucacguuaucgaga...........    1      1     dv1
..............................................................cgagauauucacguuaucUaga...........    2      1     dv1
..............................................................cgagauauucacguuaucgagaA..........    1      1     dv1
..............................................................cgagauauucacguuaucgagaG..........    1      1     dv1
..............................................................cgagauauucacguuaucgagaU..........    1      1     dv1
..............................................................cgagauauucacguuaucgagaAu.........    2      1     dv1
..............................................................cgagauauucacguuaucgagUcu.........    4      1     dv1
...............................................................gagauauucacguuaucgag............    6      0     dv1
...............................................................gagauauucGcguuaucgag............    1      1     dv1
...............................................................gagauauucacguuaucgaU............    1      1     dv1
...............................................................gagauauucacguuaucgaga...........    3      0     dv1
..................................................................auauucacguuaucgagacuga.......    1      0     dv1

...................gcgauugcgcggacaucuca........................................................    7      0     dv9
...................gcgauugcgcggacaucucC........................................................    1      1     dv9
...................gcAauugcgcggacaucuca........................................................    1      1     dv9
...................gcgauugcgcggacaucucau.......................................................    27     0     dv9
...................Ucgauugcgcggacaucucau.......................................................    2      1     dv9
...................gcgauugcgcggUcaucucauc......................................................    1      1     dv9
...................gcgauugcgcggacaucucauc......................................................    54     0     dv9
...................Ucgauugcgcggacaucucauc......................................................    4      1     dv9
...................gcgauugcgcggacaucucCuc......................................................    2      1     dv9
...................gcgauugcgcggacaucucaucg.....................................................    11     0     dv9
...................Ucgauugcgcggacaucucaucg.....................................................    1      1     dv9
.............................................................acgagauauucacguuaucg..............    1      0     dv9
..............................................................cgagauauucacguuaucg..............    36     0     dv9
..............................................................cgagauauucacgGuaucg..............    1      1     dv9
..............................................................cgagauCuucacguuaucg..............    1      1     dv9
..............................................................Agagauauucacguuaucg..............    4      1     dv9
..............................................................cgaUauauucacguuaucg..............    1      1     dv9
..............................................................cgagauauucacguuaucga.............    11     0     dv9
..............................................................cgagauauucacguuCucga.............    1      1     dv9
..............................................................cgagauauucacguuaucgC.............    1      1     dv9
..............................................................cgagauauucacguuaGcgag............    1      1     dv9
..............................................................cgagCuauucacguuaucgag............    1      1     dv9
..............................................................cgagauauucacguuaucAag............    1      1     dv9
..............................................................cgagauauucCcguuaucgag............    2      1     dv9
..............................................................Agagauauucacguuaucgag............    42     1     dv9
..............................................................cgagauauucacguuaucgaU............    6      1     dv9
..............................................................cgagaCauucacguuaucgag............    1      1     dv9
..............................................................cgagaGauucacguuaucgag............    1      1     dv9
..............................................................cgagauauucacguuaucgaA............    2      1     dv9
..............................................................cgagauGuucacguuaucgag............    1      1     dv9
..............................................................Ugagauauucacguuaucgag............    2      1     dv9
..............................................................cgagauauucacguuaucgGg............    2      1     dv9
..............................................................cgagauauucacguuaucgag............    630    0     dv9
..............................................................cgagauauucacguuaucgaC............    8      1     dv9
..............................................................cgagauaCucacguuaucgag............    1      1     dv9
..............................................................cgagauauucacguuCucgag............    2      1     dv9
..............................................................cgagauauucacguuaucgCg............    42     1     dv9
..............................................................cgagauauucGcguuaucgag............    3      1     dv9
```

```
                                                                   dvi-miR-977-3p
                    dvi-miR-977-5p
ccccuggcccagcccaguugcgauugcgcggacaucucaucguauaaacauucgauaaccaacgagauauucacguuaucgagacugagacgucg

..............................................................cgagauauucacguuaucUag............	11	1	dv9
..............................................................cgagauauucacguuauAgag............	1	1	dv9
..............................................................cgagauCuucacguuaucgag............	2	1	dv9
..............................................................cgagauauucacgCuaucgag............	4	1	dv9
..............................................................cgagauauuAacguuaucgag............	1	1	dv9
..............................................................cUagauauucacguuaucgag............	2	1	dv9
..............................................................cgagauauucacguuGucgag............	1	1	dv9
..............................................................cgagGuauucacguuaucgag............	2	1	dv9
..............................................................cgagauauucacguuaucgagC...........	11	1	dv9
..............................................................cgagauauucacCuuaucgaga...........	1	1	dv9
..............................................................cgagauauucacguuaucUaga...........	2	1	dv9
..............................................................cgagaCauucacguuaucgaga...........	2	1	dv9
..............................................................cgagauauucacguuaucgaAa...........	1	1	dv9
..............................................................cgagauauucacguuaucgaUa...........	2	1	dv9
..............................................................cgagauauucacgCuaucgaga...........	3	1	dv9
..............................................................cgagauauucacguuaucgagU...........	52	1	dv9
..............................................................cgagauauucacguuaucgGga...........	1	1	dv9
..............................................................cgCgauauucacguuaucgaga...........	2	1	dv9
..............................................................cgagauauucaAguuaucgaga...........	1	1	dv9
..............................................................cgagauauucacguuaucAaga...........	1	1	dv9
..............................................................cgagauauucacguuCucgaga...........	2	1	dv9
..............................................................Agagauauucacguuaucgaga...........	28	1	dv9
..............................................................Ugagauauucacguuaucgaga...........	2	1	dv9
..............................................................cgaAauauucacguuaucgaga...........	1	1	dv9
..............................................................cgagauauucacguuaucgaga...........	461	0	dv9
..............................................................cgagauauucacguuaucgCga...........	12	1	dv9
..............................................................cgagauauucacguuaucgagaU..........	6	1	dv9
..............................................................cgagauauucacguuaucgagaA..........	3	1	dv9
..............................................................cgagauauucacguuaucgagUc..........	1	1	dv9
..............................................................cgagauauucacguuaucgagac..........	3	0	dv9
..............................................................cgagauauucacguuaucgagaG..........	3	1	dv9
...............................................................gagauauucacguuaucgag............	4	0	dv9
...............................................................gGgauauucacguuaucgaga...........	1	1	dv9
...............................................................gagauauucacguuaucgaga...........	4	0	dv9
..................................................................auauucacguuaucgagacuga.......	1	0	dv9

...................gcgauugcgcggacaucuU.........................................................	1	1	arg
...................gcgauugcgcggacaucucau.......................................................	1	0	arg
...................gcgauugcgcggacaucucauc......................................................	9	0	arg
...................gcgauugcgcggacaucucaucg.....................................................	12	0	arg
...................gcgauugcgcggacaucucaucgua...................................................	2	0	arg
.............................................................acgagauauucacguuaucgag............	1	0	arg
..............................................................cgagauauucacguuauc...............	1	0	arg
..............................................................cgagauauucacguuaucg..............	8	0	arg
..............................................................cgagauauucacguuaucga.............	5	0	arg
..............................................................cgagauauucacguuaucUag............	1	1	arg
..............................................................cgagauauucacguuaucgCg............	1	1	arg
..............................................................cgagauauucacguuaucgag............	79	0	arg
..............................................................cAagauauucacguuaucgag............	2	1	arg
..............................................................cgagauauucacguuaCcgag............	1	1	arg
..............................................................Ugagauauucacguuaucgag............	1	1	arg
..............................................................cgagauauucacguuaucgaga...........	240	0	arg
..............................................................cgagauauucacguuaucgaUa...........	2	1	arg
..............................................................cgagauauucacguuaCcgaga...........	1	1	arg
..............................................................cgagauauucacguuaucAaga...........	1	1	arg
..............................................................cgagauauucacguuauUgaga...........	1	1	arg
..............................................................cgagauauucacguuaucgagU...........	10	1	arg
..............................................................cgagauauucacguuauAgaga...........	1	1	arg
..............................................................cgagauauucacUuuaucgaga...........	1	1	arg
..............................................................cgagauauucaUguuaucgaga...........	2	1	arg
..............................................................cgagauauucacguuaucgagG...........	1	1	arg
..............................................................cgagauauucacguuaucgagaA..........	3	1	arg
..............................................................cgagauauucacguuaucgagUcu.........	3	1	arg
..............................................................cgagauauucacguuaucgagaUu.........	1	1	arg
```

miRBase precursor : dvi-mir-9695
Total read count : 2797
dvi-miR-9695-5p read count : 385
dvi-miR-9695-3p read count : 2410
remaining reads : 2

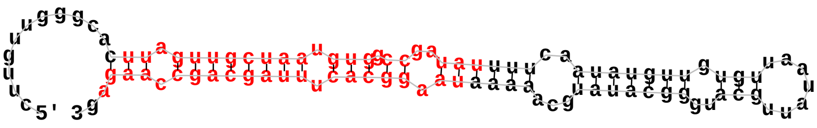

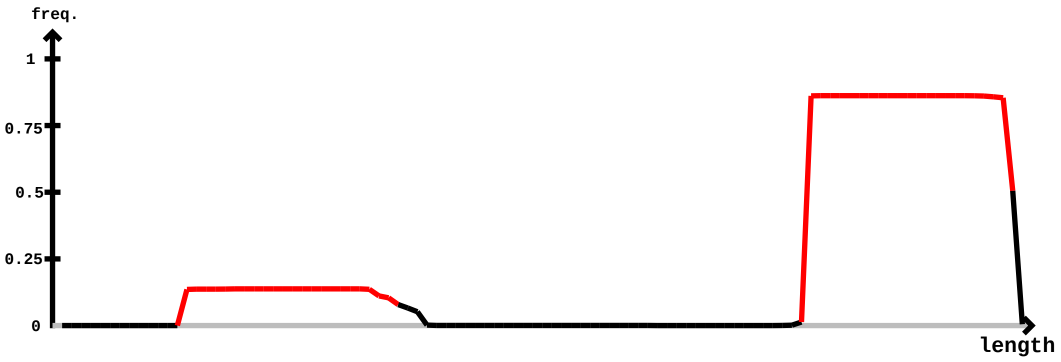

dvi-miR-9695-3p

dvi-miR-9695-5p

5'- cuuguugggcacuuaguugcuaauguggccgauauuuucaauauguuguguuaaauauugcaugggcauaugcaaaaauaaggcacuuuagcagccaagag -3' exp

...........(((.((((((((.(((.((..((((((..(((((((.(((.......)))..)))))))...)))))).))))).)))))))).)))..	reads	mm	sample
............uuaguugcuaauguggccga...	26	0	arg
............uuaguugcuaauUuggccga...	1	1	arg
............uuaguugcuaauguggccgau..	3	0	arg
............uuaguugcuaauguggccgauG...	2	1	arg
............uuaguugcuaauguggccgauC...	3	1	arg
............uuaguugcuaauguggccgaua...	4	0	arg
............uuaguugcuaauguggccgauCu..	1	1	arg
............uuaguugcuaauguggccgauau..	3	0	arg
............uuaguugcuaauguggccgauauC...	2	1	arg
............uuaguCgcuaauguggccgauauu...	1	1	arg
............uuaguugcuaauguggccgauauu...	4	0	arg
............uuaguugcuaauguggccgauauuu..	3	0	arg
............uuaguugcuaauguggccgauauuC..	5	1	arg
.............uaguugcuaauguggccgauauuC..	1	1	arg
....aauaaggcacuuuagcagccaa...	1	0	arg
....uaaggcacuuuagcagcca....	2	0	arg
....uaaggcacuuuagcagccaa...	2	0	arg
....uaaggcacuuuagcagccaag..	59	0	arg
....uaaggcacuuuagcagcUaaga.	1	1	arg
....uaaggcacuuuagcagccaaga.	49	0	arg
....uaaggcacuuuagcagccaagU.	6	1	arg
....uaaggcacuuuagcagccaagaA	1	1	arg
............uuaguugcuaauguggccg..	3	0	dv9
............uuaguugcuaauguggccga...	11	0	dv9
............uuaguugcuaauguggccgau..	7	0	dv9
............uuaguugUuaauguggccgau..	1	1	dv9
............uuaguugcuaauguggccgauC...	25	1	dv9
............uuaguugcuaauguggccgaua...	3	0	dv9
............uuaguugcuaauguggccgauU...	1	1	dv9
............uuaguugcuaauguggccgCua...	1	1	dv9
............uuaguugcuaauguggccgauaC..	1	1	dv9
............uuaguugcuaauguggccgauCu..	6	1	dv9
............uuaguugcuaauguggccgauau..	13	0	dv9

dvi-miR-9695-3p

dvi-miR-9695-5p

cuuguugggcacuuaguugcuaauguggccgauauuuucaauauguuguguuaaauauugcaugggcauaugcaaaaauaaggcacuuuagcagccaagag

............uuaguugcuaauguggccgauaA..	1	1	dv9
............uuaguugcuaauguggccgauauu...	6	0	dv9
............uuaguugcuaauguggccgauCuu...	1	1	dv9
............uuaguugcuaauguggccgauauC...	7	1	dv9
............uuaguugcuaauguggccgCuauu...	1	1	dv9
............uuaguugcuaauguggccgauauuC..	65	1	dv9
............uuaguugcuaauguggccgauauCu..	2	1	dv9
............uuaguugcuaauguggccgauauuCu...	2	1	dv9
.............uaguugcuaauguggccgauauuu..	1	0	dv9
.............uaguugcuaauguggccgauauuC..	1	1	dv9
................uugcuaauguggccgauauuC..	1	1	dv9
.................ugcuaauguggccgauauu...	1	0	dv9
.................ugcuaauguggccgauauuC..	1	1	dv9
....aauaaggcacuuuagcagccaa...	2	0	dv9
....Cuaaggcacuuuagcagccaag..	1	1	dv9
....auaaggcacuuuagcagccaag..	12	0	dv9
....auaaggcacuuuagcagccaaga.	3	0	dv9
....auaaggcacuuuagcagccaCga.	1	1	dv9
....Cuaaggcacuuuagcagccaaga.	3	1	dv9
....auaaggcacuuuagcagccaagU.	1	1	dv9
....uaaggcacuuuagcagcc.....	1	0	dv9
....uaaggcacuuuagcagcca....	1	0	dv9
....uaaggcacuuuagcagccaa...	2	0	dv9
....uaaggcacuuuagcagccaCg..	41	1	dv9
....uaaggcacuuuGgcagccaag..	1	1	dv9
....uaaggcacuuuagcagccaag..	431	0	dv9
....uaaggcacuuuagcagccGag..	1	1	dv9
....uaaggcacuuuagcUgccaag..	1	1	dv9
....uaaggcacuuuagcagAcaag..	1	1	dv9
....uaaggcacCuuagcagccaag..	2	1	dv9
....uaaggcacuuCagcagccaag..	1	1	dv9
....Caaggcacuuuagcagccaag..	1	1	dv9
....uaaggcacuCuagcagccaag..	1	1	dv9
....uaaggcacuuuaUcagccaag..	1	1	dv9
....uaaggcacuuuagcagccaaC..	5	1	dv9
....uaaggcacuuuagcCgccaag..	2	1	dv9
....uaaggcaUuuuagcagccaag..	1	1	dv9
....uaaggcacuuuagcGgccaag..	2	1	dv9
....uaaggcacuuuagcagUcaag..	1	1	dv9
....uaaggcacuuuagcagccaaA..	1	1	dv9
....uaaggcacuuuagcagccCag..	10	1	dv9
....uaaggcacuuuagcagccaaU..	1	1	dv9
....uaaggcacuuuagcagccaaga.	684	0	dv9
....uaaggcacuuuagcagccaaCa.	3	1	dv9
....uaaggcacuGuagcagccaaga.	1	1	dv9
....uaaggcacuuuagcagAcaaga.	2	1	dv9
....uaagAcacuuuagcagccaaga.	1	1	dv9
....uaCggcacuuuagcagccaaga.	1	1	dv9
....uaaggcacuuuagcagUcaaga.	1	1	dv9
....uaaggcacuuuagcagccaCga.	27	1	dv9
....uaaggcacuuuagcagccCaga.	12	1	dv9
....uaaggcacCuuagcagccaaga.	1	1	dv9
....uaaggcacuuuGgcagccaaga.	1	1	dv9
....uaaggcacuuuagcagcUaaga.	1	1	dv9
....uaaggcacuuuagcagccaagG.	2	1	dv9
....uaaUgcacuuuagcagccaaga.	1	1	dv9
....uaaggcacuuuagcaUccaaga.	1	1	dv9
....uaaggcacuuuagcagccGaga.	2	1	dv9
....uaaggcacuuuagcagccaagU.	44	1	dv9
....uaaggcacuuuagcCgccaaga.	4	1	dv9
....uaaggcacuuuagUagccaaga.	1	1	dv9
....Caaggcacuuuagcagccaaga.	2	1	dv9
....uaaggcacuuuaUcagccaaga.	1	1	dv9
....uaaggcacuuuagcagccaaUa.	2	1	dv9
....uaaggcacuuuagcagccaagC.	12	1	dv9
....uaaggcacuuuagcagccaagaC	1	1	dv9
....uaaggcacuuuagcagccaCgag	1	1	dv9
....uaaggcacuuuagcagccaagaA	4	1	dv9
....uaaggcacuuuagcagccaaUag	2	1	dv9

```
                                                                              dvi-miR-9695-3p
            dvi-miR-9695-5p
cuuguugggcacuuaguugcuaauguggccgauauuuucaauauguuguguuaaauauugcaugggcauaugcaaaaauaaggcacuuuagcagccaagag

..............................................................................uaaggcacuuuagcagccaagag   4     0     dv9
...............................................................................aaggcacuuuagcagccaag..   1     0     dv9
...............................................................................aaggcacuuuagcagccaaga.   1     0     dv9

............uuaguugcuaauguggccg......................................................................   1     0     dv1
............uuaguugcuaauUuggccg......................................................................   1     1     dv1
............uuaguugcuaauUuggccga.....................................................................   1     1     dv1
............uuaguugcuaauguggccga.....................................................................   28    0     dv1
............uuaguugcuaauguggccgC.....................................................................   2     1     dv1
............uuaguugcuaauguggccgau....................................................................   9     0     dv1
............uuaguugcuaauguggccgauC...................................................................   24    1     dv1
............uuaguugcuaauguggccgaua...................................................................   3     0     dv1
............uuaguugcuaauguggccgCua...................................................................   1     1     dv1
............uuaguugcuaauguggccgauU...................................................................   4     1     dv1
............uuaguugcuaauguggccgauau..................................................................   10    0     dv1
............uuaguugcuaauguggccgauCu..................................................................   1     1     dv1
............uuagCugcuaauguggccgauauu.................................................................   1     1     dv1
............uuaguugcuaauguggccgauauC.................................................................   7     1     dv1
............uuaguugcuaauguggccgauauu.................................................................   9     0     dv1
............uuaguugcuaauguggccgauauCu................................................................   2     1     dv1
............uuaguugcuaauguggccgauauuC................................................................   55    1     dv1
............uuaguugcuaauguggccgauauuu................................................................   3     0     dv1
............uuaguugcuaauguggccgauauuuC...............................................................   1     1     dv1
............uuaguugcuaauguggccgauauuCu...............................................................   1     1     dv1
....................................uucaauauguuguguuaaauauugca.......................................   1     0     dv1
...........................................................................aaauaaggcacuuuagcagU......   1     1     dv1
.............................................................................auaaggcacuuuagcagccaag..   10    0     dv1
.............................................................................auaaggcacuuuagcagccaCg..   1     1     dv1
.............................................................................Cuaaggcacuuuagcagccaag..   1     1     dv1
..............................................................................uaaggcacuuuagcagcc.....   2     0     dv1
..............................................................................uaaggcacuuuagcagccU....   1     1     dv1
..............................................................................uaaggcacuuuagcagcca....   4     0     dv1
..............................................................................uaaggcGcuuuagcagccaa...   1     1     dv1
..............................................................................uaaggcacuuuagcagccaa...   2     0     dv1
..............................................................................uaaggcacuuuaCcagccaag..   2     1     dv1
..............................................................................uaaCgcacuuuagcagccaag..   1     1     dv1
..............................................................................uaaggcacuuuagcagccaUg..   1     1     dv1
..............................................................................uaGggcacuuuagcagccaag..   1     1     dv1
..............................................................................uaaggcacuuuagcagccaaA..   1     1     dv1
..............................................................................uaaggcacuuuagcagccCag..   6     1     dv1
..............................................................................uaaggcacuuuagcagccaGg..   1     1     dv1
..............................................................................uaaggcacuuuagcagccaag..   343   0     dv1
..............................................................................uaaggcacuuuagcagccGag..   2     1     dv1
..............................................................................uaagCcacuuuagcagccaag..   1     1     dv1
..............................................................................uaaggcacuuuagcagccaCg..   20    1     dv1
..............................................................................uaaggcacuuuagcagAcaag..   1     1     dv1
..............................................................................uaaggcacuCuagcagccaag..   1     1     dv1
..............................................................................uaaggcacuuuagcagccaaU..   2     1     dv1
..............................................................................uaaggcacuuuagcCgccaag..   3     1     dv1
..............................................................................uaaggcacuuuagcagccaaCa.   1     1     dv1
..............................................................................uaaggUacuuuagcagccaaga.   1     1     dv1
..............................................................................uaaggcacuCuagcagccaaga.   1     1     dv1
..............................................................................uaaggcaUuuuagcagccaaga.   1     1     dv1
..............................................................................uaaggcacuuuagcagccGaga.   1     1     dv1
..............................................................................uaaCgcacuuuagcagccaaga.   1     1     dv1
..............................................................................uaaggcacuuCagcagccaaga.   1     1     dv1
..............................................................................uaaggcGcuuuagcagccaaga.   1     1     dv1
..............................................................................Aaaggcacuuuagcagccaaga.   2     1     dv1
..............................................................................uaaggcacuuuagcagccaagC.   5     1     dv1
..............................................................................uaaggcacuuuagcagccaaUa.   1     1     dv1
..............................................................................uaaggcacuuuagAagccaaga.   1     1     dv1
..............................................................................uaaggcacuuuaUcagccaaga.   4     1     dv1
..............................................................................uaagUcacuuuagcagccaaga.   1     1     dv1
..............................................................................uaaggcacuuuagcGgccaaga.   1     1     dv1
..............................................................................Gaaggcacuuuagcagccaaga.   1     1     dv1
..............................................................................uaaggcacuuuagcagccCaga.   13    1     dv1
..............................................................................uaaggcacuuuagcagccaGga.   1     1     dv1
..............................................................................uaaggcacuuuagcCgccaaga.   2     1     dv1
..............................................................................uaGggcacuuuagcagccaaga.   1     1     dv1
```

dvi-miR-9695-3p

dvi-miR-9695-5p

cuuguugggcacuuaguugcuaauguggccgauauuuucaauauguuguguuaauauugcaugggcauaugcaaaaauaaggcacuuuagcagccaagag

....uaaggcacuuuagcagccaaga.	419	0	dv1
....uaaggcacuuuagcagccaagU.	35	1	dv1
....uaaggcacuuuagcagUcaaga.	1	1	dv1
....Caaggcacuuuagcagccaaga.	1	1	dv1
....uaaggcacuuuagcagccaCga.	25	1	dv1
....uaaggcaAuuuagcagccaaga.	1	1	dv1
....uaaggcacCuuagcagccaaga.	1	1	dv1
....uaaggcacuuuagcaUccaaga.	2	1	dv1
....uaaAgcacuuuagcagccaaga.	1	1	dv1
....uaaggcacuuuagcagccaagag	1	0	dv1

miRBase precursor : dvi-mir-9696a
Total read count : 9051
dvi-miR-9696a-5p read count : 3457
dvi-miR-9696a-3p read count : 5591
remaining reads : 3

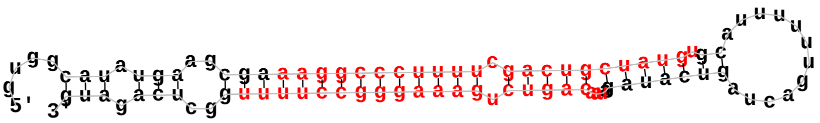

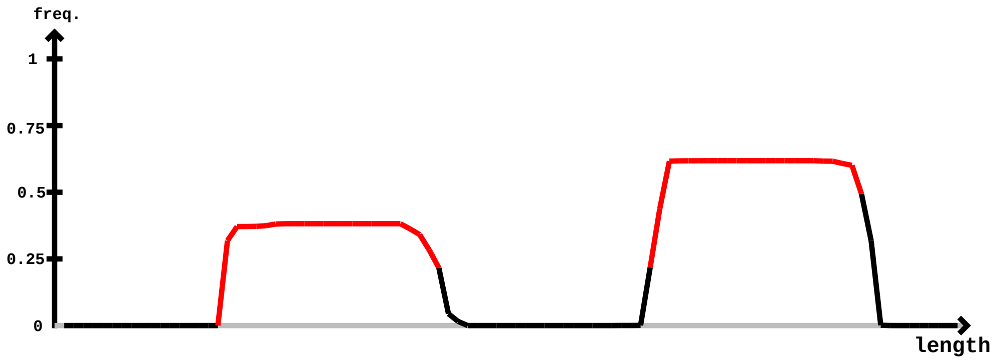

dvi-miR-9696a-5p

dvi-miR-9696a-3p

5'- guggcauaugaagcgaaaggcccuuuucgacugcuaugugcauuuuuugacuagucauagauaacagucugaaagggccuuuuggcucagaug -3' exp

....(((.(((..(((((((((((((.(((((((((.((...........)))))))....))))).))))))))))))))..))).)))	reads	mm	sample
...............Caaggcccuuuucgacugcuaug...	1	1	dv1
................aaggcccuuuucgCcugcu..	2	1	dv1
................aaggcccuuuucgacugcu..	71	0	dv1
................aaggcccuuuucgUcugcu..	1	1	dv1
................aaggccUuuuucgacugcu..	1	1	dv1
................aagCcccuuuucgacugcu..	3	1	dv1
................aaggcccuuuucgacuUcu..	1	1	dv1
................aaggcccuuuucgacugcCa...	1	1	dv1
................aaggcccCuuucgacugcua...	1	1	dv1
................aaggcccuuuucgacugcua...	73	0	dv1
................aaggcccuuuucgCcugcua...	2	1	dv1
................aaggcccuuuucgacugcuU...	1	1	dv1
................aaggcccuuuucgacugcuC...	10	1	dv1
................aaUgcccuuuucgacugcuau..	3	1	dv1
................aaggccUuuuucgacugcuau..	1	1	dv1
................aaggcccuuuucgacugcuCu..	9	1	dv1
................aaggcccuuuucgacugcuau..	205	0	dv1
................aaggcccuuuucUacugcuau..	1	1	dv1
................aaggcccuuuucgGcugcuau..	1	1	dv1
................aaggcccuuuucgacGgcuau..	1	1	dv1
................aagCcccuuuucgacugcuau..	1	1	dv1
................aaggcccuuuucgCcugcuau..	2	1	dv1
................aaggcccuuuucgacugcuauU...	4	1	dv1
................aaggcccuuuucgacugcuaug...	126	0	dv1
................aaggcccuuuucgaUugcuaug...	1	1	dv1
................aaggcccuuuucUacugcuaug...	1	1	dv1
................aaggcccuuuucgacugcuauA...	1	1	dv1
................aaggcccuuuucgCcugcuaug...	1	1	dv1
................aaggcccuuuucgacuUcuaug...	2	1	dv1
................aaggcccuuuucgacugcuauC...	37	1	dv1
................aaggcccuuuucgacugcuCug...	10	1	dv1
................aaggcccuuuucgacugcuCugu..	14	1	dv1
................aaggcccuuuucgacugcuaugC..	2	1	dv1
................aaggcccuuuucAacugcuaugu..	1	1	dv1

dvi-miR-9696a-5p

dvi-miR-9696a-3p

guggcauaugaagcgaaaggcccuuuucgacugcuaugugcauuuuuugacuagucauagauaacagucugaaagggccuuuuggcucagaug

................aaggcccuCuucgacugcuaugu..	1	1	dv1
................aaggcccuuuuUgacugcuaugu..	1	1	dv1
................aaggccUuuuucgacugcuaugu..	4	1	dv1
................aaggcccuuuucgacuCcuaugu..	2	1	dv1
................aGggcccuuuucgacugcuaugu..	2	1	dv1
................aaggcccuuuucgacugAuaugu..	1	1	dv1
................aaggcAcuuuucgacugcuaugu..	1	1	dv1
................aaggcccuuuucgCcugcuaugu..	1	1	dv1
................aaggcccuuuucgacugcuaCgu..	1	1	dv1
................aaggcccCuuucgacugcuaugu..	1	1	dv1
................aaggcccuuuucgacugcuaugA..	1	1	dv1
................aaggcccuuuucgacugcuauCu..	2	1	dv1
................aaggcccuuuucgaUugcuaugu..	1	1	dv1
................aaggcccuuuucgacugcuaugu..	225	0	dv1
................aaggcccuuuucgacugcuauUu..	1	1	dv1
................aaggcccuuuucgacugcuaugG..	1	1	dv1
................aaggcccuuuucgacugcuauguC...	47	1	dv1
................aaggcccuuuucgacugcuauguCc..	30	1	dv1
.................aggcccuuuucgacuCcuau..	2	1	dv1
.................aggcccuuuucgacugcuau..	5	0	dv1
.................aUgcccuuuucgacugcuau..	1	1	dv1
.................aggcccuuuucgacugcuaug...	7	0	dv1
.................aggcccuuuuUgacugcuaugu..	1	1	dv1
.................aggcccuuuucgacugcuCugu..	4	1	dv1
.................aggcccuuuucgacugcuaugu..	76	0	dv1
.................agUcccuuuucgacugcuaugu..	1	1	dv1
.................aggcccuuuucgacugcuauguU...	1	1	dv1
.................aggcccuuuucgacugcuauguC...	20	1	dv1
.................aggcccuuuucgacugcuauguUc..	3	1	dv1
.................aggcccuuuucgacugcuauguCc..	6	1	dv1
...................gcccuuuucgacugcuau..	1	0	dv1
...................gcccuuuucgacugcuaugu..	2	0	dv1
....................cccuuuucgacugcuCug...	1	1	dv1
....................cccuuuucgacugcuaugu..	4	0	dv1
....................cccuuuucgacugcuauguC...	1	1	dv1
.....................ccuuuucgacugcuaugu..	13	0	dv1
.....................ccuuuucgacugcuauguCc..	2	1	dv1
......................cuuuucgacugcuauguC...	1	1	dv1
....uagauaacagucugaaagggcc..............	2	0	dv1
....uagauaacagucugaaagggccu.............	1	0	dv1
....auaacagucugaaagggc...............	2	0	dv1
....auaacagucugaaagggcc..............	1	0	dv1
....auaacagucugaaagggccu.............	32	0	dv1
....auaacagucCgaaagggccuu............	1	1	dv1
....auGacagucugaaagggccuu............	1	1	dv1
....auaacagucugaaagggccuu............	28	0	dv1
....auaacagucugaaagggccuuC...........	5	1	dv1
....auaacagCcugaaagggccuuu...........	1	1	dv1
....auaacagucugaaagggAcuuu...........	1	1	dv1
....auaacagucugaaagUgccuuu...........	1	1	dv1
....auaacagucugaaCgggccuuu...........	1	1	dv1
....auaacagucugaCagggccuuu...........	3	1	dv1
....auaacagucugaaGgggccuuu...........	1	1	dv1
....auaacagucugaaaCggccuuu...........	1	1	dv1
....Cuaacagucugaaagggccuuu...........	2	1	dv1
....auaacagucugaaaUggccuuu...........	1	1	dv1
....auaacagucugaaaggCccuuu...........	1	1	dv1
....auaacagucugaaagggccuuA...........	1	1	dv1
....auaacagucugaaagggcUuuu...........	2	1	dv1
....auaacagucugaaagggccuuu...........	251	0	dv1
....aGaacagucugaaagggccuuu...........	1	1	dv1
....auaacagucugaaaggAccuuuu..........	1	1	dv1
....auaacagucugaaagggccuuCu..........	4	1	dv1
....auaacagucugaaagggccuuuA..........	1	1	dv1
....auaacagucugaaagggccuuuC..........	58	1	dv1
....auaacagucugaaaggCccuuuu..........	1	1	dv1
....auaaAagucugaaagggccuuuu..........	1	1	dv1
....auaacagucugaaagAgccuuuu..........	1	1	dv1
....auaacagucugaaagggcUuuuu..........	1	1	dv1

```
                dvi-miR-9696a-5p
                                                             dvi-miR-9696a-3p
guggcauaugaagcgaaaggcccuuuucgacugcuaugugcauuuuuugacuagucauagauaacagucugaaagggccuuuuggcucagaug

............................................................auaacagucugaaagggUcuuuu..........    1      1      dv1
............................................................auaacagucugaaagggccuuGu..........    1      1      dv1
............................................................auaacagucugaaCgggccuuuu..........    2      1      dv1
............................................................auaacagucugaGagggccuuuu..........    1      1      dv1
............................................................auaacagucuAaaagggccuuuu..........    1      1      dv1
............................................................Cuaacagucugaaagggccuuuu..........    1      1      dv1
............................................................auaacagucugaaaCggccuuuu..........    1      1      dv1
............................................................auaacagucugaaagggccuuAu..........    1      1      dv1
............................................................auaacagucugaaagggccuuuu..........    150    0      dv1
............................................................auaacagucugaaagggccuuuuU.........    1      1      dv1
............................................................auaacagucugaaagggccuuuuC.........    268    1      dv1
.............................................................uaacagucugaaagggcA..............    1      1      dv1
.............................................................uaacagucugaaagggccu.............    2      0      dv1
.............................................................uaacagucugaaagggccuu............    6      0      dv1
.............................................................uaacagucugaaagUgccuuu...........    1      1      dv1
.............................................................uaacaAucugaaagggccuuu...........    1      1      dv1
.............................................................Caacagucugaaagggccuuu...........    1      1      dv1
.............................................................uaacagucugaaagggccuCu...........    1      1      dv1
.............................................................uaacagucugaaagCgccuuu...........    1      1      dv1
.............................................................uaacagucugaaCgggccuuu...........    3      1      dv1
.............................................................uaacagucugaaaCggccuuu...........    1      1      dv1
.............................................................uaacagucugaaagggccuuu...........    179    0      dv1
.............................................................uaacagucuAaaagggccuuu...........    1      1      dv1
.............................................................uaacagucugaaaggAccuuu...........    1      1      dv1
.............................................................uaacagucuUaaagggccuuuu..........    1      1      dv1
.............................................................uaacagucugaaagggccuuCu..........    3      1      dv1
.............................................................uaUcagucugaaagggccuuuu..........    1      1      dv1
.............................................................uaacagucugaaagggccuuuu..........    171    0      dv1
.............................................................uaacagucugaaagggccuuuC..........    55     1      dv1
.............................................................Caacagucugaaagggccuuuu..........    1      1      dv1
.............................................................uaacagucugaaCgggccuuuu..........    1      1      dv1
.............................................................uaacagucugaaagggccuuGu..........    2      1      dv1
.............................................................uaacagucugaaaAggccuuuu..........    1      1      dv1
.............................................................uaacagucugaaagggccuuuuU.........    3      1      dv1
.............................................................uaacagucugaaagggccuuuug.........    1      0      dv1
.............................................................uaacagucugaaagggccuuuuC.........    369    1      dv1
.............................................................uaacagucugaaagggccuuuuUg........    1      1      dv1
..............................................................aacagucugaaagggccu.............    1      0      dv1
..............................................................aacagucugaaagggccuu............    2      0      dv1
..............................................................aacagucugaaagggccGuu...........    1      1      dv1
..............................................................aacagucugaaagggccuuu...........    32     0      dv1
..............................................................aacagucugaaagggccuuuC..........    33     1      dv1
..............................................................aacagGcugaaagggccuuuu..........    1      1      dv1
..............................................................aacagucugaaagggccuuuu..........    113    0      dv1
..............................................................aacagucugaaaUggccuuuu..........    1      1      dv1
..............................................................aacagucugaaagUgccuuuu..........    1      1      dv1
..............................................................aacagucugaaagggUcuuuu..........    1      1      dv1
..............................................................aacagucugaaagggcUuuuu..........    1      1      dv1
..............................................................aacagucugaaagggccGuuu..........    1      1      dv1
..............................................................aacagucugaaGgggccuuuug.........    1      1      dv1
..............................................................aacagucugaaagggccuuuug.........    2      0      dv1
..............................................................aacagucugaaagggccuuuuC.........    538    1      dv1
..............................................................aacagucugaaagggccuuuuA.........    1      1      dv1
..............................................................aacagucugaaagggccuuuuU.........    9      1      dv1
..............................................................aacagucugaaagggccuuuuCg........    4      1      dv1
...............................................................acagucugaaagggccuuuu..........    1      0      dv1
...............................................................acagucugaaagggccuuuuC.........    5      1      dv1
................................................................cagucugaaagggccuuuuC.........    2      1      dv1

................aaggcccuuuucgacugcu..........................................................    21     0      arg
................aaggcccuuuuUgacugcu..........................................................    1      1      arg
................aaggcccuuuucgacugcua.........................................................    15     0      arg
................aaggcccuuuucgCcugcua.........................................................    1      1      arg
................aaggcAcuuuucgacugcua.........................................................    1      1      arg
................aaggcccuuuucgacugcuau........................................................    28     0      arg
................aaggcccuuCucgacugcuau........................................................    1      1      arg
................aaggcccCuuucgacugcuau........................................................    1      1      arg
................aaggcccuuuucgacugcuauC.......................................................    7      1      arg
................aaggccUuuuucgacugcuaug.......................................................    1      1      arg
```

```
                dvi-miR-9696a-5p
                                                             dvi-miR-9696a-3p
guggcauaugaagcgaaaggcccuuuucgacugcuaugugcauuuuuugacuagucauagauaacagucugaaagggccuuuuggcucagaug

................aaggcccuuuucgacCgcuaug.......................................................	1	1	arg
................aaggcccuuuucUacugcuaug.......................................................	1	1	arg
................aaggcccuuuucgacugcuCug.......................................................	2	1	arg
................aaggAccuuuucgacugcuaug.......................................................	1	1	arg
................aaggcccuuuucgacugcuaug.......................................................	66	0	arg
................aaggcccuuuucgacugcuauUu......................................................	5	1	arg
................aaggcccuuuucgacugcuauCu......................................................	1	1	arg
................aaggcccuuuucgacugcuaugC......................................................	2	1	arg
................aaggcccuuuucgacugcuaugu......................................................	439	0	arg
................aaggcccuuuucUacugcuaugu......................................................	1	1	arg
................aagCcccuuuucgacugcuaugu......................................................	3	1	arg
................aaggcccuuuucgaUugcuaugu......................................................	1	1	arg
................aaggcccuuuucgaAugcuaugu......................................................	1	1	arg
................aaggcccuCuucgacugcuaugu......................................................	3	1	arg
................aaggcccuuuucgacugcuGugu......................................................	1	1	arg
................aaggcUcuuuucgacugcuaugu......................................................	1	1	arg
................aaggccUuuuucgacugcuaugu......................................................	1	1	arg
................aaggcccuuuCcgacugcuaugu......................................................	1	1	arg
................aaggcccuuuucgacugcuaGgu......................................................	1	1	arg
................aaggcccuuuucgacugcuauAu......................................................	2	1	arg
................aaggcccuuuucgacuAcuaugu......................................................	1	1	arg
................aaggcccuuuucgacugcuCugu......................................................	2	1	arg
................aaggcccuuuucgacGgcuaugu......................................................	1	1	arg
................aaggcccuuuuUgacugcuaugu......................................................	1	1	arg
................aaggcccuuuucgacugcuauguC.....................................................	53	1	arg
................aaggcccuuuucgacugcuauguU.....................................................	1	1	arg
................aaggcccuuuucgacugcuauguUc....................................................	1	1	arg
................aaggcccuuuucgacugcuauguCc....................................................	8	1	arg
.................aggcccuuuucgacugcuau........................................................	2	0	arg
.................Uggcccuuuucgacugcuaug.......................................................	1	1	arg
.................aggcccuuuucgacugcuaug.......................................................	6	0	arg
.................aggcccuuuucgacugcuaugu......................................................	115	0	arg
.................aggcccuuuucgUcugcuaugu......................................................	1	1	arg
.................agUcccuuuucgacugcuaugu......................................................	1	1	arg
.................aggcccuuuucgacugcuaugC......................................................	1	1	arg
.................aggcccuuuucgacugUuaugu......................................................	1	1	arg
.................aggcccuuuucgacugcuauguU.....................................................	1	1	arg
.................aggcccuuuucgacugcuauguC.....................................................	10	1	arg
.................aggcccuuuucgacugcuauguCc....................................................	4	1	arg
...................gcccuuuucgacugcuaug.......................................................	1	0	arg
....................cccuuuucgacugcuaugu......................................................	8	0	arg
.....................cAuuuucgacugcuaugu......................................................	1	1	arg
.....................Ucuuuucgacugcuaugu......................................................	1	1	arg
.....................ccuuuucgacugcGaugu......................................................	1	1	arg
.....................ccuuuucgacugcuaugu......................................................	27	0	arg
.....................ccuuuucgacugcuauguC.....................................................	1	1	arg
.....................ccuuuucgacugcuauguCc....................................................	1	1	arg
......................cuuuucgacugcuauguC.....................................................	3	1	arg
......................cuuuucgacugcuauguCc....................................................	1	1	arg
.......................uuuucgacugcuauguCc....................................................	1	1	arg
...........................................................Cauaacagucugaaagggccuuu...........	1	1	arg
............................................................auaacagucugaaagggc...............	2	0	arg
............................................................auaacagucugaaagggU...............	1	1	arg
............................................................auaaAagucugaaagggccu.............	1	1	arg
............................................................auaacagucugaaagggccu.............	13	0	arg
............................................................auaacagucugaaagggccuu............	5	0	arg
............................................................auaacagucugaaagggccuuu...........	82	0	arg
............................................................auaacagucugaaaAggccuuu...........	1	1	arg
............................................................auaacagucugaaGgggccuuu...........	1	1	arg
............................................................auaacagucugGaagggccuuuu..........	1	1	arg
............................................................auaacagucugaaagggccuuuC..........	14	1	arg
............................................................auaacagucugaaagggccuuuA..........	1	1	arg
............................................................auCacagucugaaagggccuuuu..........	1	1	arg
............................................................auaacagucugaaUgggccuuuu..........	1	1	arg
............................................................auaacagucugaaagggccuuuu..........	63	0	arg
............................................................auaacagucugaaagggccuuuuC.........	74	1	arg
............................................................auaacagucugaaagggccuuuCg.........	1	1	arg
............................................................auaacagucugaaagggccuuuuU.........	1	1	arg
.............................................................uaacagucugaaagggccu.............	4	0	arg
```

```
                dvi-miR-9696a-5p
                                                             dvi-miR-9696a-3p
guggcauaugaagcgaaaggcccuuuucgacugcuaugugcauuuuuugacuagucauagauaacagucugaaagggccuuuuggcucagaug

.............................................................uaGcagucugaaagggccuu............	1	1	arg
.............................................................uaacagucugGaagggccuuu...........	1	1	arg
.............................................................uaacagucugaaagggccuuu...........	47	0	arg
.............................................................uaacagucCgaaagggccuuu...........	1	1	arg
.............................................................uaacagucugaaaggCccuuu...........	1	1	arg
.............................................................uGacagucugaaagggccuuu...........	1	1	arg
.............................................................uaacagucCgaaagggccuuuu..........	1	1	arg
.............................................................uaacagucugaaagggcAuuuu..........	1	1	arg
.............................................................uaacagucugaaagAgccuuuu..........	1	1	arg
.............................................................uaacagucugaaagggccCuuu..........	1	1	arg
.............................................................uaacagucugaaagggccuuuC..........	13	1	arg
.............................................................uaacagucugaGagggccuuuu..........	1	1	arg
.............................................................uaacagucugaaagCgccuuuu..........	1	1	arg
.............................................................uaacagucugaaagggccuuuu..........	106	0	arg
.............................................................uaacagucugaaagggccuuuuC.........	149	1	arg
.............................................................uaacagucugaaagggccuuuuU.........	4	1	arg
.............................................................uaacagucugaaagggccuuuugA........	1	1	arg
..............................................................aacagucugaaagggccuu............	1	0	arg
..............................................................aacagucuAaaagggccuuu...........	1	1	arg
..............................................................aacagucugaaagggccuuu...........	10	0	arg
..............................................................aacagucugaaagggccuuuu..........	55	0	arg
..............................................................aacagucugaaagggccuCuu..........	1	1	arg
..............................................................aacagucugaaagggcAuuuu..........	1	1	arg
..............................................................aacagucugaaagggccuuuC..........	11	1	arg
..............................................................aacagucugaaaUggccuuuu..........	1	1	arg
..............................................................aacagucugaaagggccuuuuU.........	12	1	arg
..............................................................aacagucugaaagggccuuuuA.........	1	1	arg
..............................................................aacagucugaaagggccuuuuC.........	172	1	arg
..............................................................aacagucugaaagggccuuuuCg........	1	1	arg
...............................................................acagucugaaagggccuuuuC.........	1	1	arg

................aaggcccuuuucgacugcu..........................................................	71	0	dv9
................aaggcccuuuucUacugcu..........................................................	1	1	dv9
................aaggUccuuuucgacugcu..........................................................	1	1	dv9
................aaggcccuCuucgacugcu..........................................................	1	1	dv9
................aGggcccuuuucgacugcu..........................................................	1	1	dv9
................aaggcccuuuucgacuUcua.........................................................	1	1	dv9
................aaggcccuuuucgCcugcua.........................................................	1	1	dv9
................aaggcccuuuucgacugAua.........................................................	1	1	dv9
................aaggcccuuuucgacugcuU.........................................................	2	1	dv9
................aaggcccCuuucgacugcua.........................................................	2	1	dv9
................aaggcccuuuucgacugcua.........................................................	71	0	dv9
................aaggcccuuuucgacugcuC.........................................................	7	1	dv9
................aagCcccuuuucgacugcua.........................................................	2	1	dv9
................aCggcccuuuucgacugcuau........................................................	1	1	dv9
................aaggcccuuuucgGcugcuau........................................................	1	1	dv9
................aaggcccuuCucgacugcuau........................................................	2	1	dv9
................aaggcccuuuucgacugcuCu........................................................	7	1	dv9
................aaggcccuuuucgacugcuau........................................................	239	0	dv9
................aagCcccuuuucgacugcuau........................................................	1	1	dv9
................aaggcccuuuucgacugcuaC........................................................	1	1	dv9
................aaUgcccuuuucgacugcuau........................................................	1	1	dv9
................aaggcccuuuucgacuCcuau........................................................	2	1	dv9
................aaggcccuuuucgCcugcuau........................................................	1	1	dv9
................aaggcccuuuucgacugcuaug.......................................................	215	0	dv9
................aaggcccuuuucgacuUcuaug.......................................................	1	1	dv9
................aaggcccuCuucgacugcuaug.......................................................	1	1	dv9
................aagUcccuuuucgacugcuaug.......................................................	3	1	dv9
................aaggcccuuuucgacugcuCug.......................................................	12	1	dv9
................aGggcccuuuucgacugcuaug.......................................................	1	1	dv9
................aaCgcccuuuucgacugcuaug.......................................................	1	1	dv9
................aaggcccuuuucgaAugcuaug.......................................................	1	1	dv9
................aaggcccuuuucgacugcuauU.......................................................	3	1	dv9
................aaggcccuuuucgacugAuaug.......................................................	1	1	dv9
................aaggcccuuuucgCcugcuaug.......................................................	4	1	dv9
................aaggcccuuuucgacugcuauA.......................................................	2	1	dv9
................aaggcccuuuucgacugcuauC.......................................................	52	1	dv9
................aaggcUcuuuucgacugcuaug.......................................................	1	1	dv9
................aaggcGcuuuucgacugcuaugu......................................................	1	1	dv9
```

```
                dvi-miR-9696a-5p
                                                              dvi-miR-9696a-3p
guggcauaugaagcgaaaggcccuuuucgacugcuaugugcauuuuuugacuagucauagauaacagucugaaagggccuuuuggcucagaug

................aaggcccuuuucgacugAuaugu......................................................   1     1     dv9
................Caggcccuuuucgacugcuaugu......................................................   1     1     dv9
................aaggcccuuuucgUcugcuaugu......................................................   1     1     dv9
................aaggcccuuuucgaAugcuaugu......................................................   1     1     dv9
................aaggcccuuuucgacugcuaugA......................................................   1     1     dv9
................aaggcccuuuucgacugcuaugu......................................................   382   0     dv9
................aaggcUcuuuucgacugcuaugu......................................................   1     1     dv9
................aaggcccuuCucgacugcuaugu......................................................   1     1     dv9
................aaggcccuuuuUgacugcuaugu......................................................   1     1     dv9
................aaggcccuuuucgacugcuauCu......................................................   3     1     dv9
................aagCcccuuuucgacugcuaugu......................................................   1     1     dv9
................aaggcccuuuucgacugcGaugu......................................................   1     1     dv9
................aagUcccuuuucgacugcuaugu......................................................   1     1     dv9
................aaggcccuuuucgacugcuCugu......................................................   14    1     dv9
................aaCgcccuuuucgacugcuaugu......................................................   1     1     dv9
................aaggcccuuuucgacugcuUugu......................................................   1     1     dv9
................aaggcccuuuucUacugcuaugu......................................................   1     1     dv9
................aaggcccCuuucgacugcuaugu......................................................   1     1     dv9
................aaggcccuuuucgacugcCaugu......................................................   1     1     dv9
................aaggcccuuuucCacugcuaugu......................................................   1     1     dv9
................aaggcccuuuucgCcugcuaugu......................................................   4     1     dv9
................aaggcAcuuuucgacugcuaugu......................................................   1     1     dv9
................aaggcccuuuucgacugcuauguU.....................................................   2     1     dv9
................aaggcccuuuucgacugcuauguC.....................................................   91    1     dv9
................aaggcccuuuucgacugcuauguUc....................................................   5     1     dv9
................aaggcccuuuucgacugcuauguCc....................................................   53    1     dv9
.................aggcccuuuucgacugcuau........................................................   3     0     dv9
.................aggcccuuuucgacugcuauC.......................................................   3     1     dv9
.................aggcccuuuucgacugcuaug.......................................................   22    0     dv9
.................Gggcccuuuucgacugcuaug.......................................................   1     1     dv9
.................aggcccuuuucgacugcuCug.......................................................   1     1     dv9
.................aggcccuuuucgacugcuCugu......................................................   5     1     dv9
.................aggcccuuuucgacugcuaugC......................................................   1     1     dv9
.................aggcccuuuucUacugcuaugu......................................................   1     1     dv9
.................Cggcccuuuucgacugcuaugu......................................................   1     1     dv9
.................aggcccuuuucgacugcuauCu......................................................   2     1     dv9
.................agCcccuuuucgacugcuaugu......................................................   4     1     dv9
.................aggcccuuuucgacugcuaugu......................................................   119   0     dv9
.................aggcccuuuucgacugcuauguC.....................................................   27    1     dv9
.................aggcccuuuucgacugcuauguUc....................................................   1     1     dv9
.................aggcccuuuucgacugcuauguCc....................................................   17    1     dv9
...................gcccuuuucgacugcuaug.......................................................   1     0     dv9
...................gcccuuuucgacugcuaugu......................................................   3     0     dv9
....................cccuuuucgacugcuaugu......................................................   3     0     dv9
....................cccuuuucgacugcuauguC.....................................................   1     1     dv9
....................cccuuuucgacugcuauguCc....................................................   1     1     dv9
.....................ccuuuucgacugcuaugu......................................................   8     0     dv9
.....................ccuuuucgacugcuauguCc....................................................   3     1     dv9
......................cuuuucgacugcuauguCc....................................................   5     1     dv9
...........................................................Cauaacagucugaaagggccuuu...........   1     1     dv9
............................................................auaacagucugaaagggc...............   7     0     dv9
............................................................auaacagucugaaagggccu.............   17    0     dv9
............................................................auaacagCcugaaagggccu.............   1     1     dv9
............................................................auaacagucugaaagggccuu............   13    0     dv9
............................................................auaacagCcugaaagggccuu............   2     1     dv9
............................................................auaacagucugaaagggccuCu...........   1     1     dv9
............................................................auaacagucugaaagggccuuC...........   5     1     dv9
............................................................aCaacagucugaaagggccuuu...........   1     1     dv9
............................................................auaacagucugUaagggccuuu...........   1     1     dv9
............................................................auaacagucugaGagggccuuu...........   1     1     dv9
............................................................auaacagucugaaagggccuuu...........   229   0     dv9
............................................................auaacagucugaaagggUcuuu...........   4     1     dv9
............................................................auaacagucugaaagCgccuuu...........   1     1     dv9
............................................................auaacagucugaaagUgccuuu...........   1     1     dv9
............................................................auaacagucugGaagggccuuu...........   1     1     dv9
............................................................auaacagucugaaaUggccuuu...........   2     1     dv9
............................................................auaacagucugaaaggUccuuu...........   3     1     dv9
............................................................auaacagucugaaagggccuuCu..........   1     1     dv9
............................................................auaacagGcugaaagggccuuuu..........   1     1     dv9
```

dvi-miR-9696a-5p

dvi-miR-9696a-3p

guggcauaugaagcgaaaggcccuuuucgacugcuaugugcauuuuuugacuagucauagauaacagucugaaagggccuuuuggcucagaug

....auaacagucugaaagggccGuuu..........	1	1	dv9
....auaacagucugaaagggccuuuC..........	59	1	dv9
....Cuaacagucugaaagggccuuuu..........	1	1	dv9
....auaacagucugaGagggccuuuu..........	1	1	dv9
....auaacagucugaaagUgccuuuu..........	1	1	dv9
....auaacagucugaaagggccuuuA..........	4	1	dv9
....aGaacagucugaaagggccuuuu..........	1	1	dv9
....auaacagucugaaagggccuuuu..........	198	0	dv9
....auaacagucugaaaggCccuuuu..........	1	1	dv9
....auaacagucugaaaUggccuuuu..........	1	1	dv9
....auaacagucugaaaggUccuuuu..........	1	1	dv9
....auaacaCucugaaagggccuuuu..........	1	1	dv9
....aCaacagucugaaagggccuuuu..........	1	1	dv9
....auaacagucugaaCgggccuuuu..........	1	1	dv9
....auaacagucugaaagggccuuuuC.........	299	1	dv9
....auaacagucugaaagggccuuuuA.........	3	1	dv9
....auaacagucugaaagggccuuuuU.........	2	1	dv9
....auaacagucugaaagggccuuuuCg........	1	1	dv9
....uaacagucugaaagggccu.............	1	0	dv9
....uaacagucugaaagggccuu............	2	0	dv9
....uaacagucugaaaggCccuuu...........	1	1	dv9
....uaacagucugaaaUggccuuu...........	1	1	dv9
....uaacagucugaaagggccuuC...........	2	1	dv9
....uaacagucugaaagAgccuuu...........	1	1	dv9
....uaacagucugaaagggccuuu...........	63	0	dv9
....uaacagucugaaagAgccuuuu..........	1	1	dv9
....uaacagucugaaGgggccuuuu..........	2	1	dv9
....uaacagucugaaagggccAuuu..........	1	1	dv9
....uaacagucugaaagggccuuuC..........	70	1	dv9
....uaacagucugaaaggUccuuuu..........	1	1	dv9
....uaacagucugaaagggccCuuu..........	1	1	dv9
....uaacGgucugaaagggccuuuu..........	1	1	dv9
....uaacagucugaaCgggccuuuu..........	1	1	dv9
....uaacagucugaaagggcUuuuu..........	1	1	dv9
....uaacagucugaGagggccuuuu..........	2	1	dv9
....uaacagucugaaagggccuuuA..........	3	1	dv9
....uaacagucugaaagggccuuuu..........	209	0	dv9
....uaacagucugaaagggccuuCu..........	2	1	dv9
....uaacagucugaaagggccuuuuU.........	2	1	dv9
....uaacagucugaaagggccuuuuC.........	483	1	dv9
....uaacagucugaaagggccuuuug.........	1	0	dv9
....uaacagucugaaagggccuuuuCg........	1	1	dv9
....aacagucugaaagggccu.............	3	0	dv9
....aacagucugaaagggccuu............	1	0	dv9
....aUcagucugaaagggccuuu...........	1	1	dv9
....aacagucugaaagggccuuu...........	14	0	dv9
....aacagucugaaagggccuuuu..........	92	0	dv9
....aacagucuAaaagggccuuuu..........	1	1	dv9
....aacagucugaaagggccuuuA..........	2	1	dv9
....aacagucugaaagggccuuuG..........	1	1	dv9
....aacaguUugaaagggccuuuu..........	1	1	dv9
....aacagucugaaaggCccuuuu..........	1	1	dv9
....aacagucugaaagggccuuuC..........	22	1	dv9
....aacagucugaaagggccuuuuA.........	5	1	dv9
....aacagucugaaagggccuuuuU.........	7	1	dv9
....aacagucugaaagggccuuuug.........	3	0	dv9
....aacagucugaaagggccuuuuC.........	446	1	dv9
....aacagucugaaagggccuuuuCg........	1	1	dv9
....aacagucugaaagggccuuuugCc.......	1	1	dv9
....acagucugaaagggccuuuuC.........	2	1	dv9
....cagucugaaagggccuuuugg........	1	0	dv9
....agucugaaagggccuuuuC.........	1	1	dv9
....gucugaaagggccuuuuC.........	1	1	dv9

miRBase precursor : dvi-mir-9697
Total read count : 17403
dvi-miR-9697-5p read count : 13015
dvi-miR-9697-3p read count : 4386
remaining reads : 2

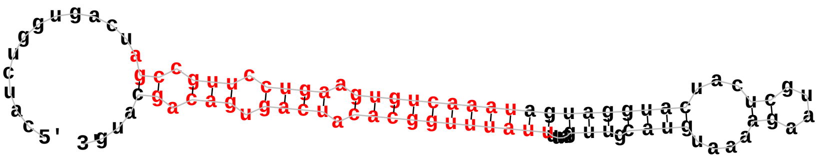

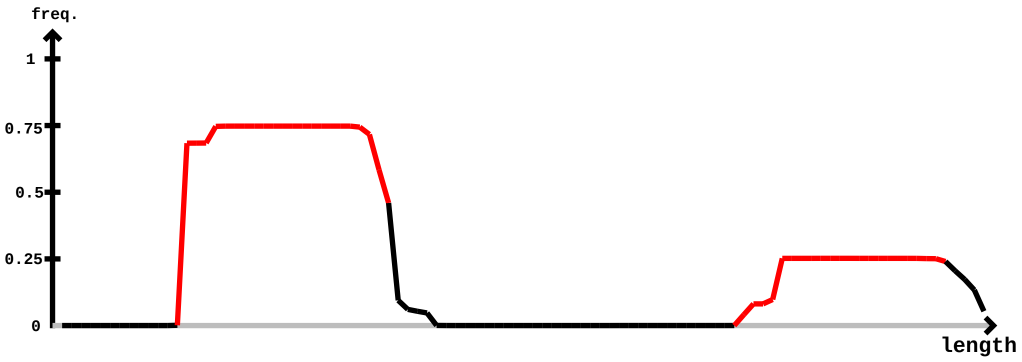

dvi-miR-9697-3p

dvi-miR-9697-5p

```
5'- caucuggugacuagccguuccugaagugucaaauaguagguacuacucguaagaaaauguacguugauuuuuauuuggcacaucagugacagcaug   -3'   exp
    ............((.(((.((((.((((((((((((((((((...((....))....)))).)))....))))))))))).)))).))).))...   reads     mm      sample
    ...........uagccguuccugaaguguca.................................................................   2         0       dv1
    ...........Cagccguuccugaaguguca.................................................................   1         1       dv1
    ...........uagccguuccugaagugucaa................................................................   4         0       dv1
    ...........uagccguuccugaagugucaaa...............................................................   1         0       dv1
    ...........uagccguuccugaCgugucaaa...............................................................   1         1       dv1
    ...........uagccguuccugaagugucaaaua.............................................................   1         0       dv1
    ............agccguucUugaaguguc..................................................................   1         1       dv1
    ............agccguuccugaaguguc..................................................................   9         0       dv1
    ............agccguuccugaCguguca.................................................................   1         1       dv1
    ............agccguuccugaagugCca.................................................................   1         1       dv1
    ............agccUuuccugaaguguca.................................................................   1         1       dv1
    ............agccguuccugaaguguca.................................................................   156       0       dv1
    ............Cgccguuccugaaguguca.................................................................   1         1       dv1
    ............agccgGuccugaaguguca.................................................................   1         1       dv1
    ............agccguuccugaagugucG.................................................................   1         1       dv1
    ............agccguuccugaagugucC.................................................................   13        1       dv1
    ............agccguuccugaaUuguca.................................................................   4         1       dv1
    ............agccguuccugaagugGcaa................................................................   1         1       dv1
    ............agccguuccugaaguUucaa................................................................   1         1       dv1
    ............agccguuccugaagugucaC................................................................   21        1       dv1
    ............agcAguuccugaagugucaa................................................................   1         1       dv1
    ............agccUuuccugaagugucaa................................................................   2         1       dv1
    ............agccguuccugaagGgucaa................................................................   1         1       dv1
    ............agccguuccugaCgugucaa................................................................   8         1       dv1
    ............agccguuccugaagCgucaa................................................................   3         1       dv1
    ............agccguuccugaaAugucaa................................................................   2         1       dv1
    ............agccguucAugaagugucaa................................................................   1         1       dv1
    ............agccguuccugaagugucaG................................................................   1         1       dv1
    ............Ugccguuccugaagugucaa................................................................   1         1       dv1
    ............agccguuccuUaagugucaa................................................................   1         1       dv1
    ............agUcguuccugaagugucaa................................................................   3         1       dv1
    ............agccguuUcugaagugucaa................................................................   1         1       dv1
    ............agccguuccugaUgugucaa................................................................   2         1       dv1
    ............agccguCccugaagugucaa................................................................   1         1       dv1
```

dvi-miR-9697-3p

dvi-miR-9697-5p

caucuggugacuagccguuccugaagugucaaauaguagguacuacucguaagaaaauguacguugauuuuuauuuggcacaucagugacagcaug

............agccCuuccugaagugucaa.. 1 1 dv1
............aCccguuccugaagugucaa.. 1 1 dv1
............agccgCuccugaagugucaa.. 1 1 dv1
............agccguuccugaagugucaa.. 711 0 dv1
............agccguuccugaagugucCa.. 6 1 dv1
............agccgCuccugaagugucaaa... 2 1 dv1
............agccguuccugaagugCcaaa... 1 1 dv1
............agccguuccuCaagugucaaa... 1 1 dv1
............agccguuccugaaUgucaaa.. 2 1 dv1
............agccguuccugaagugucaaa... 541 0 dv1
............agccguuccugaCgugucaaa... 9 1 dv1
............agccguuccugaagugucCaa... 7 1 dv1
............agccguuccugaagugucaaC... 19 1 dv1
............agccguuccugaagugucaaG... 1 1 dv1
............agUcguuccugaagugucaaa... 1 1 dv1
............agccguuccugaagugucaCa... 15 1 dv1
............agccguuccugaagugucaaU... 2 1 dv1
............agccguuccCgaagugucaaa... 1 1 dv1
............agcUguuccugaagugucaaa... 1 1 dv1
............agccCuuccugaagugucaaa... 1 1 dv1
............agccguuAcugaagugucaaa... 1 1 dv1
............agccguuccugaagugGcaaa... 1 1 dv1
............agccguuUcugaagugucaaa... 1 1 dv1
............agccguuccugGagugucaaa... 1 1 dv1
............aUccguuccugaagugucaaa... 3 1 dv1
............agccguuccugaagCgucaaa... 2 1 dv1
............agccguucUugaagugucaaau.. 1 1 dv1
............aCccguuccugaagugucaaau.. 1 1 dv1
............agccguCccugaagugucaaau.. 4 1 dv1
............agccguuccugaagugucUaau.. 1 1 dv1
............agAcguuccugaagugucaaau.. 1 1 dv1
............agccguuccugCagugucaaau.. 2 1 dv1
............agccguuccugaagGgucaaau.. 2 1 dv1
............agccAuuccugaagugucaaau.. 1 1 dv1
............agccguuccuAaagugucaaau.. 1 1 dv1
............agccguuccugaagugucaCau.. 77 1 dv1
............agccguuccugaCgugucaaau.. 16 1 dv1
............agccguuccugaagugucaaCu.. 33 1 dv1
............agccgGuccugaagugucaaau.. 1 1 dv1
............agcUguuccugaagugucaaau.. 3 1 dv1
............Ggccguuccugaagugucaaau.. 2 1 dv1
............agccguuccugaagAgucaaau.. 2 1 dv1
............agccguuccugaaguguUaaau.. 1 1 dv1
............agccguuccugaaAugucaaau.. 1 1 dv1
............agccguuUcugaagugucaaau.. 2 1 dv1
............agccUuuccugaagugucaaau.. 3 1 dv1
............aUccguuccugaagugucaaau.. 3 1 dv1
............agccguuAcugaagugucaaau.. 1 1 dv1
............agccguucAugaagugucaaau.. 2 1 dv1
............agccguuccugGagugucaaau.. 5 1 dv1
............agccguuccugaagCgucaaau.. 8 1 dv1
............agccguuccCgaagugucaaau.. 2 1 dv1
............agccguuccugaaguguAaaau.. 1 1 dv1
............agccCuuccugaagugucaaau.. 2 1 dv1
............agccguuccugaagugucaaaA.. 8 1 dv1
............agccguuccugaaUgucaaau... 1 1 dv1
............agccguuccugaagugucaaau.. 1626 0 dv1
............agccguuccugaGgugucaaau.. 2 1 dv1
............agccguuccugaagugucaUau.. 1 1 dv1
............agccguuccugaagugAcaaau.. 1 1 dv1
............agccguuccugaUgugucaaau.. 2 1 dv1
............agccgCuccugaagugucaaau.. 8 1 dv1
............agccguuccugaagugCcaaau.. 2 1 dv1
............agccguuccugaagugucaaUu.. 2 1 dv1
............agccguuccugaagugucaaaC.. 3 1 dv1
............agccguuccugaagugucCaau.. 20 1 dv1
............agccguuccugaaguUucaaau.. 2 1 dv1
............agccguuccugaagugucaaGu.. 5 1 dv1
............agccguuccugaagugucaGau.. 1 1 dv1

```
                                                                      dvi-miR-9697-3p
            dvi-miR-9697-5p
caucuggugacuagccguuccugaagugucaaauaguagguacuacucguaagaaaauguacguugauuuuuauuuggcacaucagugacagcaug

............Cgccguuccugaagugucaaau..............................................................    2          1          dv1
............agccguuccugaagugucaCaua.............................................................    1          1          dv1
............agccguuccugaagugucaaaua.............................................................    59         0          dv1
............agccguuccugaagugCcaaaua.............................................................    1          1          dv1
............agccguuccugaCgugucaaaua.............................................................    1          1          dv1
............agccguuccugaagugucaaauC.............................................................    38         1          dv1
............agccguuccugaagugucaaauU.............................................................    57         1          dv1
............agUcguuccugaagugucaaaua.............................................................    1          1          dv1
............agccguuccugaagugucaaauaA............................................................    1          1          dv1
..............ccguuccugaagugucCaa...............................................................    1          1          dv1
...............cguuccugaagugucaaa...............................................................    1          0          dv1
...............cguuccugaagugucaaau..............................................................    2          0          dv1
...............cguuccugaagugucaaaua.............................................................    4          0          dv1
...............cguuccugaagugucaCauag............................................................    1          1          dv1
...............cguuccugaagugucaaauag............................................................    25         0          dv1
...............cguuccugaagugucaaCuag............................................................    1          1          dv1
...............cguuccugaaAugucaaauag............................................................    1          1          dv1
...............cCuuccugaagugucaaauag............................................................    4          1          dv1
...............cguuccugGagugucaaauag............................................................    1          1          dv1
...............Aguuccugaagugucaaauag............................................................    1          1          dv1
...............cguuccugaagugucaaauagu...........................................................    15         0          dv1
...............Aguuccugaagugucaaauagu...........................................................    1          1          dv1
...............cguuccugaagugucaaCuagu...........................................................    1          1          dv1
...............cguuccugaagugucaaauCgu...........................................................    1          1          dv1
...............cguuccugaagugucaCauagu...........................................................    1          1          dv1
...............cguuccCgaagugucaaauagua..........................................................    1          1          dv1
...............cguuccugaagugucCaauagua..........................................................    1          1          dv1
...............cguuccugaagugucaaauaguC..........................................................    230        1          dv1
...............Aguuccugaagugucaaauagua..........................................................    1          1          dv1
...............cguuccugaaguguUaaauagua..........................................................    2          1          dv1
...............cguuccugaagugucaaauagua..........................................................    24         0          dv1
...............cguuccugaagugucaaauaguaC.........................................................    2          1          dv1
...............cguuccugaagugucaaauaguaA.........................................................    1          1          dv1
...............cguuccugaagugucaaauaguagg........................................................    1          0          dv1
................Uuccugaagugucaaau...............................................................    1          1          dv1
................guuccugaagugucaCau..............................................................    1          1          dv1
................guuccugaagugucaaau..............................................................    2          0          dv1
................guuccugaagugucaaaua.............................................................    1          0          dv1
................Cuuccugaagugucaaauagu...........................................................    2          1          dv1
.........................................................uguacguugauuuuuauuuggcacC..............    1          1          dv1
.....................................................................uuuauuuggcacaucagugaca.....    2          0          dv1
......................................................................uuauuuggcacaucaguC........    1          1          dv1
......................................................................uuauuuggcacaucagug........    1          0          dv1
......................................................................uuauuuggcacaucaguga.......    3          0          dv1
......................................................................uuauuuggcacaucagugU.......    1          1          dv1
......................................................................uuauuuggcacaucagugac......    1          0          dv1
......................................................................uuauuuggcacaucagugaA......    1          1          dv1
......................................................................uuauuuggcacaucagugCca.....    1          1          dv1
......................................................................uuauuuggcacaucaguUaca.....    2          1          dv1
......................................................................uuauuuggcacaucagugaca.....    46         0          dv1
......................................................................uuauuuggcacaucaUugaca.....    1          1          dv1
......................................................................uuauuuggcacaucUgugacag....    1          1          dv1
......................................................................uuauuuggcacaucagCgacag....    1          1          dv1
......................................................................uuauuuggcacaCcagugacag....    1          1          dv1
......................................................................uuauuuggcacaucagugacCg....    2          1          dv1
......................................................................uuauuuggcacaucagugacag....    150        0          dv1
......................................................................uuauuuggcacaucagugacaU....    1          1          dv1
......................................................................uuauuuggUacaucagugacag....    1          1          dv1
......................................................................uuauuuggcacaucagCgacagc...    1          1          dv1
......................................................................uuauuuggcacaucaUugacagc...    1          1          dv1
......................................................................uuauuuggcacaucagugacagU...    19         1          dv1
......................................................................uuauuuggcacGucagugacagc...    2          1          dv1
......................................................................Guauuuggcacaucagugacagc...    1          1          dv1
......................................................................uuauuuggcacaucagugacagc...    46         0          dv1
......................................................................uuauuuggcaUaucagugacagc...    2          1          dv1
......................................................................uuauuuggcacaucagugacagcU..    17         1          dv1
......................................................................uuauuuggcacaucagugacagca..    2          0          dv1
.......................................................................uauuuggcacaucaguga.......    3          0          dv1
.......................................................................uauuuggcaUaucagugaca.....    1          1          dv1
```

dvi-miR-9697-3p

dvi-miR-9697-5p

caucuggugacuagccguuccugaagugucaaauaguagguacuacucguaagaaaauguacguugauuuuuauuuggcacaucagugacagcaug

....uauuugUcacaucagugaca.....	1	1	dv1
....uGuuuggcacaucagugaca.....	1	1	dv1
....uauuuggcacaucagugaca.....	36	0	dv1
....uauuuggcacaCcagugacag....	1	1	dv1
....uauuuggcacaucagugacGg....	1	1	dv1
....uauuuggcacaucagugaUag....	1	1	dv1
....uauuuggcaUaucagugacag....	1	1	dv1
....uauuuggcacaucagugacag....	122	0	dv1
....uauuuggcacaucagugacCg....	3	1	dv1
....uauuuUgcacaucagugacag....	1	1	dv1
....uauuuggcacaucCgugacagc...	1	1	dv1
....uauuuggcGcaucagugacagc...	1	1	dv1
....uauuuggcacaucagugacagU...	21	1	dv1
....uauuuggcacaucagugCcagc...	1	1	dv1
....uauuuggcacaucagugacagc...	63	0	dv1
....uauuuggcacaucagugacUgc...	1	1	dv1
....uauuuggcacaucagugacCgc...	1	1	dv1
....uauuuggcacaucagugacagcU..	30	1	dv1
....uauuuggcacaucagugacagcC..	3	1	dv1
....uuuggcacaucagugacag....	2	0	dv1
....uuuggcacaucagugacagc...	3	0	dv1
....uuuggcacaucagugacagU...	1	1	dv1
....uuuggcacaucagugacagcC..	1	1	dv1
....uuuggcacaucagugacagca..	44	0	dv1
....uuuggcacaucagugacagcau.	18	0	dv1
....uuuggcacaucagugacagcauU	1	1	dv1
....uuuggcacaucagugacagcaug	22	0	dv1
....uuuggcacaucagugacagcauC	5	1	dv1
....uuuggcacaucagugacagcCug	1	1	dv1
....uuggcacaucagugacagU...	1	1	dv1
....uuggcacaucagugacagc...	13	0	dv1
....uuggcacaucagugacagcC..	8	1	dv1
....uuggcacaucagCgacagca..	2	1	dv1
....uuggcacaucagGgacagca..	2	1	dv1
....uuggcacaucagugacagca..	189	0	dv1
....uuggcacaucUgugacagca..	1	1	dv1
....uuggcacaucagugacagcG..	1	1	dv1
....uuAgcacaucagugacagca..	1	1	dv1
....uuggcacaucagugacagAa..	1	1	dv1
....uuUgcacaucagugacagca..	1	1	dv1
....uuggcacaucagugacagUau.	2	1	dv1
....uuggcacaucagCgacagcau.	4	1	dv1
....uuggcacaucagugacagcCu.	6	1	dv1
....uuggcacaCcagugacagcau.	1	1	dv1
....uuUgcacaucagugacagcau.	1	1	dv1
....uuggcacaucagGgacagcau.	1	1	dv1
....uuggcacaucagugacCgcau.	4	1	dv1
....uuggcacaucagugacagAau.	1	1	dv1
....uuggcacaucagugCcagcau.	1	1	dv1
....uuCgcacaucagugacagcau.	1	1	dv1
....Guggcacaucagugacagcau.	1	1	dv1
....uuggcacaucagugacagcau.	445	0	dv1
....uuggcacaucagAgacagcau.	2	1	dv1
....uuggcacaucaguUacagcau.	1	1	dv1
....uuggcacaucagugacGgcau.	1	1	dv1
....uuggcacaucagugaUagcau.	1	1	dv1
....uuggAacaucagugacagcau.	2	1	dv1
....uuggcacaucGgugacagcau.	1	1	dv1
....uuggcaUaucagugacagcaug	1	1	dv1
....uuggcacaucagugacagcaug	186	0	dv1
....uuggcacaucagugacaAcaug	2	1	dv1
....uuggcacaucaguCacagcaug	1	1	dv1
....uuggcacaucagugacagcCug	14	1	dv1
....uuggcacaucagugacaUcaug	1	1	dv1
....uuggcacaucagugacCgcaug	2	1	dv1
....uuggcacaucagugacagcauU	7	1	dv1
....uuggcacaucagugacagcauC	51	1	dv1
....uuggcacaucagugCcagcaug	3	1	dv1
....uCggcacaucagugacagcaug	1	1	dv1

dvi-miR-9697-3p

dvi-miR-9697-5p

caucuggugacuagccguuccugaagugucaaauaguagguacuacucguaagaaaauguacguugauuuuuauuuggcacaucagugacagcaug

....uuggcacaucagugacagUaug	2	1	dv1
...........uagccguuccugaaguguca...	1	0	dv9
...........uagccguuccugaagugucaa..	4	0	dv9
...........uagccguuccugaagugucCa..	1	1	dv9
...........uagccguuccugaagugucaaa...	1	0	dv9
...........uagccguuccugaagugucaaau..	1	0	dv9
............agccguuccugaaguguA..	1	1	dv9
............agccguuccugaaguguc..	12	0	dv9
............agccguuccugaagugGca...	1	1	dv9
............Cgccguuccugaaguguca...	1	1	dv9
............agccguuccugaagugucG...	1	1	dv9
............agccguuccugaagugucC...	15	1	dv9
............agccguuAcugaaguguca...	1	1	dv9
............agccguuccugaaguguca...	149	0	dv9
............agccguuccCgaaguguca...	1	1	dv9
............agccguuccugaCguguca...	2	1	dv9
............Ggccguuccugaagugucaa..	1	1	dv9
............agccguuccugaagugucGa..	2	1	dv9
............aCccguuccugaagugucaa..	3	1	dv9
............agccguuccuAaagugucaa..	1	1	dv9
............aAccguuccugaagugucaa..	1	1	dv9
............Cgccguuccugaagugucaa..	1	1	dv9
............aUccguuccugaagugucaa..	2	1	dv9
............agccguCccugaagugucaa..	1	1	dv9
............agccguuccugCagugucaa..	2	1	dv9
............agccguuccugaagugCcaa..	3	1	dv9
............agccguuccugaagugucaa..	1134	0	dv9
............agccguuccugaagCgucaa..	6	1	dv9
............agccguuccugaaguCucaa..	4	1	dv9
............agccguuccugaagugucaG..	1	1	dv9
............agccguuccugaaguguUaa..	1	1	dv9
............agccguuccugaagugucCa..	15	1	dv9
............agAcguuccugaagugucaa..	2	1	dv9
............agccguuccugaCgugucaa..	13	1	dv9
............agccguuccugaagugucUa..	1	1	dv9
............agccguuccugaagugucaU..	1	1	dv9
............agccguuccugGagugucaa..	2	1	dv9
............agccguuccugaUgugucaa..	1	1	dv9
............agccguuUcugaagugucaa..	1	1	dv9
............agccguuccugaagGgucaa..	1	1	dv9
............agcAguuccugaagugucaa..	1	1	dv9
............agccguuccugaagugucaC..	28	1	dv9
............agccguuccugaaUgugucaa..	3	1	dv9
............agccguuccugaaguUucaa..	1	1	dv9
............agccguuccugaGgugucaa..	1	1	dv9
............agccguuccugaaguCucaaa...	1	1	dv9
............agccguuccugaagugGcaaa...	1	1	dv9
............agccguuccugaagugucaaU...	8	1	dv9
............agccguuccugaagAgucaaa...	2	1	dv9
............agccguuccugCagugucaaa...	1	1	dv9
............agccUuuccugaagugucaaa...	2	1	dv9
............agccguuccugaagugucGaa...	1	1	dv9
............agccCuuccugaagugucaaa...	1	1	dv9
............agccguucUugaagugucaaa...	2	1	dv9
............agccAuuccugaagugucaaa...	1	1	dv9
............agccguuccCgaagugucaaa...	3	1	dv9
............agccguuccugaagugucaCa...	20	1	dv9
............agccguuccugaagugucaaa...	792	0	dv9
............agccguuccugaCgugucaaa...	11	1	dv9
............agccguuccuUaagugucaaa...	3	1	dv9
............agccguuccugaaguguUaaa...	1	1	dv9
............agccguCccugaagugucaaa...	2	1	dv9
............agcUguuccugaagugucaaa...	1	1	dv9
............agccguuccugaaUgucaaa...	1	1	dv9
............agccguuccugaagugCcaaa...	2	1	dv9
............agccguuccugaaCugucaaa...	2	1	dv9
............agccguuccugaagugucaGa...	2	1	dv9
............agccguuAcugaagugucaaa...	1	1	dv9

```
                                                                              dvi-miR-9697-3p
            dvi-miR-9697-5p
caucuggugacuagccguuccugaagugucaaauaguagguacuacucguaagaaaauguacguugauuuuuauuuggcacaucagugacagcaug

............agccgCuccugaagugucaaa...............................................................   1      1      dv9
............agccguuccugaagugucaaC...............................................................   32     1      dv9
............agccguuccugaagCgucaaa...............................................................   1      1      dv9
............Ggccguuccugaagugucaaa...............................................................   1      1      dv9
............aAccguuccugaagugucaaa...............................................................   1      1      dv9
............agccguuccugaagugucCaa...............................................................   14     1      dv9
............agccguuccugaagugucaaG...............................................................   2      1      dv9
............Cgccguuccugaagugucaaa...............................................................   5      1      dv9
............aAccguuccugaagugucaaau..............................................................   1      1      dv9
............agUcguuccugaagugucaaau..............................................................   1      1      dv9
............agccguuccugaagugucaaCu..............................................................   88     1      dv9
............aCccguuccugaagugucaaau..............................................................   4      1      dv9
............agccguuccugaagugucGaau..............................................................   6      1      dv9
............agccguuccugaagugucCaau..............................................................   43     1      dv9
............agccguuccugaaCugucaaau..............................................................   3      1      dv9
............agccguuccugaGgugucaaau..............................................................   3      1      dv9
............agccguucUugaagugucaaau..............................................................   2      1      dv9
............Ggccguuccugaagugucaaau..............................................................   1      1      dv9
............agccguuccugGagugucaaau..............................................................   4      1      dv9
............Ugccguuccugaagugucaaau..............................................................   2      1      dv9
............agccgCuccugaagugucaaau..............................................................   4      1      dv9
............agccguuccugaCgugucaaau..............................................................   26     1      dv9
............agccguuccugaagugucaCau..............................................................   132    1      dv9
............agccguuccCgaagugucaaau..............................................................   2      1      dv9
............agccguuccugaagugucaaau..............................................................   2917   0      dv9
............agccguuccugaaAugucaaau..............................................................   1      1      dv9
............agccUuuccugaagugucaaau..............................................................   4      1      dv9
............agccguuccGgaagugucaaau..............................................................   2      1      dv9
............agccguuccugaagugucaaaC..............................................................   7      1      dv9
............agccguuccugaagCgucaaau..............................................................   10     1      dv9
............agccguuccugaagugucaaaA..............................................................   16     1      dv9
............aUccguuccugaagugucaaau..............................................................   5      1      dv9
............agccguuccugaUgugucaaau..............................................................   1      1      dv9
............agccguuccugaagugucaaaG..............................................................   1      1      dv9
............agccguuccugaagugucaUau..............................................................   1      1      dv9
............agccguuccuCaagugucaaau..............................................................   1      1      dv9
............agccguucAugaagugucaaau..............................................................   2      1      dv9
............agccguuccugaagGgucaaau..............................................................   2      1      dv9
............agccCuuccugaagugucaaau..............................................................   3      1      dv9
............agccguuccugaaUgucaaau...............................................................   2      1      dv9
............agccguCccugaagugucaaau..............................................................   8      1      dv9
............agccguuccugaaguCucaaau..............................................................   2      1      dv9
............agccguuccugaagugucaGau..............................................................   1      1      dv9
............agccguuUcugaagugucaaau..............................................................   1      1      dv9
............agAcguuccugaagugucaaau..............................................................   1      1      dv9
............agccguuccugaaguguUaaau..............................................................   2      1      dv9
............agccguuccugaagugucaaGu..............................................................   4      1      dv9
............agccguuccugaagugCcaaau..............................................................   8      1      dv9
............agccguuAcugaagugucaaau..............................................................   2      1      dv9
............agccguuccugaagugAcaaau..............................................................   2      1      dv9
............agccguuccugaaguUucaaau..............................................................   6      1      dv9
............Cgccguuccugaagugucaaau..............................................................   11     1      dv9
............agccguuccugaagugucaaUu..............................................................   9      1      dv9
............agccAuuccugaagugucaaau..............................................................   3      1      dv9
............agcUguuccugaagugucaaau..............................................................   3      1      dv9
............agccguuccAgaagugucaaau..............................................................   1      1      dv9
............agccguuccugCagugucaaau..............................................................   3      1      dv9
............agccguuccugaagugucaaauG.............................................................   1      1      dv9
............agccguuccugaagugucaaauC.............................................................   88     1      dv9
............agccguuccugaagGgucaaaua.............................................................   1      1      dv9
............agccguuccugaagugucaaCua.............................................................   5      1      dv9
............agccguuccugaagugucaaaua.............................................................   109    0      dv9
............agccguuccugaagugCcaaaua.............................................................   1      1      dv9
............agccguuccugaagugucaaGua.............................................................   1      1      dv9
............agccguuccugaagugucaCaua.............................................................   5      1      dv9
............agccguuccugaagugucaaauU.............................................................   79     1      dv9
............agccguuccugaagugucaaaAa.............................................................   1      1      dv9
............agccguuccugGagugucaaaua.............................................................   1      1      dv9
............Cgccguuccugaagugucaaaua.............................................................   1      1      dv9
```

```
                                                                      dvi-miR-9697-3p
            dvi-miR-9697-5p
caucuggugacuagccguuccugaagugucaaauaguagguacuacucguaagaaaauguacguugauuuuuauuuggcacaucagugacagcaug

............agccguuccugaagugucaaauaU............................................................   1    1   dv9
............agccguuccugaagugucaaauaA............................................................   6    1   dv9
.............gccguuccugaaguguca.................................................................   1    0   dv9
.............Uccguuccugaagugucaaau..............................................................   1    1   dv9
.............gccguuccugaagugucaaau..............................................................   2    0   dv9
..............ccguuccugaagugucaaau..............................................................   2    0   dv9
..............ccguuccugaagugucaaauC.............................................................   1    1   dv9
..............ccguuccugaagugucaaaua.............................................................   1    0   dv9
...............cguuccugaagugucaaau..............................................................   10   0   dv9
...............cguuccugaagugucaaCu..............................................................   1    1   dv9
...............cguuccugaagugucaCau..............................................................   3    1   dv9
...............Aguuccugaagugucaaau..............................................................   1    1   dv9
...............cguuccugaagugucaaaua.............................................................   4    0   dv9
...............cguuccugaagugucaaaAa.............................................................   1    1   dv9
...............cguuccugaagugucaaCuag............................................................   2    1   dv9
...............cguuccugaagugucaaauaU............................................................   1    1   dv9
...............cguuccugaagugucaaauCg............................................................   5    1   dv9
...............cguuccugaagugucaCauag............................................................   1    1   dv9
...............Aguuccugaagugucaaauag............................................................   2    1   dv9
...............cguuccugaagugucaaauag............................................................   43   0   dv9
...............cguuccugCagugucaaauagu...........................................................   1    1   dv9
...............cguuUcugaagugucaaauagu...........................................................   1    1   dv9
...............cguuccugaagugucaaauagu...........................................................   43   0   dv9
...............cguuccugaaguCucaaauagu...........................................................   1    1   dv9
...............Aguuccugaagugucaaauagu...........................................................   3    1   dv9
...............cUuuccugaagugucaaauagu...........................................................   1    1   dv9
...............cguuccugaagugucaaCuagu...........................................................   2    1   dv9
...............cguuccugaagugucaaauagua..........................................................   42   0   dv9
...............cguuccugaagugucaaauaguC..........................................................   353  1   dv9
...............cguuccugaagugucaCauagua..........................................................   1    1   dv9
...............cguuccugaagugucaaauaguU..........................................................   1    1   dv9
...............cguuAcugaagugucaaauagua..........................................................   1    1   dv9
...............cguuccugaagugucaaauaguag.........................................................   1    0   dv9
...............cguuccugaagugucaaauaguaA.........................................................   1    1   dv9
................guuAcugaagugucaaau..............................................................   1    1   dv9
................guuccugaagugucaaau..............................................................   4    0   dv9
................guuccugaagugucaCau..............................................................   1    1   dv9
................guuccugaagugucaaauaguC..........................................................   2    1   dv9
.................uuccugaagugucaaauaguC..........................................................   1    1   dv9
......................................................................uuauuuggcacaucagug........   1    0   dv9
......................................................................uuauuuggcacaucaguga.......   5    0   dv9
......................................................................uuauCuggcacaucaguga.......   1    1   dv9
......................................................................uuauuuggcacaucagugac......   4    0   dv9
......................................................................uuauuuggcacaucagugaca.....   53   0   dv9
......................................................................uuauuCggcacaucagugaca.....   1    1   dv9
......................................................................uuauuuggcacGucagugaca.....   1    1   dv9
......................................................................uuauuugCcacaucagugaca.....   1    1   dv9
......................................................................uuauuuggcacaucaCugacag....   1    1   dv9
......................................................................uuauuuggcacaucagugacag....   136  0   dv9
......................................................................uuauuuggcacaucGgugacag....   1    1   dv9
......................................................................uuauuuggcacaucagugacaU....   2    1   dv9
......................................................................uuauuuggcaUaucagugacag....   1    1   dv9
......................................................................uuauuuggcacaucCgugacag....   2    1   dv9
......................................................................uuauuuggcacaucagugCcag....   2    1   dv9
......................................................................uCauuuggcacaucagugacag....   2    1   dv9
......................................................................uuauuuggcacaucagugacCg....   3    1   dv9
......................................................................uuauuuggcacaucagugacagc...   111  0   dv9
......................................................................uuauuuggcacaucagugacaUc...   3    1   dv9
......................................................................uuauuuggcacaucagugacaCc...   1    1   dv9
......................................................................uuauuuggcacaucaguUacagc...   1    1   dv9
......................................................................uuauuuggcacaucagugacagU...   25   1   dv9
......................................................................uuauuuggcacaucagugacCgc...   2    1   dv9
......................................................................uuauuuggcacaucagCgacagc...   2    1   dv9
......................................................................uCauuuggcacaucagugacagc...   2    1   dv9
......................................................................uuauuuggcacaucagugCcagc...   1    1   dv9
......................................................................uuauuuggcacaucagugacagcG..   1    1   dv9
......................................................................uuauuuggcacaucagugacagca..   2    0   dv9
......................................................................uuauuuggcacaucagugacagcU..   12   1   dv9
......................................................................uuauuuggcacaucagugacagcC..   2    1   dv9
```

```
                                                                      dvi-miR-9697-3p
            dvi-miR-9697-5p
caucuggugacuagccguuccugaagugucaaauaguagguacuacucguaagaaaauguacguugauuuuuauuuggcacaucagugacagcaug

......................................................................uuauuuggcacaucagugacagcCu.   1     1     dv9
.......................................................................uauuuggcacaucagugaca.....   19    0     dv9
.......................................................................uauuugCcacaucagugaca.....   1     1     dv9
.......................................................................uauuuggcacaucagugacCg....   2     1     dv9
.......................................................................uauuuggcaAaucagugacag....   1     1     dv9
.......................................................................uauuuggcacaucagugacaU....   1     1     dv9
.......................................................................uauuuggcacaucCgugacag....   1     1     dv9
.......................................................................Gauuuggcacaucagugacag....   1     1     dv9
.......................................................................uauuuggcacaucaguUacag....   3     1     dv9
.......................................................................uauuuggcacaucagugacag....   136   0     dv9
.......................................................................uauuuggcacaucagugCcag....   5     1     dv9
.......................................................................uauuuggcacauUagugacagc...   1     1     dv9
.......................................................................uauuuggUacaucagugacagc...   1     1     dv9
.......................................................................uauuuggcacaucaguCacagc...   1     1     dv9
.......................................................................uauuuggcacaucagugacagU...   18    1     dv9
.......................................................................uauuuggcacaucagugacagc...   135   0     dv9
.......................................................................uauuuggcacaucagugacaUc...   3     1     dv9
.......................................................................uauuuggcacaucagugacGgc...   1     1     dv9
.......................................................................uauuuggcacaucagugacagA...   1     1     dv9
.......................................................................uauuuggcacaucCgugacagc...   2     1     dv9
.......................................................................uauuuggcacaucagugacagca..   2     0     dv9
.......................................................................uauuuggcacaucagugacagcG..   1     1     dv9
.......................................................................uauuuggcacaucagugacagcU..   24    1     dv9
.......................................................................uauuuggcacaucagugacagcC..   3     1     dv9
.......................................................................uauuuggcacaucagugacagcCu.   3     1     dv9
.........................................................................uuuggcacaucagugaca.....   3     0     dv9
.........................................................................uuuggcacaucagugacag....   2     0     dv9
.........................................................................uuuggcacaucagugacagc...   11    0     dv9
.........................................................................uuCggcacaucagugacagca..   1     1     dv9
.........................................................................uuuAgcacaucagugacagca..   1     1     dv9
.........................................................................uuuggcacaucagugaUagca..   1     1     dv9
.........................................................................uuuggcacaucagugacagcG..   2     1     dv9
.........................................................................uuuggcacaucagugacagcU..   3     1     dv9
.........................................................................uuuggcacaucagugacagcC..   4     1     dv9
.........................................................................uuuggcacaucagugacagca..   38    0     dv9
.........................................................................uuuggcacaucaguCacagca..   1     1     dv9
.........................................................................uuuggcacaucagCgacagca..   1     1     dv9
.........................................................................uuuggcacaucagugacCgca..   1     1     dv9
.........................................................................uuuggcacaucagugacagcau.   38    0     dv9
.........................................................................Guuggcacaucagugacagcau.   2     1     dv9
.........................................................................uuuggcacaucagugacagcCug   1     1     dv9
.........................................................................uuuggcacCucagugacagcaug   1     1     dv9
.........................................................................uuuggcacaucagugCcagcaug   1     1     dv9
.........................................................................uuuggcacaucagugacagcauC   8     1     dv9
.........................................................................uuuggcacaucagugacagcaug   48    0     dv9
.........................................................................uuuggcacaucagugacagcUug   1     1     dv9
.........................................................................uuCggcacaucagugacagcaug   1     1     dv9
.........................................................................uuuAgcacaucagugacagcaug   2     1     dv9
.........................................................................uuuggcacaucagugacagcaCg   1     1     dv9
..........................................................................uuggcaUaucagugacagc...   1     1     dv9
..........................................................................uuggcacaucagugacagc...   23    0     dv9
..........................................................................uuggcacaucagugacagU...   2     1     dv9
..........................................................................uuggcacaucagugacagcC..   5     1     dv9
..........................................................................uuggcacaucagugacagcG..   1     1     dv9
..........................................................................uuAgcacaucagugacagca..   1     1     dv9
..........................................................................uuggcaAaucagugacagca..   1     1     dv9
..........................................................................uuggcacaucagugacCgca..   3     1     dv9
..........................................................................uuggcacaucagugacagca..   215   0     dv9
..........................................................................uugCcacaucagugacagca..   1     1     dv9
..........................................................................uuggcacaucagGgacagca..   2     1     dv9
..........................................................................uuggcacaucagGgacagcau.   1     1     dv9
..........................................................................uuggcacaucagugacaCcau.   1     1     dv9
..........................................................................uuggcacaucagugCcagcau.   3     1     dv9
..........................................................................uuggcacaucagugacCgcau.   6     1     dv9
..........................................................................uuggcaUaucagugacagcau.   4     1     dv9
..........................................................................uuggcacaucagugaUagcau.   1     1     dv9
..........................................................................uuggcacaucagugacagcaC.   5     1     dv9
..........................................................................uuggcacaucaAugacagcau.   1     1     dv9
..........................................................................uuggcGcaucagugacagcau.   1     1     dv9
```

```
                                                                                     dvi-miR-9697-3p
            dvi-miR-9697-5p
caucuggugacuagccguuccugaagugucaaauaguagguacuacucguaagaaaauguacguugauuuuuauuuggcacaucagugacagcaug

..........................................................................uuCgcacaucagugacagcau.   2      1      dv9
..........................................................................uuggcacCucagugacagcau.   1      1      dv9
..........................................................................uuUgcacaucagugacagcau.   1      1      dv9
..........................................................................uuggcacaucagugacagcCu.   10     1      dv9
..........................................................................uuggcacaucGgugacagcau.   1      1      dv9
..........................................................................uuggcacaucaguUacagcau.   1      1      dv9
..........................................................................uuggcacaucagugacagcaA.   2      1      dv9
..........................................................................uuggcacaucagugacagcaG.   1      1      dv9
..........................................................................uuggcacaucagugacagcau.   747    0      dv9
..........................................................................uuggcacaucaguCacagcau.   3      1      dv9
..........................................................................uuggcacGucagugacagcau.   1      1      dv9
..........................................................................uuggcacaucagugacGgcau.   1      1      dv9
..........................................................................uCggcacaucagugacagcau.   1      1      dv9
..........................................................................uuggcacaucUgugacagcaug   1      1      dv9
..........................................................................uuggcacaAcagugacagcaug   1      1      dv9
..........................................................................uuggcacaucagugacCgcaug   6      1      dv9
..........................................................................uuggcacaucagugacagcauC   76     1      dv9
..........................................................................uuggcacaucagugacagcaug   390    0      dv9
..........................................................................uuggcacaucagugacagcCug   34     1      dv9
..........................................................................uuggcacauUagugacagcaug   1      1      dv9
..........................................................................uuAgcacaucagugacagcaug   1      1      dv9
..........................................................................uuggcacaucagugacagcauU   6      1      dv9
..........................................................................uuggcacaucagugCcagcaug   1      1      dv9
..........................................................................uuggcacaucagugacagcaCg   1      1      dv9
..........................................................................uuggcacaucagCgacagcaug   2      1      dv9
..........................................................................uuggcacaucGgugacagcaug   2      1      dv9
..........................................................................uuggAacaucagugacagcaug   1      1      dv9
..........................................................................uGggcacaucagugacagcaug   2      1      dv9
..........................................................................uuggcacaucagugacagcauA   1      1      dv9
..........................................................................uuUgcacaucagugacagcaug   1      1      dv9
..........................................................................Guggcacaucagugacagcaug   1      1      dv9
..........................................................................uuggcacaCcagugacagcaug   1      1      dv9
...........................................................................uUgcacaucagugacagcau.   1      1      dv9
...........................................................................uggcacaucagugacagcau.   1      0      dv9
.............................................................................gcacaucagugacagcauC   1      1      dv9

...........uagccguuccugaaguguca.................................................................   1      0      arg
...........uagccguuccugaagugucaaa...............................................................   1      0      arg
............agccguuccugaagCguc..................................................................   1      1      arg
............agccguuccugaaguguU..................................................................   1      1      arg
............agccguuccugaaguguc..................................................................   42     0      arg
............agccguuccugaaguguca.................................................................   91     0      arg
............agccguuccugaagugucC.................................................................   20     1      arg
............agccguuccugaagGguca.................................................................   1      1      arg
............agccguuccugaagugucU.................................................................   5      1      arg
............agccgGuccugaaguguca.................................................................   1      1      arg
............agccguuccugaCguguca.................................................................   1      1      arg
............Ggccguuccugaagugucaa................................................................   1      1      arg
............agccguucAugaagugucaa................................................................   1      1      arg
............agccUuuccugaagugucaa................................................................   1      1      arg
............agccguuccugaagGgucaa................................................................   1      1      arg
............agccguuccugaGgugucaa................................................................   1      1      arg
............agccguuAcugaagugucaa................................................................   1      1      arg
............agccguuccugaaguguAaa................................................................   2      1      arg
............agccguuccugaagugucGa................................................................   1      1      arg
............agccguuccugaagugucaa................................................................   280    0      arg
............agccgGuccugaagugucaa................................................................   1      1      arg
............agccguuccugGagugucaa................................................................   1      1      arg
............agccguuccugaaAugucaaa...............................................................   1      1      arg
............agccguuccugaaguguAaaa...............................................................   1      1      arg
............agccguuccugaagugucaaa...............................................................   601    0      arg
............agccguCccugaagugucaaa...............................................................   1      1      arg
............agccguuccugaagugucCaa...............................................................   1      1      arg
............agccguuccugaagCgucaaa...............................................................   3      1      arg
............Ugccguuccugaagugucaaa...............................................................   1      1      arg
............agccguuccugaagugucaaC...............................................................   1      1      arg
............agGcguuccugaagugucaaa...............................................................   1      1      arg
............agccguuccugaagugAcaaa...............................................................   1      1      arg
............agccguuccugaagugCcaaa...............................................................   3      1      arg
```

```
                                                                                     dvi-miR-9697-3p
            dvi-miR-9697-5p
caucuggugacuagccguuccugaagugucaaauaguagguacuacucguaagaaaauguacguugauuuuuauuuggcacaucagugacagcaug

............agccUuuccugaagugucaaa...............................................................    1         1         arg
............agccgCuccugaagugucaaa...............................................................    1         1         arg
............agccguuccugaagugucaGa...............................................................    1         1         arg
............aCccguuccugaagugucaaa...............................................................    1         1         arg
............agccguuccGgaagugucaaa...............................................................    1         1         arg
............agccguuccugaagugucaaG...............................................................    1         1         arg
............agccguuccugaaguCucaaa...............................................................    1         1         arg
............agccguuccugaagugucaaU...............................................................    1         1         arg
............agccguuccugaagAgucaaa...............................................................    1         1         arg
............agccguuccugaGgugucaaa...............................................................    2         1         arg
............agccguuccugaagGgucaaa...............................................................    1         1         arg
............agccgAuccugaagugucaaa...............................................................    1         1         arg
............agccguuccugaagugucGaa...............................................................    2         1         arg
............agccguuccugaaguUucaaau..............................................................    1         1         arg
............agccguuccugaaguguUaaau..............................................................    1         1         arg
............agccguuccugaagugucaaCu..............................................................    1         1         arg
............agccguuccugaagugucaaaC..............................................................    4         1         arg
............agccguuccugaagugucaaau..............................................................    1018      0         arg
............agAcguuccugaagugucaaau..............................................................    1         1         arg
............agccguuccugaagugucaaaA..............................................................    7         1         arg
............agccguuccugaCgugucaaau..............................................................    1         1         arg
............agccguuccuAaagugucaaau..............................................................    2         1         arg
............agccguuccugaagugCcaaau..............................................................    3         1         arg
............agccUuuccugaagugucaaau..............................................................    1         1         arg
............agcUguuccugaagugucaaau..............................................................    2         1         arg
............agUcguuccugaagugucaaau..............................................................    1         1         arg
............agccguuccugaagugAcaaau..............................................................    1         1         arg
............agccguuccugaagugucGaau..............................................................    5         1         arg
............agccguuccugaagugucaCau..............................................................    1         1         arg
............agccguuccugaUgugucaaau..............................................................    1         1         arg
............agccguuccugaaUugucaaau..............................................................    1         1         arg
............agccguuccugaaguguAaaau..............................................................    1         1         arg
............agccguuccugaagugucaaGu..............................................................    2         1         arg
............aCccguuccugaagugucaaau..............................................................    1         1         arg
............agccguucAugaagugucaaau..............................................................    1         1         arg
............agccguuccugaaguAucaaau..............................................................    3         1         arg
............agccguuccugaagugucaaUu..............................................................    2         1         arg
............agccguuccugaaCugucaaau..............................................................    1         1         arg
............agccguuccugaagAgucaaau..............................................................    1         1         arg
............aAccguuccugaagugucaaau..............................................................    1         1         arg
............agccguuUcugaagugucaaau..............................................................    1         1         arg
............agccgGuccugaagugucaaau..............................................................    1         1         arg
............agccguuccugGagugucaaau..............................................................    2         1         arg
............agccguuccugaaAugucaaau..............................................................    1         1         arg
............agccguuccugaagGgucaaau..............................................................    5         1         arg
............agccguuccugaagugucCaau..............................................................    1         1         arg
............agccguuccugaGgugucaaau..............................................................    2         1         arg
............agccguuccugaagugucUaau..............................................................    1         1         arg
............agccguuccugaagugGcaaau..............................................................    1         1         arg
............agccgCuccugaagugucaaau..............................................................    1         1         arg
............Ggccguuccugaagugucaaau..............................................................    1         1         arg
............agccguuccugaagugucaaaG..............................................................    1         1         arg
............agccguuccugaagugucaGau..............................................................    2         1         arg
............agccguuccugaagCgucaaau..............................................................    2         1         arg
............agccguuccugaagugucaaauU.............................................................    72        1         arg
............agccguuccugaagugucaaaua.............................................................    35        0         arg
............agccguuccugaagugucaaauC.............................................................    7         1         arg
..............ccguuccugaagugucaaaua.............................................................    1         0         arg
...............cguuccugaagugucaaa...............................................................    4         0         arg
...............cgGuccugaagugucaaau..............................................................    1         1         arg
...............cguuccugaagugucaaau..............................................................    2         0         arg
...............cguuccugaagugucaaauU.............................................................    1         1         arg
...............cguuccugaagugCcaaaua.............................................................    1         1         arg
...............cguuccugaagugucaaaua.............................................................    8         0         arg
...............cguuccugaagugucaaauC.............................................................    1         1         arg
...............cguuccugaagugucaaauag............................................................    25        0         arg
...............cguucUugaagugucaaauag............................................................    1         1         arg
...............cguuccugaagugucaaaGag............................................................    1         1         arg
...............cguuccugUagugucaaauagu...........................................................    1         1         arg
```

dvi-miR-9697-3p

dvi-miR-9697-5p

caucuggugacuagccguuccugaagugucaaauaguagguacuacucguaagaaaauguacguugauuuuuauuuggcacaucagugacagcaug

...............cguuccugaagugucaaauagu...	36	0	arg
...............cguuccugaagugucaaauagua..	14	0	arg
...............cguuccugaagugucaaauaguU..	5	1	arg
...............cguuccugaagugGcaaauagua..	1	1	arg
...............cguuccugaagugucaaauaguC..	134	1	arg
...............cguuccugaagugucaaauaguaA...	1	1	arg
...............cguuccugaagugucaaauaguCg...	1	1	arg
....................cugaagugucaaauaguC..	1	1	arg
....uuauuuggGacaucagugaca.....	1	1	arg
....uuauuuggcacaucagugaca.....	7	0	arg
....uuauuuggcacaucagugacU.....	1	1	arg
....uuauuuggcacaucagugacag....	4	0	arg
....uuauuuggcacaucagugacagU...	2	1	arg
....uuauuuggcacaucagugacagc...	7	0	arg
....uuauuuggcacaucagugacagcU..	1	1	arg
....uauuuggcacaucagugac......	1	0	arg
....uauuuggcacaucagugaca.....	4	0	arg
....uauuuggcacaucagugacag....	9	0	arg
....uauuuggcacaucagugacGgc...	1	1	arg
....uauuuggcacaucagugacagc...	17	0	arg
....uauuuggcacaucagugacUgc...	1	1	arg
....uauuuggcacaucagugacagU...	4	1	arg
....uauuuggcacaucagugacagcU..	2	1	arg
....uuuggcacaucaguAacagca..	1	1	arg
....uuuggcacaucagugacagca..	6	0	arg
....uuuggcacaucagugacagcau.	5	0	arg
....uuuggcacaucagugacagcaug	3	0	arg
....uuggcacaucagugacag....	1	0	arg
....uuggcacaucagugacagU...	2	1	arg
....uuggcacaucagugacagc...	5	0	arg
....uuggcacaucagCgacagc...	1	1	arg
....uuggcGcaucagugacagca..	1	1	arg
....uuggUacaucagugacagca..	1	1	arg
....uuggcacaucagugacagcU..	1	1	arg
....uuggcacaucagugacagca..	41	0	arg
....uuggcacaucagugacagcau.	48	0	arg
....uuggcacaucagugacagcaug	34	0	arg
....uuggcacaucagugUcagcaug	1	1	arg
....uuggcacaucagugacagcauC	1	1	arg
....cacaucagugacagcaug	1	0	arg

miRBase precursor : dvi-mir-9698
Total read count : 1893
dvi-miR-9698-5p read count : 1350
dvi-miR-9698-3p read count : 543
remaining reads : 0

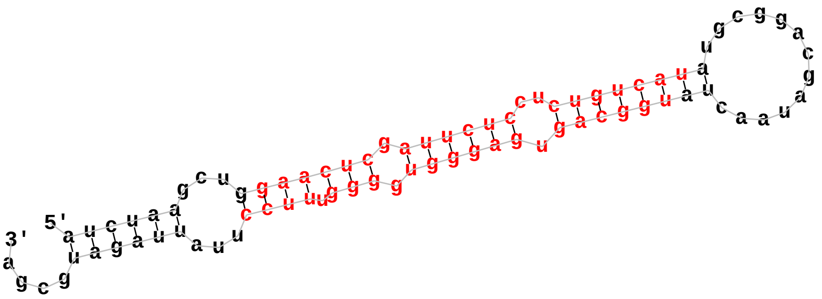

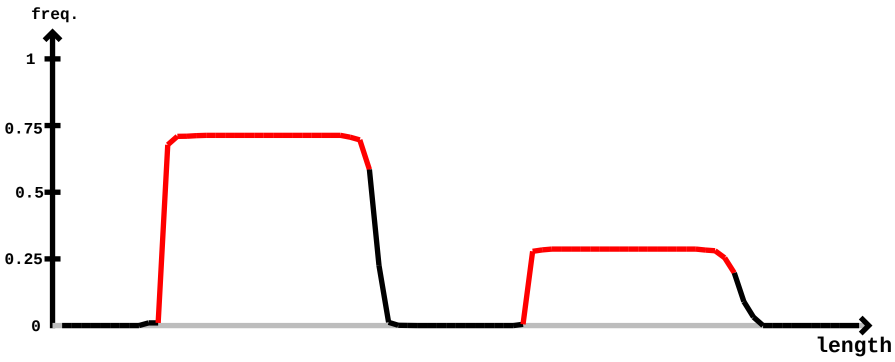


```
                                   dvi-miR-9698-5p
                                                                        dvi-miR-9698-3p
5'- aucuaagcuggaacucgauucuccucugucauaugcggacgauaacuauggcagugagggugggguuuccuuauuagaugcga   -3'    exp
    ((((((...(((((((.((((((..((((((((.............)))))))).)))))).))).))))...))))))....    reads     mm     sample
    ........uggaacucgauucuccucugu......................................................    3         0      arg
    ........uggaacucUauucuccucuguca....................................................    1         1      arg
    ..........gaacucgauucuccucugu......................................................    1         0      arg
    ..........gaacucgauucuccucuguc.....................................................    4         0      arg
    ..........gaacucgauucuccucuguca....................................................    5         0      arg
    ..........gaacucgauucuccucugucau...................................................    15        0      arg
    ..........gaacucgauucuccucugucauC..................................................    11        1      arg
    ..........gaacucgauucuccucugucaua..................................................    2         0      arg
    ...............................................auggcagugagggugggguuC...............    1         1      arg
    ...............................................auggcagugagggugggguuu...............    2         0      arg
    ................................................uggcagugagggugggguu................    4         0      arg
    ................................................uggcagugagggugggAuu................    1         1      arg
    ................................................uggcagugagggugggguuC...............    2         1      arg
    ................................................uggcagugaggguggggGuu...............    1         1      arg
    ................................................uggcagugagggugggguuu...............    21        0      arg
    ................................................uggcagugagggugggguuA...............    1         1      arg
    ................................................uggcagugagggugggguuuc..............    40        0      arg
    ................................................uggcagugagggugggguuuU..............    1         1      arg
    ................................................uggcaguAagggugggguuuc..............    1         1      arg
    ................................................uggcagugagggugggguuuG..............    1         1      arg
    ................................................uggcagugagggugggguuuUc.............    3         1      arg
    ................................................uggcGgugagggugggguuucc.............    1         1      arg
    ................................................ugCcagugagggugggguuucc.............    1         1      arg
    ................................................uggcaguga gggugggCuucc.............    1         1      arg
    ................................................uggcagugagggugggguuucA.............    2         1      arg
    ................................................uggcagugagggugggguuucc.............    53        0      arg
    ................................................uggcagugagggugggguuucU.............    3         1      arg
    ................................................uggcagugagggugggguuuccu............    13        0      arg
    ................................................uggcagugagggugggguuuUcu............    1         1      arg
    ................................................uggcagugagggugggguuuccC............    11        1      arg
    ................................................uUgcagugagggugggguuuccu............    1         1      arg
    ................................................uggcagugagggugggguuuccCu...........    1         1      arg
    ................................................uggcagugagggugggguuuccuC...........    13        1      arg
    ................................................uggcagugagggugggguuuccuu...........    3         0      arg
```

```
          dvi-miR-9698-5p
                                                dvi-miR-9698-3p
aucuaagcuggaacucgauucuccucugucauaugcggacgauaacuauggcagugagggugggguuuccuuauuagaugcga

.................................................ggcagugagggugggguuuccuu...........   1     0     arg
.................................................ggcagugagggugggguuuccuC...........   1     1     arg
..................................................gcagugagggugggguuuc..............   2     0     arg
..................................................gcagugagggugggguuuccu............   1     0     arg
..................................................gcagugagggugggguuuccuC...........   1     1     arg

........uggaacucgauucuccucugu......................................................   2     0     dv9
........uggaacucgauucuccucuguc.....................................................   2     0     dv9
........uggaacucgauucuccucuguca....................................................   7     0     dv9
........uggaacucgauucuccucugucauC..................................................   1     1     dv9
..........gaacucgauucuccucugu......................................................   2     0     dv9
..........Uaacucgauucuccucuguc.....................................................   1     1     dv9
..........gaacucgauucuccucuguc.....................................................   8     0     dv9
..........gaacucgauucuccucuCuc.....................................................   1     1     dv9
..........gaaAucgauucuccucuguca....................................................   1     1     dv9
..........gaacucgauucuccucugucC....................................................   10    1     dv9
..........gaacucgauucuccucuguca....................................................   83    0     dv9
..........gaGcucgauucuccucuguca....................................................   1     1     dv9
..........gaacucgauucuccCcuguca....................................................   2     1     dv9
..........Uaacucgauucuccucuguca....................................................   5     1     dv9
..........gGacucgauucuccucugucau...................................................   1     1     dv9
..........gaacucgauucuccucugucUu...................................................   2     1     dv9
..........gaacuAgauucuccucugucau...................................................   1     1     dv9
..........gaacucgaCucuccucugucau...................................................   1     1     dv9
..........gaacucgauucucUucugucau...................................................   3     1     dv9
..........gCacucgauucuccucugucau...................................................   1     1     dv9
..........Uaacucgauucuccucugucau...................................................   19    1     dv9
..........gaaAucgauucuccucugucau...................................................   1     1     dv9
..........gaacucgauGcuccucugucau...................................................   1     1     dv9
..........gaacucgauucuccucugucCu...................................................   16    1     dv9
..........gaacucgauucuccucugucaG...................................................   1     1     dv9
..........gaacucgauucuccucugucau...................................................   320   0     dv9
..........gaacucgauucuccucugucaC...................................................   2     1     dv9
..........gaacucgauucuccucCgucau...................................................   1     1     dv9
..........gaGcucgauucuccucugucau...................................................   3     1     dv9
..........gaacucUauucuccucugucau...................................................   2     1     dv9
..........gaacucgauucuccucugCcau...................................................   2     1     dv9
..........gaacucgauucCccucugucau...................................................   1     1     dv9
..........gaacucgauucuccucAgucau...................................................   1     1     dv9
..........gaacCcgauucuccucugucau...................................................   1     1     dv9
..........Aaacucgauucuccucugucaua..................................................   1     1     dv9
..........Uaacucgauucuccucugucaua..................................................   3     1     dv9
..........gaacucgauucuccuUugucaua..................................................   1     1     dv9
..........gaacucgauucuccucugucaua..................................................   19    0     dv9
..........gaacucgauucuccucugucauC..................................................   196   1     dv9
..........gaacucgauucuccucugucauaC.................................................   1     1     dv9
..........gaacucgauucuccucugucauCu.................................................   4     1     dv9
...........aacucgauGcuccucuguca....................................................   1     1     dv9
...........aacucgauucuccucuguca....................................................   2     0     dv9
...........aacucgauucuccucugucau...................................................   11    0     dv9
...........aacucgauucuccucugucCu...................................................   1     1     dv9
...........aacucgauucuccucugucaua..................................................   8     0     dv9
...........aacucgauucuccucugucauC..................................................   6     1     dv9
...........aacucgauucuccucugucCuau.................................................   1     1     dv9
...........aacucgauCcuccucugucauau.................................................   1     1     dv9
...........aacucgauucuccucugucauau.................................................   10    0     dv9
...........aacucgauucuccucugucauauC................................................   1     1     dv9
............acucgauucuccucugucau...................................................   1     0     dv9
.............cucgauucuccucugucau...................................................   1     0     dv9
..............ucgauucuccucugucauC..................................................   1     1     dv9
...............................................auggcagugagggugggguuC...............   1     1     dv9
...............................................auggcagugagggugggguuuc..............   2     0     dv9
...............................................auggcagugagggugggguuucc.............   3     0     dv9
................................................uggcagugaggguggggu.................   6     0     dv9
................................................uggcagugagggugggguuu...............   10    0     dv9
................................................uCgcagugagggugggguuu...............   1     1     dv9
................................................uggcagugagggugggguuC...............   1     1     dv9
................................................uggcagugagggugggguuuU..............   1     1     dv9
................................................uggcagugaggAugggguuuc..............   1     1     dv9
```

```
          dvi-miR-9698-5p
                                                dvi-miR-9698-3p
aucuaagcuggaacucgauucuccucugucauaugcggacgauaacuauggcagugagggugggguuuccuuauuagaugcga

................................................uggcagugagggugggguuuc..............    45     0     dv9
................................................uggcagugagggugggguuuG..............    1      1     dv9
................................................uggcagugagggugggguuCc..............    1      1     dv9
................................................uggcaCugagggugggguuuc..............    1      1     dv9
................................................uggcagugagggugggguuucA.............    1      1     dv9
................................................Aggcagugagggugggguuucc.............    1      1     dv9
................................................Cggcagugagggugggguuucc.............    1      1     dv9
................................................uggcagugGgggugggguuucc.............    1      1     dv9
................................................uggcagugaggguggggGuucc.............    1      1     dv9
................................................uggcagugagggugggguuucU.............    2      1     dv9
................................................uggcagugagggugggguuucc.............    100    0     dv9
................................................uggcagugagggugggguuuUc.............    1      1     dv9
................................................uggcagugagUgugggguuuccu............    1      1     dv9
................................................uggcagugagggugggguuuccC............    34     1     dv9
................................................uggcagugagggugggguuuccu............    21     0     dv9
................................................uggcagugagggugggguuuccCu...........    1      1     dv9
................................................uggcagugagggugggguuuccuC...........    26     1     dv9
................................................uggcagugagggugggguuuccuu...........    1      0     dv9
.................................................ggcagugagggugggguuuccC............    1      1     dv9
.................................................ggcagugagggugggguuuccuC...........    5      1     dv9

........uggaacucgauucucUucugu......................................................    1      1     dv1
........uggaacucgauucuccucugu......................................................    2      0     dv1
..........gaacucgauucuccucugu......................................................    2      0     dv1
..........gaacucgauucuccucuguc.....................................................    3      0     dv1
..........gaacucgauucuccucugCca....................................................    1      1     dv1
..........gaacucgauucuccCcuguca....................................................    2      1     dv1
..........gaacucgauucuccuAuguca....................................................    1      1     dv1
..........Uaacucgauucuccucuguca....................................................    1      1     dv1
..........gaacucgGuucuccucuguca....................................................    1      1     dv1
..........gaacucgauucuccucugucC....................................................    6      1     dv1
..........gaacucgauucuccucuguca....................................................    78     0     dv1
..........gaacucgauCcuccucuguca....................................................    1      1     dv1
..........gaacucgCuucuccucuguca....................................................    1      1     dv1
..........gaacucgauucucUucugucau...................................................    1      1     dv1
..........gaacucgauucuccucugCcau...................................................    1      1     dv1
..........gaacucgauCcuccucugucau...................................................    2      1     dv1
..........gaacucgaCucuccucugucau...................................................    1      1     dv1
..........Uaacucgauucuccucugucau...................................................    16     1     dv1
..........gaaUucgauucuccucugucau...................................................    1      1     dv1
..........gaacucgauucuccucCgucau...................................................    1      1     dv1
..........gaacucgauucuccucugucau...................................................    233    0     dv1
..........gaaAucgauucuccucugucau...................................................    1      1     dv1
..........gaacucgauucuccucuUucau...................................................    1      1     dv1
..........gaacucgauucuccucugucCu...................................................    7      1     dv1
..........gaacucgauucuccucugucauC..................................................    128    1     dv1
..........gaacucgauucuccucugucaua..................................................    13     0     dv1
..........gaacucgauucuccucugucCua..................................................    2      1     dv1
..........gaacucgauucuccucugucauCu.................................................    1      1     dv1
..........gaacucgauucuccucugucauauCc...............................................    1      1     dv1
...........aacucgauucuccucugucau...................................................    6      0     dv1
...........aacucgauucuccucugucGu...................................................    1      1     dv1
...........aacucgauucuccucugucaua..................................................    1      0     dv1
...........aacucgauucuccucugucauC..................................................    7      1     dv1
...........aacucgauucuccucugucauaA.................................................    1      1     dv1
...........aacucgauucuccucugucauau.................................................    2      0     dv1
.............cucgauucuccucugucaua..................................................    2      0     dv1
.............cucgauucuccucugucauC..................................................    1      1     dv1
..............ucgauucuccucugucau...................................................    1      0     dv1
................................................uggcagugaggguggggu.................    1      0     dv1
................................................uggcagugagggugggguuC...............    1      1     dv1
................................................uggcagugagggugggguuu...............    8      0     dv1
................................................uggcagugagggugggguuuc..............    8      0     dv1
................................................uggcagugagggugggguuuU..............    1      1     dv1
................................................uggcagugagggugggguuucc.............    26     0     dv1
................................................uggcagugagggugggguuuUc.............    3      1     dv1
................................................uggcagugagggugggguuuccu............    10     0     dv1
................................................ugAcagugagggugggguuuccu............    1      1     dv1
................................................uggcagugagggugggguuuccC............    13     1     dv1
```

```
          dvi-miR-9698-5p
                                                 dvi-miR-9698-3p
aucuaagcuggaacucgauucuccucugucauaugcggacgauaacuauggcagugaggguggggguuuccuuauuagaugcga

...............................................uggcagugaggguggggguuuccA............    1          1          dv1
...............................................uggcagugaggguggggguuuccuA...........    1          1          dv1
...............................................uggcagugaggguggggguuuccuC...........    4          1          dv1
................................................ggcagugaggguggggguuuccu............    1          0          dv1
................................................ggcagugaggguggggguuuccuC...........    1          1          dv1
.................................................gcagugaggguggggguuuc..............    1          0          dv1
.................................................gcagugaggguggggguuuccuC...........    1          1          dv1
```

miRBase precursor : dvi-mir-978
Total read count : 20965
dvi-miR-9699-5p read count : 12370
dvi-miR-9699-3p read count : 8592
remaining reads : 3

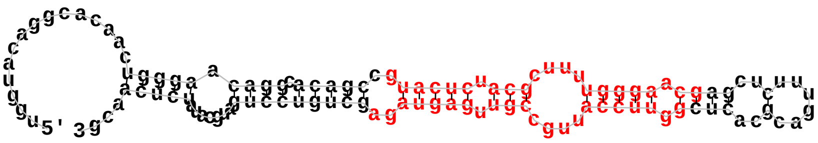

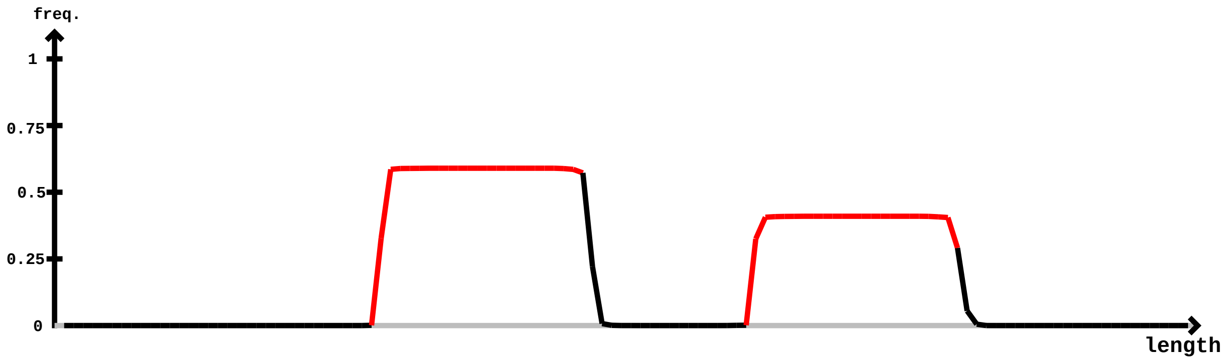

dvi-miR-9699-5p

dvi-miR-9699-3p

5'- ugguacaggcacaacugggaacaggcacagccguacucuacgcuuuugggaacgagcucuuugacgcacucgguuccauugccguugaguagagcuguccugaugcauaucucaacg -3'	exp		
.....(((......)))(.(.((((.(((((..(((((.(((....(((((.((((..(......)..)))).)))))....))).)))))..))))))))).).)...........	reads	mm	sampl
...............................cguacucuacgcuuuugggaa...	1	0	dv1
...............................cguacucuacgcuuuugggaac..	2	0	dv1
...............................cguacucuacgcuuuugggaacU...	1	1	dv1
...............................cguacucuacgcuuuugggaacg...	2	0	dv1
................................guacucuacgcuuuuggga..	4	0	dv1
................................guacucuacgcuuuugggCa...	1	1	dv1
................................Uuacucuacgcuuuugggaa...	1	1	dv1
................................guacucuacgcuuuugggaa...	12	0	dv1
................................guacucuacgcCuuugggaa...	1	1	dv1
................................guacucuacgcuuuuCggaac..	1	1	dv1
................................Uuacucuacgcuuuugggaac..	1	1	dv1
................................guacucuacgcuCuugggaac..	1	1	dv1
................................guacucuacgcuuuugggaac..	38	0	dv1
................................guacucuaAgcuuuugggaac..	1	1	dv1
................................guacucuacgcuuuugUgaacg...	36	1	dv1
................................guacucuacgcuCuugggaacg...	3	1	dv1
................................guaAucuacgcuuuugggaacg...	1	1	dv1
................................guacucuacgcuuuugggaCcg...	60	1	dv1
................................guacucuacgcuuuCgggaacg...	1	1	dv1
................................guacucuacgcuuuuggCaacg...	1	1	dv1
................................guacucuacgcuuuugggGacg...	4	1	dv1
................................guacucuacCcuuuugggaacg...	1	1	dv1
................................guacucuacgcuuuugggaaAg...	2	1	dv1
................................guGcucuacgcuuuugggaacg...	3	1	dv1
................................gAacucuacgcuuuugggaacg...	1	1	dv1
................................guacucuacgcuuuuggAaacg...	1	1	dv1
................................guacucuacgcuuuugCgaacg...	1	1	dv1
................................gGacucuacgcuuuugggaacg...	1	1	dv1
................................guacucuacgcGuuugggaacg...	1	1	dv1
................................guacucuacgcuuuugggaaUg...	4	1	dv1
................................Uuacucuacgcuuuugggaacg...	78	1	dv1
................................guacucuacgcuuuugggUacg...	1	1	dv1
................................guacucuaUgcuuuugggaacg...	6	1	dv1
................................guacucuacgcuuuuUggaacg...	8	1	dv1

```
                                dvi-miR-9699-5p
                                                                       dvi-miR-9699-3p
ugguacaggcacaacugggaacaggcacagccguacucuacgcuuuugggaacgagcucuuugacgcacucgguuccauugccguugaguagagcuguccugaugcauaucucaacg

................................guaUucuacgcuuuugggaacg...............................................................	1	1	dv1
................................guacucuacgcuuuugggaacg...............................................................	1535	0	dv1
................................guacucuacgUuuuugggaacg...............................................................	4	1	dv1
................................guCcucuacgcuuuugggaacg...............................................................	1	1	dv1
................................guacucuacgcuuuugggaacU...............................................................	11	1	dv1
................................guacucuacgcuAuugggaacg...............................................................	1	1	dv1
................................guacucuacgcuuuuggUaacg...............................................................	44	1	dv1
................................guacucuacgcCuuugggaacg...............................................................	4	1	dv1
................................guacucuGcgcuuuugggaacg...............................................................	4	1	dv1
................................guacucuacgcuuuugggaGcg...............................................................	3	1	dv1
................................guacuUuacgcuuuugggaacg...............................................................	1	1	dv1
................................gCacucuacgcuuuugggaacg...............................................................	2	1	dv1
................................guacucCacgcuuuugggaacg...............................................................	2	1	dv1
................................guacucuacUcuuuugggaacg...............................................................	2	1	dv1
................................guacucuacgcuuuugggCacg...............................................................	43	1	dv1
................................guacucuacgcuuuugggaUcg...............................................................	4	1	dv1
................................Cuacucuacgcuuuugggaacg...............................................................	3	1	dv1
................................guacucuacgcuuuugggaacgU..............................................................	15	1	dv1
................................guacucuacgcuuuugggGacga..............................................................	1	1	dv1
................................guacucuacgcuuuugggCacga..............................................................	2	1	dv1
................................guacucuacgcuuuuggUaacga..............................................................	2	1	dv1
................................guacucuacgcuuuugggaacgC..............................................................	2	1	dv1
................................guacucuacgcuuuugggaaUga..............................................................	1	1	dv1
................................Uuacucuacgcuuuugggaacga..............................................................	5	1	dv1
................................guacAcuacgcuuuugggaacga..............................................................	1	1	dv1
................................guacucuacgcuuuugggaCcga..............................................................	1	1	dv1
................................guacucuacgcuuuugggaacga..............................................................	82	0	dv1
................................Cuacucuacgcuuuugggaacga..............................................................	2	1	dv1
................................guacucuacgcuuuugggaacgaU.............................................................	2	1	dv1
................................guacucuacgcuuuugggaacUag.............................................................	1	1	dv1
.................................uacucuacgcuuuuggga..................................................................	1	0	dv1
.................................uacucuacgcuuuugUgaa.................................................................	1	1	dv1
.................................uacucuacgcuuuugggaa.................................................................	9	0	dv1
.................................uacucuacgcuuuuggUaac................................................................	1	1	dv1
.................................uacucuacgcuuuugggaac................................................................	49	0	dv1
.................................uacucuacgcuuuuggUaacg...............................................................	5	1	dv1
.................................uacucuacgcuuuugggCacg...............................................................	4	1	dv1
.................................uacucuGcgcuuuugggaacg...............................................................	1	1	dv1
.................................uacucuacgcuuuugggaCcg...............................................................	4	1	dv1
.................................uacucuacgcuuuuUggaacg...............................................................	1	1	dv1
.................................uacucuacgcuuuugggUacg...............................................................	1	1	dv1
.................................uacucuacgcuuuugggaacU...............................................................	2	1	dv1
.................................uacucuacgcuuuugggGacg...............................................................	1	1	dv1
.................................uacucuacgcuuuugggaGcg...............................................................	2	1	dv1
.................................uacucuacgcuuuugUgaacg...............................................................	2	1	dv1
.................................Gacucuacgcuuuugggaacg...............................................................	1	1	dv1
.................................uacucuacgcuuuugggaacg...............................................................	266	0	dv1
.................................uacucuacgcuuuuCggaacg...............................................................	1	1	dv1
.................................uacucuacgcuuuugggUacga..............................................................	1	1	dv1
.................................uacucuacgcuuuugggaacAa..............................................................	1	1	dv1
.................................uacucCacgcuuuugggaacga..............................................................	3	1	dv1
.................................uacucuacgcuuuugggaaAga..............................................................	1	1	dv1
.................................uacCcuacgcuuuugggaacga..............................................................	2	1	dv1
.................................uaUucuacgcuuuugggaacga..............................................................	1	1	dv1
.................................uacucuaUgcuuuugggaacga..............................................................	1	1	dv1
.................................uacucuacgcuuuuUggaacga..............................................................	8	1	dv1
.................................uacucuacgcuuuuggUaacga..............................................................	23	1	dv1
.................................uaAucuacgcuuuugggaacga..............................................................	1	1	dv1
.................................uacucuacgcuuuugggaacUa..............................................................	2	1	dv1
.................................uacucuacgcuuAugggaacga..............................................................	1	1	dv1
.................................uacucuacgcuCuugggaacga..............................................................	1	1	dv1
.................................uacucuacgcuuuugggaGcga..............................................................	2	1	dv1
.................................uacucuacgcuuuugggaaUga..............................................................	3	1	dv1
.................................uacucuacgcuuuugCgaacga..............................................................	1	1	dv1
.................................uacucuacgAuuuugggaacga..............................................................	1	1	dv1
.................................uacucuacgcuuuugUgaacga..............................................................	24	1	dv1
.................................uacucuacgcCuuugggaacga..............................................................	3	1	dv1
.................................uacucuacgcuuuugggaCcga..............................................................	15	1	dv1
.................................Cacucuacgcuuuugggaacga..............................................................	1	1	dv1
```

```
                                dvi-miR-9699-5p
                                                                      dvi-miR-9699-3p
ugguacaggcacaacugggaacaggcacagccguacucuacgcuuuugggaacgagcucuuugacgcacucgguuccauugccguugaguagagcuguccugaugcauaucucaacg

.................................uacucuacgcuuuugggaacgU..............................................................   4     1     dv1
.................................uacucuacgcuuuuCggaacga..............................................................   2     1     dv1
.................................uacucuacCcuuuugggaacga..............................................................   2     1     dv1
.................................uacucuacgcuuuugggaUcga..............................................................   1     1     dv1
.................................Gacucuacgcuuuugggaacga..............................................................   2     1     dv1
.................................uacucuacgcuuuugggCacga..............................................................   33    1     dv1
.................................uacuAuacgcuuuugggaacga..............................................................   2     1     dv1
.................................uacucuacgcuuuugggaacga..............................................................   1407  0     dv1
.................................uacucuacgcuuuugggGacga..............................................................   4     1     dv1
.................................uacucuacgcuuuuggCaacga..............................................................   3     1     dv1
.................................uacucuGcgcuuuugggaacga..............................................................   1     1     dv1
.................................uacucuacgcuuuAgggaacga..............................................................   1     1     dv1
.................................uacucuacgUuuuugggaacga..............................................................   3     1     dv1
.................................uacucuacgcuuuugggaacgC..............................................................   22    1     dv1
.................................uacucuacgcuuuugggaacgaU.............................................................   8     1     dv1
.................................uacucuacgcuuuugggaacgaA.............................................................   8     1     dv1
.................................uacucuacgcuuuugggaacCag.............................................................   1     1     dv1
.................................uacucuacgcuuuugggaacgaC.............................................................   11    1     dv1
.................................uacucuacgcuuuugggaacgag.............................................................   4     0     dv1
.................................uacucuacgcuuuugggaacgaCc............................................................   1     1     dv1
.................................uacucuacgcuuuugggaacgaAc............................................................   1     1     dv1
.................................uacucuacgcuuuugggaacgaUc............................................................   2     1     dv1
..................................acucuacgcuuuugggaacg...............................................................   2     0     dv1
..................................acucuacgcuuuugggaacga..............................................................   5     0     dv1
..................................acucuacgcuuuugggaacgag.............................................................   8     0     dv1
..................................acucuacgcuuuugggaacgagU............................................................   1     1     dv1
...................................cucuacgcuuuugggaacg...............................................................   2     0     dv1
....................................ucuacgcuuuugggaacg...............................................................   1     0     dv1
....................................ucuacgcuuuugggaacga..............................................................   1     0     dv1
....................................ucuacgcuuuugggaacgag.............................................................   1     0     dv1
.....................................cuacgcuuuugggaacga..............................................................   3     0     dv1
.....................................................................ucgguuccauugccguugag............................   1     0     dv1
.....................................................................ucgguuccauugccguugagu...........................   1     0     dv1
.....................................................................ucgguuccauugcUguugaguag.........................   1     1     dv1
......................................................................cgguuccauugccguugaguag.........................   2     0     dv1
......................................................................cgguuccauugccguugaguaga........................   2     0     dv1
.......................................................................gguuccauugccguugag............................   6     0     dv1
.......................................................................gguuccauugccguugagu...........................   7     0     dv1
.......................................................................gguuccauugccguugaUua..........................   1     1     dv1
.......................................................................Uguuccauugccguugagua..........................   1     1     dv1
.......................................................................gguuccauugccguugagua..........................   14    0     dv1
.......................................................................gguuccauugccguugaguCg.........................   20    1     dv1
.......................................................................gguuccauugccAuugaguag.........................   1     1     dv1
.......................................................................gguuccauugccguugaCuag.........................   1     1     dv1
.......................................................................gguuccauugccguugaguUg.........................   1     1     dv1
.......................................................................gguuccauugccguugaguGg.........................   1     1     dv1
.......................................................................gguuccauugccguCgaguag.........................   2     1     dv1
.......................................................................gguuccauugccguugCguag.........................   11    1     dv1
.......................................................................gguuccauugccguugaguag.........................   637   0     dv1
.......................................................................gguucUauugccguugaguag.........................   2     1     dv1
.......................................................................Cguuccauugccguugaguag.........................   1     1     dv1
.......................................................................gguuccauugccguugaguaA.........................   4     1     dv1
.......................................................................gguuccauugccgAugaguag.........................   1     1     dv1
.......................................................................gguuccauCgccguugaguag.........................   1     1     dv1
.......................................................................gguuccauugUcguugaguag.........................   1     1     dv1
.......................................................................gguuccauugccguugaguaU.........................   5     1     dv1
.......................................................................gguuccauugccguugGguag.........................   2     1     dv1
.......................................................................gguuccauugccUuugaguag.........................   3     1     dv1
.......................................................................Aguuccauugccguugaguag.........................   1     1     dv1
.......................................................................gguCccauugccguugaguag.........................   1     1     dv1
.......................................................................gguuccaCugccguugaguag.........................   1     1     dv1
.......................................................................gguuccauugccguuUaguag.........................   3     1     dv1
.......................................................................gUuuccauugccguugaguag.........................   4     1     dv1
.......................................................................gguuccauugccguugaUuag.........................   4     1     dv1
.......................................................................Uguuccauugccguugaguag.........................   32    1     dv1
.......................................................................gguuccauugAcguugaguaga........................   1     1     dv1
.......................................................................Uguuccauugccguugaguaga........................   43    1     dv1
.......................................................................gguuccauCgccguugaguaga........................   4     1     dv1
.......................................................................gguuccauugccguugaguaga........................   870   0     dv1
```

```
                                dvi-miR-9699-5p
                                                                       dvi-miR-9699-3p
ugguacaggcacaacugggaacaggcacagccguacucuacgcuuuugggaacgagcucuuugacgcacucgguuccauugccguugaguagagcuguccugaugcauaucucaacg

.......................................................................gguuccauugccguugaguaCa........................     1     1     dv1
.......................................................................gguuccauugccguugaguaUa........................     2     1     dv1
.......................................................................gguuccauugccguugaguCga........................     21    1     dv1
.......................................................................gguuccauugcUguugaguaga........................     2     1     dv1
.......................................................................gguuccauugccguuUaguaga........................     2     1     dv1
.......................................................................gUuuccauugccguugaguaga........................     1     1     dv1
.......................................................................gguuccauugccguugaUuaga........................     2     1     dv1
.......................................................................gguuccauugccgCugaguaga........................     1     1     dv1
.......................................................................gguuccauugccguuCaguaga........................     1     1     dv1
.......................................................................gguuccCuugccguugaguaga........................     1     1     dv1
.......................................................................gguuccauuCccguugaguaga........................     3     1     dv1
.......................................................................gguuccauuUccguugaguaga........................     1     1     dv1
.......................................................................gguuccauugccguCgaguaga........................     1     1     dv1
.......................................................................gguuccauugccguugagGaga........................     1     1     dv1
.......................................................................gguuccauugccguugagAaga........................     2     1     dv1
.......................................................................gguuccauugccguugaguagC........................     24    1     dv1
.......................................................................gguuUcauugccguugaguaga........................     1     1     dv1
.......................................................................gguuccauugccguugGguaga........................     2     1     dv1
.......................................................................gguuccauugccguugCguaga........................     17    1     dv1
.......................................................................gguuccauugccguugagCaga........................     2     1     dv1
.......................................................................Aguuccauugccguugaguaga........................     1     1     dv1
.......................................................................gguuccauugccguugaguagU........................     1     1     dv1
.......................................................................gguuccauugccguugaguGga........................     1     1     dv1
.......................................................................gguuccaCugccguugaguaga........................     1     1     dv1
.......................................................................gguucAauugccguugaguaga........................     2     1     dv1
.......................................................................gguuccauugccUuugaguaga........................     3     1     dv1
.......................................................................gguuccauugccCuugaguaga........................     1     1     dv1
.......................................................................gguuccauugccguugaguagag.......................     29    0     dv1
.......................................................................gguuccauugccguugCguagag.......................     5     1     dv1
.......................................................................gguuccauugccguugaguagCg.......................     2     1     dv1
.......................................................................gguCccauugccguugaguagag.......................     1     1     dv1
.......................................................................gguuccauugccguugaguagaA.......................     4     1     dv1
.......................................................................Uguuccauugccguugaguagag.......................     1     1     dv1
.......................................................................gguuccauugccguugaguagaC.......................     1     1     dv1
.......................................................................gguuccauugccguugaguagaU.......................     1     1     dv1
.......................................................................gguuccauugccguugaguagaCc......................     3     1     dv1
.......................................................................gguuccauugccguugaguagagU......................     2     1     dv1
.......................................................................gguuccauugccguugaguagagA......................     4     1     dv1
........................................................................guuccauugccguugagua..........................     1     0     dv1
........................................................................guuccauugccguugCguag.........................     1     1     dv1
........................................................................guuccauugccguugaUuag.........................     1     1     dv1
........................................................................guuccauugccguugaguag.........................     27    0     dv1
........................................................................guuccauugccgCugaguag.........................     1     1     dv1
........................................................................guuccauuUccguugaguaga........................     1     1     dv1
........................................................................guuccauugccguugCguaga........................     4     1     dv1
........................................................................guuccauugccgCugaguaga........................     1     1     dv1
........................................................................guuccauugccgGugaguaga........................     1     1     dv1
........................................................................guuccauugccguugaUuaga........................     1     1     dv1
........................................................................guuccauCgccguugaguaga........................     1     1     dv1
........................................................................guuccauugccguugGguaga........................     1     1     dv1
........................................................................guuccauugccguugaguaga........................     187   0     dv1
........................................................................guuccauugccguugaguagC........................     7     1     dv1
........................................................................Uuuccauugccguugaguaga........................     11    1     dv1
........................................................................guuccauugccguuUaguaga........................     1     1     dv1
........................................................................guuccauugccguugagCaga........................     1     1     dv1
........................................................................guuccauugccguugaguCga........................     4     1     dv1
........................................................................guuccauugccguuAaguaga........................     1     1     dv1
........................................................................guuccauugccguugaCuagag.......................     1     1     dv1
........................................................................guuccauugccgGugaguagag.......................     1     1     dv1
........................................................................guuccaCugccguugaguagag.......................     1     1     dv1
........................................................................guCccauugccguugaguagag.......................     1     1     dv1
........................................................................gCuccauugccguugaguagag.......................     2     1     dv1
........................................................................guuccauugccguugagCagag.......................     1     1     dv1
........................................................................guuccauugccguugaguagag.......................     182   0     dv1
........................................................................guuccauugccguugGguagag.......................     2     1     dv1
........................................................................Uuuccauugccguugaguagag.......................     9     1     dv1
........................................................................guuccauuUccguugaguagag.......................     2     1     dv1
........................................................................guuccauugccguugaUuagag.......................     1     1     dv1
........................................................................guuccauugccguugaguCgag.......................     9     1     dv1
```

```
                                dvi-miR-9699-5p
                                                                       dvi-miR-9699-3p
ugguacaggcacaacugggaacaggcacagccguacucuacgcuuuugggaacgagcucuuugacgcacucgguuccauugccguugaguagagcuguccugaugcauaucucaacg

........................................................................guuccauugccguugaguaUag.......................   3          1          dv1
........................................................................guuccauugccUuugaguagag.......................   1          1          dv1
........................................................................guuccauugccguugaguaCag.......................   1          1          dv1
........................................................................guuccauugccguugagGagag.......................   1          1          dv1
........................................................................guuccauugccguugCguagag.......................   7          1          dv1
........................................................................guuccauugccguugaguGgag.......................   1          1          dv1
........................................................................guuccauugccguugaguagCg.......................   5          1          dv1
........................................................................guuccauugccguugaguagaCc......................   1          1          dv1
........................................................................guuccauugccguugaguagagU......................   18         1          dv1
........................................................................guuccauugccguugaguagagA......................   19         1          dv1
........................................................................guuccauugccguugaguagagUu.....................   1          1          dv1
.........................................................................uuccauugccguugaguag.........................   1          0          dv1
.........................................................................uuccauugccguugaguaga........................   5          0          dv1
.........................................................................uuccauugccguugaguagag.......................   1          0          dv1
.........................................................................uuccauugcUguugaguagag.......................   1          1          dv1
.........................................................................uuccauugccguugaguagagU......................   1          1          dv1
.........................................................................uuccauugccguugaguagagc......................   2          0          dv1
..........................................................................uccauugccguugaguag.........................   2          0          dv1
..........................................................................uccauugccguugaguaga........................   3          0          dv1
..........................................................................uccauugccguugGguaga........................   1          1          dv1
...........................................................................ccauugccguugaguaga........................   2          0          dv1
...........................................................................ccauugccguugaguagag.......................   1          0          dv1

...............................cguacucuacgcuuuuggga..................................................................   1          0          arg
...............................cguacucuacgcuuuugggaacg...............................................................   8          0          arg
................................guacucuacgcuuuuggga..................................................................   4          0          arg
................................guacucuacgcuuuugggU..................................................................   1          1          arg
................................guacucuacgcuuuugggaa.................................................................   8          0          arg
................................guacucGacgcuuuugggaac................................................................   1          1          arg
................................guacucuacgcuuuuUggaac................................................................   1          1          arg
................................guacucuacgcuuuugggaac................................................................   22         0          arg
................................guacucuacgcuuuugggaaA................................................................   2          1          arg
................................gGacucuacgcuuuugggaacg...............................................................   4          1          arg
................................guacucCacgcuuuugggaacg...............................................................   1          1          arg
................................guacucuacgcuuuuggCaacg...............................................................   2          1          arg
................................guacAcuacgcuuuugggaacg...............................................................   2          1          arg
................................guacuAuacgcuuuugggaacg...............................................................   1          1          arg
................................guacucuacgUuuuugggaacg...............................................................   3          1          arg
................................guUcucuacgcuuuugggaacg...............................................................   1          1          arg
................................guacucuacAcuuuugggaacg...............................................................   3          1          arg
................................guacucuacgcuuuugggGacg...............................................................   5          1          arg
................................guacucuacgcuuCugggaacg...............................................................   1          1          arg
................................guacucuacgcuuuugggaCcg...............................................................   3          1          arg
................................guacucuacUcuuuugggaacg...............................................................   1          1          arg
................................guacucuaAgcuuuugggaacg...............................................................   1          1          arg
................................guacucuacgcuuuuAggaacg...............................................................   3          1          arg
................................guacucuacgcuuuuCggaacg...............................................................   1          1          arg
................................guGcucuacgcuuuugggaacg...............................................................   2          1          arg
................................guacucuacgcuuuugggaUcg...............................................................   1          1          arg
................................guacucuacgGuuuugggaacg...............................................................   1          1          arg
................................guacucuacgcuuuugggUacg...............................................................   4          1          arg
................................guacucuacgcuuuugggaacC...............................................................   2          1          arg
................................guacucuacgcuuuCgggaacg...............................................................   3          1          arg
................................guacucuaUgcuuuugggaacg...............................................................   2          1          arg
................................guacGcuacgcuuuugggaacg...............................................................   1          1          arg
................................guacucuacgcuuuugCgaacg...............................................................   1          1          arg
................................guacucuacgcuuuugggaacU...............................................................   7          1          arg
................................guacucuacgcuuuugggaacg...............................................................   1679       0          arg
................................guacucuacgcuCuugggaacg...............................................................   1          1          arg
................................guacucuacgcuuuuUggaacg...............................................................   4          1          arg
................................guacucuacgcuuuuggUaacg...............................................................   4          1          arg
................................guacucuacgcuuuuggAaacg...............................................................   1          1          arg
................................guacucuacgcCuuugggaacg...............................................................   2          1          arg
................................guacucuacgcuuuugggaacA...............................................................   2          1          arg
................................gCacucuacgcuuuugggaacg...............................................................   2          1          arg
................................guacucuacgcuuGugggaacg...............................................................   1          1          arg
................................guacucuacgcuuuugUgaacg...............................................................   6          1          arg
................................guacucuacgcuuuugggaGcg...............................................................   2          1          arg
................................Cuacucuacgcuuuugggaacg...............................................................   1          1          arg
```

dvi-miR-9699-5p

dvi-miR-9699-3p

ugguacaggcacaacugggaacaggcacagccguacucuacgcuuuugggaacgagcucuuugacgcacucgguuccauugccguugaguagagcuguccugaugcauaucucaacg

................................Uuacucuacgcuuuugggaacg...	4	1	arg
................................guacucuacgcuuuugggCacg...	2	1	arg
................................guacucuacCcuuuugggaacg...	1	1	arg
................................guacuUuacgcuuuugggaacg...	4	1	arg
................................guacucAacgcuuuugggaacg...	1	1	arg
................................guacucuacgcuuuugggaaAg...	2	1	arg
................................guacucuacgcuuuugggGacga..	1	1	arg
................................guacucuacgcuuuugggaacgC..	1	1	arg
................................guacucuacgcCuuugggaacga..	1	1	arg
................................guacucuacgcuuuugUgaacga..	2	1	arg
................................guacucuacgcuuuugggaacgU..	31	1	arg
................................guacucuacgcuuuugggaacga..	57	0	arg
.................................uacucuacgcuuuuggga..	3	0	arg
.................................uacucuacgcuuuugggaa...	3	0	arg
.................................uacucuacgcuuuugggaac..	33	0	arg
.................................uacucuacgcuuuuggUaac..	1	1	arg
.................................uacucuGcgcuuuugggaacg...	1	1	arg
.................................uacucuacgcuuuugggaUcg...	1	1	arg
.................................uacucuacgcuuuugggaCcg...	2	1	arg
.................................uacAcuacgcuuuugggaacg...	2	1	arg
.................................uacucuacgcuuCugggaacg...	1	1	arg
.................................uacucuacgcuuuuggUaacg...	3	1	arg
.................................uacucuacgcuuuuggAaacg...	1	1	arg
.................................uUcucuacgcuuuugggaacg...	1	1	arg
.................................uacucuacgcuuuugggaacg...	467	0	arg
.................................uacucuacgcCuuugggaacg...	1	1	arg
.................................uacucuacgcuuuugggaaUg...	1	1	arg
.................................uacuAuacgcuuuugggaacg...	1	1	arg
.................................uacucuacAcuuuugggaacg...	2	1	arg
.................................uaAucuacgcuuuugggaacg...	1	1	arg
.................................uGcucuacgcuuuugggaacg...	2	1	arg
.................................uaUucuacgcuuuugggaacg...	1	1	arg
.................................uacucuacgcuuuugggaacC...	2	1	arg
.................................uacucuacgcuuuugggaGcg...	4	1	arg
.................................uacucuacgcuuuugggGacg...	2	1	arg
.................................Gacucuacgcuuuugggaacg...	1	1	arg
.................................uacucuacgcuuuugggaacA...	1	1	arg
.................................uacucuacgcuuuugggaacga..	547	0	arg
.................................uacucuacCcuuuugggaacga..	1	1	arg
.................................uacucuacgcCuuugggaacga..	2	1	arg
.................................uacucuacgcuuuugCgaacga..	1	1	arg
.................................uacucuacgcuuCugggaacga..	2	1	arg
.................................uacucuacgcuAuugggaacga..	1	1	arg
.................................uacucuacgcuuuugggCacga..	2	1	arg
.................................uacucuacgcuuuugggaacUa..	1	1	arg
.................................uacucuacgcuuuugggaacgU..	10	1	arg
.................................uacucuacgcuuuugggaacgG..	1	1	arg
.................................uacucuacgAuuuugggaacga..	1	1	arg
.................................uacucuacgcuuuugggaGcga..	1	1	arg
.................................uacucuacgcuCuugggaacga..	2	1	arg
.................................uacucuacgcuuuuggUaacga..	2	1	arg
.................................uacucuacgcuuuugggaCcga..	1	1	arg
.................................uaUucuacgcuuuugggaacga..	1	1	arg
.................................uacucuacgcuuuuUggaacga..	2	1	arg
.................................uacAcuacgcuuuugggaacga..	1	1	arg
.................................uacCcuacgcuuuugggaacga..	1	1	arg
.................................uacucuacgcuuuugggaacAa..	1	1	arg
.................................uacucuaUgcuuuugggaacga..	1	1	arg
.................................uacucuacgcuuuuggAaacga..	1	1	arg
.................................Cacucuacgcuuuugggaacga..	1	1	arg
.................................uacucuacgcuuuugggCacgag...	1	1	arg
.................................uacucuacgcuuuugggaacgaA...	5	1	arg
.................................uacucuacgcuuuugggaacgaU...	3	1	arg
.................................uacucuacgcuuuugggaacgaC...	1	1	arg
.................................uacucuacgcuuuugggaacgag...	6	0	arg
.................................uacucuacgcuuuugggaacgagc..	1	0	arg
.................................uacucuacgcuuuugggaacgaUc..	2	1	arg
..................................acucuacgcuuuugggCacg...	1	1	arg
..................................acucuacgcuuuugggaacg...	2	0	arg

```
                                                dvi-miR-9699-5p
                                                                                       dvi-miR-9699-3p
ugguacaggcacaacugggaacaggcacagccguacucuacgcuuuugggaacgagcucuuugacgcacucgguuccauugccguugaguagagcuguccugaugcauaucucaacg

..................................acucuacgcuuuuUggaacg...............................................................  1    1    arg
..................................acucuacgcuuuugggaacgU..............................................................  1    1    arg
..................................aAucuacgcuuuugggaacgag.............................................................  1    1    arg
..................................acucuacgcuuuugggaacgag.............................................................  22   0    arg
..................................acucuacgcuuuugggCacgag.............................................................  1    1    arg
..................................acucuacgcuuuugggaacUag.............................................................  1    1    arg
..................................acucuacgcuuuugggUacgag.............................................................  2    1    arg
....................................................................cucgguuccauugccguugaguag.........................  1    0    arg
.....................................................................ucgguuccauugccguugaguag.........................  1    0    arg
......................................................................cgguuccauugccguugagu...........................  1    0    arg
......................................................................cgguuccauugccguugaguag.........................  2    0    arg
.......................................................................gguuccauugccguugagu...........................  13   0    arg
.......................................................................gguuccauugccguugagua..........................  10   0    arg
.......................................................................gguuccauugccguugaguC..........................  2    1    arg
.......................................................................gguuccauugccguugaguU..........................  1    1    arg
.......................................................................gguuccauugccguugaguag.........................  473  0    arg
.......................................................................gguuccauugccguCgaguag.........................  1    1    arg
.......................................................................gguuccauuAccguugaguag.........................  1    1    arg
.......................................................................gguuccauuCccguugaguag.........................  1    1    arg
.......................................................................ggAuccauugccguugaguag.........................  1    1    arg
.......................................................................gguuccauugccguuAaguag.........................  1    1    arg
.......................................................................gguuccauugUcguugaguag.........................  1    1    arg
.......................................................................gguuccauugccUuugaguag.........................  1    1    arg
.......................................................................gguuccauugccgCugaguag.........................  1    1    arg
.......................................................................gguuccauugccguugaguaA.........................  1    1    arg
.......................................................................gguuccauugccguugagCag.........................  1    1    arg
.......................................................................gguuccauugccguugaguUg.........................  2    1    arg
.......................................................................gguuccauugccguugaguaU.........................  1    1    arg
.......................................................................gguuUcauugccguugaguag.........................  1    1    arg
.......................................................................gguucUauugccguugaguag.........................  1    1    arg
.......................................................................gguuccauugccgGugaguag.........................  1    1    arg
.......................................................................ggGuccauugccguugaguag.........................  2    1    arg
.......................................................................gguuccauugAcguugaguag.........................  3    1    arg
.......................................................................gUuuccauugccguugaguag.........................  4    1    arg
.......................................................................gguuccauugccguugaguCg.........................  2    1    arg
.......................................................................gCuuccauugccguugaguag.........................  1    1    arg
.......................................................................gguuccauugccguugGguag.........................  1    1    arg
.......................................................................gguuccauugccguugaCuag.........................  2    1    arg
.......................................................................gguuccauugccguugaAuag.........................  1    1    arg
.......................................................................gguuccauugccguuUaguag.........................  1    1    arg
.......................................................................gguuccGuugccguugaguag.........................  1    1    arg
.......................................................................gguuccauugccguugaUuag.........................  1    1    arg
.......................................................................gguuccauugcUguugaguag.........................  3    1    arg
.......................................................................gguuUcauugccguugaguaga........................  1    1    arg
.......................................................................gguuccauugccguugaguagG........................  3    1    arg
.......................................................................gguuccauugccguugaUuaga........................  1    1    arg
.......................................................................gguuccauuCccguugaguaga........................  1    1    arg
.......................................................................gguuccauugccguugaguGga........................  3    1    arg
.......................................................................gguuccauugccguuAaguaga........................  2    1    arg
.......................................................................gguuccauugccguCgaguaga........................  5    1    arg
.......................................................................gguuccauugccguugaCuaga........................  1    1    arg
.......................................................................gguuccauugccguugaguCga........................  5    1    arg
.......................................................................gguuccauugccguugagCaga........................  4    1    arg
.......................................................................gguuccauugAcguugaguaga........................  1    1    arg
.......................................................................gguuccauCgccguugaguaga........................  1    1    arg
.......................................................................gguuccauugccguugCguaga........................  3    1    arg
.......................................................................gguuccauugccCuugaguaga........................  2    1    arg
.......................................................................gguuccauugccguugagGaga........................  2    1    arg
.......................................................................gguCccauugccguugaguaga........................  4    1    arg
.......................................................................gAuuccauugccguugaguaga........................  2    1    arg
.......................................................................gguuccauugccguugaguUga........................  1    1    arg
.......................................................................gguuccauugccgAugaguaga........................  1    1    arg
.......................................................................gguuccauugccguugaguaUa........................  5    1    arg
.......................................................................ggAuccauugccguugaguaga........................  1    1    arg
.......................................................................gguuccauugccAuugaguaga........................  1    1    arg
.......................................................................gguuccauugcUguugaguaga........................  4    1    arg
.......................................................................Uguuccauugccguugaguaga........................  1    1    arg
.......................................................................gguuccauugccguugaguagC........................  5    1    arg
.......................................................................gguuccaCugccguugaguaga........................  1    1    arg
```

```
                                dvi-miR-9699-5p
                                                                       dvi-miR-9699-3p
ugguacaggcacaacugggaacaggcacagccguacucuacgcuuuugggaacgagcucuuugacgcacucgguuccauugccguugaguagagcuguccugaugcauaucucaacg

.......................................................................gguuccauugccUuugaguaga........................   1      1      arg
.......................................................................ggCuccauugccguugaguaga........................   4      1      arg
.......................................................................gguuccauugccguugGguaga........................   3      1      arg
.......................................................................gguuccauuUccguugaguaga........................   1      1      arg
.......................................................................gguuccauugccguugaguaga........................   1400   0      arg
.......................................................................gguuccauugccguugaguagU........................   6      1      arg
.......................................................................gguuccGuugccguugaguaga........................   2      1      arg
.......................................................................gguuccauugccgGugaguaga........................   1      1      arg
.......................................................................gguuccauugccguugUguaga........................   5      1      arg
.......................................................................gguuccauugccgCugaguaga........................   1      1      arg
.......................................................................ggGuccauugccguugaguaga........................   1      1      arg
.......................................................................gUuuccauugccguugaguaga........................   6      1      arg
.......................................................................Aguuccauugccguugaguaga........................   4      1      arg
.......................................................................gguuAcauugccguugaguaga........................   2      1      arg
.......................................................................Cguuccauugccguugaguagag.......................   1      1      arg
.......................................................................gguuccauugccguugGguagag.......................   1      1      arg
.......................................................................gguuccauugccguugaguagaU.......................   4      1      arg
.......................................................................gguuccauugccguugaguagaA.......................   13     1      arg
.......................................................................gguCccauugccguugaguagag.......................   1      1      arg
.......................................................................gguuccauugccguugaguagag.......................   33     0      arg
.......................................................................gguuccauugccguugaguagaCc......................   1      1      arg
........................................................................guuccauugccguugaguag.........................   14     0      arg
........................................................................guuccauugccUuugaguag.........................   1      1      arg
........................................................................guuccauugccguugaguagG........................   2      1      arg
........................................................................guuccauugccguugaguagU........................   1      1      arg
........................................................................guuccauugccgCugaguaga........................   1      1      arg
........................................................................guuccauugccguCgaguaga........................   1      1      arg
........................................................................guuccauugccguugaCuaga........................   1      1      arg
........................................................................guuccauugccguugCguaga........................   2      1      arg
........................................................................Uuuccauugccguugaguaga........................   2      1      arg
........................................................................guuccauugccguugGguaga........................   1      1      arg
........................................................................guuccauugccguugaguaga........................   169    0      arg
........................................................................guucUauugccguugaguaga........................   1      1      arg
........................................................................gAuccauugccguugaguaga........................   1      1      arg
........................................................................guuccauugccgGugaguagag.......................   1      1      arg
........................................................................guuccauugccguugaguagag.......................   198    0      arg
........................................................................gCuccauugccguugaguagag.......................   1      1      arg
........................................................................guuccauuAccguugaguagag.......................   1      1      arg
........................................................................guuccauugccguugaguaUag.......................   5      1      arg
........................................................................guuccauugccguugaguUgag.......................   1      1      arg
........................................................................gGuccauugccguugaguagag.......................   1      1      arg
........................................................................guuccGuugccguugaguagag.......................   1      1      arg
........................................................................guuccauugccguuUaguagag.......................   1      1      arg
........................................................................Uuuccauugccguugaguagag.......................   2      1      arg
........................................................................guuccauugccguAgaguagag.......................   1      1      arg
........................................................................guuccauugccguugaguagaA.......................   7      1      arg
........................................................................guuccauugccguugaguagaU.......................   1      1      arg
........................................................................guucUauugccguugaguagag.......................   1      1      arg
........................................................................guuccauugccguugaguagagU......................   14     1      arg
........................................................................guuccauugccguugaguagagA......................   9      1      arg
........................................................................guuccauugccguugaguagagAu.....................   1      1      arg
.........................................................................uuccauugccguugaguag.........................   1      0      arg
.........................................................................uuccauugccguugaguaga........................   4      0      arg
.........................................................................uuccauugccguugaguagag.......................   2      0      arg
.........................................................................uuccauugccguugaguagagc......................   2      0      arg
.........................................................................uuccauugccguugaguagagU......................   2      1      arg
..........................................................................uccauugccguugaguaga........................   3      0      arg
...........................................................................ccauugccguugaguaga........................   2      0      arg
............................................................................cauugccguugaguagag.......................   4      0      arg

.........cacaacugggaacaggcacagcc.....................................................................................   1      0      dv9
...............................cguacucuacgcuuuugggaa.................................................................   3      0      dv9
...............................cguacucuacgcuuuugggaacA...............................................................   2      1      dv9
...............................cguacucuacgcuuuugggaacg...............................................................   1      0      dv9
................................guacucuacgcuuuuggga..................................................................   12     0      dv9
................................guacucuacgcuuuugUgaa.................................................................   1      1      dv9
................................guacucuacgcuuuugggCa.................................................................   1      1      dv9
................................guacucuacgcuuuugggaa.................................................................   13     0      dv9
................................guacucuacgcuuuuggUaa.................................................................   1      1      dv9
```

```
                                dvi-miR-9699-5p
                                                                       dvi-miR-9699-3p
ugguacaggcacaacugggaacaggcacagccguacucuacgcuuuugggaacgagcucuuugacgcacucgguuccauugccguugaguagagcuguccugaugcauaucucaacg

................................guacucuacgcuuuugggaUc................................................................   3      1     dv9
................................guacucuacgcuuuugUgaac................................................................   2      1     dv9
................................Uuacucuacgcuuuugggaac................................................................   4      1     dv9
................................guacucuacgcuuuugggaac................................................................   44     0     dv9
................................guacucuacgcuuuugggCac................................................................   2      1     dv9
................................guacucuacgcuuuugggaaU................................................................   2      1     dv9
................................guacucuacgcuuuuggCaacg...............................................................   4      1     dv9
................................guacucuacgcuuuugggaUcg...............................................................   1      1     dv9
................................guUcucuacgcuuuugggaacg...............................................................   1      1     dv9
................................guacucuacAcuuuugggaacg...............................................................   1      1     dv9
................................guacucuacgcuCuugggaacg...............................................................   5      1     dv9
................................guacucuacgcuuuCgggaacg...............................................................   1      1     dv9
................................guacucCacgcuuuugggaacg...............................................................   1      1     dv9
................................guacucuacUcuuuugggaacg...............................................................   2      1     dv9
................................Uuacucuacgcuuuugggaacg...............................................................   120    1     dv9
................................guacucuacgcuuuugggaaAg...............................................................   2      1     dv9
................................guacucuacgcuuuugggGacg...............................................................   6      1     dv9
................................guacucuacgcuuuugggaacU...............................................................   19     1     dv9
................................guacucuacgcuGuugggaacg...............................................................   1      1     dv9
................................guacucuacgcuuuugggaacA...............................................................   1      1     dv9
................................guacuUuacgcuuuugggaacg...............................................................   1      1     dv9
................................guacCcuacgcuuuugggaacg...............................................................   3      1     dv9
................................guacucuCcgcuuuugggaacg...............................................................   2      1     dv9
................................guacucuGcgcuuuugggaacg...............................................................   3      1     dv9
................................guacucuaUgcuuuugggaacg...............................................................   4      1     dv9
................................guacucuacgcuuuugggaaUg...............................................................   3      1     dv9
................................guacucuacgcuuuugggaacC...............................................................   12     1     dv9
................................guacucuacgcuuuuggUaacg...............................................................   29     1     dv9
................................guacucuacgcuuuuUggaacg...............................................................   4      1     dv9
................................guacucuacgcuuuugggaCcg...............................................................   77     1     dv9
................................guacucuacgcuuuugggCacg...............................................................   75     1     dv9
................................guacucuacgcuuuugCgaacg...............................................................   1      1     dv9
................................guaUcucuacgcuuuugggaacg...............................................................   1      1     dv9
................................guacucuacgcuuuugggaGcg...............................................................   6      1     dv9
................................guacucuacgcuuuugUgaacg...............................................................   15     1     dv9
................................Auacucuacgcuuuugggaacg...............................................................   1      1     dv9
................................guacuAuacgcuuuugggaacg...............................................................   1      1     dv9
................................guacucuacgcuuuugggaaGg...............................................................   2      1     dv9
................................guacucuacgcuuuGgggaacg...............................................................   2      1     dv9
................................guacucuacgcuuuugggaacg...............................................................   2204   0     dv9
................................guGcucuacgcuuuugggaacg...............................................................   1      1     dv9
................................guacucuacgUuuuugggaacg...............................................................   1      1     dv9
................................guacGcuacgcuuuugggaacg...............................................................   1      1     dv9
................................guacucuacgcuuuuCggaacg...............................................................   1      1     dv9
................................guacucuacgcuuuuggAaacg...............................................................   1      1     dv9
................................guacucuacgcCuuugggaacg...............................................................   3      1     dv9
................................guacucuacgcuuCugggaacg...............................................................   1      1     dv9
................................guaAucuacgcuuuugggaacg...............................................................   2      1     dv9
................................gCacucuacgcuuuugggaacg...............................................................   1      1     dv9
................................guacucuacgcuuuCgggaacga..............................................................   1      1     dv9
................................guacucuacgcAuuugggaacga..............................................................   1      1     dv9
................................guacucuacgcuuuugggaaGga..............................................................   1      1     dv9
................................guacucuacgcuuuugggaacga..............................................................   127    0     dv9
................................gGacucuacgcuuuugggaacga..............................................................   1      1     dv9
................................guacucuacgcuuuugggaacCa..............................................................   1      1     dv9
................................guacucuacgcuuuuggUaacga..............................................................   1      1     dv9
................................Uuacucuacgcuuuugggaacga..............................................................   16     1     dv9
................................guacucuacgcuuCugggaacga..............................................................   1      1     dv9
................................guacucuacgcuuuugggCacga..............................................................   5      1     dv9
................................guacucuacgcuuuugggaacgC..............................................................   1      1     dv9
................................guacucuacgcuuuugggaCcga..............................................................   1      1     dv9
................................guacCcuacgcuuuugggaacga..............................................................   1      1     dv9
................................guacucuacgcuuuugggaacgU..............................................................   16     1     dv9
................................guacucuacgcuuuuggCaacga..............................................................   1      1     dv9
................................guacucuacgcuuuugggaacgGg.............................................................   1      1     dv9
................................guacucuacgcuuuugggaacgaA.............................................................   1      1     dv9
................................guacucuacgcuuuugggaacgaU.............................................................   1      1     dv9
.................................uacucuacgcuuuuggga..................................................................   2      0     dv9
.................................uacucuacgcuuuugggaa.................................................................   8      0     dv9
```

dvi-miR-9699-5p

dvi-miR-9699-3p

ugguacaggcacaacugggaacaggcacagccguacucuacgcuuuugggaacgagcucuuugacgcacucgguuccauugccguugaguagagcuguccugaugcauaucucaacg

.................................uacucuacgcuuCugggaac..	1	1	dv9
.................................Gacucuacgcuuuugggaac..	1	1	dv9
.................................uacucuacgcuuuuggUaac..	1	1	dv9
.................................uacucuacgcuuuugggaac..	56	0	dv9
.................................uacucuacgcuuuugggCac..	3	1	dv9
.................................uacucuacgcuuuugggaCc..	1	1	dv9
.................................uacucuacgcuCuugggaac..	1	1	dv9
.................................uacucuacgcuuuugUgaac..	1	1	dv9
.................................uGcucuacgcuuuugggaacg...	1	1	dv9
.................................uacucuacgcuuuugggaaUg...	2	1	dv9
.................................uacucuacgcuuuuCggaacg...	1	1	dv9
.................................uacucuacgcuuuuggUaacg...	4	1	dv9
.................................uacucuacgcuuuCgggaacg...	1	1	dv9
.................................uacucuacgcuuuugggaacC...	1	1	dv9
.................................Gacucuacgcuuuugggaacg...	1	1	dv9
.................................Aacucuacgcuuuugggaacg...	1	1	dv9
.................................uacucuacgcuuuugggaacU...	2	1	dv9
.................................uacucuacgcuuuugggaUcg...	1	1	dv9
.................................uacucuacgcuuuugggaacg...	235	0	dv9
.................................uacucuacgcuuuugggaacA...	1	1	dv9
.................................uacucuacgcuuuugUgaacg...	2	1	dv9
.................................uacucuacgcuuuugggCacg...	5	1	dv9
.................................uacucuacgcuuuugggaCcg...	6	1	dv9
.................................uacucuacgcuuuugggCacga..	35	1	dv9
.................................uacucuacgcuuuuggAaacga..	1	1	dv9
.................................uacucuacgUuuuugggaacga..	2	1	dv9
.................................uacucuacgcuuuugAgaacga..	1	1	dv9
.................................uacucuacgcuGuugggaacga..	1	1	dv9
.................................uacucuacgcuuuugggaaAga..	1	1	dv9
.................................uacucuacgcuCuugggaacga..	2	1	dv9
.................................uacucuacgcuuuugggaaUga..	3	1	dv9
.................................Aacucuacgcuuuugggaacga..	1	1	dv9
.................................uacCcuacgcuuuugggaacga..	1	1	dv9
.................................uacucuGcgcuuuugggaacga..	1	1	dv9
.................................uacucuacgcuuuugggaacUa..	11	1	dv9
.................................uaAucuacgcuuuugggaacga..	2	1	dv9
.................................uacucuacgcuuuuggUaacga..	18	1	dv9
.................................uacucuacgcuuuugggaacAa..	7	1	dv9
.................................uacucuacgcuuuCgggaacga..	1	1	dv9
.................................uacucuacgcCuuugggaacga..	5	1	dv9
.................................Cacucuacgcuuuugggaacga..	2	1	dv9
.................................uacucuacgcuuuugggaacCa..	6	1	dv9
.................................uacucuacgcuuuuCggaacga..	1	1	dv9
.................................uacGcuacgcuuuugggaacga..	1	1	dv9
.................................uacucuacgcuuuuUggaacga..	1	1	dv9
.................................uacucuacgcuuuugggaGcga..	4	1	dv9
.................................uacucuacgcuuuugCgaacga..	3	1	dv9
.................................uacucuaUgcuuuugggaacga..	1	1	dv9
.................................uacucuacgcuuuugggaacgG..	4	1	dv9
.................................uacucuCcgcuuuugggaacga..	2	1	dv9
.................................uacucuacgcuuuugggaacgU..	6	1	dv9
.................................uacucuacgcuuCugggaacga..	1	1	dv9
.................................uacucuacgcuuuugggaCcga..	19	1	dv9
.................................uacucuacgcuuuugggaacga..	1699	0	dv9
.................................uacucuacgcuuuugggaacgC..	44	1	dv9
.................................uacucuacgcuuuugUgaacga..	21	1	dv9
.................................Gacucuacgcuuuugggaacga..	3	1	dv9
.................................uacucuacgcuuuugggGacga..	3	1	dv9
.................................uacucuacgcuuuugggaacgag...	5	0	dv9
.................................uacucuacgcuuuugggaacgCg...	1	1	dv9
.................................uacucuacgcuuuugggaacgaU...	9	1	dv9
.................................uacucuacgcuuuugggaacgaA...	7	1	dv9
.................................uacucuacgcuuuugggaacgaC...	14	1	dv9
.................................uacucuacgcuuuugggaacgaUc..	7	1	dv9
.................................uacucuacgcuuuugggaacgaCc..	1	1	dv9
.................................uacucuacgcuuuugggaacgagcCc..	1	1	dv9
..................................acucuacgcuuuugggaacga..	6	0	dv9
..................................acucuacgcuuuugggaacgag...	5	0	dv9
..................................acucuacgcuuuugggaCcgag...	1	1	dv9

```
                                                              dvi-miR-9699-5p
                                                                                                     dvi-miR-9699-3p
ugguacaggcacaacugggaacaggcacagccguacucuacgcuuuugggaacgagcucuuugacgcacucgguuccauugccguugaguagagcuguccugaugcauaucucaacg

..................................acucuacgcuuuugggCacgag.............................................................  1    1    dv9
..................................acucuacgcuuuugggaacgagA............................................................  2    1    dv9
..................................acucuacgcuuuugggaacgagU............................................................  1    1    dv9
..................................acucuacgcuuuugggaacgagc............................................................  1    0    dv9
...................................cucuacgcuuuugggaacg...............................................................  7    0    dv9
....................................ucuacgcuuuugggaacg...............................................................  3    0    dv9
....................................ucuacgcuuuugggaacga..............................................................  1    0    dv9
.....................................cuacgcuuuugggaacga..............................................................  5    0    dv9
......................................................agcucuuugacgcacucg.............................................  1    0    dv9
.....................................................................ucgguuccauugccguugagu...........................  1    0    dv9
.....................................................................ucgguuccauugccguuUaguag.........................  1    1    dv9
.....................................................................ucgguuccauugccguugaguag.........................  7    0    dv9
.....................................................................ucgguuccauugccguugaguaga........................  1    0    dv9
.....................................................................ucgguuccauugccguugaguagU........................  1    1    dv9
......................................................................cgguuccauugccguugagu...........................  1    0    dv9
......................................................................cgguuccauugccguugaguag.........................  1    0    dv9
......................................................................cgguuccauugccguugaguaga........................  1    0    dv9
......................................................................Agguuccauugccguugaguaga........................  1    1    dv9
.......................................................................gguuccauugccguuUag............................  1    1    dv9
.......................................................................gguuccauugccguugag............................  2    0    dv9
.......................................................................gguuccauugccguugagu...........................  11   0    dv9
.......................................................................gguuccauugccguugCgu...........................  1    1    dv9
.......................................................................Uguuccauugccguugagu...........................  1    1    dv9
.......................................................................gguuccauugccguugagua..........................  16   0    dv9
.......................................................................gguuccauugccguugaguC..........................  5    1    dv9
.......................................................................gguuccauugUcguugaguag.........................  2    1    dv9
.......................................................................Uguuccauugccguugaguag.........................  53   1    dv9
.......................................................................gAuuccauugccguugaguag.........................  1    1    dv9
.......................................................................gguuccauugccCuugaguag.........................  1    1    dv9
.......................................................................Cguuccauugccguugaguag.........................  1    1    dv9
.......................................................................gguuccauugccguuUaguag.........................  2    1    dv9
.......................................................................gguuccauugccguugaguaC.........................  2    1    dv9
.......................................................................gguuccauugccguCgaguag.........................  2    1    dv9
.......................................................................gguuccauugccguugGguag.........................  2    1    dv9
.......................................................................gguuccauugccguugagGag.........................  2    1    dv9
.......................................................................gguuccauugccguugaguaU.........................  8    1    dv9
.......................................................................gguuccauugccguugaguCg.........................  33   1    dv9
.......................................................................gguuUcauugccguugaguag.........................  1    1    dv9
.......................................................................gguuccauugccguugUguag.........................  4    1    dv9
.......................................................................gguucAauugccguugaguag.........................  1    1    dv9
.......................................................................ggCuccauugccguugaguag.........................  2    1    dv9
.......................................................................Aguuccauugccguugaguag.........................  3    1    dv9
.......................................................................gguuccauugccguugaguag.........................  879  0    dv9
.......................................................................gguuccauCgccguugaguag.........................  1    1    dv9
.......................................................................gguuccauugccguugCguag.........................  14   1    dv9
.......................................................................gguuccauugccguugaCuag.........................  1    1    dv9
.......................................................................gguuccauugccguugaguaA.........................  5    1    dv9
.......................................................................gguuccauugAcguugaguag.........................  1    1    dv9
.......................................................................gguuccauugccguugaUuag.........................  8    1    dv9
.......................................................................gguuccauuUccguugaguag.........................  4    1    dv9
.......................................................................ggGuccauugccguugaguag.........................  1    1    dv9
.......................................................................gUuuccauugccguugaguag.........................  3    1    dv9
.......................................................................gguucUauugccguugaguag.........................  1    1    dv9
.......................................................................gguuccauugccUuugaguag.........................  3    1    dv9
.......................................................................gguuccauugccgCugaguag.........................  2    1    dv9
.......................................................................gguuccCuugccguugaguag.........................  1    1    dv9
.......................................................................gguuccauugccguuCaguag.........................  2    1    dv9
.......................................................................gguuccauugccguugaguagG........................  3    1    dv9
.......................................................................gguuccauugccguugaguCga........................  33   1    dv9
.......................................................................gguuccauugccguugaguaAa........................  3    1    dv9
.......................................................................gguCccauugccguugaguaga........................  1    1    dv9
.......................................................................gguuccauugccguCgaguaga........................  4    1    dv9
.......................................................................gguuUcauugccguugaguaga........................  3    1    dv9
.......................................................................gguuccauugccUuugaguaga........................  1    1    dv9
.......................................................................gguuccauugccgCugaguaga........................  1    1    dv9
.......................................................................gguuccaCugccguugaguaga........................  3    1    dv9
.......................................................................gguucUauugccguugaguaga........................  5    1    dv9
.......................................................................Aguuccauugccguugaguaga........................  1    1    dv9
.......................................................................gguuccauugccguuUaguaga........................  6    1    dv9
```

```
                                dvi-miR-9699-5p
                                                                       dvi-miR-9699-3p
ugguacaggcacaacugggaacaggcacagccguacucuacgcuuuugggaacgagcucuuugacgcacucgguuccauugccguugaguagagcuguccugaugcauaucucaacg

.......................................................................gguuccauugAcguugaguaga........................   2      1      dv9
.......................................................................gguuccauugccguugaguaCa........................   2      1      dv9
.......................................................................ggCuccauugccguugaguaga........................   4      1      dv9
.......................................................................gUuuccauugccguugaguaga........................   4      1      dv9
.......................................................................gguuccGuugccguugaguaga........................   1      1      dv9
.......................................................................gguuccauugUcguugaguaga........................   1      1      dv9
.......................................................................gguuccauugccguugaguagC........................   30     1      dv9
.......................................................................gguuccauugccguugaguagU........................   2      1      dv9
.......................................................................Uguuccauugccguugaguaga........................   83     1      dv9
.......................................................................gguuccauugccguugaguGga........................   5      1      dv9
.......................................................................gguuccauugccguugCguaga........................   43     1      dv9
.......................................................................gguuccauCgccguugaguaga........................   1      1      dv9
.......................................................................gguuccauugccguugGguaga........................   4      1      dv9
.......................................................................gguuccauugccguugaguUga........................   1      1      dv9
.......................................................................gguuccauugccguugaUuaga........................   3      1      dv9
.......................................................................gguuccauugccguugaguaga........................   1399   0      dv9
.......................................................................gguuccauugccgGugaguaga........................   2      1      dv9
.......................................................................gCuuccauugccguugaguaga........................   3      1      dv9
.......................................................................gguuccauugccguugagCaga........................   1      1      dv9
.......................................................................gguuAcauugccguugaguaga........................   1      1      dv9
.......................................................................gguuccauugccguugaguaUa........................   15     1      dv9
.......................................................................gguuccCuugccguugaguaga........................   2      1      dv9
.......................................................................gguuccauugccguugCguagag.......................   3      1      dv9
.......................................................................gguuccauugccguugaguagag.......................   64     0      dv9
.......................................................................gguuccauugccguuUaguagag.......................   1      1      dv9
.......................................................................ggCuccauugccguugaguagag.......................   1      1      dv9
.......................................................................Uguuccauugccguugaguagag.......................   7      1      dv9
.......................................................................gguuccauugccguugaguCgag.......................   2      1      dv9
.......................................................................gguuccauugccguugaguagaU.......................   5      1      dv9
.......................................................................gguuccauugccguugaguagaA.......................   10     1      dv9
.......................................................................gguuccauugccguugaguagCg.......................   1      1      dv9
.......................................................................gguuccauugccguugaguaUag.......................   1      1      dv9
.......................................................................gguuccauugccguugaguagaC.......................   5      1      dv9
.......................................................................gguuccauugccguugaguagagA......................   1      1      dv9
.......................................................................gguuccauugccguugaguagaCc......................   6      1      dv9
........................................................................guuccauugccguugagua..........................   2      0      dv9
........................................................................guuccauugccguugaguag.........................   31     0      dv9
........................................................................Uuuccauugccguugaguag.........................   2      1      dv9
........................................................................guuccauugccguugaUuaga........................   3      1      dv9
........................................................................guuccauugccguugaguCga........................   8      1      dv9
........................................................................guuccauugccguugaguagC........................   10     1      dv9
........................................................................guuccaCugccguugaguaga........................   1      1      dv9
........................................................................guuccauugccguugCguaga........................   2      1      dv9
........................................................................guuccauugccguuUaguaga........................   1      1      dv9
........................................................................Uuuccauugccguugaguaga........................   21     1      dv9
........................................................................guuccauuCccguugaguaga........................   1      1      dv9
........................................................................guuccauugccguugaguaga........................   257    0      dv9
........................................................................guuccauCgccguugaguaga........................   2      1      dv9
........................................................................guuccauugccguugaguaUa........................   2      1      dv9
........................................................................Cuuccauugccguugaguaga........................   2      1      dv9
........................................................................guuccauugccguugaguagG........................   1      1      dv9
........................................................................guuccauuCccguugaguagag.......................   1      1      dv9
........................................................................guuccauugcAguugaguagag.......................   1      1      dv9
........................................................................guuccauugccguugaguagGg.......................   1      1      dv9
........................................................................Uuuccauugccguugaguagag.......................   22     1      dv9
........................................................................guuccauugccguugaguagag.......................   295    0      dv9
........................................................................guuccauugccguugaguaUag.......................   7      1      dv9
........................................................................guuccauugccguugaguagaC.......................   3      1      dv9
........................................................................guuccauugccUuugaguagag.......................   1      1      dv9
........................................................................guuccauugccguugaguagaU.......................   4      1      dv9
........................................................................guucAauugccguugaguagag.......................   1      1      dv9
........................................................................guuccaCugccguugaguagag.......................   1      1      dv9
........................................................................guuccauugccguugaguaCag.......................   1      1      dv9
........................................................................guuccauAgccguugaguagag.......................   1      1      dv9
........................................................................guuccGuugccguugaguagag.......................   1      1      dv9
........................................................................guuccauugccguugaguagaA.......................   1      1      dv9
........................................................................guuccauugccguugaguagCg.......................   11     1      dv9
........................................................................guuccauugccguugaguCgag.......................   9      1      dv9
........................................................................guuccauugccguugCguagag.......................   8      1      dv9
```

```
                                dvi-miR-9699-5p
                                                                       dvi-miR-9699-3p
ugguacaggcacaacugggaacaggcacagccguacucuacgcuuuugggaacgagcucuuugacgcacucgguuccauugccguugaguagagcuguccugaugcauaucucaacg

........................................................................guuccauugccguugaguagagU......................    9    1    dv9
........................................................................Uuuccauugccguugaguagagc......................    1    1    dv9
........................................................................guuccauugccguugaguagagc......................    2    0    dv9
........................................................................guuccauugccguugaguagagA......................    6    1    dv9
.........................................................................uuccauugccguugaguag.........................    2    0    dv9
.........................................................................uuccauugccguugaguagU........................    1    1    dv9
.........................................................................uuccauugccguugaguaga........................    5    0    dv9
.........................................................................uuccauugccguugaguagaC.......................    1    1    dv9
.........................................................................uuccauugccguugaguagag.......................    11   0    dv9
.........................................................................uuccauugccguugaguagagc......................    3    0    dv9
.........................................................................uuccauugccguugaguagagU......................    1    1    dv9
.........................................................................uuccauugccguugaguagagcuU....................    1    1    dv9
..........................................................................uccauugccguugaguaga........................    4    0    dv9
..........................................................................uccauugccguugaguagag.......................    3    0    dv9
...........................................................................ccauugccguugaguaga........................    2    0    dv9
............................................................................cauugccguugaguagag.......................    3    0    dv9
```

miRBase precursor : dvi-mir-9699
Total read count : 20964
dvi-miR-9699-5p read count : 12370
dvi-miR-9699-3p read count : 8592
remaining reads : 2

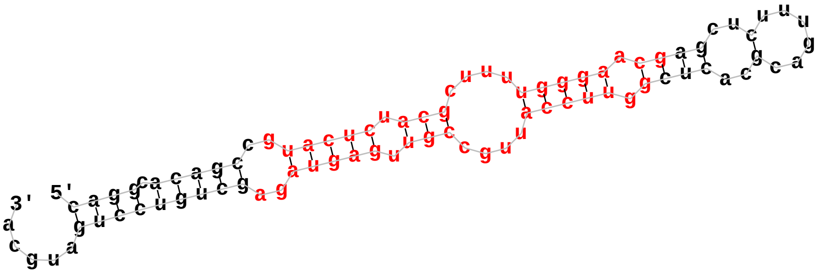

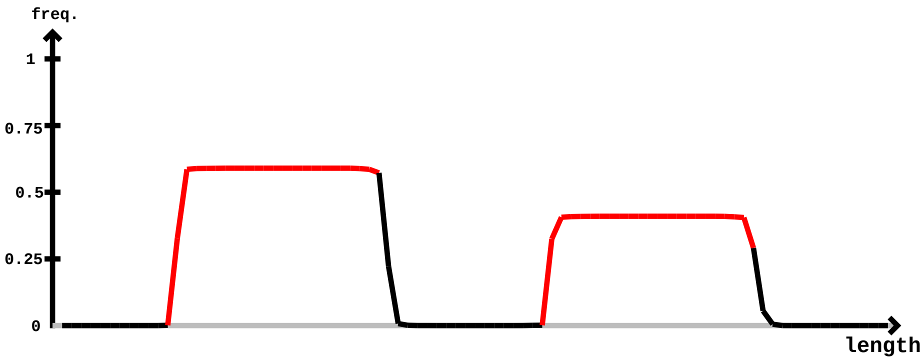

dvi-miR-9699-5p

dvi-miR-9699-3p

```
5'- caggcacagccguacucuacgcuuuugggaacgagcucuuugacgcacucgguuccauugccguugaguagagcuguccugaugca -3'   exp
    ((((.(((((..(((((.(((....(((((.((((..(......)..)))).)))))....))).)))))..))))))))).....   reads   mm   sample
    ..........cguacucuacgcuuuugggaa.......................................................   1       0    dv1
    ..........cguacucuacgcuuuugggaac......................................................   2       0    dv1
    ..........cguacucuacgcuuuugggaacg.....................................................   2       0    dv1
    ..........cguacucuacgcuuuugggaacU.....................................................   1       1    dv1
    ...........guacucuacgcuuuuggga........................................................   4       0    dv1
    ...........guacucuacgcuuuugggCa.......................................................   1       1    dv1
    ...........Uuacucuacgcuuuugggaa.......................................................   1       1    dv1
    ...........guacucuacgcCuuugggaa.......................................................   1       1    dv1
    ...........guacucuacgcuuuugggaa.......................................................   12      0    dv1
    ...........guacucuacgcuCuugggaac......................................................   1       1    dv1
    ...........guacucuacgcuuuugggaac......................................................   38      0    dv1
    ...........Uuacucuacgcuuuugggaac......................................................   1       1    dv1
    ...........guacucuaAgcuuuugggaac......................................................   1       1    dv1
    ...........guacucuacgcuuuuCggaac......................................................   1       1    dv1
    ...........guacucuacgcuuuugggGacg.....................................................   4       1    dv1
    ...........guacucuacCcuuuugggaacg.....................................................   1       1    dv1
    ...........guacucuacgcuuuugggCacg.....................................................   43      1    dv1
    ...........gCacucuacgcuuuugggaacg.....................................................   2       1    dv1
    ...........guacucuacgcuuuugUgaacg.....................................................   36      1    dv1
    ...........guacucuacgcuAuugggaacg.....................................................   1       1    dv1
    ...........guacuUuacgcuuuugggaacg.....................................................   1       1    dv1
    ...........guacucuacgcuuuugggaaAg.....................................................   2       1    dv1
    ...........guacucuacgcuuuugCgaacg.....................................................   1       1    dv1
    ...........gGacucuacgcuuuugggaacg.....................................................   1       1    dv1
    ...........guacucuacgcuuuuggAaacg.....................................................   1       1    dv1
    ...........Uuacucuacgcuuuugggaacg.....................................................   78      1    dv1
    ...........guacucuacgcuuuuUggaacg.....................................................   8       1    dv1
    ...........guacucuaUgcuuuugggaacg.....................................................   6       1    dv1
    ...........guacucuacgcuuuugggaCcg.....................................................   60      1    dv1
    ...........guacucuacgcuuuugggaaUg.....................................................   4       1    dv1
    ...........Cuacucuacgcuuuugggaacg.....................................................   3       1    dv1
    ...........guacucuacgcuuuugggaUcg.....................................................   4       1    dv1
    ...........guacucuacgcuuuCgggaacg.....................................................   1       1    dv1
    ...........guacucuacgcuuuuggUaacg.....................................................   44      1    dv1
```

```
           dvi-miR-9699-5p
                                            dvi-miR-9699-3p
caggcacagccguacucuacgcuuuugggaacgagcucuuugacgcacucgguuccauugccguugaguagagcuguccugaugca

...........guGcucuacgcuuuugggaacg.....................................................    3      1     dv1
...........guacucuacgcuuuuggCaacg.....................................................    1      1     dv1
...........guacucuGcgcuuuugggaacg.....................................................    4      1     dv1
...........guacucuacgcuCuugggaacg.....................................................    3      1     dv1
...........guacucuacgcuuuugggUacg.....................................................    1      1     dv1
...........guacucuacUcuuuugggaacg.....................................................    2      1     dv1
...........guacucuacgcuuuugggaacg.....................................................    1535   0     dv1
...........guCcucuacgcuuuugggaacg.....................................................    1      1     dv1
...........guaAucuacgcuuuugggaacg.....................................................    1      1     dv1
...........guacucuacgcuuuugggaacU.....................................................    11     1     dv1
...........guacucuacgcuuuugggaGcg.....................................................    3      1     dv1
...........guacucuacgcCuuugggaacg.....................................................    4      1     dv1
...........gAacucuacgcuuuugggaacg.....................................................    1      1     dv1
...........guacucuacgUuuuugggaacg.....................................................    4      1     dv1
...........guacucuacgcGuuugggaacg.....................................................    1      1     dv1
...........guacucCacgcuuuugggaacg.....................................................    2      1     dv1
...........guaUucuacgcuuuugggaacg.....................................................    1      1     dv1
...........guacucuacgcuuuugggaacga....................................................    82     0     dv1
...........guacucuacgcuuuugggCacga....................................................    2      1     dv1
...........guacucuacgcuuuugggaCcga....................................................    1      1     dv1
...........guacucuacgcuuuugggaacgC....................................................    2      1     dv1
...........guacucuacgcuuuugggGacga....................................................    1      1     dv1
...........Uuacucuacgcuuuugggaacga....................................................    5      1     dv1
...........guacucuacgcuuuugggaacgU....................................................    15     1     dv1
...........guacAcuacgcuuuugggaacga....................................................    1      1     dv1
...........Cuacucuacgcuuuugggaacga....................................................    2      1     dv1
...........guacucuacgcuuuuggUaacga....................................................    2      1     dv1
...........guacucuacgcuuuugggaaUga....................................................    1      1     dv1
...........guacucuacgcuuuugggaacgaU...................................................    2      1     dv1
...........guacucuacgcuuuugggaacUag...................................................    1      1     dv1
............uacucuacgcuuuuggga........................................................    1      0     dv1
............uacucuacgcuuuugUgaa.......................................................    1      1     dv1
............uacucuacgcuuuugggaa.......................................................    9      0     dv1
............uacucuacgcuuuugggaac......................................................    49     0     dv1
............uacucuacgcuuuuggUaac......................................................    1      1     dv1
............uacucuacgcuuuugggUacg.....................................................    1      1     dv1
............Gacucuacgcuuuugggaacg.....................................................    1      1     dv1
............uacucuacgcuuuugggaacU.....................................................    2      1     dv1
............uacucuacgcuuuugggGacg.....................................................    1      1     dv1
............uacucuacgcuuuugggaacg.....................................................    266    0     dv1
............uacucuacgcuuuugUgaacg.....................................................    2      1     dv1
............uacucuGcgcuuuugggaacg.....................................................    1      1     dv1
............uacucuacgcuuuuUggaacg.....................................................    1      1     dv1
............uacucuacgcuuuugggCacg.....................................................    4      1     dv1
............uacucuacgcuuuugggaCcg.....................................................    4      1     dv1
............uacucuacgcuuuugggaGcg.....................................................    2      1     dv1
............uacucuacgcuuuuggUaacg.....................................................    5      1     dv1
............uacucuacgcuuuuCggaacg.....................................................    1      1     dv1
............uacCcuacgcuuuugggaacga....................................................    2      1     dv1
............uacucuacgcuuuugggaacgU....................................................    4      1     dv1
............uacucuacgcuuuugggaUcga....................................................    1      1     dv1
............uacucuacCcuuuugggaacga....................................................    2      1     dv1
............Gacucuacgcuuuugggaacga....................................................    2      1     dv1
............uacucuacgcuuuuggUaacga....................................................    23     1     dv1
............uacucuacgcuuuuUggaacga....................................................    8      1     dv1
............uacucuacgcCuuugggaacga....................................................    3      1     dv1
............uacuAuacgcuuuugggaacga....................................................    2      1     dv1
............uacucuacgcuuuugCgaacga....................................................    1      1     dv1
............uacucuacgcuuAugggaacga....................................................    1      1     dv1
............uacucuacgcuuuugUgaacga....................................................    24     1     dv1
............uacucuGcgcuuuugggaacga....................................................    1      1     dv1
............uacucCacgcuuuugggaacga....................................................    3      1     dv1
............uacucuacgcuuuuCggaacga....................................................    2      1     dv1
............uacucuacgcuuuugggUacga....................................................    1      1     dv1
............uaUucuacgcuuuugggaacga....................................................    1      1     dv1
............uacucuacgcuuuugggaacgC....................................................    22     1     dv1
............uacucuacgcuuuugggaaAga....................................................    1      1     dv1
............Cacucuacgcuuuugggaacga....................................................    1      1     dv1
............uacucuacgcuuuugggaaUga....................................................    3      1     dv1
```

```
           dvi-miR-9699-5p
                                                  dvi-miR-9699-3p
caggcacagccguacucuacgcuuuugggaacgagcucuuugacgcacucgguuccauugccguugaguagagcuguccugaugca

............uacucuacgcuCuugggaacga....................................................   1      1    dv1
............uacucuacgcuuuugggaacUa....................................................   2      1    dv1
............uacucuacgcuuuAgggaacga....................................................   1      1    dv1
............uacucuacgcuuuuggCaacga....................................................   3      1    dv1
............uacucuacgcuuuugggaacga....................................................   1407   0    dv1
............uacucuacgUuuuugggaacga....................................................   3      1    dv1
............uacucuacgcuuuugggGacga....................................................   4      1    dv1
............uaAucuacgcuuuugggaacga....................................................   1      1    dv1
............uacucuacgcuuuugggaCcga....................................................   15     1    dv1
............uacucuaUgcuuuugggaacga....................................................   1      1    dv1
............uacucuacgAuuuugggaacga....................................................   1      1    dv1
............uacucuacgcuuuugggaGcga....................................................   2      1    dv1
............uacucuacgcuuuugggCacga....................................................   33     1    dv1
............uacucuacgcuuuugggaacAa....................................................   1      1    dv1
............uacucuacgcuuuugggaacCag...................................................   1      1    dv1
............uacucuacgcuuuugggaacgaC...................................................   11     1    dv1
............uacucuacgcuuuugggaacgaU...................................................   8      1    dv1
............uacucuacgcuuuugggaacgag...................................................   4      0    dv1
............uacucuacgcuuuugggaacgaA...................................................   8      1    dv1
............uacucuacgcuuuugggaacgaAc..................................................   1      1    dv1
............uacucuacgcuuuugggaacgaUc..................................................   2      1    dv1
............uacucuacgcuuuugggaacgaCc..................................................   1      1    dv1
.............acucuacgcuuuugggaacg.....................................................   2      0    dv1
.............acucuacgcuuuugggaacga....................................................   5      0    dv1
.............acucuacgcuuuugggaacgag...................................................   8      0    dv1
.............acucuacgcuuuugggaacgagU..................................................   1      1    dv1
..............cucuacgcuuuugggaacg.....................................................   2      0    dv1
...............ucuacgcuuuugggaacg.....................................................   1      0    dv1
...............ucuacgcuuuugggaacga....................................................   1      0    dv1
...............ucuacgcuuuugggaacgag...................................................   1      0    dv1
................cuacgcuuuugggaacga....................................................   3      0    dv1
................................................ucgguuccauugccguugag..................   1      0    dv1
................................................ucgguuccauugccguugagu.................   1      0    dv1
................................................ucgguuccauugcUguugaguag...............   1      1    dv1
.................................................cgguuccauugccguugaguag...............   2      0    dv1
.................................................cgguuccauugccguugaguaga..............   2      0    dv1
..................................................gguuccauugccguugag..................   6      0    dv1
..................................................gguuccauugccguugagu.................   7      0    dv1
..................................................gguuccauugccguugagua................   14     0    dv1
..................................................gguuccauugccguugaUua................   1      1    dv1
..................................................Uguuccauugccguugagua................   1      1    dv1
..................................................Aguuccauugccguugaguag...............   1      1    dv1
..................................................gguuccauugccAuugaguag...............   1      1    dv1
..................................................gguuccauugUcguugaguag...............   1      1    dv1
..................................................gguuccauugccguugCguag...............   11     1    dv1
..................................................gguuccauugccguCgaguag...............   2      1    dv1
..................................................gguuccauugccguugaguaU...............   5      1    dv1
..................................................gguuccauugccguugaCuag...............   1      1    dv1
..................................................gguuccauugccguuUaguag...............   3      1    dv1
..................................................Cguuccauugccguugaguag...............   1      1    dv1
..................................................gUuuccauugccguugaguag...............   4      1    dv1
..................................................gguuccauugccgAugaguag...............   1      1    dv1
..................................................gguuccaCugccguugaguag...............   1      1    dv1
..................................................gguuccauugccguugaguag...............   637    0    dv1
..................................................gguuccauugccguugaguCg...............   20     1    dv1
..................................................gguuccauugccguugaUuag...............   4      1    dv1
..................................................gguuccauugccguugaguGg...............   1      1    dv1
..................................................gguuccauugccguugaguUg...............   1      1    dv1
..................................................gguuccauugccguugaguaA...............   4      1    dv1
..................................................gguuccauugccguugGguag...............   2      1    dv1
..................................................gguuccauugccUuugaguag...............   3      1    dv1
..................................................gguuccauCgccguugaguag...............   1      1    dv1
..................................................gguCccauugccguugaguag...............   1      1    dv1
..................................................Uguuccauugccguugaguag...............   32     1    dv1
..................................................gguucUauugccguugaguag...............   2      1    dv1
..................................................gguuccauugccguugaguaCa..............   1      1    dv1
..................................................gguucAauugccguugaguaga..............   2      1    dv1
..................................................gguuccauugccguugagGaga..............   1      1    dv1
..................................................gguuccauuUccguugaguaga..............   1      1    dv1
```

dvi-miR-9699-5p

dvi-miR-9699-3p

caggcacagccguacucuacgcuuuugggaacgagcucuuugacgcacucgguuccauugccguugaguagagcuguccugaugca

....gguuccauugccguuUaguaga..............	2	1	dv1
....gguuccauugccguugaguCga..............	21	1	dv1
....gguuccauugccguugaUuaga..............	2	1	dv1
....Aguuccauugccguugaguaga..............	1	1	dv1
....gguuccauuCccguugaguaga..............	3	1	dv1
....gguuccauugcUguugaguaga..............	2	1	dv1
....gguuccauCgccguugaguaga..............	4	1	dv1
....gguuccauugccgCugaguaga..............	1	1	dv1
....Uguuccauugccguugaguaga..............	43	1	dv1
....gguuccCuugccguugaguaga..............	1	1	dv1
....gUuuccauugccguugaguaga..............	1	1	dv1
....gguuccauugccguugaguaga..............	870	0	dv1
....gguuccauugccUuugaguaga..............	3	1	dv1
....gguuUcauugccguugaguaga..............	1	1	dv1
....gguuccauugccguugaguaUa..............	2	1	dv1
....gguuccaCugccguugaguaga..............	1	1	dv1
....gguuccauugccguugagCaga..............	2	1	dv1
....gguuccauugccguugaguagC..............	24	1	dv1
....gguuccauugccguugaguagU..............	1	1	dv1
....gguuccauugccguCgaguaga..............	1	1	dv1
....gguuccauugccguugagAaga..............	2	1	dv1
....gguuccauugccCuugaguaga..............	1	1	dv1
....gguuccauugAcguugaguaga..............	1	1	dv1
....gguuccauugccguugCguaga..............	17	1	dv1
....gguuccauugccguugaguGga..............	1	1	dv1
....gguuccauugccguugGguaga..............	2	1	dv1
....gguuccauugccguuCaguaga..............	1	1	dv1
....gguuccauugccguugaguagag.............	29	0	dv1
....gguuccauugccguugaguagCg.............	2	1	dv1
....Uguuccauugccguugaguagag.............	1	1	dv1
....gguCccauugccguugaguagag.............	1	1	dv1
....gguuccauugccguugaguagaA.............	4	1	dv1
....gguuccauugccguugaguagaC.............	1	1	dv1
....gguuccauugccguugCguagag.............	5	1	dv1
....gguuccauugccguugaguagaU.............	1	1	dv1
....gguuccauugccguugaguagagA............	4	1	dv1
....gguuccauugccguugaguagagU............	2	1	dv1
....gguuccauugccguugaguagaCc............	3	1	dv1
....guuccauugccguugagua................	1	0	dv1
....guuccauugccguugaguag...............	27	0	dv1
....guuccauugccgCugaguag...............	1	1	dv1
....guuccauugccguugCguag...............	1	1	dv1
....guuccauugccguugaUuag...............	1	1	dv1
....guuccauugccguugCguaga..............	4	1	dv1
....Uuuccauugccguugaguaga..............	11	1	dv1
....guuccauugccguugaguagC..............	7	1	dv1
....guuccauCgccguugaguaga..............	1	1	dv1
....guuccauugccgCugaguaga..............	1	1	dv1
....guuccauugccguugaguaga..............	187	0	dv1
....guuccauugccguugagCaga..............	1	1	dv1
....guuccauuUccguugaguaga..............	1	1	dv1
....guuccauugccguugGguaga..............	1	1	dv1
....guuccauugccgGugaguaga..............	1	1	dv1
....guuccauugccguugaUuaga..............	1	1	dv1
....guuccauugccguugaguCga..............	4	1	dv1
....guuccauugccguuUaguaga..............	1	1	dv1
....guuccauugccguuAaguaga..............	1	1	dv1
....guuccauugccguugaguaCag.............	1	1	dv1
....guuccauugccguugaguGgag.............	1	1	dv1
....guuccauugccguugaUuagag.............	1	1	dv1
....guuccauugccguugaguCgag.............	9	1	dv1
....guuccaCugccguugaguagag.............	1	1	dv1
....guuccauugccguugCguagag.............	7	1	dv1
....Uuuccauugccguugaguagag.............	9	1	dv1
....guuccauugccguugagCagag.............	1	1	dv1
....guuccauugccguugagGagag.............	1	1	dv1
....guuccauugccgGugaguagag.............	1	1	dv1
....guuccauugccUuugaguagag.............	1	1	dv1
....guCccauugccguugaguagag.............	1	1	dv1

```
           dvi-miR-9699-5p
                                                  dvi-miR-9699-3p
caggcacagccguacucuacgcuuuugggaacgagcucuuugacgcacucgguuccauugccguugaguagagcuguccugaugca

...................................................guuccauuUccguugaguagag.............    2      1    dv1
...................................................guuccauugccguugaguagag.............    182    0    dv1
...................................................guuccauugccguugaguagCg.............    5      1    dv1
...................................................guuccauugccguugaCuagag.............    1      1    dv1
...................................................guuccauugccguugGguagag.............    2      1    dv1
...................................................gCuccauugccguugaguagag.............    2      1    dv1
...................................................guuccauugccguugaguaUag.............    3      1    dv1
...................................................guuccauugccguugaguagagA............    19     1    dv1
...................................................guuccauugccguugaguagaCc............    1      1    dv1
...................................................guuccauugccguugaguagagU............    18     1    dv1
...................................................guuccauugccguugaguagagUu...........    1      1    dv1
....................................................uuccauugccguugaguag...............    1      0    dv1
....................................................uuccauugccguugaguaga..............    5      0    dv1
....................................................uuccauugccguugaguagag.............    1      0    dv1
....................................................uuccauugcUguugaguagag.............    1      1    dv1
....................................................uuccauugccguugaguagagc............    2      0    dv1
....................................................uuccauugccguugaguagagU............    1      1    dv1
.....................................................uccauugccguugaguag...............    2      0    dv1
.....................................................uccauugccguugaguaga..............    3      0    dv1
.....................................................uccauugccguugGguaga..............    1      1    dv1
......................................................ccauugccguugaguaga..............    2      0    dv1
......................................................ccauugccguugaguagag.............    1      0    dv1

..........cguacucuacgcuuuugggaa.......................................................    3      0    dv9
..........cguacucuacgcuuuugggaacg.....................................................    1      0    dv9
..........cguacucuacgcuuuugggaacA.....................................................    2      1    dv9
...........guacucuacgcuuuuggga........................................................    12     0    dv9
...........guacucuacgcuuuugUgaa.......................................................    1      1    dv9
...........guacucuacgcuuuugggCa.......................................................    1      1    dv9
...........guacucuacgcuuuuggUaa.......................................................    1      1    dv9
...........guacucuacgcuuuugggaa.......................................................    13     0    dv9
...........guacucuacgcuuuugggaac......................................................    44     0    dv9
...........guacucuacgcuuuugggaaU......................................................    2      1    dv9
...........Uuacucuacgcuuuugggaac......................................................    4      1    dv9
...........guacucuacgcuuuugUgaac......................................................    2      1    dv9
...........guacucuacgcuuuugggaUc......................................................    3      1    dv9
...........guacucuacgcuuuugggCac......................................................    2      1    dv9
...........guacucuacgcuuuugggCacg.....................................................    75     1    dv9
...........guacucuacgcuuuugggaaGg.....................................................    2      1    dv9
...........guacucuacgcuuuuCggaacg.....................................................    1      1    dv9
...........guGcucuacgcuuuugggaacg.....................................................    1      1    dv9
...........guacCcuacgcuuuugggaacg.....................................................    3      1    dv9
...........guacucuacgcuuuGgggaacg.....................................................    2      1    dv9
...........guacucuacgcuuuugCgaacg.....................................................    1      1    dv9
...........guaUucuacgcuuuugggaacg.....................................................    1      1    dv9
...........guacucuacgcuGuugggaacg.....................................................    1      1    dv9
...........guacucuacgcuCuugggaacg.....................................................    5      1    dv9
...........guacucuacgcuuuugggaaUg.....................................................    3      1    dv9
...........guUcucuacgcuuuugggaacg.....................................................    1      1    dv9
...........guacuUuacgcuuuugggaacg.....................................................    1      1    dv9
...........Auacucuacgcuuuugggaacg.....................................................    1      1    dv9
...........guacucuacgcuuCugggaacg.....................................................    1      1    dv9
...........guacucuCcgcuuuugggaacg.....................................................    2      1    dv9
...........guacucuacgcuuuugggGacg.....................................................    6      1    dv9
...........guacucuacgcuuuugggaGcg.....................................................    6      1    dv9
...........guaAucuacgcuuuugggaacg.....................................................    2      1    dv9
...........guacucuacAcuuuugggaacg.....................................................    1      1    dv9
...........guacucuacgcuuuugggaUcg.....................................................    1      1    dv9
...........guacucuacUcuuuugggaacg.....................................................    2      1    dv9
...........guacucuacgcuuuugggaacg.....................................................    2204   0    dv9
...........guacucuacgcuuuugggaacA.....................................................    1      1    dv9
...........guacucuGcgcuuuugggaacg.....................................................    3      1    dv9
...........guacucCacgcuuuugggaacg.....................................................    1      1    dv9
...........guacGcuacgcuuuugggaacg.....................................................    1      1    dv9
...........guacucuacgcuuuugggaacU.....................................................    19     1    dv9
...........guacuAuacgcuuuugggaacg.....................................................    1      1    dv9
...........guacucuacgUuuuugggaacg.....................................................    1      1    dv9
...........gCacucuacgcuuuugggaacg.....................................................    1      1    dv9
...........guacucuacgcuuuuggAaacg.....................................................    1      1    dv9
```

```
           dvi-miR-9699-5p
                                                  dvi-miR-9699-3p
caggcacagccguacucuacgcuuuugggaacgagcucuuugacgcacucgguuccauugccguugaguagagcuguccugaugca

...........guacucuacgcuuuCgggaacg.....................................................    1       1   dv9
...........guacucuacgcuuuugUgaacg.....................................................    15      1   dv9
...........guacucuacgcCuuugggaacg.....................................................    3       1   dv9
...........guacucuacgcuuuuUggaacg.....................................................    4       1   dv9
...........guacucuacgcuuuugggaCcg.....................................................    77      1   dv9
...........Uuacucuacgcuuuugggaacg.....................................................    120     1   dv9
...........guacucuacgcuuuugggaaAg.....................................................    2       1   dv9
...........guacucuacgcuuuuggCaacg.....................................................    4       1   dv9
...........guacucuaUgcuuuugggaacg.....................................................    4       1   dv9
...........guacucuacgcuuuugggaacC.....................................................    12      1   dv9
...........guacucuacgcuuuuggUaacg.....................................................    29      1   dv9
...........guacucuacgcuuuugggaacgC....................................................    1       1   dv9
...........guacucuacgcuuuuggUaacga....................................................    1       1   dv9
...........guacucuacgcuuuugggaCcga....................................................    1       1   dv9
...........guacCcuacgcuuuugggaacga....................................................    1       1   dv9
...........guacucuacgcuuuugggaaGga....................................................    1       1   dv9
...........guacucuacgcuuCugggaacga....................................................    1       1   dv9
...........Uuacucuacgcuuuugggaacga....................................................    16      1   dv9
...........guacucuacgcuuuCgggaacga....................................................    1       1   dv9
...........guacucuacgcuuuugggaacCa....................................................    1       1   dv9
...........guacucuacgcAuuugggaacga....................................................    1       1   dv9
...........guacucuacgcuuuugggaacga....................................................    127     0   dv9
...........gGacucuacgcuuuugggaacga....................................................    1       1   dv9
...........guacucuacgcuuuuggCaacga....................................................    1       1   dv9
...........guacucuacgcuuuugggCacga....................................................    5       1   dv9
...........guacucuacgcuuuugggaacgU....................................................    16      1   dv9
...........guacucuacgcuuuugggaacgaU...................................................    1       1   dv9
...........guacucuacgcuuuugggaacgaA...................................................    1       1   dv9
...........guacucuacgcuuuugggaacgGg...................................................    1       1   dv9
............uacucuacgcuuuuggga........................................................    2       0   dv9
............uacucuacgcuuuugggaa.......................................................    8       0   dv9
............uacucuacgcuuuugggCac......................................................    3       1   dv9
............uacucuacgcuuuugggaac......................................................    56      0   dv9
............uacucuacgcuCuugggaac......................................................    1       1   dv9
............Gacucuacgcuuuugggaac......................................................    1       1   dv9
............uacucuacgcuuuuggUaac......................................................    1       1   dv9
............uacucuacgcuuuugggaCc......................................................    1       1   dv9
............uacucuacgcuuuugUgaac......................................................    1       1   dv9
............uacucuacgcuuCugggaac......................................................    1       1   dv9
............uacucuacgcuuuugggaacg.....................................................    235     0   dv9
............uacucuacgcuuuugggaCcg.....................................................    6       1   dv9
............uacucuacgcuuuuggUaacg.....................................................    4       1   dv9
............uacucuacgcuuuugggaacU.....................................................    2       1   dv9
............uacucuacgcuuuuCggaacg.....................................................    1       1   dv9
............uacucuacgcuuuugggaacC.....................................................    1       1   dv9
............uacucuacgcuuuugUgaacg.....................................................    2       1   dv9
............uacucuacgcuuuugggaUcg.....................................................    1       1   dv9
............uacucuacgcuuuugggaacA.....................................................    1       1   dv9
............uacucuacgcuuuugggCacg.....................................................    5       1   dv9
............uacucuacgcuuuCgggaacg.....................................................    1       1   dv9
............Gacucuacgcuuuugggaacg.....................................................    1       1   dv9
............Aacucuacgcuuuugggaacg.....................................................    1       1   dv9
............uGcucuacgcuuuugggaacg.....................................................    1       1   dv9
............uacucuacgcuuuugggaaUg.....................................................    2       1   dv9
............uacucuacgcuuuugggaaAga....................................................    1       1   dv9
............uacCcuacgcuuuugggaacga....................................................    1       1   dv9
............Aacucuacgcuuuugggaacga....................................................    1       1   dv9
............uacucuacgUuuuugggaacga....................................................    2       1   dv9
............uacucuacgcuuuugggaacgG....................................................    4       1   dv9
............uacucuacgcuuuugggCacga....................................................    35      1   dv9
............uacucuacgcuuuugggaacgC....................................................    44      1   dv9
............uacucuacgcCuuugggaacga....................................................    5       1   dv9
............uacucuacgcuGuugggaacga....................................................    1       1   dv9
............uacucuacgcuuuugggaacga....................................................    1699    0   dv9
............uacucuacgcuuuugggaacgU....................................................    6       1   dv9
............uacucuacgcuuuugCgaacga....................................................    3       1   dv9
............uacucuaUgcuuuugggaacga....................................................    1       1   dv9
............uacucuacgcuCuugggaacga....................................................    2       1   dv9
............Gacucuacgcuuuugggaacga....................................................    3       1   dv9
```

dvi-miR-9699-5p

dvi-miR-9699-3p

caggcacagccguacucuacgcuuuugggaacgagcucuuugacgcacucgguuccauugccguugaguagagcuguccugaugca

............uacucuacgcuuuuCggaacga..	1	1	dv9
............uacucuacgcuuuuggUaacga..	18	1	dv9
............uacucuacgcuuuugggaacCa..	6	1	dv9
............uacucuacgcuuuugggaacUa..	11	1	dv9
............uacucuacgcuuuuUggaacga..	1	1	dv9
............Cacucuacgcuuuugggaacga..	2	1	dv9
............uacucuacgcuuCugggaacga..	1	1	dv9
............uacucuacgcuuuugggGacga..	3	1	dv9
............uacucuacgcuuuugggaacAa..	7	1	dv9
............uacucuacgcuuuCgggaacga..	1	1	dv9
............uacucuacgcuuuugggaGcga..	4	1	dv9
............uacucuCcgcuuuugggaacga..	2	1	dv9
............uacucuacgcuuuugUgaacga..	21	1	dv9
............uacucuacgcuuuugggaCcga..	19	1	dv9
............uacucuacgcuuuugggaaUga..	3	1	dv9
............uacucuGcgcuuuugggaacga..	1	1	dv9
............uacucuacgcuuuuggAaacga..	1	1	dv9
............uacGcuacgcuuuugggaacga..	1	1	dv9
............uacucuacgcuuuugAgaacga..	1	1	dv9
............uaAucuacgcuuuugggaacga..	2	1	dv9
............uacucuacgcuuuugggaacgCg...	1	1	dv9
............uacucuacgcuuuugggaacgaA...	7	1	dv9
............uacucuacgcuuuugggaacgaC...	14	1	dv9
............uacucuacgcuuuugggaacgag...	5	0	dv9
............uacucuacgcuuuugggaacgaU...	9	1	dv9
............uacucuacgcuuuugggaacgaUc..	7	1	dv9
............uacucuacgcuuuugggaacgaCc..	1	1	dv9
............uacucuacgcuuuugggaacgagcCc..	1	1	dv9
.............acucuacgcuuuugggaacga..	6	0	dv9
.............acucuacgcuuuugggaCcgag...	1	1	dv9
.............acucuacgcuuuugggaacgag...	5	0	dv9
.............acucuacgcuuuugggCacgag...	1	1	dv9
.............acucuacgcuuuugggaacgagA..	2	1	dv9
.............acucuacgcuuuugggaacgagU..	1	1	dv9
.............acucuacgcuuuugggaacgagc..	1	0	dv9
..............cucuacgcuuuugggaacg...	7	0	dv9
...............ucuacgcuuuugggaacg...	3	0	dv9
...............ucuacgcuuuugggaacga..	1	0	dv9
................cuacgcuuuugggaacga..	5	0	dv9
.................................agcucuuugacgcacucg...................................	1	0	dv9
....ucgguuccauugccguugagu.................	1	0	dv9
....ucgguuccauugccguugaguag...............	7	0	dv9
....ucgguuccauugccguuUaguag...............	1	1	dv9
....ucgguuccauugccguugaguaga..............	1	0	dv9
....ucgguuccauugccguugaguagU..............	1	1	dv9
....cgguuccauugccguugagu.................	1	0	dv9
....cgguuccauugccguugaguag...............	1	0	dv9
....Agguuccauugccguugaguaga..............	1	1	dv9
....cgguuccauugccguugaguaga..............	1	0	dv9
....gguuccauugccguuUag..................	1	1	dv9
....gguuccauugccguugag..................	2	0	dv9
....gguuccauugccguugagu.................	11	0	dv9
....Uguuccauugccguugagu.................	1	1	dv9
....gguuccauugccguugCgu.................	1	1	dv9
....gguuccauugccguugagua................	16	0	dv9
....gguuccauugccguugaguC................	5	1	dv9
....gguuccauugccguCgaguag...............	2	1	dv9
....gguuccauugccguugaguaC...............	2	1	dv9
....ggGuccauugccguugaguag...............	1	1	dv9
....gguuccauugccguugaguCg...............	33	1	dv9
....gguuccauugccCuugaguag...............	1	1	dv9
....gguuccauugccguugaCuag...............	1	1	dv9
....gguucUauugccguugaguag...............	1	1	dv9
....Cguuccauugccguugaguag...............	1	1	dv9
....gguuccauugccUuugaguag...............	3	1	dv9
....gguuccauugAcguugaguag...............	1	1	dv9
....Aguuccauugccguugaguag...............	3	1	dv9
....ggCuccauugccguugaguag...............	2	1	dv9
....gguuccauugccguugCguag...............	14	1	dv9

```
           dvi-miR-9699-5p
                                                  dvi-miR-9699-3p
caggcacagccguacucuacgcuuuugggaacgagcucuuugacgcacucgguuccauugccguugaguagagcuguccugaugca

..................................................gguuccauugccguugUguag...............   4      1   dv9
..................................................gUuuccauugccguugaguag...............   3      1   dv9
..................................................gguuccauuUccguugaguag...............   4      1   dv9
..................................................gAuuccauugccguugaguag...............   1      1   dv9
..................................................gguuccauugccguuCaguag...............   2      1   dv9
..................................................gguuccCuugccguugaguag...............   1      1   dv9
..................................................Uguuccauugccguugaguag...............   53     1   dv9
..................................................gguuccauCgccguugaguag...............   1      1   dv9
..................................................gguuccauugccguugaguag...............   879    0   dv9
..................................................gguucAauugccguugaguag...............   1      1   dv9
..................................................gguuUcauugccguugaguag...............   1      1   dv9
..................................................gguuccauugccguugaUuag...............   8      1   dv9
..................................................gguuccauugccguugaguaA...............   5      1   dv9
..................................................gguuccauugccguuUaguag...............   2      1   dv9
..................................................gguuccauugccgCugaguag...............   2      1   dv9
..................................................gguuccauugccguugagGag...............   2      1   dv9
..................................................gguuccauugccguugaguaU...............   8      1   dv9
..................................................gguuccauugccguugGguag...............   2      1   dv9
..................................................gguuccauugUcguugaguag...............   2      1   dv9
..................................................gguuccauugccguugaguagG..............   3      1   dv9
..................................................gguuccauCgccguugaguaga..............   1      1   dv9
..................................................gguuccauugccguugaguaUa..............   15     1   dv9
..................................................gguuccauugccguugaguaAa..............   3      1   dv9
..................................................gguuccGuugccguugaguaga..............   1      1   dv9
..................................................Aguuccauugccguugaguaga..............   1      1   dv9
..................................................Uguuccauugccguugaguaga..............   83     1   dv9
..................................................gguuccauugccUuugaguaga..............   1      1   dv9
..................................................gguuccauugccguugagCaga..............   1      1   dv9
..................................................gguuccauugccguugGguaga..............   4      1   dv9
..................................................gguuccCuugccguugaguaga..............   2      1   dv9
..................................................gguuccauugccguugaguCga..............   33     1   dv9
..................................................gguuccauugccguugaguagC..............   30     1   dv9
..................................................gguuccauugccgGugaguaga..............   2      1   dv9
..................................................gguuccauugUcguugaguaga..............   1      1   dv9
..................................................gguuccauugccguugaguGga..............   5      1   dv9
..................................................gguuccaCugccguugaguaga..............   3      1   dv9
..................................................gguCccauugccguugaguaga..............   1      1   dv9
..................................................gguuccauugccguugCguaga..............   43     1   dv9
..................................................gguuAcauugccguugaguaga..............   1      1   dv9
..................................................gguuccauugccguugaUuaga..............   3      1   dv9
..................................................gguuccauugAcguugaguaga..............   2      1   dv9
..................................................gUuuccauugccguugaguaga..............   4      1   dv9
..................................................gguuccauugccguuUaguaga..............   6      1   dv9
..................................................gguuccauugccguCgaguaga..............   4      1   dv9
..................................................gguuccauugccguugaguUga..............   1      1   dv9
..................................................ggCuccauugccguugaguaga..............   4      1   dv9
..................................................gguuccauugccgCugaguaga..............   1      1   dv9
..................................................gCuuccauugccguugaguaga..............   3      1   dv9
..................................................gguuccauugccguugaguaga..............   1399   0   dv9
..................................................gguucUauugccguugaguaga..............   5      1   dv9
..................................................gguuccauugccguugaguaCa..............   2      1   dv9
..................................................gguuccauugccguugaguagU..............   2      1   dv9
..................................................gguuUcauugccguugaguaga..............   3      1   dv9
..................................................gguuccauugccguuUaguagag.............   1      1   dv9
..................................................gguuccauugccguugaguagaU.............   5      1   dv9
..................................................gguuccauugccguugaguagag.............   64     0   dv9
..................................................gguuccauugccguugaguCgag.............   2      1   dv9
..................................................gguuccauugccguugCguagag.............   3      1   dv9
..................................................gguuccauugccguugaguagaA.............   10     1   dv9
..................................................gguuccauugccguugaguaUag.............   1      1   dv9
..................................................Uguuccauugccguugaguagag.............   7      1   dv9
..................................................gguuccauugccguugaguagaC.............   5      1   dv9
..................................................gguuccauugccguugaguagCg.............   1      1   dv9
..................................................ggCuccauugccguugaguagag.............   1      1   dv9
..................................................gguuccauugccguugaguagagA............   1      1   dv9
..................................................gguuccauugccguugaguagaCc............   6      1   dv9
...................................................guuccauugccguugagua................   2      0   dv9
...................................................guuccauugccguugaguag...............   31     0   dv9
...................................................Uuuccauugccguugaguag...............   2      1   dv9
```

```
           dvi-miR-9699-5p
                                                  dvi-miR-9699-3p
caggcacagccguacucuacgcuuuugggaacgagcucuuugacgcacucgguuccauugccguugaguagagcuguccugaugca

...................................................guuccauCgccguugaguaga..............    2      1      dv9
...................................................guuccauugccguugaUuaga..............    3      1      dv9
...................................................guuccauugccguugaguCga..............    8      1      dv9
...................................................guuccauugccguuUaguaga..............    1      1      dv9
...................................................guuccauuCccguugaguaga..............    1      1      dv9
...................................................guuccauugccguugaguaga..............    257    0      dv9
...................................................guuccauugccguugaguaUa..............    2      1      dv9
...................................................Cuuccauugccguugaguaga..............    2      1      dv9
...................................................guuccauugccguugaguagC..............    10     1      dv9
...................................................Uuuccauugccguugaguaga..............    21     1      dv9
...................................................guuccauugccguugaguagG..............    1      1      dv9
...................................................guuccaCugccguugaguaga..............    1      1      dv9
...................................................guuccauugccguugCguaga..............    2      1      dv9
...................................................guuccauugcAguugaguagag.............    1      1      dv9
...................................................guuccauugccguugaguaUag.............    7      1      dv9
...................................................guuccaCugccguugaguagag.............    1      1      dv9
...................................................guuccGuugccguugaguagag.............    1      1      dv9
...................................................guuccauugccguugaguagaC.............    3      1      dv9
...................................................guuccauugccguugaguaCag.............    1      1      dv9
...................................................guuccauugccguugCguagag.............    8      1      dv9
...................................................Uuuccauugccguugaguagag.............    22     1      dv9
...................................................guuccauugccguugaguagCg.............    11     1      dv9
...................................................guucAauugccguugaguagag.............    1      1      dv9
...................................................guuccauugccguugaguagGg.............    1      1      dv9
...................................................guuccauugccguugaguagag.............    295    0      dv9
...................................................guuccauugccguugaguagaU.............    4      1      dv9
...................................................guuccauugccUuugaguagag.............    1      1      dv9
...................................................guuccauuCccguugaguagag.............    1      1      dv9
...................................................guuccauAgccguugaguagag.............    1      1      dv9
...................................................guuccauugccguugaguCgag.............    9      1      dv9
...................................................guuccauugccguugaguagaA.............    1      1      dv9
...................................................guuccauugccguugaguagagU............    9      1      dv9
...................................................guuccauugccguugaguagagc............    2      0      dv9
...................................................Uuuccauugccguugaguagagc............    1      1      dv9
...................................................guuccauugccguugaguagagA............    6      1      dv9
....................................................uuccauugccguugaguag...............    2      0      dv9
....................................................uuccauugccguugaguaga..............    5      0      dv9
....................................................uuccauugccguugaguagU..............    1      1      dv9
....................................................uuccauugccguugaguagaC.............    1      1      dv9
....................................................uuccauugccguugaguagag.............    11     0      dv9
....................................................uuccauugccguugaguagagU............    1      1      dv9
....................................................uuccauugccguugaguagagc............    3      0      dv9
....................................................uuccauugccguugaguagagcuU..........    1      1      dv9
.....................................................uccauugccguugaguaga..............    4      0      dv9
.....................................................uccauugccguugaguagag.............    3      0      dv9
......................................................ccauugccguugaguaga..............    2      0      dv9
.......................................................cauugccguugaguagag.............    3      0      dv9

..........cguacucuacgcuuuuggga........................................................    1      0      arg
..........cguacucuacgcuuuugggaacg.....................................................    8      0      arg
...........guacucuacgcuuuuggga........................................................    4      0      arg
...........guacucuacgcuuuugggU........................................................    1      1      arg
...........guacucuacgcuuuugggaa.......................................................    8      0      arg
...........guacucuacgcuuuugggaac......................................................    22     0      arg
...........guacucuacgcuuuugggaaA......................................................    2      1      arg
...........guacucGacgcuuuugggaac......................................................    1      1      arg
...........guacucuacgcuuuuUggaac......................................................    1      1      arg
...........guacucuacUcuuuugggaacg.....................................................    1      1      arg
...........guacucuacgGuuuugggaacg.....................................................    1      1      arg
...........guacucuacgcuuuugggaUcg.....................................................    1      1      arg
...........guacucuacgcuuuuggAaacg.....................................................    1      1      arg
...........guacucuacgcCuuugggaacg.....................................................    2      1      arg
...........guacucuacgcuuuuggCaacg.....................................................    2      1      arg
...........guacucuacgcuuuuAggaacg.....................................................    3      1      arg
...........guacucuacgcuuuuCggaacg.....................................................    1      1      arg
...........guacucuacAcuuuugggaacg.....................................................    3      1      arg
...........guacucuacCcuuuugggaacg.....................................................    1      1      arg
...........guacucuacgcuuCugggaacg.....................................................    1      1      arg
...........guacuAuacgcuuuugggaacg.....................................................    1      1      arg
```

```
           dvi-miR-9699-5p
                                                  dvi-miR-9699-3p
caggcacagccguacucuacgcuuuugggaacgagcucuuugacgcacucgguuccauugccguugaguagagcuguccugaugca

...........guacucuacgcuuuugggaacU.....................................................    7       1       arg
...........gCacucuacgcuuuugggaacg.....................................................    2       1       arg
...........guacucuacgcuuuugggaaAg.....................................................    2       1       arg
...........guacucuacgUuuuugggaacg.....................................................    3       1       arg
...........Cuacucuacgcuuuugggaacg.....................................................    1       1       arg
...........guacucuaUgcuuuugggaacg.....................................................    2       1       arg
...........guacuUuacgcuuuugggaacg.....................................................    4       1       arg
...........Uuacucuacgcuuuugggaacg.....................................................    4       1       arg
...........guacucuacgcuuuugggUacg.....................................................    4       1       arg
...........guacucAacgcuuuugggaacg.....................................................    1       1       arg
...........guacucuacgcuuuugggCacg.....................................................    2       1       arg
...........guacucuacgcuCuugggaacg.....................................................    1       1       arg
...........guacucuacgcuuGugggaacg.....................................................    1       1       arg
...........guacucuacgcuuuugCgaacg.....................................................    1       1       arg
...........guacucuacgcuuuCgggaacg.....................................................    3       1       arg
...........guacucuacgcuuuuggUaacg.....................................................    4       1       arg
...........guacucCacgcuuuugggaacg.....................................................    1       1       arg
...........guacucuacgcuuuugggaacg.....................................................    1679    0       arg
...........guacucuacgcuuuugUgaacg.....................................................    6       1       arg
...........guacucuacgcuuuugggaacC.....................................................    2       1       arg
...........guacucuacgcuuuugggaGcg.....................................................    2       1       arg
...........gGacucuacgcuuuugggaacg.....................................................    4       1       arg
...........guUcucuacgcuuuugggaacg.....................................................    1       1       arg
...........guGcucuacgcuuuugggaacg.....................................................    2       1       arg
...........guacucuaAgcuuuugggaacg.....................................................    1       1       arg
...........guacucuacgcuuuugggaacA.....................................................    2       1       arg
...........guacucuacgcuuuugggaCcg.....................................................    3       1       arg
...........guacucuacgcuuuugggGacg.....................................................    5       1       arg
...........guacGcuacgcuuuugggaacg.....................................................    1       1       arg
...........guacucuacgcuuuuUggaacg.....................................................    4       1       arg
...........guacAcuacgcuuuugggaacg.....................................................    2       1       arg
...........guacucuacgcuuuugUgaacga....................................................    2       1       arg
...........guacucuacgcuuuugggGacga....................................................    1       1       arg
...........guacucuacgcuuuugggaacgC....................................................    1       1       arg
...........guacucuacgcuuuugggaacgU....................................................    31      1       arg
...........guacucuacgcuuuugggaacga....................................................    57      0       arg
...........guacucuacgcCuuugggaacga....................................................    1       1       arg
............uacucuacgcuuuuggga........................................................    3       0       arg
............uacucuacgcuuuugggaa.......................................................    3       0       arg
............uacucuacgcuuuugggaac......................................................    33      0       arg
............uacucuacgcuuuuggUaac......................................................    1       1       arg
............uacucuacgcuuuugggaacg.....................................................    467     0       arg
............uacucuacgcuuuugggGacg.....................................................    2       1       arg
............uacucuacgcuuuugggaaUg.....................................................    1       1       arg
............uacucuacgcuuuugggaCcg.....................................................    2       1       arg
............uGcucuacgcuuuugggaacg.....................................................    2       1       arg
............uaUucuacgcuuuugggaacg.....................................................    1       1       arg
............uacucuacgcuuuugggaacC.....................................................    2       1       arg
............uacucuacgcuuCugggaacg.....................................................    1       1       arg
............uacucuacgcuuuugggaGcg.....................................................    4       1       arg
............uacAcuacgcuuuugggaacg.....................................................    2       1       arg
............uaAucuacgcuuuugggaacg.....................................................    1       1       arg
............uacucuacAcuuuugggaacg.....................................................    2       1       arg
............uUcucuacgcuuuugggaacg.....................................................    1       1       arg
............uacucuacgcuuuugggaacA.....................................................    1       1       arg
............uacucuGcgcuuuugggaacg.....................................................    1       1       arg
............uacucuacgcuuuuggUaacg.....................................................    3       1       arg
............uacucuacgcuuuuggAaacg.....................................................    1       1       arg
............uacuAuacgcuuuugggaacg.....................................................    1       1       arg
............uacucuacgcuuuugggaUcg.....................................................    1       1       arg
............Gacucuacgcuuuugggaacg.....................................................    1       1       arg
............uacucuacgcCuuugggaacg.....................................................    1       1       arg
............uacucuacgcuuuugggaCcga....................................................    1       1       arg
............uaUucuacgcuuuugggaacga....................................................    1       1       arg
............uacucuacgcuuuuggAaacga....................................................    1       1       arg
............uacucuacgcuuuugggaacga....................................................    547     0       arg
............uacucuacgcCuuugggaacga....................................................    2       1       arg
............uacAcuacgcuuuugggaacga....................................................    1       1       arg
............uacucuacgcuuuugggCacga....................................................    2       1       arg
```

dvi-miR-9699-5p

dvi-miR-9699-3p

caggcacagccguacucuacgcuuuugggaacgagcucuuugacgcacucgguuccauugccguugaguagagcuguccugaugca

............uacucuaUgcuuuugggaacga..	1	1	arg
............uacucuacgcuuuugggaacgU..	10	1	arg
............uacucuacgcuuuugggaacAa..	1	1	arg
............uacucuacgcuuuuCgaacga..	1	1	arg
............uacucuacgAuuuugggaacga..	1	1	arg
............Cacucuacgcuuuugggaacga..	1	1	arg
............uacucuacgcuuuugggaacgG..	1	1	arg
............uacucuacgcuuuuggUaacga..	2	1	arg
............uacucuacgcuuuugggaGcga..	1	1	arg
............uacucuacgcuAuugggaacga..	1	1	arg
............uacucuacgcuuuugggaacUa..	1	1	arg
............uacucuacgcuuCugggaacga..	2	1	arg
............uacCcuacgcuuuugggaacga..	1	1	arg
............uacucuacCcuuuugggaacga..	1	1	arg
............uacucuacgcuCuugggaacga..	2	1	arg
............uacucuacgcuuuuUggaacga..	2	1	arg
............uacucuacgcuuuugggaacgag...	6	0	arg
............uacucuacgcuuuugggaacgaU...	3	1	arg
............uacucuacgcuuuugggCacgag...	1	1	arg
............uacucuacgcuuuugggaacgaC...	1	1	arg
............uacucuacgcuuuugggaacgaA...	5	1	arg
............uacucuacgcuuuugggaacgagc..	1	0	arg
............uacucuacgcuuuugggaacgaUc..	2	1	arg
.............acucuacgcuuuuUggaacg...	1	1	arg
.............acucuacgcuuuugggaacg...	2	0	arg
.............acucuacgcuuuugggCacg...	1	1	arg
.............acucuacgcuuuugggaacgU..	1	1	arg
.............aAucuacgcuuuugggaacgag...	1	1	arg
.............acucuacgcuuuugggCacgag...	1	1	arg
.............acucuacgcuuuugggUacgag...	2	1	arg
.............acucuacgcuuuugggaacUag...	1	1	arg
.............acucuacgcuuuugggaacgag...	22	0	arg
....cucgguuccauugccguugaguag...............	1	0	arg
....ucgguuccauugccguugaguag...............	1	0	arg
....cgguuccauugccguugagu.................	1	0	arg
....cgguuccauugccguugaguag...............	2	0	arg
....gguuccauugccguugagu.................	13	0	arg
....gguuccauugccguugaguC................	2	1	arg
....gguuccauugccguugaguU................	1	1	arg
....gguuccauugccguugagua................	10	0	arg
....ggAuccauugccguugaguag...............	1	1	arg
....gguuUcauugccguugaguag...............	1	1	arg
....gguuccauugccguugaguaA...............	1	1	arg
....ggGuccauugccguugaguag...............	2	1	arg
....gguuccGuugccguugaguag...............	1	1	arg
....gguuccauuAccguugaguag...............	1	1	arg
....gguuccauugccgGugaguag...............	1	1	arg
....gguuccauugccUuugaguag...............	1	1	arg
....gguuccauugccguugaCuag...............	2	1	arg
....gguuccauugccguugGguag...............	1	1	arg
....gguuccauugAcguugaguag...............	3	1	arg
....gguuccauugccguugaUuag...............	1	1	arg
....gguuccauugccguugaguCg...............	2	1	arg
....gUuuccauugccguugaguag...............	4	1	arg
....gguuccauugccguuAaguag...............	1	1	arg
....gCuuccauugccguugaguag...............	1	1	arg
....gguuccauugccguugaAuag...............	1	1	arg
....gguuccauugccguuUaguag...............	1	1	arg
....gguuccauugccguugaguag...............	473	0	arg
....gguuccauuCccguugaguag...............	1	1	arg
....gguuccauugccguugaguUg...............	2	1	arg
....gguucUauugccguugaguag...............	1	1	arg
....gguuccauugccgCugaguag...............	1	1	arg
....gguuccauugccguCgaguag...............	1	1	arg
....gguuccauugccguugagCag...............	1	1	arg
....gguuccauugccguugaguaU...............	1	1	arg
....gguuccauugUcguugaguag...............	1	1	arg
....gguuccauugcUguugaguag...............	3	1	arg
....gguuccauugccguugUguaga..............	5	1	arg

```
            dvi-miR-9699-5p
                                                  dvi-miR-9699-3p
caggcacagccguacucuacgcuuuugggaacgagcucuuugacgcacucgguuccauugccguugaguagagcuguccugaugca

..................................................gguuccauugccguugaguUga..............   1      1    arg
..................................................gguuccaCugccguugaguaga..............   1      1    arg
..................................................gguuccauugcUguugaguaga..............   4      1    arg
..................................................gAuuccauugccguugaguaga..............   2      1    arg
..................................................gguuccauugccUuugaguaga..............   1      1    arg
..................................................gguuccauugccguugaUuaga..............   1      1    arg
..................................................Uguuccauugccguugaguaga..............   1      1    arg
..................................................gguuccauugccguugaCuaga..............   1      1    arg
..................................................gguuccauugccguugaguaga..............   1400   0    arg
..................................................gguuAcauugccguugaguaga..............   2      1    arg
..................................................gguuccauugccguugaguagG..............   3      1    arg
..................................................gguuccauugccguugaguCga..............   5      1    arg
..................................................gguuccauugAcguugaguaga..............   1      1    arg
..................................................gguuccauuUccguugaguaga..............   1      1    arg
..................................................gguuccauugccguugGguaga..............   3      1    arg
..................................................gguuccauugccguugaguaUa..............   5      1    arg
..................................................gguuccauugccguugagCaga..............   4      1    arg
..................................................ggGuccauugccguugaguaga..............   1      1    arg
..................................................gguuccauuCccguugaguaga..............   1      1    arg
..................................................gguuUcauugccguugaguaga..............   1      1    arg
..................................................gguuccauugccguugaguagC..............   5      1    arg
..................................................gguuccauugccgCugaguaga..............   1      1    arg
..................................................gguuccauugccgAugaguaga..............   1      1    arg
..................................................gguuccauugccgGugaguaga..............   1      1    arg
..................................................Aguuccauugccguugaguaga..............   4      1    arg
..................................................gguuccauCgccguugaguaga..............   1      1    arg
..................................................ggAuccauugccguugaguaga..............   1      1    arg
..................................................gguuccauugccCuugaguaga..............   2      1    arg
..................................................gUuuccauugccguugaguaga..............   6      1    arg
..................................................gguuccauugccguCgaguaga..............   5      1    arg
..................................................ggCuccauugccguugaguaga..............   4      1    arg
..................................................gguuccauugccguuAaguaga..............   2      1    arg
..................................................gguCccauugccguugaguaga..............   4      1    arg
..................................................gguuccauugccguugaguagU..............   6      1    arg
..................................................gguuccauugccguugCguaga..............   3      1    arg
..................................................gguuccauugccAuugaguaga..............   1      1    arg
..................................................gguuccGuugccguugaguaga..............   2      1    arg
..................................................gguuccauugccguugaguGga..............   3      1    arg
..................................................gguuccauugccguugagGaga..............   2      1    arg
..................................................gguuccauugccguugaguagaA.............   13     1    arg
..................................................Cguuccauugccguugaguagag.............   1      1    arg
..................................................gguCccauugccguugaguagag.............   1      1    arg
..................................................gguuccauugccguugGguagag.............   1      1    arg
..................................................gguuccauugccguugaguagag.............   33     0    arg
..................................................gguuccauugccguugaguagaU.............   4      1    arg
..................................................gguuccauugccguugaguagaCc............   1      1    arg
...................................................guuccauugccUuugaguag................   1      1    arg
...................................................guuccauugccguugaguag................   14     0    arg
...................................................guuccauugccguugCguaga...............   2      1    arg
...................................................guuccauugccguugaCuaga...............   1      1    arg
...................................................guuccauugccgCugaguaga...............   1      1    arg
...................................................guuccauugccguugaguaga...............   169    0    arg
...................................................guucUauugccguugaguaga...............   1      1    arg
...................................................guuccauugccguugaguagU...............   1      1    arg
...................................................guuccauugccguCgaguaga...............   1      1    arg
...................................................guuccauugccguugGguaga...............   1      1    arg
...................................................gAuccauugccguugaguaga...............   1      1    arg
...................................................guuccauugccguugaguagG...............   2      1    arg
...................................................Uuuccauugccguugaguaga...............   2      1    arg
...................................................guuccGuugccguugaguagag..............   1      1    arg
...................................................Uuuccauugccguugaguagag..............   2      1    arg
...................................................guucUauugccguugaguagag..............   1      1    arg
...................................................guuccauugccguugaguUgag..............   1      1    arg
...................................................guuccauugccguugaguagaA..............   7      1    arg
...................................................guuccauuAccguugaguagag..............   1      1    arg
...................................................guuccauugccguugaguaUag..............   5      1    arg
...................................................gGuccauugccguugaguagag..............   1      1    arg
...................................................gCuccauugccguugaguagag..............   1      1    arg
...................................................guuccauugccguuUaguagag..............   1      1    arg
```

```
           dvi-miR-9699-5p
                                                  dvi-miR-9699-3p
caggcacagccguacucuacgcuuuugggaacgagcucuuugacgcacucgguuccauugccguugaguagagcuguccugaugca

...................................................guuccauugccguugaguagag.............    198    0    arg
...................................................guuccauugccguugaguagaU.............    1      1    arg
...................................................guuccauugccgGugaguagag.............    1      1    arg
...................................................guuccauugccguAgaguagag.............    1      1    arg
...................................................guuccauugccguugaguagagU............    14     1    arg
...................................................guuccauugccguugaguagagA............    9      1    arg
...................................................guuccauugccguugaguagagAu...........    1      1    arg
....................................................uuccauugccguugaguag...............    1      0    arg
....................................................uuccauugccguugaguaga..............    4      0    arg
....................................................uuccauugccguugaguagag.............    2      0    arg
....................................................uuccauugccguugaguagagc............    2      0    arg
....................................................uuccauugccguugaguagagU............    2      1    arg
.....................................................uccauugccguugaguaga..............    3      0    arg
......................................................ccauugccguugaguaga..............    2      0    arg
.......................................................cauugccguugaguagag.............    4      0    arg
```
