# Supplementary material for: A Rapid Evolving microRNA Cluster Rewires Its Target Regulatory Networks in Drosophila
Source: Front Genet. 2021 Oct 28;12:760530. doi: 10.3389/fgene.2021.760530 (PMC8581666; doi:10.3389/fgene.2021.760530)
Supplement: Supplementary file 1 [file DataSheet1.ZIP › Supplementary Figures.docx]

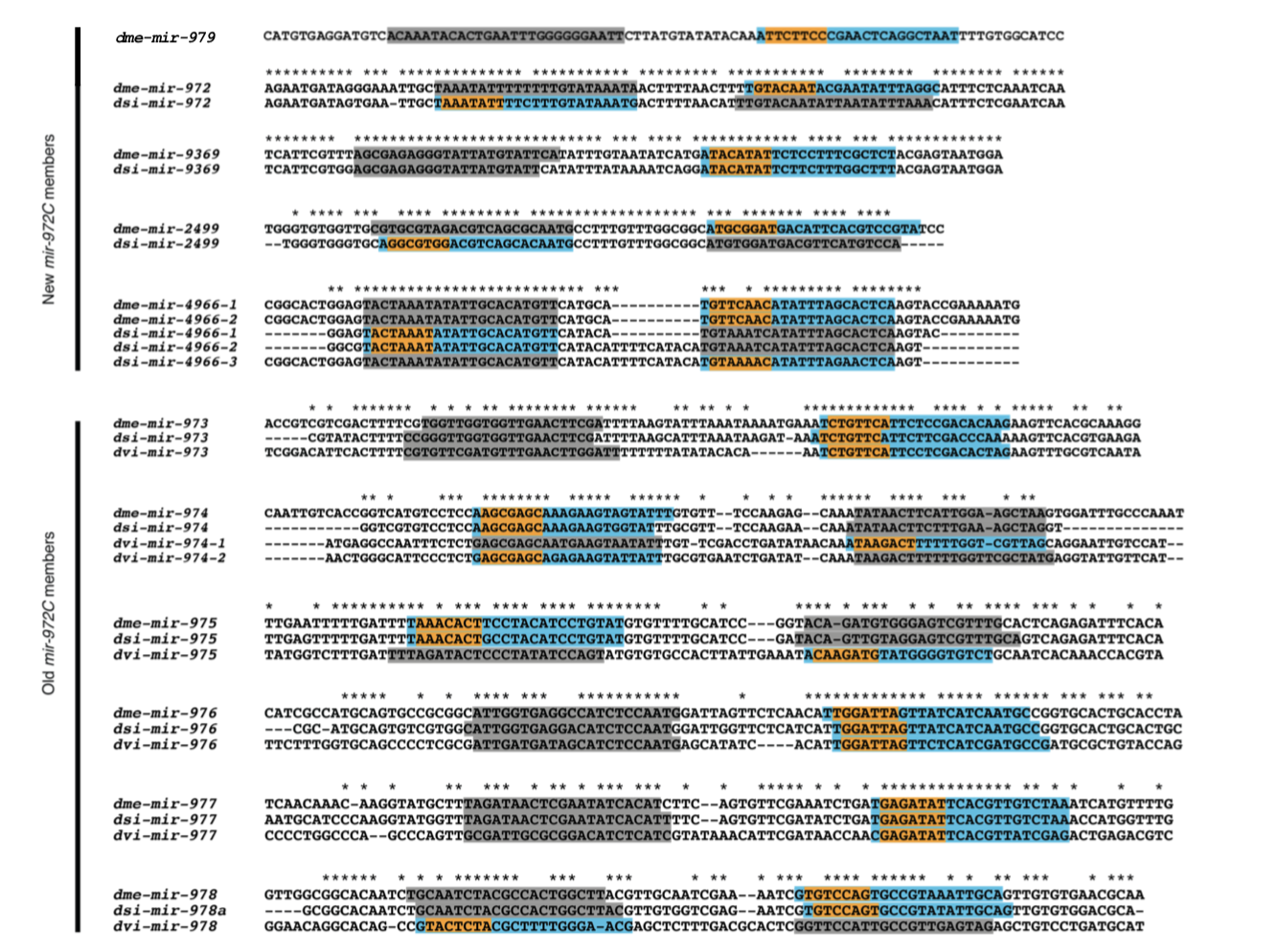


**Figure S1.** *mir-972C* precursor gene sequence divergence among *D. melanogaster, D. simulans*, and *D. virilis*. Mature miRNA and miRNA* sequences are in blue and grey, respectively. Seed regions are highlighted in orange. Species abbreviations: dme, *D. melanogaster*; dsi, *D. simulans*; dvi, *D. virilis*.

**
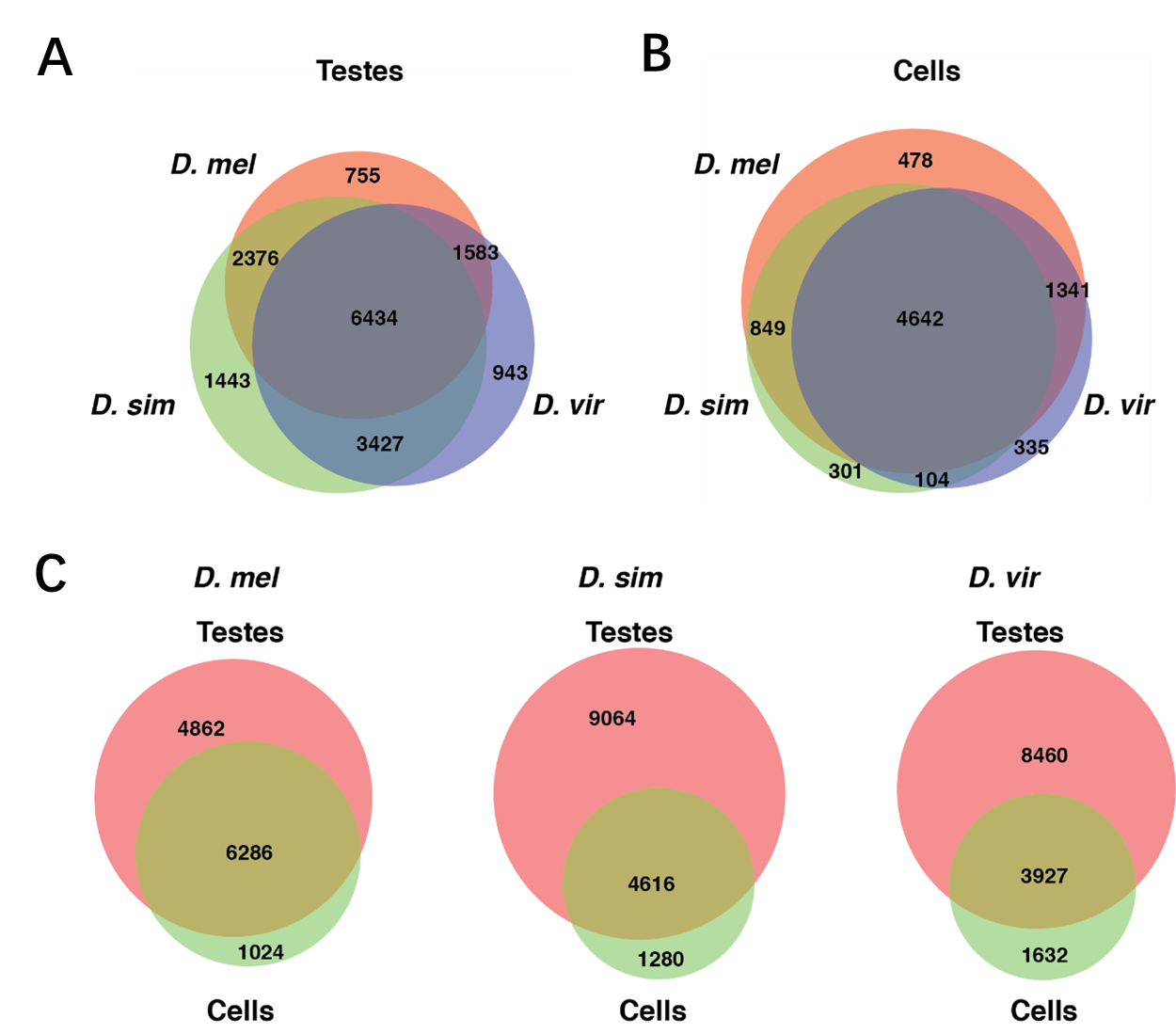
**

**Figure S2**. **(A and B)** Overlaps of genes expressed in the testes **(A)** and in the cell lines **(B)** among the three *Drosophila* species. **(C)** Overlaps of genes expressed between the testes and the cells in the three *Drosophila* species, respectively.


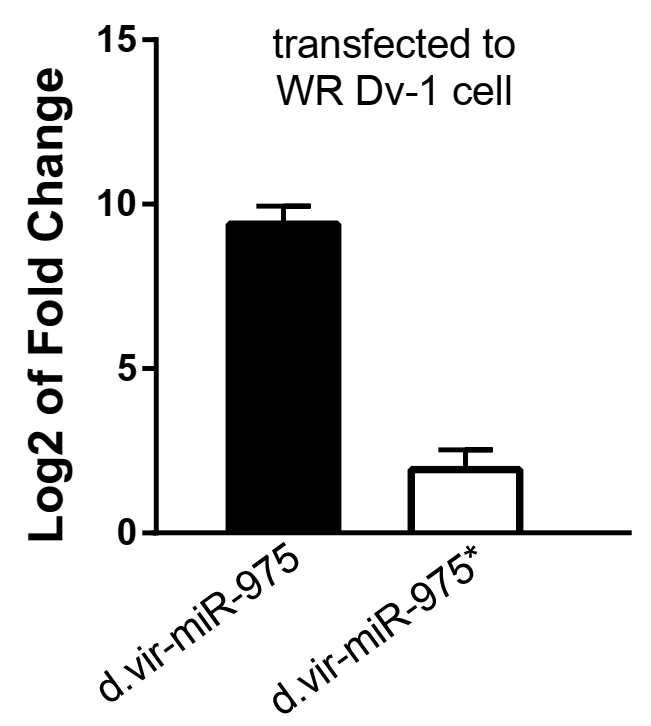


**Figure S3**. Relative miRNA and miRNA* expression levels in *D. virilis* (WR-Dv-1) cell lines. The bar plot shows log2 of fold change, which is calculated by 2^-ΔΔCt^ method.


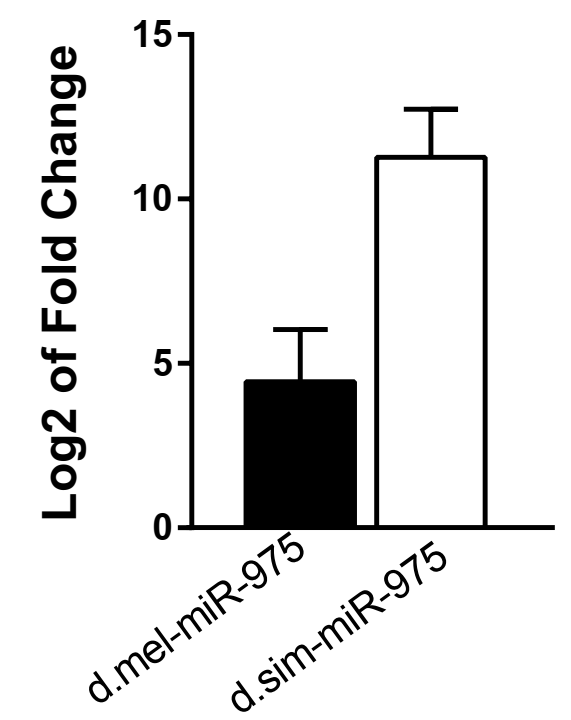


**Figure S4**. Relative dme-miR-975 and dsi-miR-975 expression levels in *S2* cell lines. d*me-mir-975* and *dsi-mir-975* expression vector were respectively co-transfected with *ub-Gal4* vector to *D. melanogaster S2* cells, and qPCR detection was performed 48 hours after transfection. The bar plot shows log2 of fold change, which is calculated by 2^-ΔΔCt^ method.


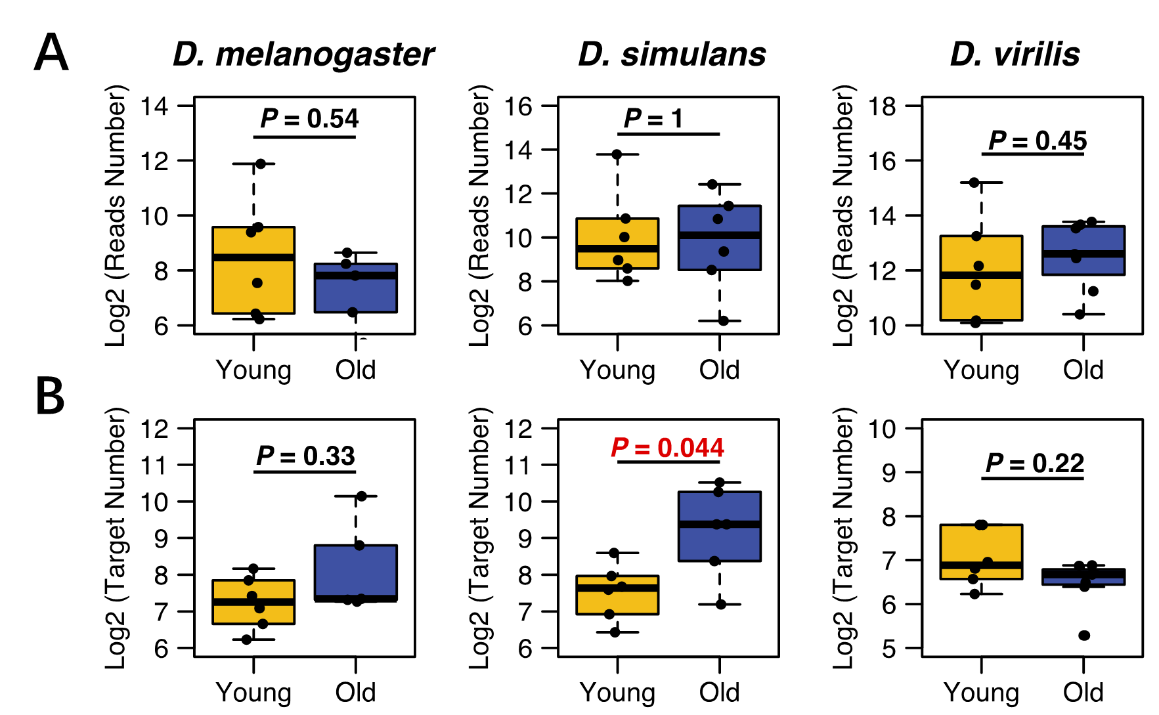


**Figure S5**. Comparison of expression **(A)** and target number **(B)** between the young and the old mir-972C members.
