# Supplementary material for: A Rapid Evolving microRNA Cluster Rewires Its Target Regulatory Networks in Drosophila
Source: Front Genet. 2021 Oct 28;12:760530. doi: 10.3389/fgene.2021.760530 (PMC8581666; doi:10.3389/fgene.2021.760530)
Supplement: Supplementary file 1 [file DataSheet1.ZIP › Supplementary Tables.docx]

**Table S1. The accession numbers of the public deep sequencing libraries used in this study**

| **GEO/SRR accession** | **Species** | **Description** | **Source** |
| --- | --- | --- | --- |
| Small RNA-seq in the testes of *Drosophila* species | | | |
| GSM2562978 | *D. melanogaster* | Canton-S testes | Zhao et al., 2018 |
| GSM1165053 | *D. simulans* | Testes | Lyu et al., 2014 |
| GSM1165055 | *D. pseudoobscura* | Testes | Lyu et al., 2014 |
| GSM548599 | *D. virilis* | Testes from the Argentina strain | Rozhkov et al., 2010 |
| GSM548610 | *D. virilis* | Testes from the 9 strain | Rozhkov et al., 2010 |
| GSM548623 | *D. virilis* | Testes from the 160 strain | Rozhkov et al., 2010 |
| mRNA-seq in the testes of *Drosophila* species | | | |
| GSM838770 | *D. melanogaster* | Testes from the Oregon R strain, replicate 1 | modENCODE project |
| GSM838771 | *D. melanogaster* | Testes from the Oregon R strain, replicate 2 | modENCODE project |
| GSM775508 | *D. simulans* | Testes, replicate 1-1 | modENCODE project |
| GSM775509 | *D. simulans* | Testes, replicate 1-2 | modENCODE project |
| GSM775510 | *D. simulans* | Testes, replicate 2 | modENCODE project |
| SRR5278984 | *D. virilis* | Testes, replicate 1 | Ahmed-Braimah et al., 2017 |
| SRR5278985 | *D. virilis* | Testes, replicate 2 | Ahmed-Braimah et al., 2017 |
| SRR5278986 | *D. virilis* | Testes, replicate 3 | Ahmed-Braimah et al., 2017 |

**Table S2. DAVID functional clustering analysis of predicted targets along various evolutionary branches**

| **ID** | **TERM** | **Enrichment Score** | **Adjusted *p*-value**  **(Benjamini-Hochberg correction)** |
| --- | --- | --- | --- |
| Targets that are shared among *D. melanogaster*, *D. simulans*, and *D. virilis* | | | |
| GO:0007391 | Dorsal closure | 2.92 | 1.0E-04 |
| UP_KEYWORDS | Cyclin | 1.87 | 4.6E-02 |
| UP_KEYWORDS | Glycoprotein | 1.54 | 3.9E-02 |
| Targets that are exclusively shared between *D. melanogaster* and *D. simulans* | | | |
| UP_KEYWORDS | Membrane | 3.18 | 3.3E-02 |
| UP_KEYWORDS | Transcription regulation | 2.55 | 2.7E-02 |
| UP_KEYWORDS | Kinase | 2.12 | 9.1E-03 |
| Targets that are specific to *D. melanogaster* | | | |
| GO:0006468 | Transcription, DNA-templated | 3.81 | 1.1E-03 |
| UP_KEYWORDS | Zinc-finger | 3.19 | 9.2E-03 |
| UP_KEYWORDS | Signal anchor | 2.59 | 2.2E-02 |
| UP_KEYWORDS | ATP binding | 2.35 | 1.9E-02 |
| UP_KEYWORDS | Chromatin regulator | 1.99 | 4.2E-02 |

**Table S3. PCR primers for constructing the miR-975 expression vector**

| **miRNA ^a^** | **Primer** | **Sequence ^b^** |
| --- | --- | --- |
| *dme-mir-975* | Forward | GAAGATCTTCAATACGAAAGTGCGGACCAC |
|  | Reverse | CCGCTCGAGCGGTTGCAGACATTCCAGTGCAT |
| *dsi-mir-975* | Forward | CCGCTCGAGCGGTCGGCACTGGAGTGCTAAAT |
|  | Reverse | TGCTCTAGAGCAGGAGGTTCCAGTGCTTCCT |
| *dvi-mir-975* | Forward | CCGCTCGAGCGGGATAAATCGGCTTTCGTGT |
|  | Reverse | TGCTCTAGAGCAAAAGAACGCACTTCAACGA |

1. Species abbreviations: dme, *D. melanogaster*; dsi, *D. simulans*; dvi, *D. virilis*
2. Restriction sites are indicated in red. They are
   1. dme-mir-975, Forward, BglII
   2. dme-mir-975, Reverse, XhoI
   3. dsi-mir-975, Forward, XhoI
   4. dsi-mir-975, Reverse, XbaI
   5. dvi-mir-975, Forward, XhoI
   6. dvi-mir-975, Reverse, XbaI

**Table S4. qPCR primers for measuring *miR-975* expression**

| **miRNA/sRNA ^a^** | **Primer** | **Sequence** |
| --- | --- | --- |
| dme-miR-975 | RT primer | GTTGGCTCTGGTGCAGGGTCCGAGGTATTCGCACCAGAGCCAACATACAG |
|  | Forward primer | GGCGTAAACACTTCCTACAT |
|  | Reverse primer | GTGCAGGGTCCGAGGT |
|  |  |  |
| dsi-miR-975 | RT primer | GTTGGCTCTGGTGCAGGGTCCGAGGTATTCGCACCAGAGCCAACATACAG |
|  | Forward primer | GGCGTAAACACTGCCTACAT |
|  | Reverse primer | GTGCAGGGTCCGAGGT |
|  |  |  |
| dvi-miR-975 | RT primer | GTTGGCTCTGGTGCAGGGTCCGAGGTATTCGCACCAGAGCCAACAGACAC |
|  | Forward primer | GGCGACAAGATGTATGGGGT |
|  | Reverse primer | GTGCAGGGTCCGAGGT |
|  |  |  |
| dvi-miR-975(*) | RT primer | GTTGGCTCTGGTGCAGGGTCCGAGGTATTCGCACCAGAGCCAACACTGGA |
|  | Forward primer | GGCGTTAGATACTCCCTATA |
|  | Reverse primer | GTGCAGGGTCCGAGGT |
|  |  |  |
| 2s RNA | RT primer | GTCGTATCCAGTGCAGGGTCCGAGGTATTCGCACTGGATACGACTACAAC |
|  | Forward primer | GCGGCTGCTTGGACTACATATGG |
|  | Reverse primer | GTGCAGGGTCCGAGGT |

**Table S5. qPCR primers for measuring the expression of *miR-975* targets**

| Gene | Primer | Sequence |
| --- | --- | --- |
| *laza* | Forward primer | TGAGCATACGGTATCCCTACAA |
|  | Reverse primer | GCGGAAGTAGAGCCTGGTG |
| *Dat* | Forward primer | TGCCCCTACACCATCGAACT |
|  | Reverse primer | GGTAGCGGTTTCAGGGAGT |
| *CG9850* | Forward primer | GCACCACGAGAACAACCACA |
|  | Reverse primer | CAGTTCGCTGTAGTAGGCGTT |
| *Eip74EF* | Forward primer | CATAAAGACGGAGCAAAATACGC |
|  | Reverse primer | CCGCTAAGCAGATTGTGGAG |
| *Gr28b* | Forward primer | ATCAATGGGACACCCGAAGC |
|  | Reverse primer | GGACTCCTGTATAATCTCCGCA |
| *CG2010* | Forward primer | GCCAAGTGCCCCAATTTGAC |
|  | Reverse primer | TCCAGGCCGTTGATGAAGTTG |
| *CG7166* | Forward primer | TGACCAAGTTCACACGGTGG |
|  | Reverse primer | GTGACGGTGCTTCCCTTCC |
| *bab2* | Forward primer | GCGAGTGACTACACAATGGCT |
|  | Reverse primer | AACCTCCCTTTCGGGTGAGT |
| *St1* | Forward primer | CAATGGCGTGGTTCCCAATG |
|  | Reverse primer | TGGGCCTCTTGCTCCAGAT |
| *rp49* | Forward primer | ATGCTAAGCTGTCGCACAAA |
|  | Reverse primer | GTTCGATCCGTAACCGATGT |

**References:**

Ahmed-Braimah, Y. H., Unckless, R. L., and Clark, A. G. (2017). Evolutionary Dynamics of Male Reproductive Genes in the Drosophila virilis Subgroup. *G3 (Bethesda)* 7, 3145–3155. doi:10.1534/g3.117.1136.

Lyu, Y., Shen, Y., Li, H., Chen, Y., Guo, L., Zhao, Y., et al. (2014). New MicroRNAs in Drosophila—Birth, Death and Cycles of Adaptive Evolution. *PLoS Genetics* 10, e1004096. doi:10.1371/journal.pgen.1004096.

Rozhkov, N. V., Aravin, A. A., Zelentsova, E. S., Schostak, N. G., Sachidanandam, R., McCombie, W. R., et al. (2010). Small RNA-based silencing strategies for transposons in the process of invading Drosophila species. *RNA* 16, 1634–1645. doi:10.1261/rna.2217810.

Zhao, Y., Lin, P., Liufu, Z., Yang, H., Lyu, Y., Shen, X., et al. (2018). Regulation of Large Number of Weak Targets—New Insights from Twin-microRNAs. *Genome biology and evolution* 10, 1255–1264. doi:10.1093/gbe/evy079.
